# Augmentation of Positive Valence System–Focused Cognitive Behavioral Therapy by Inaudible High-Frequency Sounds for Anhedonia: A Trial Protocol for a Pilot Study

**DOI:** 10.1001/jamanetworkopen.2019.15819

**Published:** 2019-11-20

**Authors:** Masaya Ito, Mitsuhiro Miyamae, Chika Yokoyama, Yuichi Yamashita, Osamu Ueno, Kazushi Maruo, Asami Komazawa, Madoka Niwa, Manabu Honda, Masaru Horikoshi

**Affiliations:** 1 National Center for Cognitive Behavior Therapy and Research, National Center of Neurology and Psychiatry, Kodaira, Japan; 2 Department of Information Medicine, National Institute of Neuroscience, National Center of Neurology and Psychiatry, Kodaira, Japan; 3 University of Tsukuba, Tsukuba, Japan; 4 National Institute of Mental Health, National Center of Neurology and Psychiatry, Kodaira, Japan

## Abstract

**Importance:**

Recent conceptualizations in Research Domain Criteria have indicated that anhedonia, 1 of 2 core symptoms of depression, which can be treatment resistant, is associated with deficits in the positive valence system, and inaudible high-frequency sound therapy has been shown to enhance reward-related brain circuitry. Hence, cognitive behavioral therapy focusing on the positive valence system enhanced with sound therapy could have a synergistic effect on anhedonia.

**Objective:**

To test the augmentation effect of inaudible high-frequency sounds on the efficacy of positive valence system–focused cognitive behavioral therapy to treat anhedonia.

**Design, Setting, and Participants:**

In this individual-level allocation, exploratory, single-center randomized superiority pilot trial, patients, therapists, and evaluators will be masked to intervention or placebo assignment. The trial will take place at a national psychiatric referral hospital in Tokyo, Japan, among 44 adult patients with clinically significant anhedonia and moderate to severe depression. Outcomes will be analyzed following the intent-to-treat principle using a repeated-measures mixed model.

**Intervention:**

The intervention group will participate in 8 weekly sessions of positive valence system–focused cognitive behavioral therapy with in-session exposure to an inaudible high-frequency sound; the comparison group will undergo cognitive behavioral therapy with in-session exposure to a placebo sound.

**Main Outcomes and Measures:**

The primary outcome is anhedonia assessed using the self-reported Snaith-Hamilton Pleasure Scale. The secondary outcome is anhedonia assessed using the clinician-administered version of the Snaith-Hamilton Pleasure Scale.

**Discussion:**

Recruitment for this study began in May 2018, and the projected date of final allocation is January 2020. A total of 21 eligible patients were registered for participation as of May 30, 2019. To date, treatments for depression do not guarantee clinically successful outcomes. This pilot trial will provide preliminary evidence of the augmentation effect of high-frequency inaudible sounds on cognitive behavioral therapy for anhedonia. Overall, exposure to an inaudible high-frequency sounds does not require attentional or cognitive effort from either patients or therapists; therefore, results from a future confirmative trial could indicate that cognitive behavioral therapy can be augmented in an effortless manner.

**Trial Registration:**

umin.ac.jp/ctr Identifier: UMIN000031948

## Key Points

**Question:**

Can inaudible high-frequency sounds augment the efficacy of cognitive behavioral therapy for anhedonia?

**Findings:**

This trial protocol describes a placebo-controlled, randomized clinical pilot study testing the augmentation effect of inaudible high-frequency sounds on positive valence system–focused cognitive behavioral therapy among patients with clinically significant anhedonia and depression. The sound presentation system is designed to optimize exposure to inaudible high-frequency sounds during face-to-face cognitive behavioral therapy sessions.

**Meaning:**

The preliminary results of this pilot study could contribute to the design of confirmatory randomized clinical trials that will examine the augmentation effect of inaudible high-frequency sounds on the treatment of anhedonia.

## Teaser

This trial protocol describes a pilot study that tests the augmentation effect of inaudible, high-frequency sounds on the efficacy of positive valence system–focused cognitive behavioral therapy (CBT) to treat anhedonia.

## Introduction

Mental disorders cause devastating effects at both the individual and societal level. Among these disorders, major depressive disorder is the leading cause of the global disease burden.^1^ According to treatment guidelines and systematic reviews, cognitive behavioral therapy (CBT) is recommended for patients with depression who experience moderate to severe symptoms.^2^ Nevertheless, the therapies available to treat depression can be improved. A 2010 meta-analysis^3^ showed that the standardized mean effect size of CBT for depression was low (*d* = 0.22) in a high-quality randomized clinical trial. A 2008 meta-analysis^4^ reported that 50% of patients relapsed after completing CBT, and 50% of those who did respond to treatment relapsed within 2 years. To overcome these situations, translational research bridging neuroscience and CBT has focused on developing novel CBT approaches or techniques that augment traditional CBT.^5,6,7^ Among them, we focused on the following 2 noninvasive interventions: (1) positive valence system–focused CBT (PoCot) and (2) inaudible high-frequency sounds. These interventions target the common neurological underpinnings of anhedonia.

Anhedonia, 1 of 2 cardinal depression symptoms, is known to be less responsive to both CBT and pharmacotherapy than other depressive symptoms.^8,9^ Anhedonia is the loss of interest or pleasure associated with deficits in reward-related brain circuity.^10,11,12^ Dysfunction of this circuity includes blunted activation of the striatal regions and orbitofrontal cortex as well as blunted mesolimbic dopamine pathways.^11,13^ It has been suggested that major depressive disorder is characterized by an underestimation of reinforcements received, a reduced expectation of future rewards, less frequent endorsement and recall of positive traits in self-referential tasks, a diminished ability to modulate behavior as a function of reinforcement history, a reduced willingness to make efforts to gain rewards, and an inability to couple the hedonic impact of stimuli and reward assumption.^13^ According to Research Domain Criteria,^14^ anhedonia reflects disturbances of the positive valence system.^15,16^

The positive valence system has been largely neglected in traditional psychotherapeutic techniques, including CBT.^17^ Findings in the field of neuroscience have led to the development of a CBT approach that directly targets the positive valence system; studies have demonstrated the efficacy of this novel CBT approach.^18,19,20,21^ It requires patients with depression to engage in activities that are designed to enhance the subconstructs of positive valence systems (eg, behavioral activation for improving motivation, positive awareness training for improving sensitivity to rewarding stimuli, and savoring training for the loss of pleasure). Craske et al^22^ reported that this positive affect training was more effective for improving anhedonia (ie, increasing positive affect) and reducing a patient’s negative affect and suicidality than more conventional types of CBT (ie, CBT approaches targeting negative affect). Therefore, we believe that the PoCot approach may improve the efficacy of traditional CBT approaches for anhedonia.

The second approach that targets the positive valence system focuses on the hypersonic effect of inaudible high-frequency sounds. Hypersonic effect refers to the whole-body response following exposure to inaudible high-frequency sounds via deep brain activity activation, including reward-related neural circuitry.^23^ Using inaudible high-frequency sounds recorded in a tropical rain forest, our research group previously demonstrated this phenomenon using various noninvasive brain function imaging and physiological measures.^23^ For example, our positron emission tomography study^23^ with healthy control participants demonstrated that inaudible high-frequency sounds, compared with high-cut placebo sounds, increased blood flow in the reward circuit and affected related circuity, including monoaminergic projections distributed across the brain stem, thalamus, and prefrontal region.

Inaudible high-frequency sounds have also been shown to produce higher electroencephalographic (EEG) alpha-band (ie, 8-13 Hz) power.^23^ In addition, in a 2013 study with healthy controls,^24^ simultaneous EEG and functional magnetic resonance image acquisitions indicated that the slow fluctuation component of EEG alpha power was positively associated with blood oxygen level–dependent signal changes in the brain stem, medial thalamus, and anterior cingulate cortex, all of which can be considered part of the reward-related neural circuitry. This observation again suggested that the changes in EEG alpha power induced by inaudible high-frequency sounds may correspond to the activation of reward-related circuitry. Moreover, inaudible high-frequency sounds have been reported to promote approach behavior^25^; this may reflect reward-system activation (eg, better subjective mood or choosing higher levels of sound volume). Overall, inaudible high-frequency sounds have been shown to robustly enhance the subjective experience of sound quality and comfort and to promote approach behavior. Regarding the underlying physiological mechanisms, our previous study^26^ demonstrated that the hypersonic effect may be induced not through the auditory system but through the body’s surface. Although the underlying physiological mechanisms remain unclear, the overall accumulated evidence robustly suggests that inaudible high-frequency sounds enhance the reward system.

We hypothesized that the combination of the PoCot approach and inaudible high-frequency sounds would have a synergistic effect on anhedonia. Although, to our knowledge, no previous direct findings support this idea, PoCot is designed to enhance the brain reward circuity,^18,20^ while inaudible high-frequency sounds increase blood flow.^23^ In addition, reward circuity activation via inaudible high-frequency sounds leads to a relaxed attentional state.^27^ Considering that CBT is a learning process^28^ and positive emotions enhance the learning process,^29^ it is expected that a positive emotional state induced by inaudible high-frequency sounds will facilitate the learning process in CBT. Furthermore, inaudible high-frequency sounds are expected to enhance learning via EEG alpha-band power activation. The alpha-band frequency range inhibits activity in task-irrelevant brain regions, enabling effective task-irrelevant disengagement for information processing.^30^ In addition, inhibiting task-irrelevant memories coupled with alpha-band activity is believed to enhance semantic orientation, allowing individuals “to selectively access stored information that represents the meaning of sensory information and ‘higher order information,’ such as language, mathematics, and geography.”^31^^(p613)^ Hence, we expect that inaudible high-frequency sounds will augment the learning process of CBT via an induced relaxed attentional state and alpha band brain activity.

## Methods

### Trial Design

This trial was designed with an individual-level allocation with a 1:1 ratio. It is an exploratory, randomized clinical superiority phase II trial. Therapists, patients, and evaluators will be masked to allocation. The trial protocol appears in Supplement 1. This report followed the Standard Protocol Items: Recommendations for Interventional Trials (SPIRIT) reporting guideline.

### Study Setting

This study will be conducted at the National Center of Neurology and Psychiatry (NCNP) Hospital in Tokyo, Japan. Patients who are nonresponsive to primary care in their local clinic or hospital are commonly referred to the NCNP Hospital. A detailed description of the setting appears elsewhere.^32^

### Eligibility Criteria

The inclusion criteria are as follows: patient (1) has anhedonia symptoms (Snaith-Hamilton Pleasure Scale [SHAPS] score ≥20), (2) has mild to severe depressive symptoms (GRID-Hamilton depression rating scale score ≥8),^33^ and (3) is aged 18 years or older. The exclusion criteria are as follows: patient has (1) current psychotic disorder, (2) current manic episodes, (3) severe substance use disorder, (4) serious suicidal ideation, (5) severe or unstable physical disorder or major cognitive deficit, and (6) other problems that may present serious obstacles regarding CBT. Exclusion criteria, except for items 5 and 6, will be assessed using the Mini-International Neuropsychiatric Interview version 7.0.2.^34^

### Interventions

#### Positive Valence System–Focused CBT

Both the intervention and control group will participate in PoCot, which will be conducted in a face-to-face, individualized format. Weekly sessions lasting 50 to 60 minutes will be conducted in a room with sound equipment. This intervention was developed using CBT advances that focus on the positive valence system,^18,19,20,21^ positive psychological interventions,^35^ positive emotion regulation interventions,^36^ traditional Japanese psychotherapy that emphasizes positive affect and reward,^37,38^ and findings from neuroscience and emotion research mentioned in the introduction.^29,39^ Biological and theoretical background information and details of the intervention are provided in the eAppendix and eTable in Supplement 2.

Early PoCot sessions will include psychoeducation that emphasizes the importance of the positive valence system for anhedonia treatment. The participants will then begin to practice savoring positive sensations and emotions and attentively monitoring positive events in daily life. Participants will be asked to identify thoughts that interrupt their mindful acceptance of positive sensations and emotions and to examine alternative thoughts. In parallel, the participants will be encouraged to identify their strengths and try to use them in daily life. To help identify strengths, participants will be asked to identify past positive memories.

The focus of the program will then gradually shift from enhancing the patient’s acceptance of short-term positive experiences (ie, short-term reward) to engaging longer-term, goal-directed, and more effortful behavior. Psychoeducation on the temporal discounting of rewards in anhedonia will be provided. The participants will be asked to identify longer-term goals or values in their lives. These goals will then be divided into smaller steps, and patients will be encouraged to engage in easier behaviors. Furthermore, patients will be asked to monitor events they are grateful for in daily life and to practice receiving and providing compliments to enhance positive interpersonal experiences. In the final session, participants will review the skills they acquired during the program and discuss how to maintain a positive lifestyle.

#### Sound Material and Presentation System for Inaudible High-Frequency and Placebo Sounds

The intervention group will be exposed to inaudible high-frequency sounds during PoCot sessions. Rainforest sounds, a natural sound source containing the richest amount of high frequencies with a conspicuously fluctuating structure, were chosen as the sound source for this trial. Sounds containing inaudible high-frequency components (HFCs), referred to as *full-range sounds*, were recorded in the rainforest of Borneo from 2004 to 2009 using a high-speed, 1-bit coding signal processor in a direct stream digital format at a sampling frequency of 5.6 MHz. The electrical signal contains a wealth of HFCs (ie, >100 kHz). The sounds include animal and bird calls, insects chirping, and wind. The track is 47 minutes long and will be presented on repeat.

We selected PoCot without inaudible high-frequency sounds (ie, placebo sounds) as the comparator because previous studies successfully used placebo sounds as a comparator.^23,26^ There was almost no difference in the subjective experience of sound quality between inaudible high-frequency sounds and the placebo sounds. To generate placebo sounds, the inaudible HFCs were excluded from the original sound material using a low-pass filter (digital finite impulse response filter; cutoff frequency, 27 kHz; allowable passband ripple, 0.5 dB; stopband, 28 kHz-150 dB) using the MATLAB signal processing toolbox (MathWorks, Inc).

**Figure 1.**
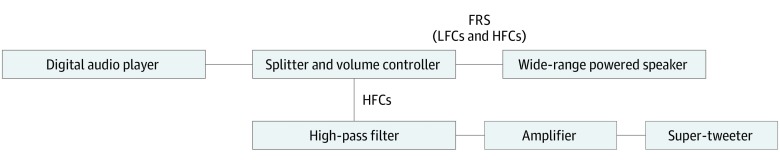
Sound Presentation System In the higher pathway, full-range sound (FRS) sources, which contain high-frequency components (HFCs) and low-frequency components (LFCs), are sent to wide-range, powered speakers (Oohashi Monitor Op. 7; Action Research Co) through a volume controller. In the lower pathway, HFCs are extracted from the sound source after passing through an independent volume controller using Butterworth Bessel high-pass filters (cutoff frequency, 40 kHz, −48 dB per octave) and sent to super-tweeters (PT-R9; Pioneer Co) through a power amplifier (Cerenate; Fidelix). Audible LFCs and HFCs are transmitted via the first pathway, while the inaudible HFCs are presented via the second pathway.

The level of audible sounds should not disturb conversations during PoCot, and the preferred sound level may vary depending on individual participants and therapists. Conversely, the inaudible HFCs should be presented equally to all participants in the intervention group. To this end, we constructed an original sound presentation system whereby the sound source signals recorded in a digital audio player (MR-2000S; KORG) can be divided into 2 pathways by a splitter with an independent volume control for each pathway (Behringer MX882; Behringer Music Group) (Figure 1). Using this presentation system, the fixed level of HFCs can be determined regardless of the audible low-frequency component level.

As the study will use a short-term intervention, all participants will be asked to participate in all 8 sessions. If participants must cancel a scheduled session, an alternative date will be scheduled immediately. Participants will be exposed to their assigned audio track during the entire PoCot session. The sound volume will be set in advance at approximately 55 dB. Participants will sit 2 m away from 2 fixed speakers and tweeters (eFigure 1 and eFigure 2 in Supplement 2).

### Objectives

#### Primary Objective

The primary objective of this trial is to compare the efficacy of PoCot with in-session exposure to inaudible high-frequency sounds vs PoCot with in-session exposure to placebo sounds on anhedonia symptoms. Anhedonia symptoms will be assessed by 44 patients who have been diagnosed with clinically significant anhedonia symptoms using the SHAPS.^40^

#### Secondary Objectives

The key secondary objective is to evaluate the severity of anhedonia symptoms using the clinician-rated version of the SHAPS (SHAPS-C).^41^ Additional outcome variables, including depression, positive emotion, negative emotion, satisfaction with life, and psychological well-being will also be assessed. In addition, a moderation and mediation analysis will be performed using the following 2 measures, which are relevant to the treatment mechanism: (1) perception of environmental rewards and (2) effort-based decision-making. Perception of environmental rewards will be assessed by the Environmental Reward Observation Scale.^42^ Based on behavioral theory, this brief scale was developed to subjectively evaluate environmental reward as an important treatment mediator of traditional and new behavioral activation techniques. Effort-based decision-making will be assessed using the Effort-Expenditure for Rewards Task.^43^ Also, the relationship between the treatment process (eg, homework adherence) and outcomes will be examined.

### Participant Timeline

**Figure 2.**
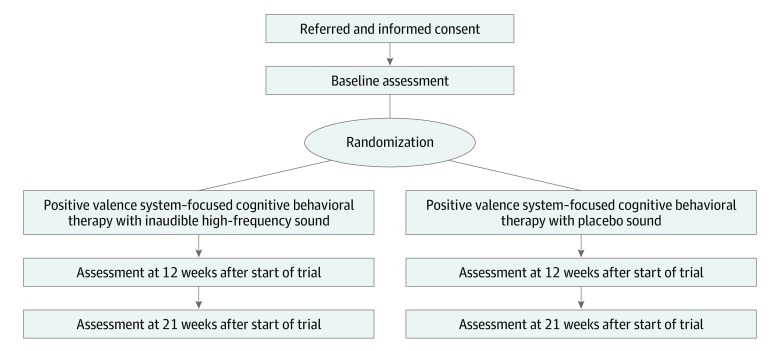
Study Flow Diagram

The timeline is depicted in Figure 2. During the study period, all patients will continue treatment as usual.

### Sample Size

It is difficult to estimate the necessary sample size because previous studies have not directly tested our hypothesis. Among the most studied augmentation strategies of CBT is the effect of D-cycloserine on CBT for anxiety disorders; a meta-analysis^44^ reported that the standardized effect size of the augmentation on various outcomes ranged from 0.07 to 0.58. Based on this range, we conservatively selected a relatively low effect size to assess the augmentation effect of inaudible high-frequency sounds on CBT efficacy. We set a standardized effect size of 0.2 to 0.3 as the clinically significant minimum level. To detect the effect size, a sample size of 344 to 786 participants is needed for the main trial. For this pilot trial, we used the approach that was proposed specifically for calculating sample size for a randomized clinical pilot trial.^45^ Using this strategy, 32 to 72 individuals are needed. Hence, a sample size of 40 is sufficient for this trial. Assuming a 12% dropout rate, we included a total of 44 patients. Before starting the main pilot trial, we will conduct an external preliminary trial on 3 patients to examine the trial’s feasibility.

### Assignment of Interventions

#### Allocation

The research coordinator will register the participants using an electronic data capture system (HOPE eACReSS version 5; Fujitsu). Sequences will be generated by this system before the patients begin the trial (ie, allocation concealment by central registration). The allocation staff will set the audio track (inaudible high-frequency sounds vs placebo sounds) in accordance with the generated sequences. Block randomization without stratification will be used.

#### Masking

All therapists, patients, evaluators, statisticians, and research coordinators will be masked to the allocation. Masking will be checked using the modified version of the Independent Evaluator Knowledge of Outcome^46^ assessment, which was successfully used in a large-scale clinical trial involving CBT.^47^ Using the data, we will calculate the blinding index.^48^

### Data Collection, Management, and Analysis

The Table shows an overall description of the assessment during each visit. Assessment tools include the SHAPS, the SHAPS-C, the GRID-Hamilton Rating Scale, and the Beck Depression Inventory-II.

**Table.** Study Measures and Points

Outcome	Tool	Method	Enrollment			Intervention								After Intervention	Follow-up
			Visit 1	Visit 2	Visit 3	Visit 4	Visit 5	Visit 6	Visit 7	Visit 8	Visit 9	Visit 10	Visit 11	Visit 12	Visit 13
Week	NA	NA	−2	−1	0	1-11								12	21
Diagnosis	MINI	IE		X											
Primary outcome, anhedonia	SHAPS	PSR		X		X	X	X	X	X	X	X	X	X	X
Key secondary outcome, anhedonia	SHAPS-C	IE		X										X	X
Other outcomes															
Depression and anhedonia	GRID-HAMD	IE		X										X	X
	BDI-II	PSR			X					X				X	X
Secondary outcomes															
Emotion	PANAS	PSR			X	X	X	X	X	X	X	X	X	X	X
Life satisfaction	SWLS	PSR			X					X				X	X
Well-being	PWB	PSR			X					X				X	X
Treatment mechanisms															
Anhedonia	EROS	PSR			X					X				X	X
Anhedonia	EEfRT	PSR		X										X	X
Safety	AE	TSR and EA				X	X	X	X	X	X	X	X	X	X
Adherence	HCS	TSR					X	X	X	X	X	X	X		
Blinding	IEKNO	PSR, TSR, and EA				X								X	X

Abbreviations: AE, adverse event; BDI-II, Beck Depression Inventory-II; EEfRT, Effort-Expenditure for Rewards Task; EROS, Environmental Reward Observation Scale; EA, evaluator assessment; GRID-HAMD, GRID-Hamilton Depression Rating Scale 17 Item; HCS, Homework Compliance Scale; IE, interview with evaluator; IEKNO, Independent Evaluator Knowledge of Outcome; MINI, Mini-International Neuropsychiatric Interview version 7.0.2; NA, not applicable; PANAS, Positive and Negative Affect Schedule; PSR, participant self-report; PWB, Psychological Well-being Inventory; SHAPS, Snaith-Hamilton Pleasure Scale; SHAPS-C, Snaith-Hamilton Pleasure Scale, clinician administered; SWLS, Satisfaction With Life Scale; TSR, therapist self-report.

#### Primary Outcome

The SHAPS is a 14-item self-report questionnaire used to assess the presence and severity of anhedonia.^40,49^ Each item is answered by different 4-point Likert anchors. Following methods used in previous studies,^50,51^ the total score will be used. A higher score implies more severe anhedonia symptoms, and a total score greater than 20 is interpreted as having anhedonia. Importantly, the reliability of the Japanese version of the SHAPS has been demonstrated (ie, α = 0.90).^49^

#### Key Secondary Outcome Measure

The SHAPS-C offers improved instructions and anchors compared with the original SHAPS.^41^ Similar to the SHAPS, the SHAPS-C consists of 14 items on a 4-point Likert scale. The SHAPS-C has been reported to have sufficient reliability (α = 0.90) and validity (concurrent validity with SHAPS, *r* = 0.85; *P* < .001).^41^ We translated the English version of the SHAPS-C to Japanese using a rigorous back translation procedure.^52,53^

#### Measures of Eligibility, Other Outcomes, Treatment Mechanisms, and Treatment Processes

The Mini-International Neuropsychiatric Interview^34^ will be used to assess eligibility. The GRID-Hamilton depression rating scale,^33,54,55^ Beck Depression Inventory-II,^56^ Positive and Negative Affect Schedule,^57^ Satisfaction With Life Scale,^58^ and Psychological Well-being Inventory^59^ will be used to evaluate the other outcomes. The Environmental Reward Observation Scale^42^ and Effort-Expenditure for Rewards Task^43^ will be used to assess treatment mechanisms, and the Homework Compliance Scale^60^ will be used to assess treatment adherence. Detailed information on these assessments appears in Supplement 1.

#### Statistical Analysis

##### Outcome Analysis

A mixed model for repeated-measures analysis will be conducted to analyze the primary outcome. The dependent variables in this model will be the participants’ SHAPS scores, assessed during 8 visits from weeks 1 to 11 (ie, intervention period) and at week 12 (ie, postintervention period). The fixed effects will be allocation, visit, allocation-by-visit interaction, and SHAPS score before the intervention (ie, week 0). Covariance will be specified as the unstructured structure. The treatment effects will be presented as the difference in adjusted means between the allocations at week 12 (ie, visit 12). The confidence interval will be estimated, and a *t* test will be conducted to determine the effects of treatment. The treatment effect at other weeks as well as the standardized treatment effect will also be analyzed. As a secondary outcome analysis, analysis of covariance, including baseline measures, will be conducted to test the intervention effects on SHAPS-C scores at week 12. The mixed model for repeated-measures analysis will be conducted for the other outcomes. Statistical significance will be set at *P* < .05, and all tests will be 2-tailed.

##### Analysis Population and Missing Data

The primary and secondary outcome analyses will be evaluated using the intent-to-treat principle. Our secondary outcome analysis will use all registered participants who do not meet the discontinuation criteria, as per the protocol set. The mixed model for repeated-measures analysis will be used to treat missing data. Details of the statistical analyses, including interim analysis and stopping guidelines, are described in the statistical analysis plan and trial protocol (Supplement 1).

### Monitoring

#### Data Monitoring

The primary investigator, in collaboration with the research coordinator and data manager, will conduct on-site and central monitoring and will report the results to the members of the data and safety monitoring board. All registered cases will be subjected to central monitoring. On-site monitoring will be conducted for the first 3 registered cases, then for 3 random, preidentified cases thereafter.

#### Adverse Events

Adverse events will be assessed using the Common Terminology Criteria for Adverse Events.^61^ These include dry mouth, astriction, dysuria, vision dysregulation, orthostatic hypotension, sleepiness, fatigue, sleeplessness, anxiety or agitation, depression or anhedonia, lack of appetite, gain or loss of body weight, loss of sexual desire, palpitations, thrill, diaphoresis, headache, dizziness, and others.

### Ethical Approval and Informed Consent

This study has been approved by the NCNP institutional review board. The initial protocol was approved on November 13, 2017, and the modified version was approved on March 5, 2019. Research coordinators will explain and obtain written informed consent from participants regarding the following items: objectives, reasons for enrollment, the methods and time period, potential burdens, predictable risks and benefits, handling of personal information, means for information storage and disposal, conflicts of interest, any potential invasiveness, and compensation for research-related injuries.

### Confidentiality

The administrator of the patients’ personal information will manage and store all research-related printed or electronic records with personal information, with a corresponding table of personal information and research identification numbers to prevent any information from being divulged, stolen, or lost. The details are described in Supplement 1.

## Discussion

This protocol described the design of our pilot study for testing the augmentation effect of inaudible high-frequency sounds on positive valence system–focused CBT. To date, treatments for depression and/or anhedonia do not guarantee clinically successful outcomes. In contrast to the studies of the augmentation effect of D-cycloserine on CBT for anxiety disorders,^44^ augmentation studies for CBT treatments of depression are limited. Exposure to inaudible high-frequency sounds does not require attentional or cognitive effort from either the patients or therapists; therefore, results from a future confirmative trial could indicate that cognitive behavioral therapy can be augmented in an effortless manner.

### Limitations

Our trial design has several limitations. First, this pilot trial aims to evaluate the potential efficacy of augmenting PoCot with inaudible high-frequency sounds. Because of its preliminary nature, this study only provides information regarding whether it is appropriate to proceed with a larger, definitive trial. Since our sample size calculation is specifically designed to obtain this information, the results of this trial should be cautiously interpreted. Second, considering the limited efficacy of traditional CBT approaches (eg, behavioral activation) and a promising novel neuroscience-driven treatment for anhedonia, we developed PoCot. However, the efficacy of this intervention has not been proven as a stand-alone treatment for anhedonia. Thus, further evidence is needed to test PoCot as a stand-alone treatment for anhedonia. A factorial design would be among the best methods to accomplish this because the use of inaudible high-frequency sound also has limited evidence for its efficacy as a stand-alone treatment for anhedonia. Third, we will not control for patient medications because this would present a potential recruitment barrier. Fourth, we included various outcome and process measures to evaluate the intervention from multiple perspectives; these evaluations should be interpreted as exploratory. Fifth, this trial cannot evaluate or offer detailed information for the following important aspects of the intervention: optimal doses or timing of PoCot and inaudible high-frequency sounds, the augmentation effect in each module of PoCot, specific subgroups that are responsive to PoCot and/or inaudible high-frequency sounds, and the influence of inaudible-high frequency sounds on several distinct reward processes.

### Conclusions

This trial is expected to provide initial findings on the synergistic effect of psychological interventions that target the positive valence system and use inaudible high-frequency sounds. The combination of CBT and sound therapy could have a synergistic effect on anhedonia.

## Notes

Supplement 1. **Trial Protocol**

Supplement 2. **eAppendix.** Biological and Theoretical Rationale and Treatment Descriptions of Positive Valence System–Focused Cognitive Behavioral Therapy **eFigure 1.** Speaker and Super-Tweeter **eFigure 2.** Layout of the Therapy Room **eTable.** Description and Findings Related to Positive Valence System–Focused Cognitive Behavioral Therapy Treatment Modules for Anhedonia **eReferences.**
