# Supplementary material for: Augmentation of Positive Valence System–Focused Cognitive Behavioral Therapy by Inaudible High-Frequency Sounds for Anhedonia: A Trial Protocol for a Pilot Study
Source: JAMA Netw Open. 2019 Nov 20;2(11):e1915819. doi: 10.1001/jamanetworkopen.2019.15819 (PMC6902816; doi:10.1001/jamanetworkopen.2019.15819)
Supplement: Supplement 1. — Trial Protocol [file jamanetwopen-2-e1915819-s001.pdf]

Trial Protocol and Statistical Analysis Plan

Supplemental text 1

IRB approved study protocol, 3rd version (February 3, 2019)

Supplemental text 2

IRB approved study protocol, 2nd version (February 20, 2018)

Supplemental text 3

IRB approved study protocol, 1st version (September 6, 2017)

Supplemental text 4

Statistical analysis plan for the augmentation of positive valence system-focused cognitive behavior therapy by inaudible high-frequency sound: A placebo-controlled randomized trial (First revised version: May 23, 2019)

研　究　計　画　書
Study Protocol

第 3 版：2019 年 2 月 3 日

Third version: 3rd, February, 2019

研究課題名	**アンヘドニアに対するポジティブ価システムに焦点を当てた認知行動療法の超高周波音響療法による増強効果：プラセボ対照ランダム化比較試験**
Research title	**Augmentation of positive valence system-focused cognitive behavior therapy by inaudible high-frequency sound therapy: A placebo-controlled randomized trial**
研究責任者（所属）	**伊藤正哉（認知行動療法センター研修指導部）**
Principal Investigator	**Masaya Ito, Ph.D（National Center of Cognitive-Behavior Therapy and Research, National Center of Neurology and Psychiatry）**

1. 研究の名称

1. Research title

アンヘドニアに対するポジティブ価システムに焦点を当てた認知行動療法の超高周波音響療法による増強効果：プラセボ対照ランダム化比較試験

Augmentation of positive valence system-focused cognitive behavior therapy by inaudible high-frequency sound therapy: A placebo-controlled randomized trial

2. 研究の実施体制

2. Site-specific research information

【国立精神・神経医療研究センターにおける共同研究者】

【Study members in National Center of Neurology and Psychiatry】

**氏名 Name**	**所属 Affiliations**	**研究における役割及び責務 Roles and responsibilities**	**倫理講座の受講の有無（1 年以内） Experience in research ethics workshop participation**	**本研究に関する利益相反申告状況 Status of conflict of interest declaration**
伊藤正哉 Masaya Ito	認知行動療法センター National Center of Cognitive-Behavior Therapy, National	研究責任者、研究デザイン、認知行動療法の開発と実施	あり Yes	2017 年 9 月 6 日提出済 Submitted on 6th of September, 2017

	Center of Neurology and Psychiatry	Principal investigator, study design, development of cognitive behavioral therapy protocol and implementation		
堀越勝<br>Masaru Horikoshi	認知行動療法センター<br>National Center of Cognitive-Behavior Therapy, National Center of Neurology and Psychiatry	研究デザイン<br><br>Study design	あり<br><br>Yes	2017 年 9 月 6 日提出済<br>Submitted on 6th of September, 2017
本田学<br>Manabu Honda	神経研究所<br>National Institute of Neuroscience, National Center of Neurology and Psychiatry	音響プロトコルの開発と運用<br>Development of protocol and management of audio intervention	あり<br><br>Yes	2017 年 9 月 6 日提出済<br>Submitted on 6th of September, 2017
山下祐一<br>Yuichi Yamashita	神経研究所<br>National Institute of Neuroscience, National Center of Neurology and Psychiatry	音響プロトコルの開発、音響機器のメンテナンス 割付の順番作成<br>Development of audio intervention protocol, maintenance of audio devices, generation and storage of comparison table of randomized sequences and sound tracks	あり<br><br>Yes	2017 年 9 月 6 日提出済<br>Submitted on 6th of September, 2017

宮前光宏<br>Mitsuhiro Miyamae	神経研究所<br>National Institute of Neuroscience, National Center of Neurology and Psychiatry	研究デザイン、認知行動療法の開発と実施、症状評価、附属研究の主任研究者<br>Study design, development of cognitive behavioral therapy protocol and implementation, symptom assessment, PI for ancillary study	あり<br><br>Yes	2017年9月6日提出済<br>Submitted on 6th of September, 2017
上野修<br>Osamu Ueno	神経研究所<br>National Institute of Neuroscience, National Center of Neurology and Psychiatry	音響プロトコルの開発と運用、音響機器のメンテナンス<br>Development of protocol and management of audio intervention, maintenance of audio devices	あり<br><br>Yes	2017年9月6日提出済<br>Submitted on 6th of September, 2017
横山知加<br>Chika Yokoyama	認知行動療法センター<br>National Institute of Neuroscience, National Center of Neurology and Psychiatry	認知行動療法の開発と実施、試験コーディネート<br>Development of cognitive behavioral therapy protocol and implementation, coordination of trial	あり<br><br>Yes	2017年9月6日提出済<br>Submitted on 6th of September, 2017
伊藤まどか<br>Madoka	精神保健研究所成人精神保健研究部・流動研究員	認知行動療法の開発<br>Development of	あり<br><br>Yes	2017年9月6日提出済<br>Submitted on 6th

Ito	National Institute of Mental Health, Neuroscience, National Center of Neurology and Psychiatry	cognitive behavioral therapy protocol		of September, 2017
丸尾和司<br>Kazushi Maruo	トランスレーショナル･メディカルセンター<br>Translational Medical Center, National Center of Neurology and Psychiatry	解析計画書の作成と、解析の実施<br>Development of statistical analysis plan and statistical analysis	あり<br><br>Yes	2017年9月6日提出済<br>Submitted on 6th of September, 2017
駒沢あさみ<br>Asami Komazawa	認知行動療法センター<br>National Institute of Neuroscience, National Center of Neurology and Psychiatry	認知行動療法の開発と実施<br>Development of cognitive behavioral therapy protocol and implementation	あり<br><br>Yes	2017年9月6日提出済<br>Submitted on 6th of September, 2017

【国立精神・神経医療研究センターにおける共同研究者以外の研究協力者の実施体制】

【Personnel external to National Center of Neurology and Psychiatry】

**氏名**<br>**Name**	**所属・役職**<br>**Affiliations**	**研究における役割及び責務**<br>**Roles and responsibilities**	**倫理講座の受講の有無（1年以内）**<br>**Experience in research ethics workshop participation**	**本研究に関する利益相反審査結果**<br>**Status of conflict of interest declaration**
中島俊<br><br>Shun Nakajima	認知行動療法センター<br><br>National Institute of Neuroscience, National Center of Neurology and Psychiatry	個人情報管理者・個人情報匿名化担当者<br>Administrator of anonymization management	あり<br><br>Yes	提出予定<br>To be Submitted

【効果安全性評価委員会】

【Members of Data Safety Monitoring Board】

所属<br>Affiliation	氏名<br>Name	専門<br>Expertise
武蔵野大学<br>Musashino University	中島聡美<br>Satomi Nakajima, M.D., Ph.D.	精神医学<br>Psychiatry
東京大学<br>Tokyo University	西大輔<br>Daisuke Nishi, M.D., Ph.D.	精神医学<br>Psychiatry

【モニタリング】

【Monitoring】

所属<br>Affiliations	氏名<br>Name	研究における役割及び責務<br>Roles and responsibilities
認知行動療法センター<br>National Center of Cognitive-Behavior Therapy, National Center of Neurology and Psychiatry	伊藤正哉<br>Masaya Ito	モニタリング責任者<br>Responsible for monitoring

3. 研究の背景、科学的合理性の根拠及び社会的意義

3. Research background, scientific validity, and social significance

精神疾患が個人と社会に及ぼす損失は甚大である。なかでも、うつ病は世界の疾病負担の 3 位である（Vos et al., 2015）。精神疾患はわが国の 5 大疾病のひとつであり、最も患者数が多い（約 323 万人）。うつ病と不安障害の 1 年間の時点有病率は 7.9%である。うつ病の 1 年間の社会経済コストは年間 3 兆 900 億円に上る。

認知行動療法(Cognitive Behavioral Therapies; CBT)は、学習理論や認知理論に基づき、治療手続きが体系化された精神療法の総称である。診療ガイドラインや系統的レビューによると、中等症以上のうつ病(大うつ病性障害、持続性抑うつ障害)には、認知行動療法が推奨されている（e,g., 英国医療技術評価機構（National Collaborating Centre for Mental Health）, 2010）。日本においても、うつ病に対する認知行動療法が健康保険適用となった。現在の日本では、認知行動療法をいかに均てん化するかについての努力が展開しているところである。

Mental disorders cause devastating effects at both the individual and societal level. Among them, depressive disorder ranks third as a cause of global disease burden (Vos et al., 2015). Mental disorders are designated as one of the big-five diseases in Japan and together constitute the largest patient population among these big-five diseases (more than 3 million and 230,000 patients in Japan). The annual prevalence of depressive and anxiety disorders in Japan is reported to be 7.9%. The annual socio-economic cost of depressive disorder is estimated to be more than 3 trillion yen.

Cognitive behavioral therapy (CBT) is a form of psychotherapy with systematic intervention

protocols based on learning and cognitive theories. According to treatment guidelines and the results of systematic reviews, cognitive behavioral therapy is recommended for treating depression with moderate or severe symptoms (major depressive disorder and dysthymia) (e.g., National Institute of Clinical Excellence, 2010). In Japan, CBT for depression was subjected to national medical insurance beginning in 2013. There is a current focus to increase the use of CBT in the Japanese medical setting.

うつ病治療において、いまや認知行動療法は第一治療選択のひとつである。しかし、依然として改良の余地も大きい。認知行動療法を受けても約半数が再発したり、治療反応を示さない患者も一定数いることが知られている。そうした背景から、認知行動療法の効果を増強させる先端研究が展開されつつある。例えば、d-cycloserine という物質（もともとは結核薬）を認知行動療法の前に投与することにより、不安症に対する認知行動療法で行われる"情報の再学習"が強化される可能性が期待されている。他にも、増強を検討するさまざまな研究が芽生えつつある（治療中の記憶力の増強（Harvey et al., 2016）。

CBT is currently the first-line treatment for depression. Nevertheless, there is significant scope for improvement. Approximately half of patients show relapse of depression after the completion of CBT. A substantial proportion of patients do not respond to CBT. Based on these shortcomings, there have been increased research efforts to augment the effect of CBT for depression. For example, d-cycloserine, originally utilized for treating tuberculosis, is used to augment "information re-learning" during CBT for anxiety disorders. Other techniques for augmenting the efficacy of CBT have also been used, such as augmentation of memory technique (Harvey et al., 2016).

われわれは、認知行動療法を増強させる方法として、"アンヘドニアに対するポジティブ価システムに焦点を当てた認知行動療法"と"ハイパーソニック・エフェクト（超高周波音響効果, Hypersonic Effect）"に着目した。まず、ポジティブ価システムに焦点を当てる認知行動療法とは、うつ病の中でもアンヘドニア症状を改善することに焦点を当てた認知行動療法である。アンヘドニア症状は従来の治療では改善されにくい症状であり、ポジティブ感情の低下、意欲の減退、報酬に対する感受性の低下、歓びの喪失などの様々な定義が与えられている。近年提唱された研究領域基準（Insel et al., 2010）においては、このアンヘドニア症状はポジティブ価システム（Positive-Valence System）の失調として捉えられる。ポジティブ価システムの下位概念として、接近動機づけ、報酬獲得への初発反応性、報酬獲得への反応性の維持と長期反応性、報酬学習、習慣が含まれている。近年の認知行動療法は、神経科学とポジティブ心理学の知見を導入し、これらのシステムを特に強化するための介入を導入し、うつ病治療の改善を検証しつつある（Craske et al., 2016, Taylor et al., 2016, Alexopoulos et al., 2016, Blom et al., 2016）。

We focused on the "positive valence system-focused CBT for anhedonia" and "hyper-sonic

effect” (effect of inaudible hyper sound) for this study. Positive valence system-focused CBT is a CBT that focuses on improving anhedonia. Anhedonia is defined as decreased positive emotions, decreased motivation for performing activities, deceased sensitivity to rewarding stimuli, or loss of pleasure. Anhedonia is known to be resistant to conventional treatments for depression. In the context of Research Domain Criteria (Insel et al., 2010), anhedonia can be classified as a dysfunction of the positive valence system. Sub-constructs of the positive valence system are constituted by approach motivation, initial responsiveness to reward attainment, sustained/longer-term responsiveness to reward attainment, reward learning, and habit. Recent studies on CBT have attempted to incorporate findings from neuroscience and positive psychology to augment these systems and test their efficacy (Craske et al., 2016; Taylor et al., 2016; Alexopoulos et al., 2016; Blom et al., 2016）.

一方で、ハイパーソニック・エフェクトとは、人間の可聴域上限を超える超高周波成分を豊富に含む音響情報が、報酬系神経回路を含む脳深部の神経活動を活性化し、人体に全身的影響を及ぼすことを指す。これまで研究協同者（本田学）の研究グループは、この現象を複数の非侵襲脳機能イメージングと様々な生理活性指標を用いて明らかにしてきた（Oohashi et al., 2000 他）。具体的には、超高周波成分を豊富に含む音情報は、同じ音から超高周波成分を除去した音情報（ハイカット音）と比較して、脳幹、視床から前頭前野に拡がるモノアミン作動性神経投射を含む情動系神経回路や報酬系回路の血流を増加させるとともに、それと並行して脳波 α 波のパワーを増強させること、視床下部の活性化を反映して NK 細胞活性を上昇させ、ストレスホルモンを低下させるといった全身反応を導くことを明らかにした。加えて、超高周波成分を含む音情報は、音質を向上し音の快適性を増強させるとともに、超高周波成分を豊富に含む音をより多く受容しようとする接近行動を引き起こすなど、報酬系の活性化を反映した被呈示者の心理行動的効果を導く。

The hyper-sonic effect refers to the whole-body effect from exposure to inaudible high-frequency sound via the activation of deep brain activity including reward related neural circuits. Our research group has demonstrated the phenomenon using various non-invasive brain function imaging and physiological measures (Ohashi et al., 2000). For example, we have demonstrated that inaudible high-frequency sound, in comparison to high-cut placebo sound, increases blood flow in the reward circuity and affects related circuity including monoaminergic projections distributed across the brain stem, thalamus, and prefrontal region, in parallel with enhancement of alpha brain-wave power. Compared to high-cut placebo sound, inaudible high-frequency sound enhanced natural killer cell activation, which reflects hypothalamic activity and decreased stress hormone levels. In addition, inaudible high-frequency sound promoted psycho-behavioral effects that reflect activation of the reward system. Therefore, inaudible high-frequency sound seems to robustly enhance the subjective experience of sound quality and

comfort, and promotes approach behavior to listen to the sound.

　こうした複雑に変化する超高周波成分は、人間の遺伝子が進化的に形成されたと考えられる熱帯雨林の環境音に豊富に含まれる一方で、現代人の多くが生活する都市環境音にはほとんど含まれない。そこで研究分担者らは、自然環境音に豊富に含まれ現代社会の環境音にほとんど含まれない複雑性をもった超高周波成分という「必須情報」の不足が、深部脳を起点とするモノアミン神経系の変調を介して、気分障害をはじめとする精神・神経疾患の発症に無視できない影響を及ぼす可能性があるのではないかとの仮説を立てた。この仮説のもと、平成 22〜24 年度の厚生労働科研費医療技術実用化総合研究事業では、超高周波成分を豊富に含む音響情報の曝露の前後で、うつ病患者の状態不安指標が有意に改善することを示した。

Although such inaudible high-frequency sound with complex, changing sound quality is prevalent in environmental sounds in tropical rain forests, it is absent in urban city settings in which most modern people around the world live. We hypothesized that inaudible high-frequency with complex sound quality could be conceptualized as “essential information”, and the lack of this could have considerable effects on the onset of mental and neurological disorders via the dysfunction of monoaminergic neural systems. Based on this hypothesis, we have demonstrated that the exposure to sound information with inaudible high-frequency sound significantly improved state anxiety among patients with depression.

　このように、ハイパーソニック・エフェクトは様々なイメージング・生理・心理・行動指標で観察されてきた。なかでも今回われわれが注目したメカニズムとして、うつ病患者の精神症状の改善効果と、学習効果の増強に関する知見を指摘できる。前者においては、超高周波音響の呈示によって、脳内の報酬系回路が活性化させることにより、「必須情報」が補完された結果として解釈できる。認知行動療法においても、報酬系回路を活性化させる治療要素（行動活性化）が含まれているため、そうした治療要素との相乗効果が期待できる。さらに、ハイパーソニックの学習効果増強に関する研究では、超高周波音響を呈示されながら実施される認知課題（N-back 課題）では、呈示がない場合よりも優れた成績が示された。先に述べた通り、認知行動療法は“情報の再学習”を治療媒介とする。そのため、ハイパーソニック・エフェクトによる学習効果の増強は、認知行動療法の効果増強にも応用できると考えられる。

This hyper-sonic effect has been observed in various imaging, physiological, psychological, and behavioral measures. Among these, we focused on two findings of the hyper-sonic effect: the improvement of mental status among patients with depression, and the enhancement of learning. The former could be interpreted as a supplement of “essential information” via activation of brain reward circuity by exposure to inaudible high-frequency sound. One of the intervention techniques

in CBT, behavioral activation, is aimed at activating the reward circuitry. Therefore, it is expected that inaudible high-frequency sound and behavioral activation may have a synergistic effect. Furthermore, the latter findings showed that performance on a cognitive task (N-back task) was superior in groups exposed to inaudible high-frequency sound compared to groups not exposed to the sound. As discussed above, one of the treatment mechanisms of CBT is “re-learning of information.” Hence, enhancement of learning performance by the hyper-sonic effect may be applicable for augmenting CBT.

4. 研究の目的及び意義

4. Research objectives and significance

上述の背景から、本研究ではポジティブ価システムに焦点を当てた認知行動療法と超高周波音響を併用することにより、うつ症状のなかでもとくにアンヘドニアが顕著に改善されるかどうかを検討することを目的とした。すなわち、本臨床試験は、アンヘドニア症状を呈する成人 44 名を被験者対象集団とし、ポジティブ価システムに焦点を当てた認知行動療法に超高周波音響を呈示した試験治療の、同治療に超高周波を含まないプラセボ音響を呈示した対照治療に対する、Snaith-Hamilton Pleasure Scale にて測定されるアンヘドニア症状への有効性に対する優越性を検証することを目的とする。

Based on the rationale discussed above, we aimed to test the efficacy of combining the positive valence system-focused CBT with inaudible high-frequency sound to improve anhedonia. The objective of this clinical trial is to test the efficacy of the positive valence system-focused CBT with inaudible high-frequency sound compared to the positive valence system-focused CBT with placebo sound on anhedonia symptoms (Snaith-Hamilton Pleasure Scale) among 44 patients with anhedonia.

本研究では、さまざまな認知行動療法の中でも、アンヘドニアに対するポジティブ価システムに焦点を当てた介入技法がハイパーソニック・エフェクトにより増強されるかどうかを検証する。もしその増強効果が同定されれば、今後はより大規模な検証的ランダム化比較試験に進む。同時に、様々な疾患（e.g., 全般不安症、社交不安症、パニック症、心的外傷後ストレス障害など）や認知行動療法の他の治療要素（e.g., モニタリング、認知再構成、エクスポージャー、マインドフルネス）への適用へと拡張させて増強効果を検証する。検証的試験により、パイパーソニック・エフェクトによる増強効果が確認できれば、これまで認知行動療法に反応しなかった患者や、再発を呈していた患者に対して、新たな治療選択肢を提示することができるようになる。

Among the various forms of CBT, we will test the augmentation effect for intervention techniques that are focused on the positive valence system for anhedonia. If augmentation effects are observed in this study, we will proceed to a larger confirmatory randomized controlled trial. In

parallel, we will extend research on this augmentation effect for various disorders (e.g., generalized anxiety disorder, social anxiety disorder, panic disorder, posttraumatic stress disorder, etc.) and various treatment components of CBT (e.g., monitoring, cognitive restructuring, exposure, and mindfulness). Confirming the augmentation of CBT by the hyper-sonic effect will enable new treatment choices for patients who do not respond to CBT or who experience relapse following the completion of CBT.

本研究で増強効果が観察できた場合には、その科学的根拠は多方面に重要なインパクトを持つ。有効な精神療法は認知行動療法以外にも指摘されている。また、そもそも薬物療法などの“物質”療法においても、医療者と患者間のコミュニケーション、すなわち“情報の相互伝達”を基盤として医療が行われる。このように、他の精神療法や、医療コミュニケーションにおける情報伝達と学習の効率化にハイパーソニックが寄与できる可能性への扉が開かれることになる。

If we are able to prove an augmentation effect, this will have impact on various fields. Effective psychotherapy is not limited to CBT. Pharmacotherapy is also based upon the communication or reciprocal exchange of information between physicians and patients. This study may open the door to the potential contribution of the hyper-sonic effect on information exchange in medical communication and learning efficiency.

また、本研究は先端的電子情報技術というわが国の強みを最大限に活用したアプローチをとる。音響療法を含む情報技術を応用した統合医療という大きな未来性を持つ学術・産業領域を、わが国先導の下に世界に提案するとともに、電子情報通信産業やメディア産業など、異分野から医療分野への効果的で摩擦の少ない参入を促すことが期待される。

This study adopts an approach that fully utilizes advanced electronic information technology, which is one of Japan’s strong points. This study takes the initiative of promoting integrative medicine utilizing information technology including sound therapy as highly promising academic and industrial endeavors. Furthermore, this study is expected to effectively promote the integration of the electronic information technology and media industries with the medical field.

5. 研究の方法及び期間

5. Research methods and timeline

（1）研究実施期間

（1）Research timeline

倫理委員会承認後から 2020 年 3 月 31 日まで

（研究対象者登録締切予定日：2019 年 5 月 31 日）

（倫理委員会承認後から 12 月にかけて、3 症例に対して、本研究のフローに従った予備試行を行う。その上で、必要に応じて変更申請を行う。）

From the date of IRB approval to 31st March, 2020

(Planned date of the final registration of participant, 31st May, 2020)

(After the date of IRB approval, we will conduct three pilot trial cases that is followed by procedure of the main trial to confirm the feasibility. If needed, we will submit the modified protocol to IRB.)

（2）研究の種類・デザイン

（2）Trial design

個人割り付け、治療介入、探索的、無作為化、マスキング（治療者、患者）、並行群間比較、プラセボ対照、単施設、第 II 相試験

Individual-level allocated, treatment intervention, exploratory, randomized, therapist and patient masked, parallel group, placebo controlled, single-site, phase II trial

（3）予定する研究対象者数

（3）Targeted sample size

本研究は先行研究が存在せず、事前に症例数設計で用いる適切な効果量を推定することが困難である。本研究で狙いとする認知行動療法の増強効果という点で参考になる文献として、不安症に対する認知行動療法の d-cycloserine による増強効果のメタアナリシスが報告されている。この報告では、諸種のアウトカムでの増強効果の基準化効果量が 0.07〜0.58 であった（Ori et al., 2015）。この範囲の中でも、本研究の増強効果では比較的小さい効果サイズを想定するのが妥当であると考えられる。そこで、臨床的意義のある最小限の基準化効果量を 0.2〜0.3 と設定し、パイロット RCT の精度に基づく症例数設計手順(Cocks & Torgerson, 2013)から、1 群 20 例とした。これにうつ病に対する認知行動療法の脱落率 12.1% を考慮して、本研究では 2 群で計 44 例を目標症例数として設定した。なお、この症例数には、予備試行で行われる 3 症例を含めない。

平成 30 年 4 月－平成 32 年 1 月までの 34 ヶ月間に、毎月 2–3 例程度の登録を予定している。

It is difficult to estimate the appropriate sample size to be used as there are no previous studies directly related to this trial. It would be informative to refer to the results of a meta-analysis of the augmentation effect of d-cycloserine on CBT for anxiety disorders. It was reported that the standardized effect size of the augmentation effect on various outcomes ranged from 0.07–0.58 (Ori et al., 2015). Based on this range, we conservatively selected a relatively low effect size for the augmentation effect of inaudible high-frequency sound on the efficacy of CBT. Hence, we set the

standardized effect size as 0.2–0.3 as the clinically significant minimum level. Following the procedure of sample size estimation for pilot randomized controlled trial (Cocks & Torgerson, 2013), we set the sample size per group as 20. Considering the reported proportion of drop-outs (12.1%), we set 44 as the targeted total sample size. Before starting this main pilot trial, we will conduct an external preliminary trial with three patients to examine the feasibility of this trial.

We estimate a registration of two to three patients per month from April 2018 to January 2020.

（4）研究のアウトライン

（4）Research outline

本研究への参加は計 21 週間であり、組入れ期間が-4–0 週、介入期間が 1–11 週、介入後評価が 12 週、追跡評価が 21 週に実施される。タイムラインを Fig.1（次ページ）に示す。この間、患者は通常の診療を続ける。本研究に関係する部分は、Fig.1 にあるように、説明と同意、評価、登録、介入 8 セッション、である。

Participation in this study is for 21 weeks: enrolment period from -4 to 0-week, intervention period from 1 to 11-week, post-intervention assessment at 12-week, and follow-up assessment at 21-week. The timeline is depicted in Fig. 1. During the study period, all patients will continue treatment as usual. Activities related to this trial are informed consent, assessment, registration, and 8 session intervention (Fig. 1).

本臨床試験の広報は、認知行動療法センターのホームページおよび病院内に設置したパンフレットを通して行う。国立精神・神経医療研究センター病院に外来通院する患者を対象として、患者本人の参加希望があれば、主治医と相談してもらうよう広報する。主治医の許可が得られれば、主治医から精神リハビリテーション部の認知行動療法初診に紹介していただくようにする。認知行動療法初診後に、臨床心理室で開かれているカンファレンスにおいて、本研究への紹介が妥当と判断されれば、本研究への紹介となり、患者とコンタクトをとり、説明と同意へと進む。

This clinical trial will be advertised on the National Center of Cognitive-Behavior Therapy and Research website and with pamphlets placed in the hospital. This study is intended to recruit outpatients in the NCNP hospital. If patients are willing to participate in the trial, we encourage the patient to first consult their main doctors. After permission has been obtained from their doctors, they will be referred to the intake for CBT at the department of psychiatric rehabilitation in the NCNP hospital. After the intake, treatment indications, including referral to this study, will be examined at weekly meetings attended by staff including psychiatrists and clinical psychologists in the department. If the patient is subsequently referred to this study, the research coordinator will contact the patients to proceed with informed consent.

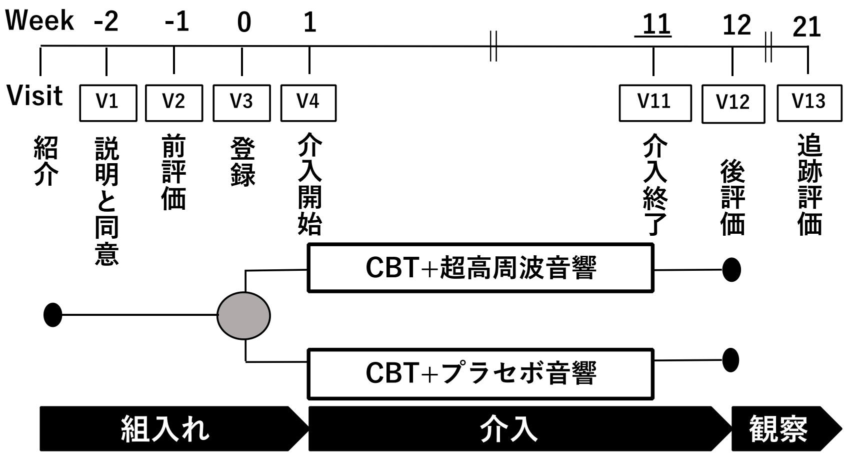

Fig.1 試験のタイムライン

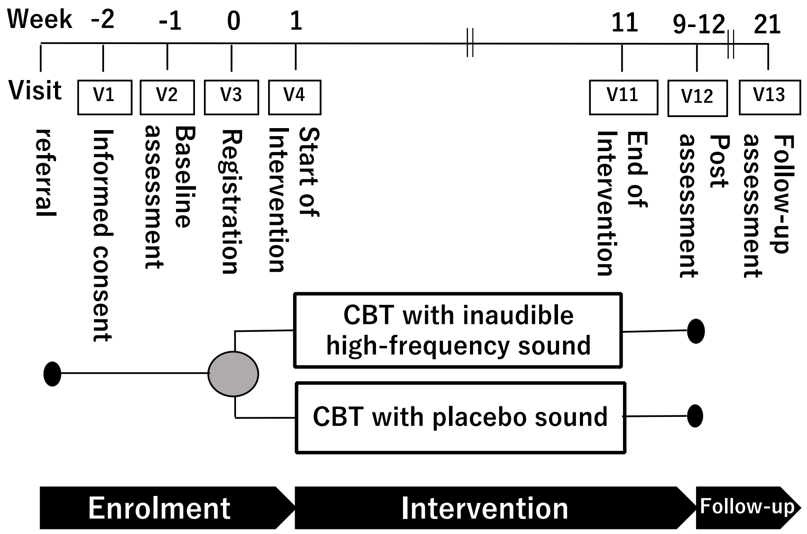

Fig.1 Timeline of the trial

（5）研究に用いる医薬品・医療機器、治療法等の情報

（5）Medicinal drugs, devises, or interventions for this study

アンヘドニアに対するポジティブ価システムに焦点を当てた認知行動療法(**Positive-valence system focused Cognitive-Behavioral Therapy for Anhedonia; PoCot**)：

ポジティブ価システムに焦点を当てたアンヘドニアに対する認知行動療法(**Positive-valence system focused Cognitive-Behavioral Therapy for Anhedonia; PoCot**)は、うつに対する認知行動療法の中でも行動活性化で用いられる技法を土台としつつ、近年のポジティブ価システムに介入する内容（Craske et al., 2016, Taylor et al., 2016,

Alexopoulos et al., 2016, Blom et al., 2016）を参考に開発したものである。理論的な背景としては、報酬系に関する神経科学(Pizzagalli, 2014)、精神病理学(Pelizza & Ferrari, 2009; Franken et al., 2007)、そして感情調整研究（Carl et al., 2013）を基盤としている。基本的な治療原理は、うつ病という病態が報酬系情報システムが機能していない状態であるという理解のもとに、報酬系に関わるシステムが活性化されるような体験に従事することで、報酬系システムとそれが関与するうつという病態が改善されるという仮定に立っている。治療内容は下記の(6)に示す通りである。

Positive valence system-focused Cognitive-Behavioral Therapy for Anhedonia; PoCot：

The PoCot is developed based on the behavioral activation for depression and recently proposed interventions for positive valence system (Craske et al., 2016; Taylor et al., 2016; Alexopoulos et al., 2016; Blom et al., 2016). Theoretically, this program is based on the neuroscience of the reward system (Pizzagalli, 2014), psychopathology underlying anhedonia (Pelizza & Ferrari, 2009; Franken et al., 2007), and emotional regulation (Carl et al., 2013). The central treatment rationale is based on the concept of depression as a dysfunction of the reward-related information system (i.e., positive valence system). It is assumed that reward-related systems may be improved by engaging rewarding behaviors and fully experiencing positive emotions.

超高周波音響とプラセボ音響：

超高周波音響は、人の可聴域上限をこえる超高周波成分を豊富に含む熱帯雨林の自然環境音を用いる。プラセボ音響は、超高周波音響と同一の音源から高周波成分のみを取り除いた音響を用いる。音響提示装置には研究参加者自身では操作できない安全装置（操作パネルカバーなど）を装備し、装置の制御は実験スタッフが行う。音響情報の種類については、これまでの健常人を対象とした一連の実験室実験や、市街地で多数の健常者を対象とした実験、外来うつ病患者を対象とした実験などで使用実績があり、問題が生じていない音源を、本研究の目的のために特別に編集して用いる。音響情報の呈示音量は、これまでの実験同様、室内の会話の支障にならない低音量とし、その最適音量を検討する。

Inaudible high-frequency sound and placebo sound:

We will use natural environmental sounds that have been proven to include many inaudible high-frequency sounds for auditory intervention. We will exclude the inaudible high-frequency sound component from the same natural environmental sound source, and use this as placebo sound. The operational panel of sound equipment will be concealed to prevent participants from touching and/or manipulating it. Operation of the sound equipment will be conducted by the therapist. The sound source has been used in laboratory experiments for healthy populations, large scale experiments in urban districts, and experiments for outpatients with depressive

disorder. No adverse events or related problems were reported in these studies. Similar to previous studies, the volume of sound will be low to avoid disturbing the natural conversation in the room.

（6）試験薬の用法・用量、投与方法又は試験機器の適用方法

（6）Dosage and administration of trial medicine or method of trial device

アンヘドニアに対するポジティブ価システムに焦点を当てた認知行動療法：

対面での個人療法の形式をとった、ポジティブ価システムを強化する認知行動療法を行う。毎週 60 分、計 8 回のセッションを、国立精神・神経医療研究センターの音響装置が設定された部屋にて行う。60 分のセッションを毎週、計 8 回、個人療法として、訓練を受けた臨床心理士や医師が実施する。治療内容としては、初期のセッションでは、ポジティブ価システムが大切な理由を患者が理解できるように心理教育を行う。その上で、ポジティブな感覚や感情を５感で味わうための訓練や、ポジティブな出来事に注意を向けるためのモニタリングを開始する。さらには、ポジティブな感覚や感情を感じることを妨害する認知を同定し、代わりとなる認知を検討する。このようにして、日々の生活体験の中でポジティブな体験を重ねるとともに、本人にとってのポジティブな側面（強みや趣味）を同定し、それを意識したエクササイズを行う。このようにして、短期的なポジティブ体験（短期的報酬）への感受性と高めた上で、長期的な目標に向けた行動に従事するモジュールにとり組む。アンヘドニアの状態では、長期報酬への割引効果（※時間や労力のかかる目標は価値を置かれにくく行動が生起にくくなる）について心理教育を行った上で、本人にとっての長期的目標や価値を同定する。その上で、本人の長期目標のために取りうる行動の段階に分けて検討し、日々の生活において実施しやすい行動から取り組む。さらに、人間関係におけるポジティブ体験を強化するために、日々の対人的に感謝できる出来事を記録するとともに、コンプリメントを与え受けるという課題に取り組む。最終セッションでは、介入を通して学習した取り組みを継続するための方法を確認し、終結となる。

PoCot の介入遵守については、全 44 例の８回のセッションのうち 10 分の１の 35 セッション分をランダムに抽出し、セッション早見表に基づくアドヒアランス評価尺度を用いて、当該患者の担当セラピストではない治療者が評価を行う。

Positive Valence System-focused Cognitive-Behavioral Therapy for Anhedonia

The PoCot will be conducted in a face-to-face, individual format. Sixty-minute weekly sessions will be conducted in a room with sound equipment in the National Center of Neurology and Psychiatry. A trained clinical psychologist or physician will administer the

PoCot. The earlier PoCot sessions will include psychoeducation emphasizing the importance of the positive valence system to recover from depression. The participant will then begin to practice savoring positive sensations and emotions using the five senses, and in monitoring the enhancement of attention to positive events in daily life. Participants will be requested to identify thoughts that interrupt the mindful acceptance of positive sensations and emotions, and to try to examine alternative thoughts. These practices are intended to promote accumulation of positive experiences in daily life. In parallel, the participant is gradually encouraged to identify and exercise their own positive aspects (e.g., strength and avocation). The focus of this program gradually shifts from enhancing the acceptability of short-term positive experiences (short-term reward) to engaging longer-term, goal-directed behavior. Psychoeducation on temporal discounting of reward in anhedonia will be provided. The participant will be asked to identify longer-term goals or values in their life. The goal will be divided into smaller steps and patients encouraged to engage in easier behaviors. Furthermore, patients will be asked to monitor events for which they are grateful in daily life, and to practice receiving and providing compliments to enhance positive interpersonal experiences. In the final session, participants will review the skills learned in the program and examine the ways of maintaining such activities.

Adherence for PoCot will be evaluated by the other therapist stuff using the adherence evaluation scale. We will sample the 35 sessions randomly (10 percent of all planned 8 sessions for 44 participants.

超高周波音響とプラセボ音響呈示のための音源と音響機器：

熱帯雨林の音響—豊富な超高周波構造の音響を含む—が実験音響源として選定された。この音響には可聴不能な超高周波音響（HFC）が含まれており、2004 年から 2009 年に掛けてボルネオ島の熱帯雨林において、5.6MHz のサンプリング周波を有するダイレクトストリームデジタル（DSD）フォーマットによる超速、1 ビット符号シグナルプロセッサーにより録音された音響である。この電子音響は、100kHz 以上に達する豊かな高周波を含んでいる。本音響は動物や鳥類の鳴き声、昆虫の鳴き声、風の音等を含んでいる。47 分のトラックを繰り返し呈示される。プラセボ音響では、オリジナル音源素材に含まれる超高周波音響のみが、MATLAB (The MathWorks, Inc. U.S.A)による信号処理ツールボックスを伴うローパスフィルター(digital FIR filter, cut-off frequency 27kHz, allowable passband ripple 0.5dB, stopband 28kHz -150dB)を用いて削除された。

音響の水準は CBT における対話を阻害しない程度とする。しかし、好まれる水準は参加者やセラピストに依存すると考えられる。これに対応するため、オリジナルの音響呈示システムを構築し、デジタル・オーディオプレイヤー(KORG MR-2000S)により録音された信号が 2 つの経路に分割され、分割された音量がそれぞれ別個にコントロールできるようにした(Behringer MX882, MUSIC Group IP Ltd., Virgin Islands, British)（Fig. 1）第一の経路では、FRS へ

の音量コントローラーを通したワイドレンジパワードスピーカー(OOHASHI MONITOR Op. 7, Action Research Co., Ltd., Tokyo, Japan)に音源が送られる。第二の経路では、独立した音量コントローラを通過した後に、HFC のみがバッターワースベゼルハイパスフィルター(cut-off frequency 40kHz, -48 dB/oct.)により抽出され､それがスーパーツイーター(PT-R9, Pioneer Co., Ltd., Kanagawa, Japan)にパワーアンプ(CERENATE, FIDELIX Co., Ltd., Tokyo, Japan)を通して送られる。それにより、音源が FRS のときに、低周波要素と高周波要素が第一の経路で呈示され、高周波のみが第二の経路で呈示される。本呈示システムを用いることにより、予め定められた高周波音響が、低周波音響の音量に関わらず呈示することができる。

超高周波音響への曝露は、音響担当により(YY, SK)、ウルトラサウンド検知器(D240X, Pettersson Elektronik AB, Sweden)を用いて、月に一度確認される。セラピストが音響トラックを切り替える際には、セラピストは自身の操作を録画し、適切なトラック番号と音量等を確認する；

本研究では短期間の介入であるため、すべての参加者はすべてのセッションに参加することを求められる。もし参加者が予定されたセッションをキャンセルしなければならない場合､即座に代替セッションを調整する｡参加者は PoCot のセッションにおいて録音音源に､50-60 分、暴露される。音量は予め 55 デシベルに設定される。参加者は固定されたスピーカーとツイーターからおよそ 2 メートル離れた着席する（写真１,2）。

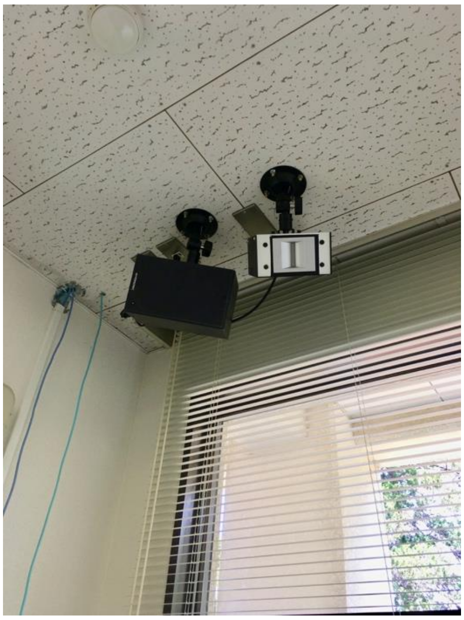

写真 **1.** 部屋に設置されたスピーカーとツイーター

Photo 1. Speaker and Tweeter sat in the therapy room

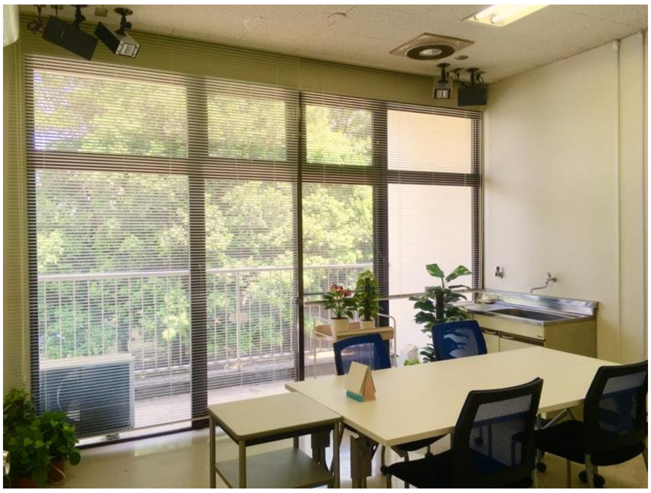

写真 **2.** 治療部屋

Photo 2. Therapy room

Sound material and sound presentation system for inaudible high-frequency sound and placebo sound:

Rain forest sounds, a natural sound source containing the richest amount of high frequencies with a conspicuously fluctuating structure, were chosen as the sound source for the experiments. Sounds containing inaudible high-frequency components (HFC) referred to as full range sounds (FRS) were recorded in the rain forest of Borneo island from 2004 through 2009 with a high-speed, one-bit coding signal processor in Direct Stream Digital (DSD) format having a sampling frequency of 5.6 MHz. The electrical signal contained a wealth of high-frequency components, even reaching 100 kHz and above. This sound contains calls of animals and birds, chirping of insects, sound of winds etc. The 47 minutes track will be repeatedly presented. As placebo sound, only the inaudible HFCs were excluded from the original sound material by using a low pass filter (digital FIR filter, cut-off frequency 27kHz, allowable passband ripple 0.5dB, stopband 28kHz -150dB) with the signal processing toolbox with MATLAB (The MathWorks, Inc. U.S.A).

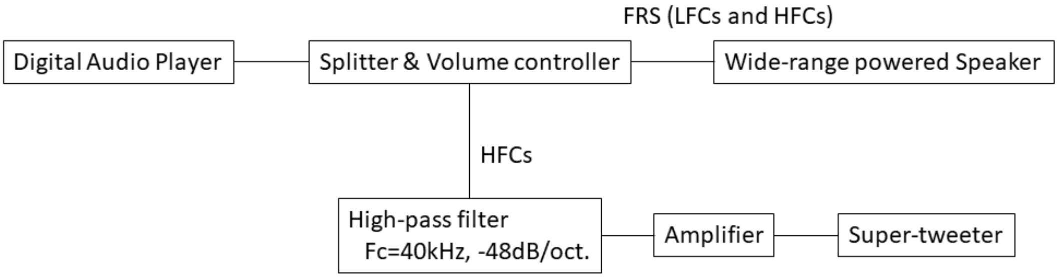

Figure 1. Sound presentation system

Level of audible sounds should not disturb conversations in CBT. However, preferred level of sounds may vary depending on individuals of participants and therapists. On the other hand, in order to expose inaudible HFCs of sounds should be stably exposed to all participant. In order to achieve this, we constructed an original sound presentation system where sound source signals recorded in a digital audio player (KORG MR-2000S) were divided into two pathways by a splitter with an independent volume controller for each pathway (Behringer MX882, MUSIC Group IP Ltd., Virgin Islands, British) (Fig. 1). In the first pathway, sound sources were just sent to wide-range powered speakers (OOHASHI MONITOR Op. 7, Action Research Co., Ltd., Tokyo, Japan) through a volume controller for FRS. In the second pathway, after passing through an independent volume controller, only HFCs were extracted from the sound source with Butterworth Bessel high-pass filters (cut-off frequency 40kHz, -48 dB/oct.) and sent to super-tweeters (PT-R9, Pioneer Co., Ltd., Kanagawa, Japan) through a power amplifier (CERENATE, FIDELIX Co., Ltd., Tokyo, Japan). As a result, when sound source is FRS, both audible low frequency components (LFCs) and HFCs were presented via the first pathway, and HFCs were presented via the second pathway. Using this presentation system, predetermined fixed level of HFCs can be presented regardless of the level of audible LFCs.

Exposure of inaudible high-frequency sound will be checked once in a month by sound management stuff (YY, SK) by using the Ultrasound Detector (D240X, Pettersson Elektronik AB, Sweden). When therapist operate the sound panel to switching on the audio track, the therapist video record the her/him operation and confirm the track number and knob for volume etc.

As short-term intervention will be employed, all participants will be requested to participate in every eight session. If participants have to cancel a scheduled session, an alternative date will be scheduled immediately. Participants will be exposed to their assigned audio track during the PoCot session, which will be 50–60 minutes. Sound volume will be set in advance as about 55 decibel. Participants will sit 2 meter from the two fixed speakers and tweeters (Photo.1).

用量・スケジュール変更規準

Criteria for changes in dose or frequency

本研究では短期の介入を取るために、全８セッションへの参加を被験者に求める。止むを得ずセッションのキャンセルが生じた場合には、遅滞なく日程の再調整を行う。

As short-term intervention will be employed, all participants will be requested to participate in every eight session. If participants have to cancel a scheduled session, an alternative date will be scheduled immediately.

（7）併用薬・併用療法についての規定

（7）Regulation for concurrent drugs or treatments

向精神薬の併用がある場合には、介入期間中には薬剤の種類と用量を一定にするよう、本人及び主治医の同意を予め得ることとする。他に、電気けいれん療法と構造化された精神療法は研究期間には実施しないよう依頼する。

If the participant takes any type of psychotropic medicine, we will obtain consent from the participant and their primary doctors to keep the dose and types of medicine consistent throughout the study period. In addition, we will request that participants refrain from receiving electroconvulsive therapy and/or other systematic psychotherapy during the study period.

（8）評価項目、評価方法

（8）Outcomes and assessment method

主要評価項目は、１週から 12 週にかけて毎週 Snaith–Hamilton Pleasure Scale 日本語版（SHAPS）で測定されるアンヘドニア症状である。

副次評価項目は、12 週に測定される Snaith–Hamilton Pleasure Scale-clinician administered(SHAPS-C)で測定されるアンヘドニア症状である。

The primary outcome is anhedonia symptoms assessed by the Snaith-Hamilton Pleasure Scale from week 1 to 12.

The secondary outcome is anhedonia symptoms assessed by the Snaith-Hamilton Pleasure Scale-clinician administered at week 12.

(9)　観察及び検査項目

(9)　Observation or examination items

観察・検査・調査項目

Observation, examination, or survey items

1. 基礎情報：性別、生年月日、婚姻状況、年収、就学・就労状況、病名（主治医による診断）、主訴、登録以前までの治療歴（心理相談含む）、精神科既往歴、教育歴、家族歴、飲酒・喫煙
2. 宿題遵守：治療の一環として宿題の実施遵守を評価する。
3. 併用治療の遵守状況：Visit 毎に患者に確認するとともに、可能な場合はカルテ記載を随時確認することにより、薬物療法の安定性を確認する。
4. アンヘドニア症状：SHAPS 日本語版、SHAPS-C
5. うつ症状の重症度：GRID-HAMD, BDI-II

6. 精神疾患の有無：MINI（除外基準、併存疾患の確認）
7. 他のアウトカム指標：PANAS, SWLS, PWB
8. 治療メカニズム：EROS, EEfRT
9. 有害事象の確認：Visit 毎に口頭および所定の用紙にて確認する。
10. 治療プロセス：HC
11. 盲検化：IEKNO

1. Basic information: Gender, date of birth, marital status, income, current education and/or employment, diagnosis by primary doctor, chief complaints, treatment history, psychiatric history, educational history, family history, alcohol use, smoking.
2. Homework compliance: Homework compliance will be assessed as part of treatment.
3. Compliance for concurrent treatment: Consistency of pharmacotherapy will be checked at every visit. Where possible, the research coordinator will check it via electronic medical charts.
4. Anhedonia: Japanese version of SHAPS, SHAPS-C
5. Depression: GRID-HAMD, BDI-II
6. Presence or absence of mental disorders: MINI (confirmation of exclusion criteria and concurrent disorders)
7. Other outcomes: PANAS, SWLS, PWB
8. Treatment mechanism: EROS, EEfRT
9. Adverse events: Will be checked at every visit by oral questioning and a self-report sheet.
10. Treatment process: HC
11. Masking: IEKNO

観察・検査・調査スケジュール

Schedule for observation, examination, or survey

					ENROLMENT			INTERVENTION								POST	FU
TIME POINT (Week)					-2	-1	0	1–11								12	21
Visit					V1	V2	V3	V4	V5	V6	V7	V8	V9	V10	V11	V12	V13
(Burden for patient (minutes))																	
ENROLLMENT :																	
	Informed Concent			(50)	X												
	Randomization			(5)			X										
	Intake			(60)			X										
ASSESSMENTS :																	
Diagnosis		MINI	IE	(25)		X											
Primary Outcome	Anhedonia	SHAPS	Pt	(4)		X		X	X	X	X	X	X	X	X	X	X
Secondary Outcome	Anhedonia	SHAPS-C	IE	(10)		X										X	X
Other Outcome	Depression/Anhedonia	GRID-HAMD	IE	(30)		X										X	X
		BDI-II	Pt	(5)			X					X				X	X
	Emotion	PANAS	Pt	(3)			X	X	X	X	X	X	X	X	X	X	X
	Life Satisfaction	SWLS	Pt	(2)			X					X				X	X
	Well-being	PWB	Pt	(6)			X					X				X	X
Treatment Mechanism	Anhedonia	EROS	Pt	(3)			X					X				X	X
		EEfRT	Pt	(20)		X										X	X
Safety	Adverse Event	AE	Th	-				X	X	X	X	X	X	X	X	X	X
Adherence	Homework Compliance	HC	Th	-					X	X	X	X	X	X	X		
Blinding		IEKNO	Pt/Th/IE	(2)				X								X	X
Burden for Participants(minutes)					(50)	(89)	(84)	(9)	(7)	(7)	(7)	(23)	(7)	(7)	(7)	(83)	(83)

Fig 2. Overall description of the measures and its timepoints.

IE: Interview with Independent Evaluator, Pt: Participant self-report, Th: Therapist self-report

上記の項目は調査スケジュール表（Fig.2）に基づき、試験施設にて検査する。調査結果を CRF に記載する。規定された観察日（検査日）のずれの許容範囲は±7 日とする。

The items above will be assessed at the trial site following the Examination Schedule Table. Results of the examination will be filled in CRF. An acceptable range of dates is one week before and after the scheduled date.

（10） 実施する検査について

(10) Assessment

1. 精神疾患の診断（Mini International Neuropsychiatric Interview 7.0.0）：

   MINI は、DSM-5 の主要な精神疾患や臨床状態を診断するための簡易構造化面接法である(Sheehan et al., 1998)。本研究では、DSM-5 診断を評価するために最新版の MINI 7.0.0 を使用する。評価項目は、抑うつエピソード、うつ病/大うつ病性障害、自殺念慮、自傷及び自殺行動、自殺行動障害、躁病エピソード、軽躁病エピソード、双極 I 型障害、双極 II 型障害、双極性障害、特定不能のもの、精神病性の特徴を伴う双極 I 型障害、パニック症/パニック障害、広場恐怖症、社交不安症/社会不安障害（社交恐怖）、強迫症/強迫性障害、心的外傷後ストレス障害、アルコール使用障害、物質使用障害（非アルコール）、精神病性障害、精神病性の特徴を伴う気分障害、神経性やせ症/神経性無食欲症、神経性過食症/神経性大食症、過食性障害、全般性不安症/全般性不安障害、医学的、器質的および薬物関連の病因を除外、反社会性パーソナリティ障害であり、各診断基準について「はい」「いいえ」で回答する。短時間で施行可能である（18.7±11.6 分、中央値 15 分）。旧版については、日本版についても信頼性と妥当性が確認されている(Otsubo et al., 2005)。妥当性については、Structured Clinical Interview for Diagnostic and Statistical Manual-III-R-patient version との基準関連妥当性が示されており（Kappa > .49)、信頼性については高い評者間一致度が報告されている（Kappa > .72)。

1. Diagnostic status of mental disorders (Mini International Neuropsychiatric Interview 7.0.0):

   The MINI is a brief structured interview for assessing major mental disorders and clinical status (Sheehan et al., 1998). We will use the updated 7.0.0 version. The MINI assesses the presence or absence of major depressive episodes, major depressive disorder, suicidality, suicide behavior disorder, manic episode, hypomanic episode, bipolar I disorder, bipolar II disorder, other specified bipolar and related disorder, panic disorder, agoraphobia, social anxiety disorder, obsessive-compulsive disorder, posttraumatic stress disorder, alcohol use disorder, substance use disorder (non-alcohol), any psychotic disorder, major depressive disorder with psychotic features, bipolar I disorder with psychotic features, anorexia nervosa, bulimia nervosa, binge-eating disorder, generalized anxiety disorder, and antisocial personality disorder. Medical, organic, or drug causes will be ruled out. Each diagnostic item is answered by YES/NO. Earlier versions of MINI have been demonstrated to be administrated in a short

period of time (mean 18.7 ± minutes, median 15 minutes). The reliability and validity of the Japanese translation of the earlier version of MINI has been demonstrated (Otsubo et al., 2005). In terms of validity, criterion-related validity with Structured Clinical Interview for Diagnostic and Statistical Manual-III-R-patient version has been reported (Kappa > .49). In terms of reliability, sufficient inter-rater concordance has been reported (Kappa > .72).

2. アンヘドニア（Snaith–Hamilton Pleasure Scale; SHAPS, Snaith–Hamilton Pleasure Scale-clinician administered; SHAPS-C）：

SHAPS は、アンヘドニアの有無およびその重症度を測定するための自己記入式尺度である（Nagayama et al., 2012; Snaith et al., 1995）。14 項目から成り、1 から 4 の 4 件法で回答するが、評定肢は項目によって異なる（たとえば、項目 1「好きなテレビ番組やラジオ番組を楽しめますか？」に対する評定肢は「1. 少しも楽しくない」「2. 楽しくない」「3. 楽しい」「4. とても楽しい」）。得点が高いほど、アンヘドニアの重症度が高いことを示す。SHAPS 得点の合計が 20 点以上の場合、アンヘドニア症状を有するとみなされる（e.g. ClinicalTrials.gov; 臨床試験 NCT02874534, NCT02494050）。日本語版の信頼性に関しては、十分な値（α=.90）が報告されている（Nagayama et al., 2012）。回答時間 4 分程度。

SHAPS-C は、SHAPS に基づいて教示や評定肢を改良した、他者評価式尺度である（Ameli et al., 2014）。SHAPS と同様に、SHAPS-C は 14 項目から成り、１から４の 4 件法で回答するが、評定肢は全項目で共通である（１＝多くの喜びを感じる、２＝平均的／いつもと同じくらい喜びを感じる、３＝いくらか喜びを感じる、４＝喜びをまったく感じない）。得点が高いほど、アンヘドニアの重症度が高いことを示す。SHAPS-C 英語版の信頼性（Cronbach’s α＝.90）および妥当性（e.g. SHAPS との併存的妥当性; r=.85, p<.001）は十分であることが示されているが（Ameli et al., 2014）、SHAPS-C 日本語版は開発されていない。施行時間は 10 分程度。

2. Anhedonia（Snaith–Hamilton Pleasure Scale, SHAPS; Snaith–Hamilton Pleasure Scale-clinician administered, SHAPS-C）：

The SHAPS is a self-report questionnaire for assessing the presence/absence of anhedonia and its severity (Nagayama et al., 2012; Snaith et al., 1995). It consists of 14 items with 4-point Likert anchors. Each item is answered by different anchors. For example, Item 1 “I would enjoy my favourite television or radio programme” is answered as follows: strongly disagree, disagree, agree, or strongly agree. A higher score means more severe anhedonia symptoms. A total score higher than 20 is interpreted as the presence of anhedonia (e.g. ClinicalTrials.gov: NCT02874534, NCT02494050). Reliability of the Japanese version of SHAPS has been demonstrated (alpha = .90; Nagayama et al., 2012). The average time to answer is 4 minutes.

SHAPS-C is an interview method that improves the instruction and anchors based on the SHAPS (Ameli et al., 2014). Similar to SHAPS, SHAPS-C consists of 14 items with a 4-point Likert scale. The anchor is the same for all items (1 = lots of pleasure, 2 = average/usual pleasure, 3 = some pleasure, 4 = no pleasure). A higher score indicates more severe anhedonia. The original version of SHAPS-C has been reported to have sufficient reliability (alpha = .90) and validity (concurrent validity with SHAPS; r = .85, p<.001) (Ameli et al., 2014). We translated the English version of SHAPS-C into Japanese via a rigorous back-translation procedure. It takes approximately 10 minutes to complete the interview.

3. 抑うつ症状（GRID Hamilton Depression Rating Scale 17 Item; GRID-HAMD）：

ハミルトンうつ病評価尺度（Hamilton Depression Rating Scale; HAMD）は、抑うつ症状の重症度を測定するための評価尺度である（Hamilton, 1960）。本研究では、その改良版である GRID-HAMD17 項目版を用いる。GRID-HAMD は半構造化面接の形式をとり、評価者によって実施される。評価対象となる期間は、過去 1 週間である。評価項目は、抑うつ症状に関する 17 項目から成る。各項目の評点は、程度（5 段階評定）と頻度（４段階評定）をそれぞれ評価した上で、縦軸に程度、横軸に頻度をとったグリッド上に表現される基準に従って決定される（たとえば、項目 1「抑うつ気分」では、程度が「軽度」で頻度が「ほとんど常に」であれば 2 点という評点が与えられる）。合計得点は 17 項目すべての評点を合算して算出され、0-52 点の範囲を取る。高ければ高いほど、抑うつ症状が重症であることを示す。本尺度の信頼性に関しては、日本語版に関して優れた評定者間一致度（intraclass correlation coefficient; ICC = 0.95-0.99: Tabuse et al., 2007）が報告されている。また、内的一貫性に関しては原版において十分な値（α = 0.78）が示されている（Williams et al., 2008）。並存的妥当性に関しては、HAMD の別版である Structured Interview Guide for HAMD（SIGH-D）の各項目および合計得点と高い相関が報告されている（Williams et al., 2008）。施行時間 25 分程度。

3. Depression（GRID Hamilton Depression Rating Scale 17 Item, GRID-HAMD）：

Hamilton Depression Rating Scale (HAMD) is an interview for assessing the severity of depression (Hamilton, 1960). We will use the improved version of the original HAMD, which is called GRID-HAMD 17 item version. GRID-HAMD is conducted by the interviewer using a semi-structured format. The assessed period is during the week before the date of assessment. Each item is assessed in terms of severity (5-point Likert, vertical axis) and frequency (4-point Likert, horizontal axis). For example, item 1 “depressive mood” could be rated as 2 if the severity is “mild” and frequency is “almost always”. The total score ranges from 0 to 52. A higher score means more severe depression. In terms of reliability of the Japanese version, high inter-rater concordance has been reported (intraclass correlation coefficient; ICC = .95–.99, Tabuse et al., 2007). Sufficient internal consistency has been reported (alpha = .78)

(Williams et al., 2008). In terms of concurrent validity, high correlations between GRID-HAMD and Structured Interview Guide for HAMD（SIGH-D）have been reported (Williams et al., 2008). It takes approximately 25 minutes to complete this interview.

4. 抑うつ症状（Beck Depression Inventory-II; BDI-II）：

BDI-II は、過去 2 週間の抑うつ症状を測定するための自己記入式尺度である（Beck, Steer, & Brown, 1996; 小嶋・古川, 2003）。21 項目から成る。各項目は、0 から 3 の 4 件法で回答されるが、評定肢は項目によって異なる。得点範囲は 0–63 点であり、得点が高いほど抑うつ症状が重症であることを示す。日本語版の信頼性に関しては、一般青年・成人を対象とした研究で十分な値（α=.87）が報告されている（Kojima, Furukawa, Takahashi, Kawai, Nagaya, & Tokudome, 2002）。また、日本語版の並存的妥当性に関しては、CES-D と有意な中程度の正の相関（r = .69）が報告されている（Kojima et al, 2002）。回答時間 5 分程度。

4. Depression（Beck Depression Inventory-II; BDI-II）:

BDI-II is a self-report scale for assessing depression over the past two weeks (Beck et al., 1996; Kojima & Furukawa, 2003). It consists of 21 items with a 4-point Likert scale (0–3). The anchor varies from item to item. The score range is 0 to 63. A higher score indicates more severe depression. In terms of reliability of the Japanese version, sufficient internal consistency has been reported (alpha =.87) (Kojima et al., 2002). As for concurrent validity, the Japanese version of BDI-II was moderately correlated with CES-D (r = .69) (Kojima et al., 2002). It takes approximately 5 minutes to complete the scale.

5. ポジティブ・ネガティブ感情（The Positive and Negative Affect Schedule; PANAS）：

PANAS は、日々の感情体験を測定するための自己記入式尺度である（Clark & Watson, 1988; Clark & Watson, 1989）。22 項目から成る。各項目は、「1. ほとんど、または全くあてはまらない」から「5. 非常にあてはまる」の 5 件法で回答される。ネガティブな感情とポジティブな感情についての 2 つの下位尺度がある。各下位尺度は 11 項目で構成され、11-55 点という得点範囲をとる。得点が高いほど、ネガティブな感情またはポジティブな感情をより多く体験していることを示す。PANAS は様々なタイムフレームで用いられることがあるが、本研究では「過去 1 週間」というタイムフレームを採用する。回答時間 5 分程度。

5. Positive and Negative emotion (The Positive and Negative Affect Schedule, PANAS):

PANAS is a self-report measure for assessing daily emotional experiences (Clark & Watson, 1988; Clark & Watson, 1989). It consists of 22 items. Each item is rated on a 5-point Likert scale from 0 (very slightly or not at all) to 5 (extremely). There are two sub-scales: positive affect and negative affect. Each subscale consists of 11 items. The score range for the subscales

is 11 to 55. A higher score means more frequent experience of positive and negative affect. PANAS can be used at various time frames. We use the time frame of “past one week.” It takes approximately 5 minutes to answer this scale.

6. 人生満足度（Satisfaction With Life Scale; SWLS）：

SWLS は、人生に対する満足の程度を測定するための自己記入式尺度である（Diener et al., 1985; 大石、2009; 角野、1994）。5 項目から成る。各項目は、「1. 全くそうではない」から「7. 全くそうだ」の 7 件法で回答される。得点が高いほど、人生に対する満足度が高いことを示す。日本語版の信頼性に関しては、十分な値（α= .84〜.90）が報告されている（角野, 1994）。また、日本語版の妥当性に関しては、自尊心尺度との間に有意な中程度の正の相関（r = .59）および Y-G 性格検査の D 尺度（抑うつ性）と N 尺度（神経質）との間に有意な中程度の負の相関（D 尺度: r = -.50; N 尺度: r = -.34）が報告されている（角野, 1994）。回答時間 1 分程度。

6. Satisfaction with life（Satisfaction With Life Scale, SWLS）：

SWLS is a self-report measure for assessing the degree of life satisfaction (Diener et al., 1985; Oishi, 2009; Sumino, 1994). It consists of five items. Each item is answered using a 7-point Likert scale from “1. Strongly disagree” to “7. Strongly agree.” A higher score indicates higher satisfaction with life. The Japanese version is reported to have high internal consistency (alpha = .84–.90) (Sumino, 1994). In terms of the validity of the Japanese version, a moderate positive correlation with self-esteem (r = .59) and a moderate negative correlation with the Depression and Neuroticism scale in the Y-G personality inventory has been reported (r = -.50, -.34) (Sumino, 1994). It takes approximately 1 minute to answer this scale.

7. 心理的 well-being（Psychological Well-being Inventory; PWB）：

PWB は、心理的 well-being を測定するための自己記入式尺度である（Kitamura, Kishida, Katayama, Matsuoka, Miura, & Yamabe, 2004; Ryff, 1989）。人格的成長，人生における目的，自律性，環境制御力，自己受容，積極的な他者関係の 6 因子、計 18 項目で構成されている。各項目は「1. 全く当てはまらない」から「6. 大変当てはまる」の 6 件法で回答される。人格的成長は，発達と可能性の連続上にいて，新しい経験に向けて開かれている感覚を表す。人生における目的は，人生における目的と方向性の感覚を表す。自律性は，自己決定し，独立，内的に行動を調整できるという感覚を表す。自己受容は，自己に対する積極的な感覚を表す。環境制御力は，複雑な周囲の環境を統制できる有能さの感覚を表す。積極的な対人関係は，温かく，信頼できる他者関係を築いているという感覚を表す。回答時間 4 分程度。

7. Psychological well-being（Psychological Well-being Inventory, PWB）：

PWB is a self-report measure for assessing psychological well-being (Kitamura et al., 2004; Ryff, 1989). This 18-item scale has six subscales: purpose in life, autonomy, environmental mastery, personal growth, positive relations with others, and self-acceptance. Each item is rated using a 6-point Likert scale from “1. Completely disagree” to “6. Completely agree.” The definition of each sub-scale is described by Ryff (2014). Purpose in life is the feeling of meaning, purpose, and direction in one’s life. Autonomy is the feeling that one’s life is in accordance with one’s own personal convictions. Personal growth is the feeling that one is making use of one’s own personal talents and potential. Environmental mastery is the feeling of how well one manages life situations. Self-acceptance is the knowledge and acceptance one has of oneself, including awareness of personal limitations. Positive relations with others is a feeling of depth of connections one has in ties with significant others. It takes approximately 4 minutes to answer.

8. 環境内の報酬知覚（Environmental Reward Observation Scale; EROS）：

EROS は、行動に随伴する正の強化について主観的に評価する尺度である。10 項目からなり、4 件法（1-4 点）で回答する。得点範囲は 10-40 点であり、得点が高いほど、主観的に多くの報酬を知覚していることを表す。様々な population において信頼性および妥当性が報告されている（Armento & Hopko, 2007; 国里・高垣・岡島・中島・石川・金井・岡本・坂野・山脇、2011）。日本人サンプルを対象としたデータにおいては、Cronbach’s α=.78、再検査信頼性は r =.75 と高い信頼性が報告されている（国里他、2011）。また、妥当性に関しては、抑うつ・不安症状（BDI-II, CES-D, STAI）との中程度から強い負の相関（順に r = -.55, -.57, -.65）が報告されている（国里他、2011）。回答時間 2 分程度。

8. Environmental Reward Observation (Environmental Reward Observation Scale, EROS):

EROS is a self-report measure for assessing the subjective feeling of positive reinforcement contingent with behavior. In total, 10 items are answered using 4-point Likert anchors (1 to 4). The score ranges from 10 to 40. A higher score means a more subjective observation of reward. Its reliability and validity has been reported in multiple populations (Armento & Hopko, 2007; Kunisato et al., 2011). High reliability has been reported in the results of the Japanese population (alpha = .78, test-retest correlation = .75) (Kunisato et al., 2011). In terms of validity, EROS was moderately to strongly correlated with depression and anxiety (r = -.55, -.57, -.65 with BDI-II, CES-D, STAI) (Kunisato et al., 2011). It takes approximately 2 minutes to answer this scale.

9. 報酬の行動評価(Effort-Expenditure for Rewards Task; EEfRT)：

EEfRT は、報酬の獲得可能性に関する予測的価値およびエフォートに基づいた意思決定の程度を測定するための行動課題であり、コンピュータを用いて実施される（Treadway, Buckholtz, Schwartzman, Lambert, & Zald, 2009）。主な手順は以下の通りである；（１）報酬獲得のための課題として「易しい課題」と「難しい課題」を選択する画面が 5 秒間呈示される。（２）「易しい課題」では、7 秒以内に 30 回のボタン押し（利き手使用）が求められる。（３）「難しい課題」では、21 秒以内に 100 回のボタン押し（非利き手使用）が求められる。（４）それぞれの課題を達成すると決められた報酬を得ることができる。具体的には、「易しい課題」では一律 1 ドル、「難しい課題」では 1.24 ドルから 4.30 ドルの幅のある報酬が設定される。つまり、低コスト/低報酬条件、高コスト/低報酬条件、高コスト/高報酬条件の 3 条件が設定される。ただし､研究参加者は毎回の試行で報酬を必ず得られるわけではなく､3 種類の確率（12%, 50%, 88%）のいずれかで報酬を得ることができる。（５）研究参加者は、各試行終了後に報酬を得られるかどうかのフィードバックを受ける。（６）研究参加者は、これまでの一連の行程を 1 試行とし、20 分間の制限時間の間にできだけ多く行う。施行時間 20 分程度。

9. Behavioral assessment for reward (Effort-Expenditure for Rewards Task, EEfRT)：

EEfRT is a computerized behavioral task for assessing reward expectancy and effort-based decision making (Treadway et al., 2009). The procedure of this task is as follows: 1) 5-second display for selecting an easy or difficult task to obtain the reward, 2) easy task requires participants to push a key 30 times in 7 seconds using their dominant hand, 3) difficult task requires participants to push a key 100 time in 21 seconds using their non-dominant hand, 4) participants are eligible for a reward if they succeed in the task. The reward for the easy task is one dollar. The reward for the difficult task ranges from 1.24 to 4.30 dollars. As such, there are three conditions: low effort and low reward, high effort and low reward, and high effort and high reward. However, participants may not obtain a reward in every trial. The probability of receiving a reward is set at 12%, 50%, or 88%, 5) participants who succeed in the trial notice whether they get the reward immediately after each trial, 6) participants continue trials for 20 minutes. It takes 20 minutes to complete this task.

10. 宿題遵守（Homework Compliance Scale; HCS）：

HCS は、患者が宿題をどの程度遂行したかについて治療者が評価する尺度である（Primakoff, Epstein, & Covi, 1986）。1 項目からなり、毎セッションの終了時に 0-6 点で評価する。筆者らは、原著者の許可および協力を得て、バックトランスレーションのプロセスを経て日本語に翻訳した。

10. Homework compliance（Homework Compliance Scale, HCS）：

HCS is a therapist rating scale for assessing the degree of homework completion (Primakoff et

al., 1986) It consists of one item. Therapist rates 0 to 6 points after each session. We translated this scale into Japanese via rigorous back-translation procedures with permission from the author of original version.

11. マスキングの評価（Independent Evaluator Knowledge of Outcome; IEKNO）：

本尺度は、パニック障害に対する大規模な臨床研究(Barlow, Gorman, Shear, & Woods, 2000)や、統一プロトコルの RCT（Farchione et al., 2012）において利用された尺度である(Roll et al., 2004)。3 項目からなり、患者がどちらの群に割り付けられたと考えるか、それについてどの程度の自信を持っているか 0 から 8 の Likert で回答を求める。そして、最後の項目では、割付を見抜いたと考えられる場合には、どのような情報からそう考えられるかを尋ねる。本研究では、Blinding Index(Bang et al., 2004)を算出するために、割付の推測について“介入群”、“対照群”、“わからない”の 3 つから選択する回答方式とする。

11. Masking (Independent Evaluator Knowledge of Outcome, IEKNO):

This scale has been used in a large scale clinical trial for panic disorder (Barlow, Gorman, Shear, & Woods, 2000) and randomized controlled trial for unified protocol （Farchione et al., 2012). It consists of three items: judgement of allocated groups, confidence, and source of the judgement. In this study, we used the anchors “intervention group”, “comparison group”, and “I don’t know” to calculate the blinding index (Bang et al., 2004).

（11） 症例登録、割付の方法

(11) Method of registration and randomization

症例登録

Registration of participants

インフォームド・コンセントを経て評価面接により全ての選択・除外基準を確認されれば、NCNP 開発の EDC システム上で当該参加者の登録を研究コーディネーターが行う。順番の作成は研究開始前に、割付担当者がコンピュータにより行われる（中央登録による割付の隠蔵）。試験開始前に、割付担当者は作成された順番をもとに、各登録 ID と一致した音源トラックに介入条件（超高周波音源 vs. プラセボ音源）を設定する。

After obtaining informed consent and confirming eligibility by an assessment interview, research coordinator will register the participant using EDC system developed by NCNP. Sequences will be generated by this EDC system in advance of starting this trial (allocation concealment by central registration). Personnel for allocation stuff will set the audio track (inaudible high-frequency sound vs. placebo sound) in accordance with the generated sequences.

割付方法と割付調整因子

Method of randomization and stratification

ブロックランダム化を行い、調整因子は設定しない。

We will use block randomization without any stratification.

割付表の保管と開錠手続き

Storage of randomization table and procedure for unblinding

割付表は割付担当者が保管する。重篤な有害事象が発生するなど、研究責任者が必要と認めた場合には、研究責任者が担当者に開錠を依頼する。

Personnel for allocation will store the randomization table. Whenever the primary investigator acknowledges the need for unblinding such as in the occurrence of a severe adverse event, the primary investigator will request the personnel for anonymization to open the key.

（12）　統計解析方法

(12) Statistical analysis

解析の概要

Summary of statistical analysis plan

データマネジャーがデータの固定と解析を行う。全ての主要・副次評価項目の解析において、介入群と対照群を比較する。全ての解析で、P 値が小数点 3 までの値で表現され、0.001 以下のものは p < .001 と記載する。解析ソフトは、SPSS、SAS、R を用いる予定である。統計的検定を用いる解析では、両側検定として有意水準を 5%とする。本研究は主要評価項目の解析のためにデザインされているため、副次評価およびその他の解析（サブグループ解析・調整解析）は探索的なものとなる。

The data manager will fix and analyze the data. We will compare the intervention and comparison groups in primary and secondary analyses. The p-value will be expressed to three decimal places. If the p-value is under .001, it will be described as p < .001. We will use statistical software SPSS, SAS, and/or R. When we apply statistical tests, we will set statistical significance for 5% of a two-tailed test. As this study is designed to test the primary outcome, analyses for secondary and other outcomes, as well as for other purposes (sub-group analysis, adjusted analysis, etc.) will be exploratory.

主要評価項目の解析

Analysis of primary outcome

評価項目：1 週から 12 週にかけての９度測定される SHAPS 日本語版得点

主要評価項目について反復測定混合モデル（MMRM）により検討する。従属変数は 1–11 週までの 8 ビジット時点、および 12 週時点の評価項目とする。独立変数の固定効果を割付と測定時点、割り付けと測定時点の交互作用項 (割付け*測定時点)、および評価項目の pre 値（-1 週）とする。12 週時点（ビジット９；主要ビジット）での調整された平均値についての割付群による差を t 検定にて検討する。他のビジット時点での差については二次解析として検討する。

Outcome: Score of the Japanese version of SHAPS assessed nine times from 1 to 12 weeks. Mixed model for repeated measures (MMRM) will be conducted to analyze the primary outcome. The dependent variable is participants' SHAPS scores assessed at eight visit points from week 1 to 11 (intervention period) and week 12 (post-assessment). Fixed-effects are allocation, visit, and allocation-by-visit interaction, and SHAPS at pre-intervention (week -1). Then, t-test for difference of adjusted means between allocations at week 12 (visit 9: primary visit) will be conducted. Inference on the differences at the other visits will be also conducted as secondary analyses.

副次評価項目の解析

Analysis of secondary outcome

評価項目：12 週時点の SHAPS-C

ベースラインを共変量とした共分散分析を行う。

Outcome: Score of SHAPS-C at week 12

Analysis of covariance including baseline as a covariate will be conducted.

他の評価項目の解析

Analysis of other outcomes

評価項目：12 週時点の BDI-II, PANAS, SWLS, PWB, EROS, EEfRT

主要評価項目と同様に、MMRM により検討する。各測定時点での割付による調整された平均値の割付群による差を検討する。

Outcome: BDI-II, PANAS, SWLS, PWB, EROS, EEfRT at weeks 5 and 12MMRM analysis will be conducted as same as primary analysis. Thus, inference on differences of adjusted means between allocations at each visit will be conducted.

欠測値の処理

Handling of missing data and sensitivity analysis

混合モデルによって解析することで欠損値に対処する。さらに、“best”または”worst”のそれぞれのケースシナリオを仮定した感度分析を実施する。

We use mixed model to treat missing data. Furthermore, we will conduct sensitivity analyses by assuming both “best” and “worst” case scenarios.

上記に定めていない症例やデータの取り扱いは、研究責任者と解析責任者で協議、決定する。

Other statistical analyses or data handling will be discussed and judged by the primary investigator and study statistician.

解析対象集団

Analysis populations

主要・副次評価項目についての解析はすべて、Intent-To-Treat 原則に基づき登録されて全参加者を対象として実施する。副次的な解析対象集団として、研究登録された後に中止症例に該当しなかった全例を当研究計画書に適合した対象集団（Per Protocol Set, PPS=Completer case）として、評価項目についての解析を行う。

Analyses for the primary and secondary outcome analysis will be conducted on Intent-To-Treat principle, which all registered participant are subject to analysis. As the secondary analysis set, we will conduct the outcome analysis by using the all registered participant who will not meet the discontinuing criteria as Per Protocol Set.

（13）　試料・情報の授受

(13) Provision or receipt of specimens or information

本研究は単施設で実施するため、外部との情報の授受はない。

We will not exchange any specimens or information as this is a single site study.

6.　研究対象者の選定方針

6. Enrolment criteria of research participants

（1）選択基準

（1）Inclusion criteria

1）　アンヘドニア症状を有すること（SHAPS 日本語版得点の合計が 20 点以上）

2）　うつ症状が軽症（GRID-HAMD の 8 点）以上

3）　18 歳以上

(1) Anhedonia symptoms (Snaith-Hamilton Pleasure Scale score >= 20)

(2) Depressive symptoms are mild or severe (GRID Hamilton depression rating scale >= 8)

(3) Aged 18 years or older

【各選択基準の設定理由】

1) 有効性評価のため
2) 有効性評価のため
3) 有効性評価のため（研究対象者を SHAPS 日本語版の妥当性が検証されている成人に限定するため）

【Reason for each inclusion criteria】

1) For efficacy evaluation
2) For efficacy evaluation
3) For efficacy evaluation (Japanese version of SHAPS has not been examined for reliability and validity among the younger Japanese population)

（2）除外基準

(2) Exclusion criteria

1） 精神病性障害（現在）（MINI にて評価）
2） 躁病エピソード（現在）（MINI にて評価）
3） 物質使用障害（現在、重度）（MINI にて評価）
4） 重篤な自殺念慮（MINI にて評価）
5） 治療に支障のある身体疾患や認知機能障害
6） その他に、認知行動療法を遂行する上で障害となる問題のある者

1） No current psychotic disorders at baseline assessed by the Mini-International Neuropsychiatric Interview (MINI)
2） No current manic episode at baseline assessed by MINI
3） No severe substance use disorders at baseline assessed by MINI
4） No serious suicidal ideation at baseline assessed by MINI
5） No severe or unstable physical disorders or major cognitive deficits at baseline
6） Other problems that may be serious obstacles for conducting CBT

【各除外基準の設定理由】

1) 有効性評価のため
2) 安全性のため
3) 安全性のため
4) 安全性のため
5) 有効性評価のため
6) 安全性及び有効性評価のため

【Reason for each exclusion criteria】

1) For efficacy evaluation
2) For safety

3) For safety
4) For safety
5) For efficacy evaluation
6) For efficacy evaluation and safety

7. インフォームド・コンセント等を受ける手続等

7. Procedure for obtaining informed consent, etc.

倫理委員会で承認の得られた同意説明文書を研究対象者（代諾者が必要な場合は代諾者を含む、以下同じ）に渡し、文書及び口頭による十分な説明を行い、研究対象者の自由意思による同意を文書で取得する。研究対象者の同意に影響を及ぼす情報が得られたときや、研究対象者の同意に影響を及ぼすような研究計画書等の変更が行われるときは、速やかに研究対象者に情報提供し、研究に参加するか否かについて研究対象者の意思を予め確認するとともに、事前に倫理委員会の承認を得て同意説明文書等の改訂を行い、研究対象者の再同意を得ることとする。本研究では、有効なインフォームドコンセントを与えることができると判断された 18 歳以上の未成年者を対象に加える。それらの者を加える理由は、世界的には、成人として 18 歳以上を対象として研究されることが一般的であるためである。

Research coordinators will present the IRB approved informed consent briefing paper to the participants (including to legally acceptable representatives, if applicable), orally explain the research sufficiently using the briefing paper, and obtain consent that is reflected by the participant’s free will in written document. If any changes occur in the research protocol or the primary investigator acknowledges information that may influence participants’ consent, the primary investigator will immediately provide such information to participants and confirm continued participation in this study. The primary investigator will also revise the informed consent briefing paper and re-obtain participants consent using the revised paper. Subjects in this study are those older than 18 years old with sufficient capacity to provide informed consent. This is because in this type of study around the world, 18 years of age or older is sufficient to be regarded as “adult”; in Japan, 20 years of age or older is considered “adult” `.

1. 同意説明文書の概略、研究により生じる知的財産権の帰属
2. 研究の名称、研究実施について所属機関の長の許可を受けている旨
3. 研究の目的及び意義
4. 研究対象者として選定された理由（研究対象となる方）、研究の方法及び期間、参加協力事項
5. 研究対象者に生じる負担並びに予測されるリスク及び利益

6. 研究が実施又は継続されることに同意した場合であっても随時これを撤回できる旨、研究が実施又は継続されることに同意しないこと又は同意を撤回することによって研究対象者等が不利益な取扱いを受けない旨
7. 研究に関する情報公開の方法
8. 研究対象者等の求めに応じて、他の研究対象者等の個人情報等の保護及び当該研究の独創性の確保に支障がない範囲内で研究計画書及び研究の方法に関する資料を入手又は閲覧できる旨並びにその入手又は閲覧の方法
9. 個人情報等の取り扱い（匿名化の方法を含む）、情報の保管
10. 情報の廃棄の方法、研究終了後のデータ取扱の方針
11. 研究の資金源、利益相反
12. 研究対象者等及びその関係者からの相談等への対応
13. 経済的負担及び謝礼の内容
14. 研究で用いられる治療方法以外の治療方法の内容
15. 研究終了後の医療の提供の有無とその内容
16. 研究対象者に関わる偶発的所見への対応
17. 健康被害に対する補償の有無及びその内容
18. 研究終了後のデータの二次利用の可能性とその対応方法
19. 研究機関の名称及び研究責任者の氏名
20. 問い合わせ先

1. Outline of the informed consent briefing paper, ownership of intellectual property
2. Title of the research and information on approval given by the chief executive of the research implementing entity concerning its implementation
3. Objectives and significance of the research
4. Reasons for enrolment in the research, method, and time period of the research
5. Potential burdens on research subjects and predictable risks and benefits
6. Condition that research subjects may withdraw their consent at any time even after they have given consent that the research commences or continues. Condition that the refusal or withdrawal of consent by a research subject regarding commencement or continual of the research does not cause any disadvantage to them
7. Means to make information on the research public
8. The fact that research subjects can request and obtain or read the research protocol and documents concerning method of the research, to the extent that it does not interfere with the protection of personal information, etc. of other research subjects or the originality of the research, as well as the procedure to obtain or read such protocols and documents
9. Handling of personal information, etc. (including process of anonymization and when anonymization is conducted), means for storage information

10. Means for disposal of information
11. Status of research-related conflicts of interest of the research implementing entity, such as research fund resources, as well as research-related conflicts of interest of each investigator such as his/her individual income
12. Response to consultation, etc. made by research subjects and other individuals concerned
13. When the research involves any financial expenditure on or remuneration for the research subject; a statement to that effect and details of such
14. When the research involves any medical technique beyond usual medical practice, description of alternative procedure(s) or course(s) of treatment
15. When the research involves any medical technique beyond usual medical practice, response related to the healthcare delivery to the research subjects after the research
16. When any significant finding concerning the subject’s health or generic characteristics which may be inherited by his/her offspring, etc. may be obtained through implementing the research, handling of the research results related to the research subject (including incidental findings)
17. When the research involves any invasiveness, whether compensation will be offered for research-related injury and details of such compensation
18. With respect to specimens and information acquired from the research subject, when any of those may be utilized or provided to other research implementing entities for research in the future that is not identified at the time of obtaining consent from the research subject; a statement to that effect and the contents of utilization assumed at the time of obtaining consent;
19. Names of the research implementing entity and the principal investigator
20. Contact information

8. 試料・情報、個人情報等の取扱い（匿名化する場合の方法、個人情報の安全管理方法など）

8. Handling of specimens, information, and personal information (method of anonymization, handling of personal information, etc.);

個人情報管理者（田島美幸）は、同意書など個人情報を含む研究必須文書等の紙媒体や電子媒体、個人情報と研究用 ID の対応表について、漏洩、盗難、紛失しないように以下の通り管理・保存する。

Administrator of the management of personal information (i.e., Miyuki Tajima, Ph.D.) will manage and store all research-related printed or electronic records with personal information (e.g., informed consent documents), correspondence table of personal information and research ID number to prevent any information being divulged, stolen, or lost. Details of the management and storage are as follows.

物理的安全管理

Physical security management

物理的安全管理措置は、あらゆる紙資料や電子データ（同意書、対照表、録音・録画のデータを含む）の取り扱いを想定している。

Physical security management concerns any printed or electronic data including signed informed consent documents, correspondence table, recorded audio or video, etc.

1. 個人データを取り扱う区域の管理：個人データは国立精神・神経医療研究センター内において、患者から直接取得される（病院内での症状評価面接や自己記入式尺度への回答、PC を用いて行われる認知課題のデータ、認知行動療法セッションの録音・録画）。この個人データは、国立精神・神経医療研究センター７号館３階の認知行動療法センター内の居室のキャビネットの中に保管する。個人データを保管し扱う区域は国立精神・神経医療研究センター７号館３階の認知行動療法センター内とする。認知行動療法センターへの入室はカードキーで管理されており、カードキーはセンターに雇用されている者のみが保有する。カードの所持状況は、認知行動療法センター長が帳簿を作り管理する。居室の鍵は、NCNP 全体の鍵管理の方針に従い、使用外の時間は NCNP 防災センターにて保管される。

1. Restriction area for handling the personal information

   All personal information will be obtained from participants in the National Center of Neurology and Psychiatry (e.g., answering the psychiatric symptoms interview or self-report scales in the hospital, cognitive task using PC, and recorded audio or video date of the CBT session). These data will be stored in the cabinet in the National Center for Cognitive-Behavior Therapy and Research located on the 3rd floor of 7th building in the National Center of Neurology and Psychiatry. Entry to the National Center for Cognitive-Behavior Therapy and Research is access restricted. Only center staff are permitted entry. The director of the center is responsible for the management of the card key and maintaining the information of the possession of card keys. Keys for each office room will be returned to the disaster prevention center at the end of every day.

2. 機器及び電子媒体等の盗難等の防止：盗難防止のために、上記①の管理に加えて、キャビネットもそれぞれ施錠可能なものを用いる。さらに、キャビネットの鍵は、パスワード認証が必要なキーボックスに保管する。キーボックスは、特殊な方法を用いなければ脱着できないような強度で壁に固定する。

2. Prevention of theft of devices and electronic media

   To prevent theft, we will use a lockable cabinet. Furthermore, we will use the key box with password authentication. This key box will be placed on the wall firmly so as not to easily removed.

3. 電子媒体等を持ち運ぶ場合の漏えい等の防止：電子媒体は管理区域内のみで利用する。

3. Prevention of divulgation during electronic media usage

Electronic media will be used in the restricted area

4. 個人データの削除及び機器、電子媒体等の廃棄：匿名化された検査データなどの紙資料は、すべてただちに個人を特定するような情報（固有名詞など）を削除したかたちで保管する。これらの検査データなどと、ただちに個人が特定できる情報を含む研究データ（同意書、対応表、録音・録画データ）は研究終了後 5 年後に個人情報をわからなくして、復元不可能な形で廃棄する。

4. Disposal of personal information and electronic devices and media

Printed data such as self-report scales will be immediately anonymized by deleting any identifying information (e.g., specific names etc.) and securely stored. Five years after the completion of the study, these anonymized data and other personal identifying data (signed informed consent documents, correspondence table, and recorded audio and video files) will be disposed of to avoid restoration.

技術的安全管理：

Technical Security Management:

技術的安全管理は電子化されたデータ（録音・録画のデータを含む）の安全管理措置を想定しており、下記のような措置をとる。

Technical security management concerns the handling of electronic data including audio and video data.

1. アクセス制御：研究代表者の伊藤正哉、コーディネート担当の横山知加、宮前光宏のみが録音・録画データが保管された HDD にアクセスできる。研究遂行上の必要に応じて、研究代表者が認める者に一時的にアクセスを許可することがある（データ入力やモニタリングなど）が、その場合にはアクセス制御を許可された者の監督下での扱いとする。電子データは強制暗号化とパスワード認証が求められる HDD を用いてデータを保管する。

1. Access restriction: Only the primary investigator (MI) and coordinators (CY, MM) are able to access the HDD where the audio and video data are stored. If necessary, the primary investigator may temporarily allow another person to access the data (e.g., for data entry and monitoring). In that case, the person has to treat the data under the monitoring of MI, CY, or MM. All electronic data will be stored in the encrypted HDD with password protection.

2. アクセス者の識別と認証：上記①に定めた者のみが HDD に保管されたデータにアクセスでき、認証パスワードを知ることができるようにする。

2. Identification and authentication of accessible personnel:

Only MI, CY, and MM can access the HDD and have the password.

3. 外部からの不正アクセス等の防止：録音・録画データの再生においては、インターネットに接続されない端末もしくは機器を用いる。

3. Prevention of unauthorized access: When handling the data such as playing the recorded audio or video files, we will use the devices offline while not connected to the Internet.

4. 情報システムの使用に伴う漏えい等の防止：電子データは外部ネットワークにつながった状態の機器には接続しない。

4. Prevention of divulgation in the process of using electronic information system: We will not connect any electronic data to outside networks.

人的安全管理：

Human security management:

1. 雇用契約及び委託契約の締結時における守秘義務規定：当研究は『平成 22 年規程第４０号保有する個人情報の保護に関する規程』が適用される NCNP 職員もしくは研究生によって実施される。

1. Confidentiality obligation in the employment and consignment contracts: All study staff in this study are subject to the policy for protection of personal information (NCNP policy No. 44, 2010).

2. 研究者等に対する教育・訓練の実施：手順書の徹底を図るよう訓練を実施するとともに、NCNP で実施される『平成 22 年規程第４０号保有する個人情報の保護に関する規程』第８条にて規定されている研修をはじめ、コンプライアンス研修、倫理研修に参加する。

2. Education and training for the researchers:

All study staff are subject to training following the standard procedure operations for this study. They will participate in the workshop described in article 8 in the NCNP policy for protection of personal information (NCNP policy No. 44, 2010) and other workshops for compliance and research ethics.

9. 研究参加のリスクと研究がもたらすベネフィット

9. Risks and benefits of research participation

（1）研究対象者に生じる負担

(1) Burdens on research subjects

本研究によって、研究の実施に伴って確定的に生じる事象としては、全 12 週間にわたる毎週の通院と評価と介入治療にかかる時間と労力の消費であるが、これらがすべからく研究対象者にとって好ましくない事象として知覚・認識されるとは限らない。

Burdens for the participants will be time and effort for the 12-week intervention and symptom evaluation. Perceptions of these burdens will vary between participants.

(2) 研究対象者に生じ得る不利益（有害事象不具合等）

(2) Potential disadvantages for research subjects (e.g., adverse events)

本研究によって生じ得る不利益は想定されないが、自身の人生や生活を振り返って、今までしてきたのとは違う取り組みを行う中で、一時的にネガティブな気分を体験することはあり得る。

We do not expect any disadvantages to participants for participating in this research. However, participants may temporarily experience a negative mood in response to reflecting on their past life and practicing new behaviors.

(3) リスクを最小化する方法

(3) Strategy to minimize risks

健康被害が生じた場合、適切な医療等の対応が行われるように図る。本研究では、研究に伴う健康被害のリスクは、一般の外来診療に伴うもの以上は特にないものと考えられるため、健康被害に対する医療も通常の診療と同様に、被験者の保険診療内で行う。

If any health damage occurs, the primary investigator will arrange an appropriate medical response. As we assume that the risk of health damage is not likely to occur as is usual in outpatient care, we will use participants' medical insurance for the medical expenses of any health damage, as used in usual-care.

(4) 予想されるベネフィットと (1) (2) (3) を踏まえた総合評価

(4) Comprehensive assessment of burdens, risks, and benefits

本研究の参加者は、本研究で有効性を検証しようとしている症状を有する方であり、本研究に参加し介入を受けることで、直接的に本人の症状が改善する可能性がある。明確に想定される不利益は、評価と介入に要する労力と時間である。万が一に不利益が生じた場合には、適切な医療等の対応が行われるように図る。これらを総合評価し、本人に利益が生じる可能性があるだけでなく、研究成果により将来の医療の進歩に貢献できる可能性がある。その具体的意義については 1 に記載した通りである。

We developed the intervention and are going to test its efficacy specifically for anhedonia symptoms. We hypothesize that there are potential benefits for participants for symptom improvement by participating in this research. Clear disadvantages for the participants will be the effort and time required for symptom evaluation and treatment. If by any chance other

disadvantages should occur, the primary investigator will arrange an appropriate medical response. By comprehensively assessing these aspects, this study has potential benefits for the participants and the study results may promote the progress of future medicine. The medical significance of the study has already been discussed in section 1 of this protocol.

(5）個々の研究対象者における中止基準

(5）Criteria for discontinuing interventions for a given trial participant

研究中止時の対応

Correspondence when the discontinuing occurs

個々の症例が以下のいずれかの中止基準に該当する場合、その症例の試験治療を中止する。中止の日付・時期(治療期間・追跡調査期間)、中止の理由、経過をカルテならびに CRF に明記するとともに、中止時点で主要評価項目と副次評価項目に関する評価を行い有効性・安全性の評価を行う。有害事象発生により中止した場合は、研究責任者は担当治療者、スーパーバイザー、主治医、NCNP 担当医と状況を検討し、介入中止になった理由に応じて適切な対応を決定し、可能なかぎり原状に回復するまでフォローする。中止後も可能なかぎり 12 週時点での評価を行う。

If a participant meets either of the following discontinuing criteria, the participant will terminate participation. The trial coordinator will record the date and period (intervention or follow-up period) of discontinuation, its reason, and the course in CRF. Whenever possible, participants will be requested to participate in primary and secondary outcome evaluations at the time of discontinuation. If the discontinuation occurred due to an adverse event, the primary investigator will discuss with the therapist, supervisor, primary doctor, and responsive doctor at NCNP about the possible response to the patients, and monitor patients until they recover to the degree of baseline. Whenever possible, the trial coordinator will ask patients to participate in the outcome evaluation at week 12.

中止基準

Criteria for discontinuation of participation in the trial

1） 被験者から試験参加の辞退の申し出や同意の撤回があった場合
2） 被験者との連絡が不通となった場合
3） 重篤な有害事象により試験治療の継続が困難な場合
4） 試験全体が中止された場合
5） その他の理由により、研究責任者が試験を中止することが適当と判断した場合

1） Participant requests discontinuation or withdraws consent
2） Unable to contact the participant
3） Difficulty continuing participation due to severe adverse events
4） Discontinuation of the whole trial
5） Primary investigator’s judgement of discontinuation to be appropriate for any other reason

1） 研究全体の中止基準

1） Discontinuation of trial

1） 倫理委員会により中止の勧告あるいは指示があった場合は、試験を中止する。研究責任者および効果安全性評価委員で構成する委員会は、以下の事項に該当する場合は試験実施継続の可否を検討する。
2） 治療の品質、安全性、有効性に関する重大な情報が得られたとき。
3） 倫理委員会により実施計画等の変更の指示があり、これを受入れることが困難と判断されたとき。試験の中止または中断を決定した時は、速やかに倫理委員会にその理由とともに文書で報告する。試験中止の決定を行った後、関係医師および心理士等に速やかに伝達し、中止後の処理にあたるものとする。

1） If the IRB advises or orders trial discontinuation, this trial will be stopped. The committee consisting of the primary investigator and members of DSMB will examine the appropriateness of continuing the trial when the following occurs.
2） Acknowledgement of important information regarding quality, safety, and efficacy of the intervention.
3） When the IRB orders modification of trial procedures and the primary investigator judges such modification to be difficult. If the primary investigator decides to stop or discontinue the trial, the primary investigator will report the reason immediately to IRB in written form. After deciding to discontinue the trial, the primary investigator will immediately notify the related doctors, psychologists, and other medical staff, and deal with the discontinuation.

10. 研究に用いられる情報に係る資料の保管及び廃棄の方法

10. Method for storage and disposal of information including records related to information utilized in research

個人情報管理者は、同意書など個人情報を含む研究必須文書等の紙媒体や電子媒体、個人情報と研究用 ID の対照表を、認知行動療法センターに設置した鍵のかかる保管庫にて漏洩、盗難、紛失しないように管理・保存する。紙媒体や電子媒体は、研究終了後から 5 年間経過

後に、個人情報を分からなくしたことを確認した上で破棄される。なお、個人情報と研究用 ID の対照表の破棄後、連結不可能匿名化状態となった電子データベースは、追加解析や他の研究のため利用されることがある。この連結不可能匿名化データの電子ファイルは認知行動療法センター内の LAN やインターネットに接続されていないパソコンにてパスワードをかけて暗号化した上でハードディスクドライブに保存し、研究責任者の管理のもと保存される。なお、録音・録画された記録の一部については、コンサルテーションや事例検討のために、セキュリティの確保されたクラウド上で共有されることがある。共有された録音・録画データは、毎回のコンサルテーションや事例検討後に、確実にデータ消去を行う。

本試験のデータが他機関にて分析されたり、他の研究のために利用されたりする具体的な予定はないものの、その可能性は否定できない。本試験の匿名化されたデータの将来的な活用については、被験者に説明し、同意を得る。

A manager of private information will store the necessary documents or electronic records that contain personal information (such as documents for informed consent) and correspondence table for personal information and research ID in a locked cabinet to prevent any divulgation, theft, or loss. All documents and electronic records will be disposed of after the termination of research with erasure of personal information. After the disposal of correspondence table, the unlinked anonymized data may be used for additional analysis or other research purposes. The primary investigator will store the unlinked anonymized data in the encrypted HDD under password lock separated from any LAN or Internet. Parts of the recorded audio or video may be shared on a secure cloud system for case consultation. Such shared audio or video dates will be erased with absolute certainty after every consultation session.

Although we have no plans to transfer the data to other institutions or to use the data for other research purposes at this time, we cannot exclude these possibilities. We will obtain informed consent from the participants regarding the future utilization of the anonymized data.

11. 研究機関の長への報告内容及び方法

11. Matters to be reported to the chief executive of the research implementing entity and relevant procedures

研究機関の長（理事長）への報告については下記の通りとする。

Primary investigator will report to the chief executive as follows.

1） 年 1 回、研究実施状況について報告し、研究継続の適否について倫理委員会の審査を受ける。

2） 重篤な有害事象が発生した場合は、速やかに理事長に報告し、研究継続の適否について倫理委員会の審査を受ける。

3） 介入法の有効性・安全性に関する重要な情報が得られた場合は、研究責任者の見解を記載し、理事長に報告し、研究継続の適否について倫理委員会の審査を受ける。

4） 研究の終了時（中止または中断の場合を含む）には、理事長に報告する。

1） Primary investigator will report the progress of research once a year. The IRB will review it and examine the appropriateness of continuing the research.

2） If a severe adverse event occurs, the primary investigator will immediately report it to the chief executive. The IRB will review it and examine the appropriateness of continuing the research.

3） If the primary investigator acknowledges any important information regarding the efficacy and safety of the intervention, the primary investigator will report the event with assessment by the investigator. The IRB will review it and examine the appropriateness of continuing the research.

4） Primary investigator will report the termination or discontinuation of research.

12. 研究に係る資金と利益相反に関する状況

12. Status of research-related conflicts of interest of the research implementing entity, such as research fund resources, and research-related conflicts of interest of each investigator, such as his/her individual income

本研究は、"日本医療研究開発機構研究費　平成 29 年度「統合医療」に係る医療の質向上・科学的根拠収集研究事業　1-4 漢方及び鍼灸を除く各種療法に関する科学的知見を創出するための研究"に対して、課題名『超高周波音響療法による認知行動療法の増強効果』を資金源とし、研究代表者は堀越勝、研究期間は本倫理申請承認から平成 32 年 3 月 31 日である。また、行動指標をアウトカムとした附属研究については、パブリックヘルス財団　2017 年度ストレス科学分野「ストレスマネジメント」に対して、課題名『認知行動療法面接中における超高周波音響呈示のブースト効果研究：抑うつ症状の改善に焦点を当てて』を資金源とし、研究代表者は宮前光宏、研究期間は 2017 年４月１日から 2018 年 3 月 31 日で助成を受けている。また、本研究に係る全ての研究者及びその配偶者などの家族は、本研究で用いる超高周波音響装置を製造している業者との間に経済的利害関係、雇用関係は一切無い。従って、研究者が企業等とは独立して計画し実施するものであり、研究結果及び解析等に影響を及ぼすことは無い。本研究に携わる研究者等は、いずれも利益相反状態にないことを確認している。

Funding resources for this research are provided by the Japan Agency for Medical Research and Development as the Grant for Research Project for Improving Quality in Healthcare and Collecting Scientific Evidence on Integrative Medicine (2017). The approved title for the project was "Augmentation of cognitive behavior therapy by inaudible high-frequency sound therapy" awarded to Masaru Horikoshi. The planned study period is from the approval date of this research protocol by

IRB to 31st of March 2020. The ancillary study for examining the augmentation effect of inaudible high-frequency sound of positive valence system-focused CBT on the performance of behavioral tasks is funded by the Public Health Research Foundation as the research grant for stress management in the field of stress science (“Research on the boosting effect of inaudible high-frequency sound exposure during the session of cognitive behavioral therapy: Focusing on the improvement of depression”) awarded to Mitsuhiro Miyamae. The research period was from 1st of April 2017 to 31st of March 2018. None of the researchers involved in this project, or their family members, had any conflict of interest with the company that built the audio systems. Hence, this research will be conducted independently of any company or other entity, resulting in no influence on the results or analysis. All of the relevant researchers in this study confirmed that they had no conflict of interest.

13. 研究に関する情報公開の方法

13. Means to disclose research information

臨床試験登録

Registration in clinical trial registry

本臨床試験は、大学病院医療情報ネットワーク(UMIN)「臨床試験登録システム」に登録している（UMIN000031948）。

This clinical trial has been registered in a public database operated by the National University Hospital Council of Japan (UMIN000031948).

成果の帰属と公表

Attribution and publication of research outcomes

本研究によっていかなる結果が得られても、その結果は被験者を特定できないようにした上で、学術専門誌などにて公表される。Primary Outcome Paper とプロトコル論文は伊藤が草稿を執筆し、共同主任研究者（堀越）がその内容を確認した上で、共著者の確認・修正を全著者が納得するまで繰り返した上で投稿する。二次解析などの他の論文は、Primary Outcome Paper が公表された後に公表することとする。行動指標をメインアウトカムとして、治療メカニズムを検証する論文に関しては、宮前が草稿を執筆し、共著者の確認・修正を全著者が納得するまで繰り返した上で投稿する。

Regardless of the results, the primary investigator will publish the results in anonymized format in the academic journal. MI will write the first draft for the primary outcome paper and protocol paper. After confirmation by the co-primary investigator (MH), the draft will be repeatedly modified until all authors have approved the final version. Other papers for secondary analysis will be published after acceptance of the primary outcome paper. For the behavioral task outcomes, MM will write a first draft and continually modify the paper until all co-authors have given approval.

14. 研究対象者等及びその関係者からの相談等への対応

14. Means to respond to inquiries made by the research subjects and/or other individuals concerned

研究対象者等及びその関係者からの相談については、下記相談窓口を説明文書に明示し、申し出があった場合には遅滞なく対応する。

The primary investigator will reveal the address and phone number of the inquiry counter on the document used for informed consent for any possible consultation by subjects or related individuals. If any consultation is raised, the primary investigator will respond immediately.

研究相談窓口

Inquiry counter

〒187-8551

東京都小平市小川東町四丁目 1 番 1 号

国立研究開発法人国立精神・神経医療研究センター 認知行動療法センター

電話番号 042-341-271２(内線 3605 または 3606)

対応時間：月・水・金（9 : 30−17 : 00）

氏名　伊藤正哉　所属・職名 認知行動療法センター 室長

Masaya Ito,

Ogawa higashi 4-1-1, Kodaira, Tokyo,

National Center of Neurology and Psychiatry, 187-8551

Phone: 042-341-2712 (Ex 3605, 3606)

Time: 9:30 to 17:00; Monday, Wednesday, and Friday

倫理委員会事務局への連絡先

Contact to Institution Review Board

〒187-8551

東京都小平市小川東町四丁目 1 番 1 号

国立研究開発国立精神・神経医療研究センター倫理委員会事務局

e-mail:rinri-jimu@ncnp.go.jp

Ogawa higashi 4-1-1, Kodaira, Tokyo,

National Center of Neurology and Psychiatry, 187-8551

Executive office of IRB

e-mail: rinri-jimu@ncnp.go.jp

15. 研究対象者等に経済的負担又は謝礼がある場合には、その旨及びその内容

15. Financial expenditure or remuneration for research subjects and associated details

本研究への参加により追加でご負担いただく費用、また、研究参加に対する謝礼はない。

There is no financial expenditure or remuneration for participation in this research.

16. 侵襲（軽微な侵襲を除く。）を伴う研究において重篤な有害事象が発生した際の対応

16. Means to respond in the case of serious adverse events (e.g., if the research involves invasiveness, not including minor invasiveness)

有害事象及び副作用の定義

Definition of adverse event or side effect

有害事象には、研究期間中に発生する有害と認められるあらゆる事象が含まれる。

An adverse event could be any adverse phenomenon during participation in this research.

有害事象の評価及び判定規準

Evaluation and criteria for adverse event

所定の項目（口渇、便秘、排尿障害、視力調節障害、起立性低血圧、眠気、倦怠感、不眠、不安・焦燥、落ち込み・意欲低下、食欲不振、体重増加、体重減少、性欲低下、動悸、ふるえ、発汗、頭痛、ふらつき、その他）について、その有無を確認する。担当者は所定の用紙を用いて、「前回以来、身体的または精神的な症状で急に悪化したり発生しましたか」と口頭にて尋ね、患者の応答を求める（Solicit 形式であり、Voluntary 形式ではない）。

The presence or absence of adverse events will be assessed using the following items: dry mouth, astriction, dysuria, vision dysregulation (accommodation disturbance), orthostatic hypotension, sleepiness, fatigue, sleeplessness, anxiety/agitation, depression/anhedonia, lack of appetite, gain or loss of body weight, loss of sexual desire, palpitations, thrill, diaphoresis, headache, dizziness, other. Using forms, research staff will ask about any adverse event as follows, “Have you been experiencing any worsening or occurrence of physical or mental symptoms?”

有害事象の報告と発現時の対応

Response to adverse events

本研究では被験者の Visit ごとに、有害事象全体の評価を行う。有害事象が発現した場合、担当者はその内容と重篤性を評価する。担当者は出来る範囲で必要な対応を施し、研究責任者と相談の上で必要と判断され、被験者がうつ症状について何らかの治療を受けている場合には、NCNP での主治医または外部医療機関での主治医と相談し、必要な処置を施すよう求め、経過を充分観察することとする。担当者は有害事象の内容、発現日・消失日、程度、処置、転帰、重篤性評価、治療との関連性等を CRF に記載する。また、有害事象に対する治療が必要となった場合には、担当医または主治医と相談の上、担当者、担当医、または主治医が被験者にその旨を伝える。

We will evaluate the occurrence of any adverse event at every visit. In the case of an adverse event, research staff will evaluate the content and severity, and enact the necessary response. If the patient receives usual outpatient treatment in NCNP hospital or other medical institutes, the primary investigator will report the event to the primary doctors and request the necessary responses under their treatment. The primary investigator will continue the course after the occurrence of an adverse event. The research staff will record the content of the adverse event, occurrence date, date of improvement, severity, conducted response, course, and relevance to the intervention on the CRF. If additional treatment is required for the adverse event, the primary investigator, therapist, or primary doctor will provide an explanation.

予測される有害事象等

Expected adverse events

精神障害を対象とした治療において、もっとも注意が払うべき有害事象として、自殺念慮や企図が挙げられる。大規模な疫学調査によれば、大うつ病性障害における自殺企図は OR=3.2（95% CI 2.5–4.2）と報告されている(Nock et al., 2009)。その他に、上記に挙げた症状はうつ症状を有する者において予測される有害事象であると考えられる。

Generally, adverse events that should be taken into consideration are suicidal thoughts and attempts. According to the result of a large scale epidemiological survey, suicidal attempts among patients with major depressive disorder is OR=3.2 (95% CI 2.5–4.2) (Nock et al., 2009). Other expected adverse events are reflected in the items for adverse event.

17. 当該研究によって生じた健康被害に対する補償の有無及びその内容

17. Compensation for any harm caused by study participation

万が一、本研究の介入期間中に健康被害が生じた場合、適切な医療等の対応が行われるように図る。本研究では、研究に伴う健康被害のリスクは、一般の言語をコミュニケーション（面接、診療など）に伴うもの以上は特にないものと考えられるため、健康被害に対する医療も通常の診療と同様に、被験者の保険診療内で行う。なお、この本研究への参加に起因した健康被

害が生じた場合、補償金、医療費・医療手当等の補償は行われないことを、あらかじめ被験者から同意を得るものとする。

If any harm occurs due to participation in this research, the primary investigator will respond to ensure the appropriate medical care or treatment. As we do not expect the risk of any harm beyond what typically occurs for face-to-face verbal communication (e.g., interview or examination), any medical care for harm will be conducted using medical insurance as is typically used for usual care. We will provide explanations and obtain consent from subjects about the lack of compensation for any harm during participation in this research.

18. 研究対象者への研究実施後における医療の提供に関する対応

18. Response related to healthcare delivery to research subjects following research

本試験参加終了後においても、本試験に関する疑問等を被験者が抱いた場合には、研究事務局への連絡ができるものとする。

If the subject raises any questions regarding this trial after completing participation, the subject will be able to contact the inquiry counter of this research.

19. 研究対象者に係る研究結果（偶発的所見を含む。）の取扱い

19. Means of handling subjects' information (including incidental findings)

本研究において、偶発的所見が発見される可能性はほとんど存在しない。

We expect no incidental findings from participation in this trial.

20. 委託する業務内容及び委託先の監督方法

20. Content of the work to be entrusted and means of supervision over the contractors

本研究において、外部組織に業務を委託する予定はない。

No part of the work will be entrusted.

21. 試料及び情報の二次利用とそれに伴う他機関への提供の可能性

21. Possibility of secondary utilization and/or provision of the specimens and information acquired from subjects

本試験のデータが他機関にて分析されたり、他の研究のために利用されたりする具体的な予定はないものの、その可能性は否定できない。本試験の匿名化されたデータの将来的な活用については、被験者に説明し、同意を得る。

Although there is currently no plan for other institutions to analyze or utilize the data acquired by this trial, we cannot exclude this possibility. We will obtain informed consent from the subjects regarding the future utilization of anonymized data.

22. モニタリング及び監査

22. Monitoring and audit

（1）モニタリング

(1) Monitoring

本試験では、研究責任者自身が、コーディネーター及びデータ管理者と連携して、施設モニタリング及び中央モニタリングを行い、その結果を効果安全性委員に報告する。基本的には、データ管理者が CRF 又は原資料とデータベースに入力されたデータの照合（施設モニタリング）を行う。中央モニタリングは解析担当者が行い、年に一度（10 月）研究責任者から効果安全性委員に報告書が送られる。中央モニタリングは全症例に対して行い、施設モニタリングは登録された最初の 3 例を対象に行い、その後、登録第 4 – 44 番目の症例に対して、予めランダム抽出しておいた 3 例を対象に行う。どの事例をランダム抽出したかは、CRF 作成者およびデータ入力担当者には伝えないようにする。研究責任者及び効果安全性委員会の委員が必要性を指摘した場合には、モニタリング委員会を開催する。また、効果安全性委員は、データ管理者へとデータ公開を求めることができる。データ取得と入力、モニタリングの流れは以下の通りである。

The primary investigator, in collaboration with the research coordinator and data manager, will conduct on-side and central monitoring, and report the results to the members of DSMB. The data manager will verify the entered data by referring to the CRF and/or primary source material (i.e., on-site monitoring). The statistician will conduct the central monitoring. The primary investigator will report the results to members of DSMB once a year (in October). All registered cases will be subject to central monitoring. On-site monitoring will be conducted for the first three registered cases, and then for three randomly pre-identified cases from the 4th to 44th registered cases. Developer of CRF and staff for entering data will not be masked to the pre-identified cases for on-site monitoring. A meeting for monitoring will be convened if the primary investigator or members of DSMB suggest the need. In addition, the members of DSMB may request data from the data manager. The procedure for data acquisition, entry, and monitoring is as follows.

1. 研究候補者に関係する First contact（紹介等）：コーディネーターが研究用 ID を付与
2. 同意取得後、ベースライン評価：評価結果をコーディネーターが CRF に記入

3. コーディネーター作成の CRF 及び自記式尺度（原資料）を研究スタッフがデータベースに入力
4. 担当者が基礎情報に関する CRF を記入
5. プロセス指標等、介入群の評価結果を研究スタッフがデータベースに入力
6. 中間・介入後評価結果をコーディネーターが CRF に入力
7. コーディネーターが記入した CRF 及び自記式尺度（原資料）を研究スタッフがデータベースに入力

※ 随時、データ管理者が CRF 及び自記式尺度と入力されたデータの照合（施設モニタリング）
※ 中央モニタリングは解析担当者が行う
※ 中央モニタリング毎に、研究責任者が効果安全性委員に報告書を送付

1. First contact with the participant (referral, etc.): Research coordinator gives research ID to the participant.
2. Baseline assessment after obtaining informed consent: Research coordinator records the assessment results of on the CRF.
3. Research staff will enter the data by referring to the CRF and self-report measures (primary source).
4. Therapist will fill in the CRF regarding the basic information.
5. Research staff will enter the results of process measures and other assessments.
6. Research coordinator will fill in the CRF regarding the results of mid- and post-assessment.
7. Research staff will enter the information on the CRF and self-report measures.
    - Data manager will check the accuracy of data entry from the CRF and self-report measures (i.e., on-site monitoring).
    - Statistician will conduct central monitoring.
    - Primary investigator will send the central monitoring report document to the members of DSMB.

（2）監査

(2) Audit

本試験についての監査は予定していない。

No audit is planned.

23.　用語の解説

23. Explanation of terms

アンヘドニア

Anhedonia

古典的には「歓びの喪失」として定義される状態を指す（Ribot, 1896）。ただし、近年では、この定義はアンヘドニアの一側面を表現しているだけに過ぎないという指摘があり、より多面的な構成概念であることが示唆されている。具体的には、アンヘドニアは研究領域基準におけるポジティブ価システムの失調として捉えることができ、報酬獲得に対する動機づけおよびエフォートの低減、報酬獲得時の快感情の低減および持続困難、報酬獲得に関する学習の困難などを含む状態と考えられる。

Anhedonia is classically defined as the “loss of pleasure (Ribot, 1896).” Recently, this definition has been criticized as it only partially describes anhedonia, and there are suggestions that that anhedonia comprises multiple aspects. Specifically, anhedonia may be regarded as the dysregulation of the positive valence system within the framework of Research Domain Criteria, which includes the decline of motivation and effort for reward acquisition, decrease of intensity and maintenance of positive emotion at the time of reward acquisition, and difficulty in reward learning.

認知行動療法

Cognitive behavioral therapy

人はうつや不安状態になると物事のとらえ方が、より悲観的・否定的になり、その結果気分や行動が影響されるという理解にもとづき、その人の認知のあり方を治療者との対話を通じて検討し、問題解決につながる方策を案出し、実施することにより、その人の気分の改善を図ることを目的とした、構造化された精神療法の１つ。

Cognitive behavioral therapy (CBT) is a form of structured psychotherapy. CBT is based on the concept that humans experiencing depressed mood or anxiety tend to think negatively, and such cognition affects behavior. In CBT, the therapist examines patients’ cognition, elaborates, and tries ways to solve problems to improve the patients’ mood.

24. 参考文献リスト、研究に関係する指針・ガイドライン

24. References and related guidelines

1. Alexopoulos, G. S., Raue, P. J., Gunning, F., Kiosses, D. N., Kanellopoulos, D., Pollari, C., Banerjee, S., & Arean, P. A. (2016). “Engage” therapy: behavioral activation and improvement of late-life major depression, *The American Journal of Geriatric Psychiatry*, **24**(4), 320-326.

2. Ameli, R., Luckenbaugh, D.A., Gould, N. F., Holmes, M. K., Lally, N., Ballard, E.D., & Zarate, C.A. Jr. (2014) SHAPS-C: the Snaith-Hamilton pleasure scale modified for clinician administration. *PeerJ*, **17**(2), e429

3. Armento, M. E., & Hopko, D. R. (2007). The Environmental Reward Observation Scale (EROS): development, validity, and reliability, *Behavior Therapy*, **38**(2), 107-119.

4. Bang, H., Ni, L., & Davis, C. E. (2004). Assessment of blinding in clinical trials. *Controlled Clinical Trials*, **25**(2), 143-156.

5. Barlow, D. H., Gorman, J. M., Shear, M. K., & Woods, S. W. (2000). Cognitive-behavioral therapy, imipramine, or their combination for panic disorder: A randomized controlled trial. *JAMA*, **283**(19), 2529-2536.

6. Beck, A. T., Steer, R. A., & Brown, G. K. (1996). *Manual for the Beck Depression Inventory-II.* San Antonio, TX: Psychological Corporation. (小嶋雅代・古川壽亮（訳）(2003). 日本版 BDI-II—ベック抑うつ質問票—手引き　日本文化科学社).

7. Blom, E. H., Tymofiyeva, O., Chesney, M. A., Ho, T. C., Moran, P., Connolly, C. G., Duncan, L. G., Baldini, L., Weng, H. Y., Acree, M., Goldman, V., Hecht, F. M., & Yang, T. T. (2016). Feasibility and preliminary efficacy of a novel RDoC-based treatment program for adolescent depression: “Training for Awareness Resilience and Action” (TARA)—A pilot study. *Frontiers in Psychiatry*, **7,** 208.

8. Carl, J. R., Soskin, D. P., Kerns, C., & Barlow, D. H. (2013). Positive emotion regulation in emotional disorders: A theoretical review. *Clinical Psychology Review*, **33**, 343-360.

9. Clark, L. A., & Watson, D. (1988). Mood and the mundane: Relations between daily life events and self-reported mood. *Journal of Personality and Social Psychology*, **54,** 296-308.

10. Clark, L. A., & Watson, D. (1989). *The Japanese Positive and Negative Affect Schedule: Factor-based scales for the assessment of mood.* Unpublished manuscript. University of Iowa.

11. Craske, M. G., Meuret, A. E., Ritz, T., Treanor, M., & Dour, H. J. (2016). Treatment for anhedonia: A neuroscience driven approach. *Depression and Anxiety*, **33**, 927–938.

12. Cocks, K., & Torgerson, D. J. (2013). Sample size calculations for pilot randomized trials: a confidence interval approach. *Journal of Clinical Epidemiology*, **66**(2), 197-201.

13. Diener, E., Emmons, R.A., Larsen, R.J., & Griffin, S. (1985). The Satisfaction With Life Scale. *Journal of Personality Assessment*, **49**, 71-75.

14. Franken, I. H. A., Rassin, E., & Muris, P. (2007). The assessment of anhedonia in clinical and non-clinical populations: Further validation of the Snaith-Hamilton Pleasure Scale (SHAPS). *Journal of Affective Disorders*, **99**, 83-89.

15. Farchione, T. J., Fairholme, C. P., Ellard, K. K., Boisseau, C. L., Thompson-Hollands, J., Carl, J. R., Gallagher, M. W., & Barlow, D. H. (2012). Unified protocol for transdiagnostic treatment of emotional disorders: A randomized controlled trial. *Behavior Therapy*, **43**(3), 666–678.

16. Hamilton, M. (1960). A rating scale for depression. *Journal of Neurology, Neurosurgery & Psychiatry*, **23**, 56-62.

17. Harvey, A. G., Lee, J., Smith, R.L., Gumport, N.B., Hollon, S. D., Rabe-Hesketh, S., Hein, K., Dolsen, M. R., Hman, K. L., Kanady, J. C., Thompson, M.A., & Abrons, D. (2016). Improving outcome for mental disorders by enhancing memory for treatment. *Behaviour Research and Therapy*, **81**, 35-46.

18. Insel T, Cuthbert B, Garvey M, Heinssen R, Pine DS, Quinn K, Sanislow, C., & Wang, P. (2010). Research domain criteria (RDoC): toward a new classification framework for research on mental disorders. *American Journal of Psychiatry*, **167**, 748–751.

19. Kitamura, T., Kishida, Y., Katayama, R., Matsuoka, T., Miura, S. & Yamabe, K. (2003). Ryff's psychological well-being inventory: factorial structure and life history correlates among Japanese university students. *Psychological Reports*, **94**, 83-103.

20. Kojima, M., Furukawa, T. A., Takahashi, H., Kawai, M., Nagaya, T., & Tokudome, S. (2002). Cross-cultural validation of the Beck Depression Inventory-II in Japan. *Psychiatry Research*, **110**, 291-299.

21. 国里愛彦・高垣耕企・岡島義・中島俊・石川信一・金井嘉宏・岡本泰昌・坂野雄二・山脇成人. (2011). 日本語版 Environmental Reward Observation Scale (EROS) の作成と信頼性・妥当性の検討. 行動療法研究, **37**(1), 21-31.

22. 文部科学省・厚生労働省. (2014). 人を対象とする医学系研究に関する倫理指針.

23. 文部科学省・厚生労働省. (2015). 人を対象とする医学系研究に関する倫理指針　ガイダンス.

24. Nagayama, H., Kubo, S., Hatano, T., Hamada, S., Maeda, T., Hasegawa, T., Kadowaki, T., Terashi, H., Yoshioka, M., Nomoto, N., Kano, O., Inoue, M., Shimura, H., Takahashi, T., Uchiyama, T., Watanabe, H., Kaneko, S., Takahashi, T., & Baba, Y. (2012). Validity and reliability assessment of a Japanese version of the Snaith–Hamilton pleasure scale. *Internal Medicine.* **51,** 865–869.

25. National Collaborating Centre for Mental Health. (2010). Depression: the treatment and management of depression in adults. Updated ed. Leicester: British Psychological Society/London: Royal College of Psychiatrists.

26. Nock, M. K., Hwang, I., Sampson, N. A., & Kessler, R. C. (2009). Mental disorders, comorbidity and suicidal behavior: Results from the National Comorbidity Survey Replication. *Molecular Psychiatry*, **15**(8), 868–876.

27. Oohashi, T., Nishina, E., Honda, M., Yonekura, Y., Fuwamoto, Y., Kawai, N., Maekawa, T., Nakamura, S., Fukuyama, H., & Shibasaki, H. (2000). Inaudible high-frequency sounds affect brain activity: hypersonic effect. *Journal of Neurophysiology*, **83**, 3548-3558.

28. Ori, R., Amos, T., Bergman, H., Soares-Weiser, K., Ipser, J. C., & Stein, D. J. (2015). Augmentation of cognitive and behavioural therapies (CBT) with d-cycloserine for anxiety and related disorders. *Cochrane Database of Systematic Reviews*, **5**, Art. No.: CD007803. DOI: 10.1002/14651858.CD007803.pub2.

29. Otsubo, T., Tanaka, K., Koda, R., Shinoda, J., Sano, N., Tanaka, S., Aoyama, H., Mimura, M., Kamijima K. (2005). Reliability and validity of Japanese version of the mini-international neuropsychiatric interview. *Psychiatry and Clinical Neurosciences*, **59**(5), 517-526.

30. Pelizza, L., & Ferrari, A. (2009). Anhedonia in schizophrenia and major depression: state or trait? *Annals of General Psychiatry,* **8**(22), doi: 10.1186/1744-859X-8-22

31. Pizzagalli, D. A. (2014). Depression, stress, and anhedonia: toward a synthesis and integrated model. *Annual Review of Clinical Psychology,* **10,** 393–423.

32. Primakoff, L., Epstein, N., & Covi, L. (1986). Homework compliance: An uncontrolled variable in cognitive therapy outcome research. *Behavior Therapy*, **17**, 433–446.

33. Ribot T. (1896). La Psychologie des Sentiment [The Psychology of Feelings]. Paris: Felix Alcan.

34. Roll, D., Ray, S. E., Marcus, S. M., Passarelli, V., Money, R., Barlow, D. H., Wood, S. W., Shear, M K., & Gorman, J. M. (2004). Independent evaluator knowledge of treatment in a multicenter comparative treatment study of panic disorder. *Neuropsychopharmacology,* **29**(3), 612-618.

35. Ryff, C. D. (1989). Beyond Ponce de Leon and life satisfaction: new directions in quest of successful aging. *International Journal of Behavioral Development*, **12**, 35-55.

36. Snaith, R.P., Hamilton, M., Morley, S., Humayan, A., Hargreaves, D., & Trigwell, P. (1995). A scale for the assessment of hedonic tone the Snaith–Hamilton Pleasure Scale. *British Journal of Psychiatry*. **167**, 99–103.

37. Sheehan, D. V., Lecrubier, Y., Harnett-Sheehan, K., Amorim, P., Janavs, J., Weiller, E., Hergueta, T., Baker, R., & Dunbar, G. (1998). The Mini International Neuropsychiatric Interview (M.I.N.I.): The Development and Validation of a Structured Diagnostic Psychiatric Interview. *Journal of Clinical Psychiatry*, **59**(20), 22-33.

38. 角野善司. (1994). 人生に対する満足度尺度（the Satisfaction With Life Scale [SWLS]）日本語版作成の試み. 日本教育心理学会総会発表論文集, **36**, 192.

39. 大石繁宏. (2009). 幸せを科学する一心理学からわかったこと. 新曜社

40. Tabuse, H., Kalali, A., Azuma, H., Ozaki, N., Iwata, N., Naitoh, H., Higuchi, T., Kanba, S., Shioe, K., Akechi, T., & Furukawa, T. A. (2007). The new GRID Hamilton Rating Scale for depression demonstrates excellent inter-rater reliability for inexperienced and experienced raters before and after training. *Psychiatry Research*, **153**(1), 61–67.

41. Taylor, C. T., Lyubomirsky, S., and Stein, M. B. (2017). Upregulating the positive affect system in anxiety and depression: Outcomes of a positive activity intervention. *Depression and Anxiety*, **34**, 267–280.

42. Treadway, M. T., Buckholtz, J. W., Schwartzman, A. N., Lambert, W. E., & Zald, D. H. (2009). Worth the ‘EEfRT’? The effort expenditure for rewards task as an objective measure of motivation and anhedonia. *PLoS ONE*, **4**(8), e6598.

43. Williams, J. B., Kobak, K. A., Bech, P.,. Evans, K., Lipsitz, J., Olin, J., Pearson, J., & Kalali, A. (2008). The GRID- HAMD: standardization of the Hamilton Depression Rating Scale. *International Clinical Psychopharmacology*, **23**(3), 120–129.

44. Vos, T., Barber, R. M., Bell, B., Bertozzi-Villa, A., Biryukov, S., Bolliger, I., et al. (2015). Global, regional, and national incidence, prevalence, and years lived with disability for 301 acute and chronic diseases and injuries in 188 countries, 1990–2013, a systematic analysis for the Global Burden of Disease Study 2013. *Lancet*, **386**(9995), 743–800.

研　究　計　画　書

Study Protocol

第 2 版：2018 年 2 月 20 日

Second version: 20th, February 2018

研究課題名	**アンヘドニアに対するポジティブ価システムに焦点を当てた認知行動療法の超高周波音響療法による増強効果：プラセボ対照ランダム化比較試験**
Research title	**Augmentation of positive valence system-focused cognitive behavior therapy by inaudible high-frequency sound therapy: A placebo-controlled randomized trial**
研究責任者（所属）	**伊藤正哉（認知行動療法センター研修指導部）**
Principal Investigator	**Masaya Ito, Ph.D（National Center of Cognitive-Behavior Therapy and Research, National Center of Neurology and Psychiatry）**

1. 研究の名称

1. Research title

アンヘドニアに対するポジティブ価システムに焦点を当てた認知行動療法の超高周波音響療法による増強効果：プラセボ対照ランダム化比較試験

Augmentation of positive valence system-focused cognitive behavior therapy by inaudible high-frequency sound therapy: A placebo-controlled randomized trial

2. 研究の実施体制

2. Site-specific research information

【国立精神・神経医療研究センターにおける共同研究者】

【Study members in National Center of Neurology and Psychiatry】

氏名 Name	所属 Affiliations	研究における役割及び責務 Roles and responsibilities	倫理講座の受講の有無（1 年以内） Experience in research ethics workshop participation	本研究に関する利益相反申告状況 Status of conflict of interest declaration
伊藤正哉 Masaya Ito	認知行動療法センター National Center of Cognitive-Behavior Therapy, National	研究責任者、研究デザイン、認知行動療法の開発と実施	あり Yes	2017 年 9 月 6 日提出済 Submitted on 6th of September, 2017

	Center of Neurology and Psychiatry	Principal investigator, study design, development of cognitive behavioral therapy protocol and implementation		
堀越勝<br>Masaru Horikoshi	認知行動療法センター<br>National Center of Cognitive-Behavior Therapy, National Center of Neurology and Psychiatry	研究デザイン<br><br>Study design	あり<br><br>Yes	2017年9月6日提出済<br>Submitted on 6th of September, 2017
本田学<br>Manabu Honda	神経研究所<br>National Institute of Neuroscience, National Center of Neurology and Psychiatry	音響プロトコルの開発と運用<br>Development of protocol and management of audio intervention	あり<br><br>Yes	2017年9月6日提出済<br>Submitted on 6th of September, 2017
山下祐一<br>Yuichi Yamashita	神経研究所<br>National Institute of Neuroscience, National Center of Neurology and Psychiatry	音響プロトコルの開発、音響機器のメンテナンス 割付の順番作成<br>Development of audio intervention protocol, maintenance of audio devices, generation and storage of comparison table of randomized sequences and sound tracks	あり<br><br>Yes	2017年9月6日提出済<br>Submitted on 6th of September, 2017

宮前光宏<br>Mitsuhiro Miyamae	神経研究所<br>National Institute of Neuroscience, National Center of Neurology and Psychiatry	研究デザイン、認知行動療法の開発と実施、症状評価、附属研究の主任研究者<br>Study design, development of cognitive behavioral therapy protocol and implementation, symptom assessment, PI for ancillary study	あり<br><br>Yes	2017 年 9 月 6 日提出済<br>Submitted on 6th of September, 2017
上野修<br>Osamu Ueno	神経研究所<br>National Institute of Neuroscience, National Center of Neurology and Psychiatry	音響プロトコルの開発と運用、音響機器のメンテナンス<br>Development of protocol and management of audio intervention, maintenance of audio devices	あり<br><br>Yes	2017 年 9 月 6 日提出済<br>Submitted on 6th of September, 2017
横山知加<br>Chika Yokoyama	認知行動療法センター<br>National Institute of Neuroscience, National Center of Neurology and Psychiatry	認知行動療法の開発と実施、試験コーディネート<br>Development of cognitive behavioral therapy protocol and implementation, coordination of trial	あり<br><br>Yes	2017 年 9 月 6 日提出済<br>Submitted on 6th of September, 2017
伊藤まどか<br>Madoka	精神保健研究所成人精神保健研究部・流動研究員	認知行動療法の開発<br>Development of	あり<br><br>Yes	2017 年 9 月 6 日提出済<br>Submitted on 6th

Ito	National Institute of Mental Health, Neuroscience, National Center of Neurology and Psychiatry	cognitive behavioral therapy protocol		of September, 2017
丸尾和司<br>Kazushi Maruo	トランスレーショナル･メディカルセンター<br>Translational Medical Center, National Center of Neurology and Psychiatry	解析計画書の作成と、解析の実施<br>Development of statistical analysis plan and statistical analysis	あり<br><br>Yes	2017 年 9 月 6 日提出済<br>Submitted on 6th of September, 2017
駒沢あさみ<br>Asami Komazawa	認知行動療法センター<br>National Institute of Neuroscience, National Center of Neurology and Psychiatry	認知行動療法の開発と実施<br>Development of cognitive behavioral therapy protocol and implementation	あり<br><br>Yes	2017 年 9 月 6 日提出済<br>Submitted on 6th of September, 2017

【国立精神・神経医療研究センターにおける共同研究者以外の研究協力者の実施体制】

【Personnel external to National Center of Neurology and Psychiatry】

氏名<br>Name	所属・役職<br>Affiliations	研究における役割及び責務<br>Roles and responsibilities	倫理講座の受講の有無（1 年以内）<br>Experience in research ethics workshop participation	本研究に関する利益相反審査結果<br>Status of conflict of interest declaration
田島美幸<br><br>Miyuki Tajima	認知行動療法センター<br><br>National Institute of Neuroscience, National Center of Neurology and Psychiatry	個人情報管理者・個人情報匿名化担当者<br>Administrator of anonymization management	あり<br><br>Yes	9 月 6 日提出済<br><br>Submitted on 6th of September, 2017

【効果安全性評価委員会】

【Members of Data Safety Monitoring Board】

所属<br>Affiliation	氏名<br>Name	専門<br>Expertise
武蔵野大学<br>Musashino University	中島聡美<br>Satomi Nakajima, M.D., Ph.D.	精神医学<br>Psychiatry
東京大学<br>Tokyo University	西大輔<br>Daisuke Nishi, M.D., Ph.D.	精神医学<br>Psychiatry

【モニタリング】

【Monitoring】

所属<br>Affiliations	氏名<br>Name	研究における役割及び責務<br>Roles and responsibilities
認知行動療法センター<br>National Center of Cognitive-Behavior Therapy, National Center of Neurology and Psychiatry	伊藤正哉<br>Masaya Ito	モニタリング責任者<br>Responsible for monitoring

3. 研究の背景、科学的合理性の根拠及び社会的意義

3. Research background, scientific validity, and social significance

精神疾患が個人と社会に及ぼす損失は甚大である。なかでも、うつ病は世界の疾病負担の 3 位である（Vos et al., 2015)。精神疾患はわが国の 5 大疾病のひとつであり、最も患者数が多い（約 323 万人)。うつ病と不安障害の 1 年間の時点有病率は 7.9%である。うつ病の 1 年間の社会経済コストは年間 3 兆 900 億円に上る。

認知行動療法(Cognitive Behavioral Therapies; CBT)は、学習理論や認知理論に基づき、治療手続きが体系化された精神療法の総称である。診療ガイドラインや系統的レビューによると、中等症以上のうつ病(大うつ病性障害、持続性抑うつ障害)には、認知行動療法が推奨されている（e,g., 英国医療技術評価機構（National Collaborating Centre for Mental Health）, 2010)。日本においても、うつ病に対する認知行動療法が健康保険適用となった。現在の日本では、認知行動療法をいかに均てん化するかについての努力が展開しているところである。

Mental disorders cause devastating effects at both the individual and societal level. Among them, depressive disorder ranks third as a cause of global disease burden (Vos et al., 2015). Mental disorders are designated as one of the big-five diseases in Japan and together constitute the largest patient population among these big-five diseases (more than 3 million and 230,000 patients in Japan). The annual prevalence of depressive and anxiety disorders in Japan is reported to be 7.9%. The annual socio-economic cost of depressive disorder is estimated to be more than 3 trillion yen.

Cognitive behavioral therapy (CBT) is a form of psychotherapy with systematic intervention

protocols based on learning and cognitive theories. According to treatment guidelines and the results of systematic reviews, cognitive behavioral therapy is recommended for treating depression with moderate or severe symptoms (major depressive disorder and dysthymia) (e.g., National Institute of Clinical Excellence, 2010). In Japan, CBT for depression was subjected to national medical insurance beginning in 2013. There is a current focus to increase the use of CBT in the Japanese medical setting.

うつ病治療において、いまや認知行動療法は第一治療選択のひとつである。しかし、依然として改良の余地も大きい。認知行動療法を受けても約半数が再発したり、治療反応を示さない患者も一定数いることが知られている。そうした背景から、認知行動療法の効果を増強させる先端研究が展開されつつある。例えば、d-cycloserine という物質（もともとは結核薬）を認知行動療法の前に投与することにより、不安症に対する認知行動療法で行われる“情報の再学習”が強化される可能性が期待されている。他にも、増強を検討するさまざまな研究が芽生えつつある（治療中の記憶力の増強（Harvey et al., 2016）。

CBT is currently the first-line treatment for depression. Nevertheless, there is significant scope for improvement. Approximately half of patients show relapse of depression after the completion of CBT. A substantial proportion of patients do not respond to CBT. Based on these shortcomings, there have been increased research efforts to augment the effect of CBT for depression. For example, d-cycloserine, originally utilized for treating tuberculosis, is used to augment “information re-learning” during CBT for anxiety disorders. Other techniques for augmenting the efficacy of CBT have also been used, such as augmentation of memory technique (Harvey et al., 2016).

われわれは、認知行動療法を増強させる方法として、“アンヘドニアに対するポジティブ価システムに焦点を当てた認知行動療法”と“ハイパーソニック・エフェクト（超高周波音響効果, Hypersonic Effect）”に着目した。まず、ポジティブ価システムに焦点を当てる認知行動療法とは、うつ病の中でもアンヘドニア症状を改善することに焦点を当てた認知行動療法である。アンヘドニア症状は従来の治療では改善されにくい症状であり、ポジティブ感情の低下、意欲の減退、報酬に対する感受性の低下、歓びの喪失などの様々な定義が与えられている。近年提唱された研究領域基準（Insel et al., 2010）においては、このアンヘドニア症状はポジティブ価システム（Positive-Valence System）の失調として捉えられる。ポジティブ価システムの下位概念として、接近動機づけ、報酬獲得への初発反応性、報酬獲得への反応性の維持と長期反応性、報酬学習、習慣が含まれている。近年の認知行動療法は、神経科学とポジティブ心理学の知見を導入し、これらのシステムを特に強化するための介入を導入し、うつ病治療の改善を検証しつつある（Craske et al., 2016, Taylor et al., 2016, Alexopoulos et al., 2016, Blom et al., 2016）。

We focused on the “positive valence system-focused CBT for anhedonia” and “hyper-sonic

effect” (effect of inaudible hyper sound) for this study. Positive valence system-focused CBT is a CBT that focuses on improving anhedonia. Anhedonia is defined as decreased positive emotions, decreased motivation for performing activities, deceased sensitivity to rewarding stimuli, or loss of pleasure. Anhedonia is known to be resistant to conventional treatments for depression. In the context of Research Domain Criteria (Insel et al., 2010), anhedonia can be classified as a dysfunction of the positive valence system. Sub-constructs of the positive valence system are constituted by approach motivation, initial responsiveness to reward attainment, sustained/longer-term responsiveness to reward attainment, reward learning, and habit. Recent studies on CBT have attempted to incorporate findings from neuroscience and positive psychology to augment these systems and test their efficacy (Craske et al., 2016; Taylor et al., 2016; Alexopoulos et al., 2016; Blom et al., 2016）.

一方で、ハイパーソニック・エフェクトとは、人間の可聴域上限を超える超高周波成分を豊富に含む音響情報が、報酬系神経回路を含む脳深部の神経活動を活性化し、人体に全身的影響を及ぼすことを指す。これまで研究協同者（本田学）の研究グループは、この現象を複数の非侵襲脳機能イメージングと様々な生理活性指標を用いて明らかにしてきた（Oohashi et al., 2000 他）。具体的には、超高周波成分を豊富に含む音情報は、同じ音から超高周波成分を除去した音情報（ハイカット音）と比較して、脳幹、視床から前頭前野に拡がるモノアミン作動性神経投射を含む情動系神経回路や報酬系回路の血流を増加させるとともに、それと並行して脳波 α 波のパワーを増強させること、視床下部の活性化を反映して NK 細胞活性を上昇させ、ストレスホルモンを低下させるといった全身反応を導くことを明らかにした。加えて、超高周波成分を含む音情報は、音質を向上し音の快適性を増強させるとともに、超高周波成分を豊富に含む音をより多く受容しようとする接近行動を引き起こすなど、報酬系の活性化を反映した被呈示者の心理行動的効果を導く。

The hyper-sonic effect refers to the whole-body effect from exposure to inaudible high-frequency sound via the activation of deep brain activity including reward related neural circuits. Our research group has demonstrated the phenomenon using various non-invasive brain function imaging and physiological measures (Ohashi et al., 2000). For example, we have demonstrated that inaudible high-frequency sound, in comparison to high-cut placebo sound, increases blood flow in the reward circuity and affects related circuity including monoaminergic projections distributed across the brain stem, thalamus, and prefrontal region, in parallel with enhancement of alpha brain-wave power. Compared to high-cut placebo sound, inaudible high-frequency sound enhanced natural killer cell activation, which reflects hypothalamic activity and decreased stress hormone levels. In addition, inaudible high-frequency sound promoted psycho-behavioral effects that reflect activation of the reward system. Therefore, inaudible high-frequency sound seems to robustly enhance the subjective experience of sound quality and

comfort, and promotes approach behavior to listen to the sound.

こうした複雑に変化する超高周波成分は、人間の遺伝子が進化的に形成されたと考えられる熱帯雨林の環境音に豊富に含まれる一方で、現代人の多くが生活する都市環境音にはほとんど含まれない。そこで研究分担者らは、自然環境音に豊富に含まれ現代社会の環境音にほとんど含まれない複雑性をもった超高周波成分という「必須情報」の不足が、深部脳を起点とするモノアミン神経系の変調を介して、気分障害をはじめとする精神・神経疾患の発症に無視できない影響を及ぼす可能性があるのではないかとの仮説を立てた。この仮説のもと、平成 22〜24 年度の厚生労働科研費医療技術実用化総合研究事業では、超高周波成分を豊富に含む音響情報の曝露の前後で、うつ病患者の状態不安指標が有意に改善することを示した。

Although such inaudible high-frequency sound with complex, changing sound quality is prevalent in environmental sounds in tropical rain forests, it is absent in urban city settings in which most modern people around the world live. We hypothesized that inaudible high-frequency with complex sound quality could be conceptualized as “essential information”, and the lack of this could have considerable effects on the onset of mental and neurological disorders via the dysfunction of monoaminergic neural systems. Based on this hypothesis, we have demonstrated that the exposure to sound information with inaudible high-frequency sound significantly improved state anxiety among patients with depression.

このように、ハイパーソニック・エフェクトは様々なイメージング・生理・心理・行動指標で観察されてきた。なかでも今回われわれが注目したメカニズムとして、うつ病患者の精神症状の改善効果と、学習効果の増強に関する知見を指摘できる。前者においては、超高周波音響の呈示によって、脳内の報酬系回路が活性化させることにより、「必須情報」が補完された結果として解釈できる。認知行動療法においても、報酬系回路を活性化させる治療要素（行動活性化）が含まれているため、そうした治療要素との相乗効果が期待できる。さらに、ハイパーソニックの学習効果増強に関する研究では、超高周波音響を呈示されながら実施される認知課題（N-back 課題）では、呈示がない場合よりも優れた成績が示された。先に述べた通り、認知行動療法は“情報の再学習”を治療媒介とする。そのため、ハイパーソニック・エフェクトによる学習効果の増強は、認知行動療法の効果増強にも応用できると考えられる。

This hyper-sonic effect has been observed in various imaging, physiological, psychological, and behavioral measures. Among these, we focused on two findings of the hyper-sonic effect: the improvement of mental status among patients with depression, and the enhancement of learning. The former could be interpreted as a supplement of “essential information” via activation of brain reward circuity by exposure to inaudible high-frequency sound. One of the intervention techniques

in CBT, behavioral activation, is aimed at activating the reward circuity. Therefore, it is expected that inaudible high-frequency sound and behavioral activation may have a synergistic effect. Furthermore, the latter findings showed that performance on a cognitive task (N-back task) was superior in groups exposed to inaudible high-frequency sound compared to groups not exposed to the sound. As discussed above, one of the treatment mechanisms of CBT is “re-learning of information.” Hence, enhancement of learning performance by the hyper-sonic effect may be applicable for augmenting CBT.

4. 研究の目的及び意義

4. Research objectives and significance

上述の背景から、本研究ではポジティブ価システムに焦点を当てた認知行動療法と超高周波音響を併用することにより、うつ症状のなかでもとくにアンヘドニアが顕著に改善されるかどうかを検討することを目的とした。すなわち、本臨床試験は、アンヘドニア症状を呈する成人 44 名を被験者対象集団とし、ポジティブ価システムに焦点を当てた認知行動療法に超高周波音響を呈示した試験治療の、同治療に超高周波を含まないプラセボ音響を呈示した対照治療に対する、Snaith-Hamilton Pleasure Scale にて測定されるアンヘドニア症状への有効性に対する優越性を検証することを目的とする。

Based on the rationale discussed above, we aimed to test the efficacy of combining the positive valence system-focused CBT with inaudible high-frequency sound to improve anhedonia. The objective of this clinical trial is to test the efficacy of the positive valence system-focused CBT with inaudible high-frequency sound compared to the positive valence system-focused CBT with placebo sound on anhedonia symptoms (Snaith-Hamilton Pleasure Scale) among 44 patients with anhedonia.

本研究では、さまざまな認知行動療法の中でも、アンヘドニアに対するポジティブ価システムに焦点を当てた介入技法がハイパーソニック・エフェクトにより増強されるかどうかを検証する。もしその増強効果が同定されれば、今後はより大規模な検証的ランダム化比較試験に進む。同時に、様々な疾患（e.g., 全般不安症、社交不安症、パニック症、心的外傷後ストレス障害など）や認知行動療法の他の治療要素（e.g., モニタリング、認知再構成、エクスポージャー、マインドフルネス）への適用へと拡張させて増強効果を検証する。検証的試験により、パイパーソニック・エフェクトによる増強効果が確認できれば、これまで認知行動療法に反応しなかった患者や、再発を呈していた患者に対して、新たな治療選択肢を提示することができるようになる。

Among the various forms of CBT, we will test the augmentation effect for intervention techniques that are focused on the positive valence system for anhedonia. If augmentation effects are observed in this study, we will proceed to a larger confirmatory randomized controlled trial. In

parallel, we will extend research on this augmentation effect for various disorders (e.g., generalized anxiety disorder, social anxiety disorder, panic disorder, posttraumatic stress disorder, etc.) and various treatment components of CBT (e.g., monitoring, cognitive restructuring, exposure, and mindfulness). Confirming the augmentation of CBT by the hyper-sonic effect will enable new treatment choices for patients who do not respond to CBT or who experience relapse following the completion of CBT.

本研究で増強効果が観察できた場合には、その科学的根拠は多方面に重要なインパクトを持つ。有効な精神療法は認知行動療法以外にも指摘されている。また、そもそも薬物療法などの“物質”療法においても、医療者と患者間のコミュニケーション、すなわち“情報の相互伝達”を基盤として医療が行われる。このように、他の精神療法や、医療コミュニケーションにおける情報伝達と学習の効率化にハイパーソニックが寄与できる可能性への扉が開かれることになる。

If we are able to prove an augmentation effect, this will have impact on various fields. Effective psychotherapy is not limited to CBT. Pharmacotherapy is also based upon the communication or reciprocal exchange of information between physicians and patients. This study may open the door to the potential contribution of the hyper-sonic effect on information exchange in medical communication and learning efficiency.

また、本研究は先端的電子情報技術というわが国の強みを最大限に活用したアプローチをとる。音響療法を含む情報技術を応用した統合医療という大きな未来性を持つ学術・産業領域を、わが国先導の下に世界に提案するとともに、電子情報通信産業やメディア産業など、異分野から医療分野への効果的で摩擦の少ない参入を促すことが期待される。

This study adopts an approach that fully utilizes advanced electronic information technology, which is one of Japan’s strong points. This study takes the initiative of promoting integrative medicine utilizing information technology including sound therapy as highly promising academic and industrial endeavors. Furthermore, this study is expected to effectively promote the integration of the electronic information technology and media industries with the medical field.

5. 研究の方法及び期間

5. Research methods and timeline

（1）研究実施期間

（1）Research timeline

倫理委員会承認後から 2020 年 3 月 31 日まで

（研究対象者登録締切予定日：2019 年 5 月 31 日）

（倫理委員会承認後から 12 月にかけて、3 症例に対して、本研究のフローに従った予備試行を行う。その上で、必要に応じて変更申請を行う。）

From the date of IRB approval to 31st March, 2020

(Planned date of the final registration of participant, 31st May, 2020)

(After the date of IRB approval, we will conduct three pilot trial cases that is followed by procedure of the main trial to confirm the feasibility. If needed, we will submit the modified protocol to IRB.)

（2）研究の種類・デザイン

（2）Trial design

個人割り付け、治療介入、探索的、無作為化、マスキング（治療者、患者）、並行群間比較、プラセボ対照、単施設、第 II 相試験

Individual-level allocated, treatment intervention, exploratory, randomized, therapist and patient masked, parallel group, placebo controlled, single-site, phase II trial

（3）予定する研究対象者数

（3）Targeted sample size

本研究は先行研究が存在せず、事前に症例数設計で用いる適切な効果量を推定することが困難である。本研究で狙いとする認知行動療法の増強効果という点で参考になる文献として、不安症に対する認知行動療法の d-cycloserine による増強効果のメタアナリシスが報告されている。この報告では、諸種のアウトカムでの増強効果の基準化効果量が 0.07〜0.58 であった（Ori et al., 2015）。この範囲の中でも、本研究の増強効果では比較的小さい効果サイズを想定するのが妥当であると考えられる。そこで、臨床的意義のある最小限の基準化効果量を 0.2〜0.3 と設定し、パイロット RCT の精度に基づく症例数設計手順(Cocks & Torgerson, 2013)から、1 群 20 例とした。これにうつ病に対する認知行動療法の脱落率 12.1% を考慮して、本研究では 2 群で計 44 例を目標症例数として設定した。なお、この症例数には、予備試行で行われる 3 症例を含めない。

平成 30 年 4 月－平成 32 年 1 月までの 34 ヶ月間に、毎月 2–3 例程度の登録を予定している。

It is difficult to estimate the appropriate sample size to be used as there are no previous studies directly related to this trial. It would be informative to refer to the results of a meta-analysis of the augmentation effect of d-cycloserine on CBT for anxiety disorders. It was reported that the standardized effect size of the augmentation effect on various outcomes ranged from 0.07–0.58 (Ori et al., 2015). Based on this range, we conservatively selected a relatively low effect size for the augmentation effect of inaudible high-frequency sound on the efficacy of CBT. Hence, we set the

standardized effect size as 0.2–0.3 as the clinically significant minimum level. Following the procedure of sample size estimation for pilot randomized controlled trial (Cocks & Torgerson, 2013), we set the sample size per group as 20. Considering the reported proportion of drop-outs (12.1%), we set 44 as the targeted total sample size. Before starting this main pilot trial, we will conduct an external preliminary trial with three patients to examine the feasibility of this trial.

We estimate a registration of two to three patients per month from April 2018 to January 2020.

（4）研究のアウトライン

（4）Research outline

本研究への参加は計 19 週間であり、組入れ期間が-4–0 週、介入期間が 1–11 週、介入後評価が 12 週、追跡評価が 21 週に実施される。タイムラインを Fig.1（次ページ）に示す。この間、患者は通常の診療を続ける。本研究に関係する部分は、Fig.1 にあるように、説明と同意、評価、登録、介入 8 セッション、である。

Participation in this study is for 19 weeks: enrolment period from -4 to 0-week, intervention period from 1 to 11-week, post-intervention assessment at 12-week, and follow-up assessment at 21-week. The timeline is depicted in Fig. 1. During the study period, all patients will continue treatment as usual. Activities related to this trial are informed consent, assessment, registration, and 8 session intervention (Fig. 1).

本臨床試験の広報は、認知行動療法センターのホームページおよび病院内に設置したパンフレットを通して行う。国立精神・神経医療研究センター病院に外来通院する患者を対象として、患者本人の参加希望があれば、主治医と相談してもらうよう広報する。主治医の許可が得られれば、主治医から精神リハビリテーション部の認知行動療法初診に紹介していただくようにする。認知行動療法初診後に、臨床心理室で開かれているカンファレンスにおいて、本研究への紹介が妥当と判断されれば、本研究への紹介となり、患者とコンタクトをとり、説明と同意へと進む。

This clinical trial will be advertised on the National Center of Cognitive-Behavior Therapy and Research website and with pamphlets placed in the hospital. This study is intended to recruit outpatients in the NCNP hospital. If patients are willing to participate in the trial, we encourage the patient to first consult their main doctors. After permission has been obtained from their doctors, they will be referred to the intake for CBT at the department of psychiatric rehabilitation in the NCNP hospital. After the intake, treatment indications, including referral to this study, will be examined at weekly meetings attended by staff including psychiatrists and clinical psychologists in the department. If the patient is subsequently referred to this study, the research coordinator will contact the patients to proceed with informed consent.

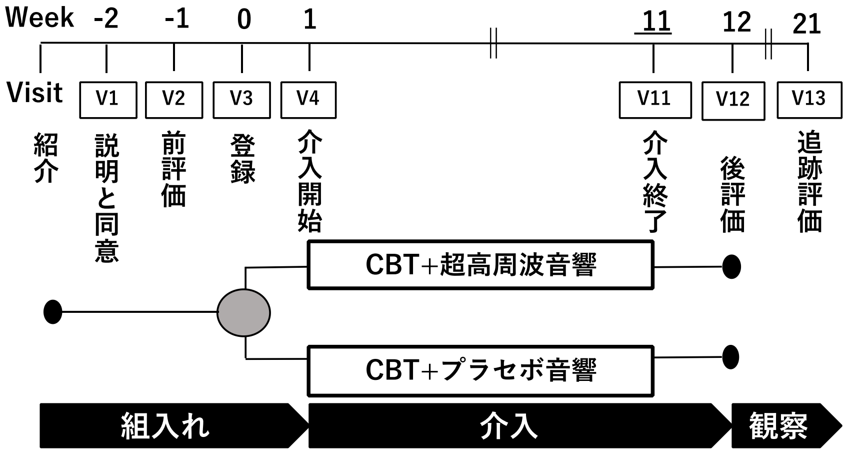

Fig.1 試験のタイムライン

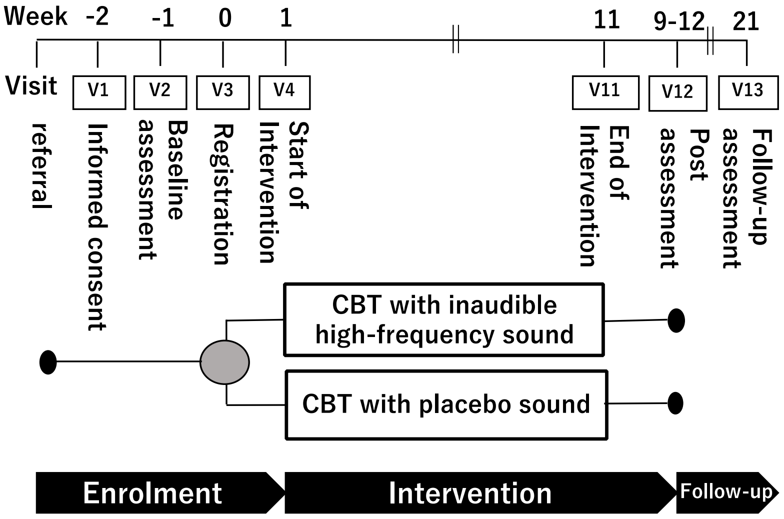

Fig.1 Timeline of the trial

(5) 研究に用いる医薬品・医療機器、治療法等の情報

(5) Medicinal drugs, devises, or interventions for this study

アンヘドニアに対するポジティブ価システムに焦点を当てた認知行動療法(**Positive-valence system focused Cognitive-Behavioral Therapy for Anhedonia; PoCot**)：

ポジティブ価システムに焦点を当てたアンヘドニアに対する認知行動療法(**Positive-valence system focused Cognitive-Behavioral Therapy for Anhedonia; PoCot**)は、うつに対する認知行動療法の中でも行動活性化で用いられる技法を土台としつつ、近年のポジティブ価システムに介入する内容（Craske et al., 2016, Taylor et al., 2016,

Alexopoulos et al., 2016, Blom et al., 2016）を参考に開発したものである。理論的な背景としては、報酬系に関する神経科学(Pizzagalli, 2014)、精神病理学(Pelizza & Ferrari, 2009; Franken et al., 2007)、そして感情調整研究（Carl et al., 2013）を基盤としている。基本的な治療原理は、うつ病という病態が報酬系情報システムが機能していない状態であるという理解のもとに、報酬系に関わるシステムが活性化されるような体験に従事することで、報酬系システムとそれが関与するうつという病態が改善されるという仮定に立っている。治療内容は下記の(6)に示す通りである。

Positive valence system-focused Cognitive-Behavioral Therapy for Anhedonia; PoCot：

The PoCot is developed based on the behavioral activation for depression and recently proposed interventions for positive valence system (Craske et al., 2016; Taylor et al., 2016; Alexopoulos et al., 2016; Blom et al., 2016). Theoretically, this program is based on the neuroscience of the reward system (Pizzagalli, 2014), psychopathology underlying anhedonia (Pelizza & Ferrari, 2009; Franken et al., 2007), and emotional regulation (Carl et al., 2013). The central treatment rationale is based on the concept of depression as a dysfunction of the reward-related information system (i.e., positive valence system). It is assumed that reward-related systems may be improved by engaging rewarding behaviors and fully experiencing positive emotions.

超高周波音響とプラセボ音響：

超高周波音響は、人の可聴域上限をこえる超高周波成分を豊富に含む熱帯雨林の自然環境音を用いる。プラセボ音響は、超高周波音響と同一の音源から高周波成分のみを取り除いた音響を用いる。音響提示装置には研究参加者自身では操作できない安全装置（操作パネルカバーなど）を装備し、装置の制御は実験スタッフが行う。音響情報の種類については、これまでの健常人を対象とした一連の実験室実験や、市街地で多数の健常者を対象とした実験、外来うつ病患者を対象とした実験などで使用実績があり、問題が生じていない音源を、本研究の目的のために特別に編集して用いる。音響情報の呈示音量は、これまでの実験同様、室内の会話の支障にならない低音量とし、その最適音量を検討する。

Inaudible high-frequency sound and placebo sound:

We will use natural environmental sounds that have been proven to include many inaudible high-frequency sounds for auditory intervention. We will exclude the inaudible high-frequency sound component from the same natural environmental sound source, and use this as placebo sound. The operational panel of sound equipment will be concealed to prevent participants from touching and/or manipulating it. Operation of the sound equipment will be conducted by the therapist. The sound source has been used in laboratory experiments for healthy populations, large scale experiments in urban districts, and experiments for outpatients with depressive

disorder. No adverse events or related problems were reported in these studies. Similar to previous studies, the volume of sound will be low to avoid disturbing the natural conversation in the room.

（6）試験薬の用法・用量、投与方法又は試験機器の適用方法

（6）Dosage and administration of trial medicine or method of trial device

アンヘドニアに対するポジティブ価システムに焦点を当てた認知行動療法：

対面での個人療法の形式をとった、ポジティブ価システムを強化する認知行動療法を行う。毎週 60 分、計 8 回のセッションを、国立精神・神経医療研究センターの音響装置が設定された部屋にて行う。60 分のセッションを毎週、計 8 回、個人療法として、訓練を受けた臨床心理士や医師が実施する。治療内容としては、初期のセッションでは、ポジティブ価システムが大切な理由を患者が理解できるように心理教育を行う。その上で、ポジティブな感覚や感情を５感で味わうための訓練や、ポジティブな出来事に注意を向けるためのモニタリングを開始する。さらには、ポジティブな感覚や感情を感じることを妨害する認知を同定し、代わりとなる認知を検討する。このようにして、日々の生活体験の中でポジティブな体験を重ねるとともに、本人にとってのポジティブな側面（強みや趣味）を同定し、それを意識したエクササイズを行う。このようにして、短期的なポジティブ体験（短期的報酬）への感受性と高めた上で、長期的な目標に向けた行動に従事するモジュールにとり組む。アンヘドニアの状態では、長期報酬への割引効果（※時間や労力のかかる目標は価値を置かれにくく行動が生起にくくなる）について心理教育を行った上で、本人にとっての長期的目標や価値を同定する。その上で、本人の長期目標のために取りうる行動の段階に分けて検討し、日々の生活において実施しやすい行動から取り組む。さらに、人間関係におけるポジティブ体験を強化するために、日々の対人的に感謝できる出来事を記録するとともに、コンプリメントを与え受けるという課題に取り組む。最終セッションでは、介入を通して学習した取り組みを継続するための方法を確認し、終結となる。

Positive Valence System-focused Cognitive-Behavioral Therapy for Anhedonia

The PoCot will be conducted in a face-to-face, individual format. Sixty-minute weekly sessions will be conducted in a room with sound equipment in the National Center of Neurology and Psychiatry. A trained clinical psychologist or physician will administer the PoCot. The earlier PoCot sessions will include psychoeducation emphasizing the importance of the positive valence system to recover from depression. The participant will

then begin to practice savoring positive sensations and emotions using the five senses, and in monitoring the enhancement of attention to positive events in daily life. Participants will be requested to identify thoughts that interrupt the mindful acceptance of positive sensations and emotions, and to try to examine alternative thoughts. These practices are intended to promote accumulation of positive experiences in daily life. In parallel, the participant is gradually encouraged to identify and exercise their own positive aspects (e.g., strength and avocation). The focus of this program gradually shifts from enhancing the acceptability of short-term positive experiences (short-term reward) to engaging longer-term, goal-directed behavior. Psychoeducation on temporal discounting of reward in anhedonia will be provided. The participant will be asked to identify longer-term goals or values in their life. The goal will be divided into smaller steps and patients encouraged to engage in easier behaviors. Furthermore, patients will be asked to monitor events for which they are grateful in daily life, and to practice receiving and providing compliments to enhance positive interpersonal experiences. In the final session, participants will review the skills learned in the program and examine the ways of maintaining such activities.

超高周波音響とプラセボ音響呈示のための音源と音響機器：

超高周波音響は、人の可聴域上限をこえる超高周波成分を豊富に含む熱帯雨林の自然環境音を用いる。プラセボ音響は、超高周波音響と同一の音源から高周波成分のみを取り除いた音響を用いる。音響提示装置には研究参加者自身では操作できない安全装置（操作パネルカバーなど）を装備し、装置の制御は実験スタッフが行う。音響情報の種類については、これまでの健常人を対象とした一連の実験室実験や、市街地で多数の健常者を対象とした実験、外来うつ病患者を対象とした実験などで使用実績があり、問題が生じていない音源を、本研究の目的のために特別に編集して用いる。音響情報の呈示音量は、これまでの実験同様、室内の会話の支障にならない低音量とし、その最適音量を検討する。

Sound material and sound presentation system for inaudible high-frequency sound and placebo sound:

Rain forest sounds, a natural sound source containing the richest amount of high frequencies with a conspicuously fluctuating structure, were chosen as the sound source for the experiments. As placebo sound, only the inaudible HFCs were excluded from the original sound material. The sound equipment system will be covered so as not to be able to operate by participant. Experimental staff will regulate the sound presentation equipment. The contents of sound has been used previous study in city environment for healthy population and in experiments with individual with depression without any problem. Sound volume will be small enough to be able to have a conversation in the therapy room.

用量・スケジュール変更規準

Criteria for changes in dose or frequency

本研究では短期の介入を取るために､全８セッションへの参加を被験者に求める。止むを得ずセッションのキャンセルが生じた場合には､遅滞なく日程の再調整を行う。

As short-term intervention will be employed, all participants will be requested to participate in every eight session. If participants have to cancel a scheduled session, an alternative date will be scheduled immediately.

（7）併用薬・併用療法についての規定

（7）Regulation for concurrent drugs or treatments

向精神薬の併用がある場合には、介入期間中には薬剤の種類と用量を一定にするよう、本人及び主治医の同意を予め得ることとする。他に、電気けいれん療法と構造化された精神療法は研究期間には実施しないよう依頼する。

If the participant takes any type of psychotropic medicine, we will obtain consent from the participant and their primary doctors to keep the dose and types of medicine consistent throughout the study period. In addition, we will request that participants refrain from receiving electroconvulsive therapy and/or other systematic psychotherapy during the study period.

（8）評価項目、評価方法

（8）Outcomes and assessment method

主要評価項目は、１週から 12 週にかけて毎週 Snaith–Hamilton Pleasure Scale 日本語版（SHAPS）で測定されるアンヘドニア症状である。

副次評価項目は、12 週に測定される Snaith–Hamilton Pleasure Scale-clinician administered(SHAPS-C)で測定されるアンヘドニア症状である。

The primary outcome is anhedonia symptoms assessed by the Snaith-Hamilton Pleasure Scale from week 1 to 12.

The secondary outcome is anhedonia symptoms assessed by the Snaith-Hamilton Pleasure Scale-clinician administered at week 12.

（14） 観察及び検査項目

(9) Observation or examination items

観察・検査・調査項目

Observation, examination, or survey items

12. 基礎情報：性別、生年月日、婚姻状況、年収、就学・就労状況、病名（主治医による診断）、主訴、登録以前までの治療歴（心理相談含む）、精神科既往歴、教育歴、家族歴、飲酒・喫煙
13. 宿題遵守：治療の一環として宿題の実施遵守を評価する。
14. 併用治療の遵守状況：Visit 毎に患者に確認するとともに、可能な場合はカルテ記載を随時確認することにより、薬物療法の安定性を確認する。
15. アンヘドニア症状：SHAPS 日本語版、SHAPS-C
16. うつ症状の重症度：GRID-HAMD, BDI-II
17. 精神疾患の有無：MINI（除外基準、併存疾患の確認）
18. 他のアウトカム指標：PANAS, SWLS, PWB
19. 治療メカニズム：EROS, EEfRT
20. 有害事象の確認：Visit 毎に口頭および所定の用紙にて確認する。
21. 治療プロセス：HC
22. 盲検化：IEKNO

12. Basic information: Gender, date of birth, marital status, income, current education and/or employment, diagnosis by primary doctor, chief complaints, treatment history, psychiatric history, educational history, family history, alcohol use, smoking.
13. Homework compliance: Homework compliance will be assessed as part of treatment.
14. Compliance for concurrent treatment: Consistency of pharmacotherapy will be checked at every visit. Where possible, the research coordinator will check it via electronic medical charts.
15. Anhedonia: Japanese version of SHAPS, SHAPS-C
16. Depression: GRID-HAMD, BDI-II
17. Presence or absence of mental disorders: MINI (confirmation of exclusion criteria and concurrent disorders)
18. Other outcomes: PANAS, SWLS, PWB
19. Treatment mechanism: EROS, EEfRT
20. Adverse events: Will be checked at every visit by oral questioning and a self-report sheet.
21. Treatment process: HC
22. Masking: IEKNO

観察・検査・調査スケジュール

Schedule for observation, examination, or survey

					ENROLMENT			INTERVENTION								POST	FU
TIME POINT (Week)					-2	-1	0	1–11								12	21
Visit					V1	V2	V3	V4	V5	V6	V7	V8	V9	V10	V11	V12	V13
(Burden for patient (minutes))																	
ENROLLMENT :																	
	Informed Concent			(50)	X												
	Randomization			(5)			X										
	Intake			(60)			X										
ASSESSMENTS :																	
Diagnosis		MINI	IE	(25)		X											
Primary Outcome	Anhedonia	SHAPS	Pt	(4)		X		X	X	X	X	X	X	X	X	X	X
Secondary Outcome	Anhedonia	SHAPS-C	IE	(10)		X										X	X
Other Outcome	Depression/Anhedonia	GRID-HAMD	IE	(30)		X										X	X
		BDI-II	Pt	(5)			X					X				X	X
	Emotion	PANAS	Pt	(3)			X	X	X	X	X	X	X	X	X	X	X
	Life Satisfaction	SWLS	Pt	(2)			X					X				X	X
	Well-being	PWB	Pt	(6)			X					X				X	X
Treatment Mechanism	Anhedonia	EROS	Pt	(3)			X					X				X	X
		EEfRT	Pt	(20)		X										X	X
Safety	Adverse Event	AE	Th	-				X	X	X	X	X	X	X	X	X	X
Adherence	Homework Compliance	HC	Th	-					X	X	X	X	X	X	X		
Blinding		IEKNO	Pt/Th/IE	(2)				X								X	X
Burden for Participants(minutes)					(50)	(89)	(84)	(9)	(7)	(7)	(7)	(23)	(7)	(7)	(7)	(83)	(83)

Fig 2. Overall description of the measures and its timepoints.

IE: Interview with Independent Evaluator, Pt: Participant self-report, Th: Therapist self-report

上記の項目は調査スケジュール表（Fig.2）に基づき、試験施設にて検査する。調査結果を CRF に記載する。規定された観察日（検査日）のずれの許容範囲は±7 日とする。

The items above will be assessed at the trial site following the Examination Schedule Table. Results of the examination will be filled in CRF. An acceptable range of dates is one week before and after the scheduled date.

（15）　実施する検査について

(10)　Assessment

4. 精神疾患の診断（Mini International Neuropsychiatric Interview 7.0.0）：

   MINI は、DSM-5 の主要な精神疾患や臨床状態を診断するための簡易構造化面接法である(Sheehan et al., 1998)。本研究では、DSM-5 診断を評価するために最新版の MINI 7.0.0 を使用する。評価項目は、抑うつエピソード、うつ病/大うつ病性障害、自殺念慮、自傷及び自殺行動、自殺行動障害、躁病エピソード、軽躁病エピソード、双極 I 型障害、双極 II 型障害、双極性障害、特定不能のもの、精神病性の特徴を伴う双極 I 型障害、パニック症/パニック障害、広場恐怖症、社交不安症/社会不安障害（社交恐怖）、強迫症/強迫性障害、心的外傷後ストレス障害、アルコール使用障害、物質使用障害（非アルコール）、精神病性障害、精神病性の特徴を伴う気分障害、神経性やせ症/神経性無食欲症、神経性過食症/神経性大食症、過食性障害、全般性不安症/全般性不安障害、医学的、器質的および薬物関連の病因を除外、反社会性パーソナリティ障害であり、各診断基準について「はい」「いいえ」で回答する。短時間で施行可能である（18.7±11.6 分、中央値 15 分）。旧版については、日本版についても信頼性と妥当性が確認されている(Otsubo et al., 2005)。妥当性については、Structured Clinical Interview for Diagnostic and Statistical Manual-III-R-patient version との基準関連妥当性が示されており（Kappa > .49）、信頼性については高い評者間一致度が報告されている（Kappa > .72）。

12. Diagnostic status of mental disorders (Mini International Neuropsychiatric Interview 7.0.0):

    The MINI is a brief structured interview for assessing major mental disorders and clinical status (Sheehan et al., 1998). We will use the updated 7.0.0 version. The MINI assesses the presence or absence of major depressive episodes, major depressive disorder, suicidality, suicide behavior disorder, manic episode, hypomanic episode, bipolar I disorder, bipolar II disorder, other specified bipolar and related disorder, panic disorder, agoraphobia, social anxiety disorder, obsessive-compulsive disorder, posttraumatic stress disorder, alcohol use disorder, substance use disorder (non-alcohol), any psychotic disorder, major depressive disorder with psychotic features, bipolar I disorder with psychotic features, anorexia nervosa, bulimia nervosa, binge-eating disorder, generalized anxiety disorder, and antisocial personality disorder. Medical, organic, or drug causes will be ruled out. Each diagnostic item is answered by YES/NO. Earlier versions of MINI have been demonstrated to be administrated in a short

period of time (mean 18.7 ± minutes, median 15 minutes). The reliability and validity of the Japanese translation of the earlier version of MINI has been demonstrated (Otsubo et al., 2005). In terms of validity, criterion-related validity with Structured Clinical Interview for Diagnostic and Statistical Manual-III-R-patient version has been reported (Kappa > .49). In terms of reliability, sufficient inter-rater concordance has been reported (Kappa > .72).

13.　アンヘドニア（Snaith–Hamilton Pleasure Scale; SHAPS, Snaith–Hamilton Pleasure Scale-clinician administered; SHAPS-C）：

SHAPS は、アンヘドニアの有無およびその重症度を測定するための自己記入式尺度である（Nagayama et al., 2012; Snaith et al., 1995）。14 項目から成り、1 から 4 の 4 件法で回答するが、評定肢は項目によって異なる（たとえば、項目 1「好きなテレビ番組やラジオ番組を楽しめますか？」に対する評定肢は「1. 少しも楽しくない」「2. 楽しくない」「3. 楽しい」「4. とても楽しい」）。得点が高いほど、アンヘドニアの重症度が高いことを示す。SHAPS 得点の合計が 20 点以上の場合、アンヘドニア症状を有するとみなされる（e.g. ClinicalTrials.gov; 臨床試験 NCT02874534, NCT02494050）。日本語版の信頼性に関しては、十分な値（α=.90）が報告されている（Nagayama et al., 2012）。回答時間 4 分程度。

SHAPS-C は、SHAPS に基づいて教示や評定肢を改良した、他者評価式尺度である（Ameli et al., 2014）。SHAPS と同様に、SHAPS-C は 14 項目から成り、１から４の 4 件法で回答するが、評定肢は全項目で共通である（１＝多くの喜びを感じる、２＝平均的／いつもと同じくらい喜びを感じる、３＝いくらか喜びを感じる、４＝喜びをまったく感じない）。得点が高いほど、アンヘドニアの重症度が高いことを示す。SHAPS-C 英語版の信頼性（Cronbach's α＝.90）および妥当性（e.g. SHAPS との併存的妥当性; r=.85, p<.001）は十分であることが示されているが（Ameli et al., 2014）、SHAPS-C 日本語版は開発されていない。施行時間は 10 分程度。

5.　Anhedonia（Snaith–Hamilton Pleasure Scale, SHAPS; Snaith–Hamilton Pleasure Scale-clinician administered, SHAPS-C）：

The SHAPS is a self-report questionnaire for assessing the presence/absence of anhedonia and its severity (Nagayama et al., 2012; Snaith et al., 1995). It consists of 14 items with 4-point Likert anchors. Each item is answered by different anchors. For example, Item 1 “I would enjoy my favourite television or radio programme” is answered as follows: strongly disagree, disagree, agree, or strongly agree. A higher score means more severe anhedonia symptoms. A total score higher than 20 is interpreted as the presence of anhedonia (e.g. ClinicalTrials.gov: NCT02874534, NCT02494050). Reliability of the Japanese version of SHAPS has been demonstrated (alpha = .90; Nagayama et al., 2012). The average time to answer is 4 minutes.

SHAPS-C is an interview method that improves the instruction and anchors based on the SHAPS (Ameli et al., 2014). Similar to SHAPS, SHAPS-C consists of 14 items with a 4-point Likert scale. The anchor is the same for all items (1 = lots of pleasure, 2 = average/usual pleasure, 3 = some pleasure, 4 = no pleasure). A higher score indicates more severe anhedonia. The original version of SHAPS-C has been reported to have sufficient reliability (alpha = .90) and validity (concurrent validity with SHAPS; *r* = .85, p<.001) (Ameli et al., 2014). We translated the English version of SHAPS-C into Japanese via a rigorous back-translation procedure. It takes approximately 10 minutes to complete the interview.

6. 抑うつ症状（GRID Hamilton Depression Rating Scale 17 Item; GRID-HAMD）：
ハミルトンうつ病評価尺度（Hamilton Depression Rating Scale; HAMD）は、抑うつ症状の重症度を測定するための評価尺度である（Hamilton, 1960）。本研究では、その改良版である GRID-HAMD17 項目版を用いる。GRID-HAMD は半構造化面接の形式をとり、評価者によって実施される。評価対象となる期間は、過去 1 週間である。評価項目は、抑うつ症状に関する 17 項目から成る。各項目の評点は、程度（5 段階評定）と頻度（４段階評定）をそれぞれ評価した上で、縦軸に程度、横軸に頻度をとったグリッド上に表現される基準に従って決定される（たとえば、項目 1「抑うつ気分」では、程度が「軽度」で頻度が「ほとんど常に」であれば 2 点という評点が与えられる）。合計得点は 17 項目すべての評点を合算して算出され、0-52 点の範囲を取る。高ければ高いほど、抑うつ症状が重症であることを示す。本尺度の信頼性に関しては、日本語版に関して優れた評定者間一致度（intraclass correlation coefficient; ICC = 0.95-0.99: Tabuse et al., 2007）が報告されている。また、内的一貫性に関しては原版において十分な値（α = 0.78）が示されている（Williams et al., 2008）。並存的妥当性に関しては、HAMD の別版である Structured Interview Guide for HAMD（SIGH-D）の各項目および合計得点と高い相関が報告されている（Williams et al., 2008）。施行時間 25 分程度。

14. Depression（GRID Hamilton Depression Rating Scale 17 Item, GRID-HAMD）：
Hamilton Depression Rating Scale (HAMD) is an interview for assessing the severity of depression (Hamilton, 1960). We will use the improved version of the original HAMD, which is called GRID-HAMD 17 item version. GRID-HAMD is conducted by the interviewer using a semi-structured format. The assessed period is during the week before the date of assessment. Each item is assessed in terms of severity (5-point Likert, vertical axis) and frequency (4-point Likert, horizontal axis). For example, item 1 “depressive mood” could be rated as 2 if the severity is “mild” and frequency is “almost always”. The total score ranges from 0 to 52. A higher score means more severe depression. In terms of reliability of the Japanese version, high inter-rater concordance has been reported (intraclass correlation coefficient; ICC = .95–.99, Tabuse et al., 2007). Sufficient internal consistency has been reported (alpha = .78)

(Williams et al., 2008). In terms of concurrent validity, high correlations between GRID-HAMD and Structured Interview Guide for HAMD（SIGH-D）have been reported (Williams et al., 2008). It takes approximately 25 minutes to complete this interview.

15. 抑うつ症状（Beck Depression Inventory-II; BDI-II）：

BDI-II は、過去 2 週間の抑うつ症状を測定するための自己記入式尺度である（Beck, Steer, & Brown, 1996; 小嶋・古川, 2003）。21 項目から成る。各項目は、0 から 3 の 4 件法で回答されるが、評定肢は項目によって異なる。得点範囲は 0–63 点であり、得点が高いほど抑うつ症状が重症であることを示す。日本語版の信頼性に関しては、一般青年・成人を対象とした研究で十分な値（α=.87）が報告されている（Kojima, Furukawa, Takahashi, Kawai, Nagaya, & Tokudome, 2002）。また、日本語版の並存的妥当性に関しては、CES-D と有意な中程度の正の相関（r = .69）が報告されている（Kojima et al, 2002）。回答時間 5 分程度。

4. Depression（Beck Depression Inventory-II; BDI-II）:

BDI-II is a self-report scale for assessing depression over the past two weeks (Beck et al., 1996; Kojima & Furukawa, 2003). It consists of 21 items with a 4-point Likert scale (0–3). The anchor varies from item to item. The score range is 0 to 63. A higher score indicates more severe depression. In terms of reliability of the Japanese version, sufficient internal consistency has been reported (alpha =.87) (Kojima et al., 2002). As for concurrent validity, the Japanese version of BDI-II was moderately correlated with CES-D (r = .69) (Kojima et al., 2002). It takes approximately 5 minutes to complete the scale.

16. ポジティブ・ネガティブ感情（The Positive and Negative Affect Schedule; PANAS）：

PANAS は、日々の感情体験を測定するための自己記入式尺度である（Clark & Watson, 1988; Clark & Watson, 1989）。22 項目から成る。各項目は、「1. ほとんど、または全くあてはまらない」から「5. 非常にあてはまる」の 5 件法で回答される。ネガティブな感情とポジティブな感情についての 2 つの下位尺度がある。各下位尺度は 11 項目で構成され、11-55 点という得点範囲をとる。得点が高いほど、ネガティブな感情またはポジティブな感情をより多く体験していることを示す。PANAS は様々なタイムフレームで用いられることがあるが、本研究では「過去 1 週間」というタイムフレームを採用する。回答時間 5 分程度。

5. Positive and Negative emotion (The Positive and Negative Affect Schedule, PANAS):

PANAS is a self-report measure for assessing daily emotional experiences (Clark & Watson, 1988; Clark & Watson, 1989). It consists of 22 items. Each item is rated on a 5-point Likert scale from 0 (very slightly or not at all) to 5 (extremely). There are two sub-scales: positive affect and negative affect. Each subscale consists of 11 items. The score range for the subscales

is 11 to 55. A higher score means more frequent experience of positive and negative affect. PANAS can be used at various time frames. We use the time frame of “past one week.” It takes approximately 5 minutes to answer this scale.

17. 人生満足度（Satisfaction With Life Scale; SWLS）：

SWLS は、人生に対する満足の程度を測定するための自己記入式尺度である（Diener et al., 1985; 角野、1994）。5 項目から成る。各項目は、「1. 全くそうではない」から「7. 全くそうだ」の 7 件法で回答される。得点が高いほど、人生に対する満足度が高いことを示す。日本語版の信頼性に関しては、十分な値（α= .84〜.90）が報告されている（角野, 1994）。また、日本語版の妥当性に関しては、自尊心尺度との間に有意な中程度の正の相関（r = .59）および Y-G 性格検査の D 尺度（抑うつ性）と N 尺度（神経質）との間に有意な中程度の負の相関（D 尺度: r = -.50; N 尺度: r = -.34）が報告されている（角野, 1994）。回答時間 1 分程度。

6. Satisfaction with life（Satisfaction With Life Scale, SWLS）：

SWLS is a self-report measure for assessing the degree of life satisfaction (Diener et al., 1985; Sumino, 1994). It consists of five items. Each item is answered using a 7-point Likert scale from “1. Strongly disagree” to “7. Strongly agree.” A higher score indicates higher satisfaction with life. The Japanese version is reported to have high internal consistency (alpha = .84–.90) (Sumino, 1994). In terms of the validity of the Japanese version, a moderate positive correlation with self-esteem (r = .59) and a moderate negative correlation with the Depression and Neuroticism scale in the Y-G personality inventory has been reported (r = -.50, -.34) (Sumino, 1994). It takes approximately 1 minute to answer this scale.

18. 心理的 well-being（Psychological Well-being Inventory; PWB）：

PWB は､心理的 well-being を測定するための自己記入式尺度である（Kitamura, Kishida, Katayama, Matsuoka, Miura, & Yamabe, 2004; Ryff, 1989）。人格的成長，人生における目的，自律性，環境制御力，自己受容，積極的な他者関係の 6 因子、計 18 項目で構成されている。各項目は「1. 全く当てはまらない」から「6. 大変当てはまる」の 6 件法で回答される。人格的成長は，発達と可能性の連続上にいて，新しい経験に向けて開かれている感覚を表す。人生における目的は，人生における目的と方向性の感覚を表す。自律性は，自己決定し，独立，内的に行動を調整できるという感覚を表す。自己受容は，自己に対する積極的な感覚を表す。環境制御力は，複雑な周囲の環境を統制できる有能さの感覚を表す。積極的な対人関係は，温かく，信頼できる他者関係を築いているという感覚を表す。回答時間 4 分程度。

9. Psychological well-being（Psychological Well-being Inventory, PWB）：

PWB is a self-report measure for assessing psychological well-being (Kitamura et al., 2004; Ryff, 1989). This 18-item scale has six subscales: purpose in life, autonomy, environmental mastery, personal growth, positive relations with others, and self-acceptance. Each item is rated using a 6-point Likert scale from “1. Completely disagree” to “6. Completely agree.” The definition of each sub-scale is described by Ryff (2014). Purpose in life is the feeling of meaning, purpose, and direction in one’s life. Autonomy is the feeling that one’s life is in accordance with one’s own personal convictions. Personal growth is the feeling that one is making use of one’s own personal talents and potential. Environmental mastery is the feeling of how well one manages life situations. Self-acceptance is the knowledge and acceptance one has of oneself, including awareness of personal limitations. Positive relations with others is a feeling of depth of connections one has in ties with significant others. It takes approximately 4 minutes to answer.

10. 環境内の報酬知覚（Environmental Reward Observation Scale; EROS）：

EROS は、行動に随伴する正の強化について主観的に評価する尺度である。10 項目からなり、4 件法（1-4 点）で回答する。得点範囲は 10-40 点であり、得点が高いほど、主観的に多くの報酬を知覚していることを表す。様々な population において信頼性および妥当性が報告されている（Armento & Hopko, 2007; 国里・高垣・岡島・中島・石川・金井・岡本・坂野・山脇、2011）。日本人サンプルを対象としたデータにおいては、Cronbach’s α=.78、再検査信頼性は r =.75 と高い信頼性が報告されている（国里他、2011）。また、妥当性に関しては、抑うつ・不安症状（BDI-II, CES-D, STAI）との中程度から強い負の相関（順に r = -.55, -.57, -.65）が報告されている（国里他、2011）。回答時間 2 分程度。

19. Environmental Reward Observation (Environmental Reward Observation Scale, EROS):

EROS is a self-report measure for assessing the subjective feeling of positive reinforcement contingent with behavior. In total, 10 items are answered using 4-point Likert anchors (1 to 4). The score ranges from 10 to 40. A higher score means a more subjective observation of reward. Its reliability and validity has been reported in multiple populations (Armento & Hopko, 2007; Kunisato et al., 2011). High reliability has been reported in the results of the Japanese population (alpha = .78, test-retest correlation = .75) (Kunisato et al., 2011). In terms of validity, EROS was moderately to strongly correlated with depression and anxiety (r = -.55, -.57, -.65 with BDI-II, CES-D, STAI) (Kunisato et al., 2011). It takes approximately 2 minutes to answer this scale.

20. 報酬の行動評価(Effort-Expenditure for Rewards Task; EEfRT)：

EEfRT は、報酬の獲得可能性に関する予測的価値およびエフォートに基づいた意思決定の程度を測定するための行動課題であり、コンピュータを用いて実施される（Treadway, Buckholtz, Schwartzman, Lambert, & Zald, 2009）。主な手順は以下の通りである；（１）報酬獲得のための課題として「易しい課題」と「難しい課題」を選択する画面が 5 秒間呈示される。（２）「易しい課題」では、7 秒以内に 30 回のボタン押し（利き手使用）が求められる。（３）「難しい課題」では、21 秒以内に 100 回のボタン押し（非利き手使用）が求められる。（４）それぞれの課題を達成すると決められた報酬を得ることができる。具体的には、「易しい課題」では一律 1 ドル、「難しい課題」では 1.24 ドルから 4.30 ドルの幅のある報酬が設定される。つまり、低コスト/低報酬条件、高コスト/低報酬条件、高コスト/高報酬条件の 3 条件が設定される。ただし、研究参加者は毎回の試行で報酬を必ず得られるわけではなく、3 種類の確率（12%, 50%, 88%）のいずれかで報酬を得ることができる。（５）研究参加者は、各試行終了後に報酬を得られるかどうかのフィードバックを受ける。（６）研究参加者は、これまでの一連の行程を 1 試行とし、20 分間の制限時間の間にできだけ多く行う。施行時間 20 分程度。

9. Behavioral assessment for reward (Effort-Expenditure for Rewards Task, EEfRT)：

EEfRT is a computerized behavioral task for assessing reward expectancy and effort-based decision making (Treadway et al., 2009). The procedure of this task is as follows: 1) 5-second display for selecting an easy or difficult task to obtain the reward, 2) easy task requires participants to push a key 30 times in 7 seconds using their dominant hand, 3) difficult task requires participants to push a key 100 time in 21 seconds using their non-dominant hand, 4) participants are eligible for a reward if they succeed in the task. The reward for the easy task is one dollar. The reward for the difficult task ranges from 1.24 to 4.30 dollars. As such, there are three conditions: low effort and low reward, high effort and low reward, and high effort and high reward. However, participants may not obtain a reward in every trial. The probability of receiving a reward is set at 12%, 50%, or 88%, 5) participants who succeed in the trial notice whether they get the reward immediately after each trial, 6) participants continue trials for 20 minutes. It takes 20 minutes to complete this task.

21. 宿題遵守（Homework Compliance Scale; HCS）：

HCS は、患者が宿題をどの程度遂行したかについて治療者が評価する尺度である（Primakoff, Epstein, & Covi, 1986）。1 項目からなり、毎セッションの終了時に 0-6 点で評価する。筆者らは、原著者の許可および協力を得て、バックトランスレーションのプロセスを経て日本語に翻訳した。

10. Homework compliance（Homework Compliance Scale, HCS）：

HCS is a therapist rating scale for assessing the degree of homework completion (Primakoff et

al., 1986) It consists of one item. Therapist rates 0 to 6 points after each session. We translated this scale into Japanese via rigorous back-translation procedures with permission from the author of original version.

22. マスキングの評価（Independent Evaluator Knowledge of Outcome; IEKNO）：

本尺度は、パニック障害に対する大規模な臨床研究(Barlow, Gorman, Shear, & Woods, 2000)や、統一プロトコルの RCT（Farchione et al., 2012）において利用された尺度である(Roll et al., 2004)。3 項目からなり、患者がどちらの群に割り付けられたと考えるか、それについてどの程度の自信を持っているか 0 から 8 の Likert で回答を求める。そして、最後の項目では、割付を見抜いたと考えられる場合には、どのような情報からそう考えられるかを尋ねる。本研究では、Blinding Index(Bang et al., 2004)を算出するために、割付の推測について“介入群”、“対照群”、“わからない”の 3 つから選択する回答方式とする。

11. Masking (Independent Evaluator Knowledge of Outcome, IEKNO):

This scale has been used in a large scale clinical trial for panic disorder (Barlow, Gorman, Shear, & Woods, 2000) and randomized controlled trial for unified protocol （Farchione et al., 2012). It consists of three items: judgement of allocated groups, confidence, and source of the judgement. In this study, we used the anchors “intervention group”, “comparison group”, and “I don’t know” to calculate the blinding index (Bang et al., 2004).

（16） 症例登録、割付の方法

(11) Method of registration and randomization

症例登録

Registration of participants

割付は、インフォームド・コンセントを経て評価面接により全ての選択・除外基準を確認した上で、研究スタッフとは独立した専用の担当者が、NCNP 開発の EDC システムによりオンライン上で行う（中央登録による割付の隠蔵）。順番の作成はコンピュータにより行われる。

After obtaining informed consent and confirming eligibility by an assessment interview, research staff specifically for this role will register the participant using EDC system developed by NCNP. Sequences will be generated by this EDC system.

割付方法と割付調整因子

Method of randomization and stratification

ブロックランダム化を行い、調整因子は設定しない。

We will use block randomization without any stratification.

割付表の保管と開錠手続き

Storage of randomization table and procedure for unblinding

割付表は割付担当者が保管する。重篤な有害事象が発生するなど、研究責任者が必要と認めた場合には、研究責任者が担当者に開錠を依頼する。

Personnel for allocation will store the randomization table. Whenever the primary investigator acknowledges the need for unblinding such as in the occurrence of a severe adverse event, the primary investigator will request the personnel for anonymization to open the key.

（17） 統計解析方法

(12) Statistical analysis

解析の概要

Summary of statistical analysis plan

データマネジャーがデータの固定と解析を行う。全ての主要・副次評価項目の解析において、介入群と対照群を比較する。全ての解析で、P 値が小数点 3 までの値で表現され、0.001 以下のものは $p < .001$ と記載する。解析ソフトは、SPSS、SAS、R を用いる予定である。統計的検定を用いる解析では、両側検定として有意水準を 5%とする。本研究は主要評価項目の解析のためにデザインされているため、副次評価およびその他の解析（サブグループ解析・調整解析）は探索的なものとなる。

The data manager will fix and analyze the data. We will compare the intervention and comparison groups in primary and secondary analyses. The *p*-value will be expressed to three decimal places. If the *p*-value is under .001, it will be described as $p < .001$. We will use statistical software SPSS, SAS, and/or R. When we apply statistical tests, we will set statistical significance for 5% of a two-tailed test. As this study is designed to test the primary outcome, analyses for secondary and other outcomes, as well as for other purposes (sub-group analysis, adjusted analysis, etc.) will be exploratory.

主要評価項目の解析

Analysis of primary outcome

評価項目：1 週から 12 週にかけての SHAPS 日本語版得点

従属変数を 1–11 週までの 8 ビジット時点、および 12 週時点の評価項目とし、独立変数の固定効果要因を割付（介入群=0 vs 対照群=1）と測定時点 (visit 1=1, visit 2=2, visit 3=3, visit 4=4, visit 5=5, visit 6=6, visit 7=7, visit 8=8, week 12=9)、両要因の交互作用項(割付け*測定時点)、および評価項目の pre 値（-1 週）とし、変量効果要因を反復測定した被験者として、線形混合モデルにより検討する。

Outcome: Score of the Japanese version of SHAPS assessed nine times from 1 to 12 weeks.

Liner mixed model will be conducted to analyze the primary outcome. The dependent variable is participants' SHAPS scores assessed at eight visit points from week 1 to 11 and week 12. Fixed-effects are allocation (intervention group = 0 vs. comparison group = 1), visit (visit 1=1, visit 2=2, visit 3=3, visit 4=4, visit 5=5, visit 6=6, visit 7=7, visit 8=8, week 12=9), and allocation-by-visit interaction, and SHAPS at pre-intervention (week -1). The random variable will be participant.

副次評価項目の解析

Analysis of secondary outcome

評価項目：12 週時点の SHAPS-C

ベースラインを共変量とした共分散分析を行う。

Outcome: Score of SHAPS-C at week 12

As with the primary analysis, linear mixed model will be conducted. In the model, the time point will be pre and post only.

他の評価項目の解析

Analysis of other outcomes

評価項目：12 週時点の BDI-II, PANAS, SWLS, PWB, EROS, EEfRT

主要評価項目と同様に、線形混合モデルによる検討する。ただし測定時点は、pre, week 5, post となる。

Outcome: BDI-II, PANAS, SWLS, PWB, EROS, EEfRT at weeks 5 and 12

LMM analysis will be conducted as same as primary analysis. In the model, the time point will be pre, week 5, and post.

欠測値の処理

Handling of missing data and sensitivity analysis

混合モデルによって解析することで欠損値に対処する。さらに、"best"または"worst"のそれぞれのケースシナリオを仮定した感度分析を実施する。

We use mixed model to treat missing data. Furthermore, we will conduct sensitivity analyses by assuming both "best" and "worst" case scenarios.

上記に定めていない症例やデータの取り扱いは、研究責任者と解析責任者で協議、決定する。

Other statistical analyses or data handling will be discussed and judged by the primary investigator and study statistician.

解析対象集団

Analysis populations

主要・副次評価項目についての解析はすべて、Intent-To-Treat 原則に基づき登録されて全参加者を対象として実施する。副次的な解析対象集団として、研究登録された後に中止症例に該当しなかった全例を当研究計画書に適合した対象集団（Per Protocol Set, PPS=Completer case）として、評価項目についての解析を行う。

Analyses for the primary and secondary outcome analysis will be conducted on Intent-To-Treat principle, which all registered participant are subject to analysis. As the secondary analysis set, we will conduct the outcome analysis by using the all registered participant who will not meet the discontinuing criteria as Per Protocol Set.

（18）　試料・情報の授受

(13) Provision or receipt of specimens or information

本研究は単施設で実施するため、外部との情報の授受はない。

We will not exchange any specimens or information as this is a single site study.

6．研究対象者の選定方針

6. Enrolment criteria of research participants

（1）選択基準

（1）Inclusion criteria

4）　アンヘドニア症状を有すること（SHAPS 日本語版得点の合計が 20 点以上）

5）　うつ症状が軽症（GRID-HAMD の 8 点）以上

6）　18 歳以上

(1) Anhedonia symptoms (Snaith-Hamilton Pleasure Scale score >= 20)

(2) Depressive symptoms are mild or severe (GRID Hamilton depression rating scale >= 8)

(3) Aged 18 years or older

【各選択基準の設定理由】

1)　有効性評価のため

2)　有効性評価のため

3)　有効性評価のため（研究対象者を SHAPS 日本語版の妥当性が検証されている成人に限定するため）

【Reason for each inclusion criteria】

4) For efficacy evaluation

5) For efficacy evaluation

6) For efficacy evaluation (Japanese version of SHAPS has not been examined for reliability and validity among the younger Japanese population)

（2）除外基準

(2) Exclusion criteria

7） 精神病性障害（現在）（MINI にて評価）

8） 躁病エピソード（現在）（MINI にて評価）

9） 物質使用障害（現在、重度）（MINI にて評価）

10） 重篤な自殺念慮（MINI にて評価）

11） 治療に支障のある身体疾患や認知機能障害

12） その他に、認知行動療法を遂行する上で障害となる問題のある者

7） No current psychotic disorders at baseline assessed by the Mini-International Neuropsychiatric Interview (MINI)

8） No current manic episode at baseline assessed by MINI

9） No severe substance use disorders at baseline assessed by MINI

10） No serious suicidal ideation at baseline assessed by MINI

11） No severe or unstable physical disorders or major cognitive deficits at baseline

12） Other problems that may be serious obstacles for conducting CBT

【各除外基準の設定理由】

1) 有効性評価のため

2) 安全性のため

3) 安全性のため

4) 安全性のため

5) 有効性評価のため

6) 安全性及び有効性評価のため

【Reason for each exclusion criteria】

7) For efficacy evaluation

8) For safety

9) For safety

10) For safety

11) For efficacy evaluation

12) For efficacy evaluation and safety

7. インフォームド・コンセント等を受ける手続等

7. Procedure for obtaining informed consent, etc.

倫理委員会で承認の得られた同意説明文書を研究対象者（代諾者が必要な場合は代諾者を含む、以下同じ）に渡し、文書及び口頭による十分な説明を行い、研究対象者の自由意思による同意を文書で取得する。研究対象者の同意に影響を及ぼす情報が得られたときや、研究対象者の同意に影響を及ぼすような研究計画書等の変更が行われるときは、速やかに研究対象者に情報提供し、研究に参加するか否かについて研究対象者の意思を予め確認するとともに、事前に倫理委員会の承認を得て同意説明文書等の改訂を行い、研究対象者の再同意を得ることとする。本研究では、有効なインフォームドコンセントを与えることができると判断された 18 歳以上の未成年者を対象に加える。それらの者を加える理由は、世界的には、成人として 18 歳以上を対象として研究されることが一般的であるためである。

Research coordinators will present the IRB approved informed consent briefing paper to the participants (including to legally acceptable representatives, if applicable), orally explain the research sufficiently using the briefing paper, and obtain consent that is reflected by the participant’s free will in written document. If any changes occur in the research protocol or the primary investigator acknowledges information that may influence participants’ consent, the primary investigator will immediately provide such information to participants and confirm continued participation in this study. The primary investigator will also revise the informed consent briefing paper and re-obtain participants consent using the revised paper. Subjects in this study are those older than 18 years old with sufficient capacity to provide informed consent. This is because in this type of study around the world, 18 years of age or older is sufficient to be regarded as “adult”; in Japan, 20 years of age or older is considered “adult” `.

21. 同意説明文書の概略、研究により生じる知的財産権の帰属
22. 研究の名称、研究実施について所属機関の長の許可を受けている旨
23. 研究の目的及び意義
24. 研究対象者として選定された理由（研究対象となる方）、研究の方法及び期間、参加協力事項
25. 研究対象者に生じる負担並びに予測されるリスク及び利益
26. 研究が実施又は継続されることに同意した場合であっても随時これを撤回できる旨、研究が実施又は継続されることに同意しないこと又は同意を撤回することによって研究対象者等が不利益な取扱いを受けない旨
27. 研究に関する情報公開の方法
28. 研究対象者等の求めに応じて、他の研究対象者等の個人情報等の保護及び当該研究の独創性の確保に支障がない範囲内で研究計画書及び研究の方法に関する資料を入手又は閲覧できる旨並びにその入手又は閲覧の方法

29. 個人情報等の取り扱い（匿名化の方法を含む）、情報の保管
30. 情報の廃棄の方法、研究終了後のデータ取扱の方針
31. 研究の資金源、利益相反
32. 研究対象者等及びその関係者からの相談等への対応
33. 経済的負担及び謝礼の内容
34. 研究で用いられる治療方法以外の治療方法の内容
35. 研究終了後の医療の提供の有無とその内容
36. 研究対象者に関わる偶発的所見への対応
37. 健康被害に対する補償の有無及びその内容
38. 研究終了後のデータの二次利用の可能性とその対応方法
39. 研究機関の名称及び研究責任者の氏名
40. 問い合わせ先

21. Outline of the informed consent briefing paper, ownership of intellectual property
22. Title of the research and information on approval given by the chief executive of the research implementing entity concerning its implementation
23. Objectives and significance of the research
24. Reasons for enrolment in the research, method, and time period of the research
25. Potential burdens on research subjects and predictable risks and benefits
26. Condition that research subjects may withdraw their consent at any time even after they have given consent that the research commences or continues. Condition that the refusal or withdrawal of consent by a research subject regarding commencement or continual of the research does not cause any disadvantage to them
27. Means to make information on the research public
28. The fact that research subjects can request and obtain or read the research protocol and documents concerning method of the research, to the extent that it does not interfere with the protection of personal information, etc. of other research subjects or the originality of the research, as well as the procedure to obtain or read such protocols and documents
29. Handling of personal information, etc. (including process of anonymization and when anonymization is conducted), means for storage information
30. Means for disposal of information
31. Status of research-related conflicts of interest of the research implementing entity, such as research fund resources, as well as research-related conflicts of interest of each investigator such as his/her individual income
32. Response to consultation, etc. made by research subjects and other individuals concerned
33. When the research involves any financial expenditure on or remuneration for the research subject; a statement to that effect and details of such

34. When the research involves any medical technique beyond usual medical practice, description of alternative procedure(s) or course(s) of treatment
35. When the research involves any medical technique beyond usual medical practice, response related to the healthcare delivery to the research subjects after the research
36. When any significant finding concerning the subject's health or generic characteristics which may be inherited by his/her offspring, etc. may be obtained through implementing the research, handling of the research results related to the research subject (including incidental findings)
37. When the research involves any invasiveness, whether compensation will be offered for research-related injury and details of such compensation
38. With respect to specimens and information acquired from the research subject, when any of those may be utilized or provided to other research implementing entities for research in the future that is not identified at the time of obtaining consent from the research subject; a statement to that effect and the contents of utilization assumed at the time of obtaining consent;
39. Names of the research implementing entity and the principal investigator
40. Contact information

8. 試料・情報、個人情報等の取扱い（匿名化する場合の方法、個人情報の安全管理方法など）

8. Handling of specimens, information, and personal information (method of anonymization, handling of personal information, etc.);

個人情報管理者（田島美幸）は、同意書など個人情報を含む研究必須文書等の紙媒体や電子媒体、個人情報と研究用 ID の対応表について、漏洩、盗難、紛失しないように以下の通り管理・保存する。

Administrator of the management of personal information (i.e., Miyuki Tajima, Ph.D.) will manage and store all research-related printed or electronic records with personal information (e.g., informed consent documents), correspondence table of personal information and research ID number to prevent any information being divulged, stolen, or lost. Details of the management and storage are as follows.

物理的安全管理

Physical security management

物理的安全管理措置は、あらゆる紙資料や電子データ（同意書、対照表、録音・録画のデータを含む）の取り扱いを想定している。

Physical security management concerns any printed or electronic data including signed informed consent documents, correspondence table, recorded audio or video, etc.

5. 個人データを取り扱う区域の管理：個人データは国立精神・神経医療研究センター内において、患者から直接取得される（病院内での症状評価面接や自己記入式尺度への回答、PC を用いて行われる認知課題のデータ、認知行動療法セッションの録音・録画）。この個人データは、国立精神・神経医療研究センター７号館３階の認知行動療法センター内の居室のキャビネットの中に保管する。個人データを保管し扱う区域は国立精神・神経医療研究センター７号館３階の認知行動療法センター内とする。認知行動療法センターへの入室はカードキーで管理されており、カードキーはセンターに雇用されている者のみが保有する。カードの所持状況は、認知行動療法センター長が帳簿を作り管理する。居室の鍵は、NCNP 全体の鍵管理の方針に従い、使用外の時間は NCNP 防災センターにて保管される。

2. Restriction area for handling the personal information

All personal information will be obtained from participants in the National Center of Neurology and Psychiatry (e.g., answering the psychiatric symptoms interview or self-report scales in the hospital, cognitive task using PC, and recorded audio or video date of the CBT session). These data will be stored in the cabinet in the National Center for Cognitive-Behavior Therapy and Research located on the 3rd floor of 7th building in the National Center of Neurology and Psychiatry. Entry to the National Center for Cognitive-Behavior Therapy and Research is access restricted. Only center staff are permitted entry. The director of the center is responsible for the management of the card key and maintaining the information of the possession of card keys. Keys for each office room will be returned to the disaster prevention center at the end of every day.

6. 機器及び電子媒体等の盗難等の防止：盗難防止のために、上記①の管理に加えて、キャビネットもそれぞれ施錠可能なものを用いる。さらに、キャビネットの鍵は、パスワード認証が必要なキーボックスに保管する。キーボックスは、特殊な方法を用いなければ脱着できないような強度で壁に固定する。

2. Prevention of theft of devices and electronic media

To prevent theft, we will use a lockable cabinet. Furthermore, we will use the key box with password authentication. This key box will be placed on the wall firmly so as not to easily removed.

7. 電子媒体等を持ち運ぶ場合の漏えい等の防止：電子媒体は管理区域内のみで利用する。

3. Prevention of divulgation during electronic media usage

Electronic media will be used in the restricted area

8. 個人データの削除及び機器、電子媒体等の廃棄：匿名化された検査データなどの紙資料は、すべてただちに個人を特定するような情報（固有名詞など）を削除したかたちで保管する。これらの検査データなどと、ただちに個人が特定できる情

報を含む研究データ（同意書、対応表、録音・録画データ）は研究終了後 5 年後に個人情報をわからなくして、復元不可能な形で廃棄する。

4. Disposal of personal information and electronic devices and media

Printed data such as self-report scales will be immediately anonymized by deleting any identifying information (e.g., specific names etc.) and securely stored. Five years after the completion of the study, these anonymized data and other personal identifying data (signed informed consent documents, correspondence table, and recorded audio and video files) will be disposed of to avoid restoration.

技術的安全管理：

Technical Security Management:

技術的安全管理は電子化されたデータ（録音・録画のデータを含む）の安全管理措置を想定しており、下記のような措置をとる。

Technical security management concerns the handling of electronic data including audio and video data.

5. アクセス制御：研究代表者の伊藤正哉、コーディネート担当の横山知加、宮前光宏のみが録音・録画データが保管された HDD にアクセスできる。研究遂行上の必要に応じて、研究代表者が認める者に一時的にアクセスを許可することがある（データ入力やモニタリングなど）が、その場合にはアクセス制御を許可された者の監督下での扱いとする。電子データは強制暗号化とパスワード認証が求められる HDD を用いてデータを保管する。

1. Access restriction: Only the primary investigator (MI) and coordinators (CY, MM) are able to access the HDD where the audio and video data are stored. If necessary, the primary investigator may temporarily allow another person to access the data (e.g., for data entry and monitoring). In that case, the person has to treat the data under the monitoring of MI, CY, or MM. All electronic data will be stored in the encrypted HDD with password protection.

6. アクセス者の識別と認証：上記①に定めた者のみが HDD に保管されたデータにアクセスでき、認証パスワードを知ることができるようにする。

2. Identification and authentication of accessible personnel:

Only MI, CY, and MM can access the HDD and have the password.

7. 外部からの不正アクセス等の防止：録音・録画データの再生においては、インターネットに接続されない端末もしくは機器を用いる。

3. Prevention of unauthorized access: When handling the data such as playing the recorded audio or video files, we will use the devices offline while not connected to the Internet.

8. 情報システムの使用に伴う漏えい等の防止：電子データは外部ネットワークにつながった状態の機器には接続しない。

4. Prevention of divulgation in the process of using electronic information system: We will not connect any electronic data to outside networks.

人的安全管理：

Human security management:

3. 雇用契約及び委託契約の締結時における守秘義務規定：当研究は『平成 22 年規程第４０号保有する個人情報の保護に関する規程』が適用される NCNP 職員もしくは研究生によって実施される。

1. Confidentiality obligation in the employment and consignment contracts: All study staff in this study are subject to the policy for protection of personal information (NCNP policy No. 44, 2010).

4. 研究者等に対する教育・訓練の実施：手順書の徹底を図るよう訓練を実施するとともに、NCNP で実施される『平成 22 年規程第４０号保有する個人情報の保護に関する規程』第８条にて規定されている研修をはじめ、コンプライアンス研修、倫理研修に参加する。

2. Education and training for the researchers:

All study staff are subject to training following the standard procedure operations for this study. They will participate in the workshop described in article 8 in the NCNP policy for protection of personal information (NCNP policy No. 44, 2010) and other workshops for compliance and research ethics.

9. 研究参加のリスクと研究がもたらすベネフィット

9. Risks and benefits of research participation

（1）研究対象者に生じる負担

(1) Burdens on research subjects

本研究によって、研究の実施に伴って確定的に生じる事象としては、全 12 週間にわたる毎週の通院と評価と介入治療にかかる時間と労力の消費であるが、これらがすべからく研究対象者にとって好ましくない事象として知覚・認識されるとは限らない。

Burdens for the participants will be time and effort for the 12-week intervention and symptom evaluation. Perceptions of these burdens will vary between participants.

（2）研究対象者に生じ得る不利益（有害事象不具合等）

(2) Potential disadvantages for research subjects (e.g., adverse events)

本研究によって生じ得る不利益は想定されないが、自身の人生や生活を振り返って、今

までしてきたのとは違う取り組みを行う中で、一時的にネガティブな気分を体験することはあり得る。

We do not expect any disadvantages to participants for participating in this research. However, participants may temporarily experience a negative mood in response to reflecting on their past life and practicing new behaviors.

（3）リスクを最小化する方法

(3) Strategy to minimize risks

健康被害が生じた場合、適切な医療等の対応が行われるように図る。本研究では、研究に伴う健康被害のリスクは、一般の外来診療に伴うもの以上は特にないものと考えられるため、健康被害に対する医療も通常の診療と同様に、被験者の保険診療内で行う。

If any health damage occurs, the primary investigator will arrange an appropriate medical response. As we assume that the risk of health damage is not likely to occur as is usual in outpatient care, we will use participants' medical insurance for the medical expenses of any health damage, as used in usual-care.

（4）予想されるベネフィットと（1）（2）（3）を踏まえた総合評価

(4) Comprehensive assessment of burdens, risks, and benefits

本研究の参加者は、本研究で有効性を検証しようとしている症状を有する方であり、本研究に参加し介入を受けることで、直接的に本人の症状が改善する可能性がある。明確に想定される不利益は、評価と介入に要する労力と時間である。万が一に不利益が生じた場合には、適切な医療等の対応が行われるように図る。これらを総合評価し、本人に利益が生じる可能性があるだけでなく、研究成果により将来の医療の進歩に貢献できる可能性がある。その具体的意義については 1 に記載した通りである。

We developed the intervention and are going to test its efficacy specifically for anhedonia symptoms. We hypothesize that there are potential benefits for participants for symptom improvement by participating in this research. Clear disadvantages for the participants will be the effort and time required for symptom evaluation and treatment. If by any chance other disadvantages should occur, the primary investigator will arrange an appropriate medical response. By comprehensively assessing these aspects, this study has potential benefits for the participants and the study results may promote the progress of future medicine. The medical significance of the study has already been discussed in section 1 of this protocol.

（5）個々の研究対象者における中止基準

（5）Criteria for discontinuing interventions for a given trial participant

研究中止時の対応

Correspondence when the discontinuing occurs

　個々の症例が以下のいずれかの中止基準に該当する場合、その症例の試験治療を中止する。中止の日付・時期(治療期間・追跡調査期間)、中止の理由、経過をカルテならびに CRF に明記するとともに、中止時点で主要評価項目と副次評価項目に関する評価を行い有効性・安全性の評価を行う。有害事象発生により中止した場合は、研究責任者は担当治療者、スーパーバイザー、主治医、NCNP 担当医と状況を検討し、介入中止になった理由に応じて適切な対応を決定し、可能なかぎり原状に回復するまでフォローする。中止後も可能なかぎり 12 週時点での評価を行う。

If a participant meets either of the following discontinuing criteria, the participant will terminate participation. The trial coordinator will record the date and period (intervention or follow-up period) of discontinuation, its reason, and the course in CRF. Whenever possible, participants will be requested to participate in primary and secondary outcome evaluations at the time of discontinuation. If the discontinuation occurred due to an adverse event, the primary investigator will discuss with the therapist, supervisor, primary doctor, and responsive doctor at NCNP about the possible response to the patients, and monitor patients until they recover to the degree of baseline. Whenever possible, the trial coordinator will ask patients to participate in the outcome evaluation at week 12.

中止基準

Criteria for discontinuation of participation in the trial

6） 被験者から試験参加の辞退の申し出や同意の撤回があった場合
7） 被験者との連絡が不通となった場合
8） 重篤な有害事象により試験治療の継続が困難な場合
9） 試験全体が中止された場合
10） その他の理由により、研究責任者が試験を中止することが適当と判断した場合

6） Participant requests discontinuation or withdraws consent
7） Unable to contact the participant
8） Difficulty continuing participation due to severe adverse events
9） Discontinuation of the whole trial
10） Primary investigator’s judgement of discontinuation to be appropriate for any other reason

2） **研究全体の中止基準**

2） **Discontinuation of trial**

4） 倫理委員会により中止の勧告あるいは指示があった場合は、試験を中止する。研究責任者および効果安全性評価委員で構成する委員会は、以下の事項に該当する場合は試験実施継続の可否を検討する。

5） 治療の品質、安全性、有効性に関する重大な情報が得られたとき。

6） 倫理委員会により実施計画等の変更の指示があり、これを受入れることが困難と判断されたとき。試験の中止または中断を決定した時は、速やかに倫理委員会にその理由とともに文書で報告する。試験中止の決定を行った後、関係医師および心理士等に速やかに伝達し、中止後の処理にあたるものとする。

4） If the IRB advises or orders trial discontinuation, this trial will be stopped. The committee consisting of the primary investigator and members of DSMB will examine the appropriateness of continuing the trial when the following occurs.

5） Acknowledgement of important information regarding quality, safety, and efficacy of the intervention.

6） When the IRB orders modification of trial procedures and the primary investigator judges such modification to be difficult. If the primary investigator decides to stop or discontinue the trial, the primary investigator will report the reason immediately to IRB in written form. After deciding to discontinue the trial, the primary investigator will immediately notify the related doctors, psychologists, and other medical staff, and deal with the discontinuation.

10. 研究に用いられる情報に係る資料の保管及び廃棄の方法

10. Method for storage and disposal of information including records related to information utilized in research

個人情報管理者は、同意書など個人情報を含む研究必須文書等の紙媒体や電子媒体、個人情報と研究用 ID の対照表を、認知行動療法センターに設置した鍵のかかる保管庫にて漏洩、盗難、紛失しないように管理・保存する。紙媒体や電子媒体は、研究終了後から 5 年間経過後に、個人情報を分からなくしたことを確認した上で破棄される。なお、個人情報と研究用 ID の対照表の破棄後、連結不可能匿名化状態となった電子データベースは、追加解析や他の研究のため利用されることがある。この連結不可能匿名化データの電子ファイルは認知行動療法センター内の LAN やインターネットに接続されていないパソコンにてパスワードをかけて暗号化した上でハードディスクドライブに保存し、研究責任者の管理のもと保存される。なお、録音・録画された記録の一部については、コンサルテーションや事例検討のため

に、セキュリティの確保されたクラウド上で共有されることがある。共有された録音・録画データは、毎回のコンサルテーションや事例検討後に、確実にデータ消去を行う。

本試験のデータが他機関にて分析されたり、他の研究のために利用されたりする具体的な予定はないものの、その可能性は否定できない。本試験の匿名化されたデータの将来的な活用については、被験者に説明し、同意を得る。

A manager of private information will store the necessary documents or electronic records that contain personal information (such as documents for informed consent) and correspondence table for personal information and research ID in a locked cabinet to prevent any divulgation, theft, or loss. All documents and electronic records will be disposed of after the termination of research with erasure of personal information. After the disposal of correspondence table, the unlinked anonymized data may be used for additional analysis or other research purposes. The primary investigator will store the unlinked anonymized data in the encrypted HDD under password lock separated from any LAN or Internet. Parts of the recorded audio or video may be shared on a secure cloud system for case consultation. Such shared audio or video dates will be erased with absolute certainty after every consultation session.

Although we have no plans to transfer the data to other institutions or to use the data for other research purposes at this time, we cannot exclude these possibilities. We will obtain informed consent from the participants regarding the future utilization of the anonymized data.

11. 研究機関の長への報告内容及び方法

11. Matters to be reported to the chief executive of the research implementing entity and relevant procedures

研究機関の長（理事長）への報告については下記の通りとする。

Primary investigator will report to the chief executive as follows.

5） 年 1 回、研究実施状況について報告し、研究継続の適否について倫理委員会の審査を受ける。

6） 重篤な有害事象が発生した場合は、速やかに理事長に報告し、研究継続の適否について倫理委員会の審査を受ける。

7） 介入法の有効性・安全性に関する重要な情報が得られた場合は、研究責任者の見解を記載し、理事長に報告し、研究継続の適否について倫理委員会の審査を受ける。

8） 研究の終了時（中止または中断の場合を含む）には、理事長に報告する。

5） Primary investigator will report the progress of research once a year. The IRB will review it and examine the appropriateness of continuing the research.

6) If a severe adverse event occurs, the primary investigator will immediately report it to the chief executive. The IRB will review it and examine the appropriateness of continuing the research.

7) If the primary investigator acknowledges any important information regarding the efficacy and safety of the intervention, the primary investigator will report the event with assessment by the investigator. The IRB will review it and examine the appropriateness of continuing the research.

8) Primary investigator will report the termination or discontinuation of research.

12. 研究に係る資金と利益相反に関する状況

12. Status of research-related conflicts of interest of the research implementing entity, such as research fund resources, and research-related conflicts of interest of each investigator, such as his/her individual income

本研究は、"日本医療研究開発機構研究費　平成 29 年度「統合医療」に係る医療の質向上・科学的根拠収集研究事業　1-4 漢方及び鍼灸を除く各種療法に関する科学的知見を創出するための研究"に対して、課題名『超高周波音響療法による認知行動療法の増強効果』を資金源とし、研究代表者は堀越勝、研究期間は本倫理申請承認から平成 32 年 3 月 31 日である。また、行動指標をアウトカムとした附属研究については、パブリックヘルス財団　2017 年度ストレス科学分野「ストレスマネジメント」に対して、課題名『認知行動療法面接中における超高周波音響呈示のブースト効果研究：抑うつ症状の改善に焦点を当てて』を資金源とし、研究代表者は宮前光宏、研究期間は 2017 年４月１日から 2018 年 3 月 31 日で助成を受けている。また、本研究に係る全ての研究者及びその配偶者などの家族は、本研究で用いる超高周波音響装置を製造している業者との間に経済的利害関係、雇用関係は一切無い。従って、研究者が企業等とは独立して計画し実施するものであり、研究結果及び解析等に影響を及ぼすことは無い。本研究に携わる研究者等は、いずれも利益相反状態にないことを確認している。

Funding resources for this research are provided by the Japan Agency for Medical Research and Development as the Grant for Research Project for Improving Quality in Healthcare and Collecting Scientific Evidence on Integrative Medicine (2017). The approved title for the project was "Augmentation of cognitive behavior therapy by inaudible high-frequency sound therapy" awarded to Masaru Horikoshi. The planned study period is from the approval date of this research protocol by IRB to 31st of March 2020. The ancillary study for examining the augmentation effect of inaudible high-frequency sound of positive valence system-focused CBT on the performance of behavioral tasks is funded by the Public Health Research Foundation as the research grant for stress management in the field of stress science ("Research on the boosting effect of inaudible high-frequency sound exposure during the session of cognitive behavioral therapy: Focusing on the improvement of depression") awarded to Mitsuhiro Miyamae. The research period was from 1st of April 2017 to 31st of March 2018.

None of the researchers involved in this project, or their family members, had any conflict of interest with the company that built the audio systems. Hence, this research will be conducted independently of any company or other entity, resulting in no influence on the results or analysis. All of the relevant researchers in this study confirmed that they had no conflict of interest.

13. 研究に関する情報公開の方法

13. Means to disclose research information

臨床試験登録

Registration in clinical trial registry

本臨床試験は、大学病院医療情報ネットワーク(UMIN)「臨床試験登録システム」に登録予定である。

This clinical trial will be registered in a public database operated by the National University Hospital Council of Japan.

成果の帰属と公表

Attribution and publication of research outcomes

本研究によっていかなる結果が得られても、その結果は被験者を特定できないようにした上で、学術専門誌などにて公表される。Primary Outcome Paper とプロトコル論文は伊藤が草稿を執筆し、共同主任研究者（堀越）がその内容を確認した上で、共著者の確認・修正を全著者が納得するまで繰り返した上で投稿する。二次解析などの他の論文は、Primary Outcome Paper が公表された後に公表することとする。行動指標をメインアウトカムとして、治療メカニズムを検証する論文に関しては、宮前が草稿を執筆し、共著者の確認・修正を全著者が納得するまで繰り返した上で投稿する。

Regardless of the results, the primary investigator will publish the results in anonymized format in the academic journal. MI will write the first draft for the primary outcome paper and protocol paper. After confirmation by the co-primary investigator (MH), the draft will be repeatedly modified until all authors have approved the final version. Other papers for secondary analysis will be published after acceptance of the primary outcome paper. For the behavioral task outcomes, MM will write a first draft and continually modify the paper until all co-authors have given approval.

14. 研究対象者等及びその関係者からの相談等への対応

14. Means to respond to inquiries made by the research subjects and/or other individuals concerned

研究対象者等及びその関係者からの相談については、下記相談窓口を説明文書に明示し、申し

出があった場合には遅滞なく対応する。

The primary investigator will reveal the address and phone number of the inquiry counter on the document used for informed consent for any possible consultation by subjects or related individuals. If any consultation is raised, the primary investigator will respond immediately.

研究相談窓口

Inquiry counter

〒187-8551

東京都小平市小川東町四丁目 1 番 1 号

国立研究開発法人国立精神・神経医療研究センター 認知行動療法センター

電話番号 042-341-271２(内線 3605 または 3606)

対応時間：月・水・金（9 : 30−17 : 00）

氏名　伊藤正哉　所属・職名 認知行動療法センター 室長

Masaya Ito,

Ogawa higashi 4-1-1, Kodaira, Tokyo,

National Center of Neurology and Psychiatry, 187-8551

Phone: 042-341-2712 (Ex 3605, 3606)

Time: 9:30 to 17:00; Monday, Wednesday, and Friday

倫理委員会事務局への連絡先

Contact to Institution Review Board

〒187-8551

東京都小平市小川東町四丁目 1 番 1 号

国立研究開発国立精神・神経医療研究センター倫理委員会事務局

e-mail:rinri-jimu@ncnp.go.jp

Ogawa higashi 4-1-1, Kodaira, Tokyo,

National Center of Neurology and Psychiatry, 187-8551

Executive office of IRB

e-mail: rinri-jimu@ncnp.go.jp

15. 研究対象者等に経済的負担又は謝礼がある場合には、その旨及びその内容

15. Financial expenditure or remuneration for research subjects and associated details

本研究への参加により追加でご負担いただく費用、また、研究参加に対する謝礼はない。

There is no financial expenditure or remuneration for participation in this research.

24. 侵襲（軽微な侵襲を除く。）を伴う研究において重篤な有害事象が発生した際の対応

16. Means to respond in the case of serious adverse events (e.g., if the research involves invasiveness, not including minor invasiveness)

有害事象及び副作用の定義

Definition of adverse event or side effect

有害事象には、研究期間中に発生する有害と認められるあらゆる事象が含まれる。

An adverse event could be any adverse phenomenon during participation in this research.

有害事象の評価及び判定規準

Evaluation and criteria for adverse event

所定の項目（口渇、便秘、排尿障害、視力調節障害、起立性低血圧、眠気、倦怠感、不眠、不安・焦燥、落ち込み・意欲低下、食欲不振、体重増加、体重減少、性欲低下、動悸、ふるえ、発汗、頭痛、ふらつき、その他）について、その有無を確認する。担当者は所定の用紙を用いて、「前回以来、身体的または精神的な症状で急に悪化したり発生しましたか」と口頭にて尋ね、患者の応答を求める（Solicit 形式であり、Voluntary 形式ではない）。

The presence or absence of adverse events will be assessed using the following items: dry mouth, astriction, dysuria, vision dysregulation (accommodation disturbance), orthostatic hypotension, sleepiness, fatigue, sleeplessness, anxiety/agitation, depression/anhedonia, lack of appetite, gain or loss of body weight, loss of sexual desire, palpitations, thrill, diaphoresis, headache, dizziness, other. Using forms, research staff will ask about any adverse event as follows, “Have you been experiencing any worsening or occurrence of physical or mental symptoms?”

有害事象の報告と発現時の対応

Response to adverse events

本研究では被験者の Visit ごとに、有害事象全体の評価を行う。有害事象が発現した場合、担当者はその内容と重篤性を評価する。担当者は出来る範囲で必要な対応を施し、研究責任者と相談の上で必要と判断され、被験者がうつ症状について何らかの治療を受けている場合には、NCNP での主治医または外部医療機関での主治医と相談し、必要な処置を施すよう求め、経過を充分観察することとする。担当者は有害事象の内容、発現日・消失日、程度、処置、転帰、重篤性評価、治療との関連性等を CRF に記載す

る。また、有害事象に対する治療が必要となった場合には、担当医または主治医と相談の上、担当者、担当医、または主治医が被験者にその旨を伝える。

We will evaluate the occurrence of any adverse event at every visit. In the case of an adverse event, research staff will evaluate the content and severity, and enact the necessary response. If the patient receives usual outpatient treatment in NCNP hospital or other medical institutes, the primary investigator will report the event to the primary doctors and request the necessary responses under their treatment. The primary investigator will continue the course after the occurrence of an adverse event. The research staff will record the content of the adverse event, occurrence date, date of improvement, severity, conducted response, course, and relevance to the intervention on the CRF. If additional treatment is required for the adverse event, the primary investigator, therapist, or primary doctor will provide an explanation.

予測される有害事象等

Expected adverse events

精神障害を対象とした治療において、もっとも注意が払うべき有害事象として、自殺念慮や企図が挙げられる。大規模な疫学調査によれば、大うつ病性障害における自殺企図は OR=3.2（95% CI 2.5–4.2）と報告されている(Nock et al., 2009)。その他に、上記に挙げた症状はうつ症状を有する者において予測される有害事象であると考えられる。

Generally, adverse events that should be taken into consideration are suicidal thoughts and attempts. According to the result of a large scale epidemiological survey, suicidal attempts among patients with major depressive disorder is OR=3.2 (95% CI 2.5–4.2) (Nock et al., 2009). Other expected adverse events are reflected in the items for adverse event.

25. 当該研究によって生じた健康被害に対する補償の有無及びその内容

17. Compensation for any harm caused by study participation

万が一、本研究の介入期間中に健康被害が生じた場合、適切な医療等の対応が行われるように図る。本研究では、研究に伴う健康被害のリスクは、一般の言語をコミュニケーション（面接、診療など）に伴うもの以上は特にないものと考えられるため、健康被害に対する医療も通常の診療と同様に、被験者の保険診療内で行う。なお、この本研究への参加に起因した健康被害が生じた場合、補償金、医療費・医療手当等の補償は行われないことを、あらかじめ被験者から同意を得るものとする。

If any harm occurs due to participation in this research, the primary investigator will respond to ensure the appropriate medical care or treatment. As we do not expect the risk of any harm beyond what typically occurs for face-to-face verbal communication (e.g., interview or examination), any medical care for harm will be conducted using medical insurance as is typically used for usual care. We will

provide explanations and obtain consent from subjects about the lack of compensation for any harm during participation in this research.

26.　　研究対象者への研究実施後における医療の提供に関する対応

18. Response related to healthcare delivery to research subjects following research

本試験参加終了後においても、本試験に関する疑問等を被験者が抱いた場合には、研究事務局への連絡ができるものとする。

If the subject raises any questions regarding this trial after completing participation, the subject will be able to contact the inquiry counter of this research.

27.　　研究対象者に係る研究結果（偶発的所見を含む。）の取扱い

19.　Means of handling subjects' information (including incidental findings)

本研究において、偶発的所見が発見される可能性はほとんど存在しない。

We expect no incidental findings from participation in this trial.

28.　　委託する業務内容及び委託先の監督方法

20. Content of the work to be entrusted and means of supervision over the contractors

本研究において、外部組織に業務を委託する予定はない。

No part of the work will be entrusted.

29.　　試料及び情報の二次利用とそれに伴う他機関への提供の可能性

21.　Possibility of secondary utilization and/or provision of the specimens and information acquired from subjects

本試験のデータが他機関にて分析されたり、他の研究のために利用されたりする具体的な予定はないものの、その可能性は否定できない。本試験の匿名化されたデータの将来的な活用については、被験者に説明し、同意を得る。

Although there is currently no plan for other institutions to analyze or utilize the data acquired by this trial, we cannot exclude this possibility. We will obtain informed consent from the subjects regarding the future utilization of anonymized data.

30.　　モニタリング及び監査

22. Monitoring and audit

（1）モニタリング

(1) Monitoring

本試験では、研究責任者自身が、コーディネーター及びデータ管理者と連携して、施設モニタリング及び中央モニタリングを行い、その結果を効果安全性委員に報告する。基本的には、データ管理者が CRF 又は原資料とデータベースに入力されたデータの照合（施設モニタリング）を行う。中央モニタリングは解析担当者が行い、年に一度（10 月）研究責任者から効果安全性委員に報告書が送られる。中央モニタリングは全症例に対して行い、施設モニタリングは登録された最初の 3 例を対象に行い、その後、登録第 4 – 44 番目の症例に対して、予めランダム抽出しておいた 3 例を対象に行う。どの事例をランダム抽出したかは、CRF 作成者およびデータ入力担当者には伝えないようにする。研究責任者及び効果安全性委員会の委員が必要性を指摘した場合には、モニタリング委員会を開催する。また、効果安全性委員は、データ管理者へとデータ公開を求めることができる。データ取得と入力、モニタリングの流れは以下の通りである。

The primary investigator, in collaboration with the research coordinator and data manager, will conduct on-side and central monitoring, and report the results to the members of DSMB. The data manager will verify the entered data by referring to the CRF and/or primary source material (i.e., on-site monitoring). The statistician will conduct the central monitoring. The primary investigator will report the results to members of DSMB once a year (in October). All registered cases will be subject to central monitoring. On-site monitoring will be conducted for the first three registered cases, and then for three randomly pre-identified cases from the 4th to 44th registered cases. Developer of CRF and staff for entering data will not be masked to the pre-identified cases for on-site monitoring. A meeting for monitoring will be convened if the primary investigator or members of DSMB suggest the need. In addition, the members of DSMB may request data from the data manager. The procedure for data acquisition, entry, and monitoring is as follows.

8. 研究候補者に関係する First contact（紹介等）：コーディネーターが研究用 ID を付与
9. 同意取得後、ベースライン評価：評価結果をコーディネーターが CRF に記入
10. コーディネーター作成の CRF 及び自記式尺度（原資料）を研究スタッフがデータベースに入力
11. 担当者が基礎情報に関する CRF を記入
12. プロセス指標等、介入群の評価結果を研究スタッフがデータベースに入力
13. 中間・介入後評価結果をコーディネーターが CRF に入力

14. コーディネーターが記入した CRF 及び自記式尺度（原資料）を研究スタッフがデータベースに入力

※ 随時、データ管理者が CRF 及び自記式尺度と入力されたデータの照合（施設モニタリング）

※ 中央モニタリングは解析担当者が行う

※ 中央モニタリング毎に、研究責任者が効果安全性委員に報告書を送付

8. First contact with the participant (referral, etc.): Research coordinator gives research ID to the participant.
9. Baseline assessment after obtaining informed consent: Research coordinator records the assessment results of on the CRF.
10. Research staff will enter the data by referring to the CRF and self-report measures (primary source).
11. Therapist will fill in the CRF regarding the basic information.
12. Research staff will enter the results of process measures and other assessments.
13. Research coordinator will fill in the CRF regarding the results of mid- and post-assessment.
14. Research staff will enter the information on the CRF and self-report measures.
    - Data manager will check the accuracy of data entry from the CRF and self-report measures (i.e., on-site monitoring).
    - Statistician will conduct central monitoring.
    - Primary investigator will send the central monitoring report document to the members of DSMB.

（2）監査

(2) Audit

本試験についての監査は予定していない。

No audit is planned.

31. 用語の解説

23. Explanation of terms

アンヘドニア

Anhedonia

古典的には「歓びの喪失」として定義される状態を指す（Ribot, 1896）。ただし、近年で

は、この定義はアンヘドニアの一側面を表現しているだけに過ぎないという指摘があり、より多面的な構成概念であることが示唆されている。具体的には、アンヘドニアは研究領域基準におけるポジティブ価システムの失調として捉えることができ、報酬獲得に対する動機づけおよびエフォートの低減、報酬獲得時の快感情の低減および持続困難、報酬獲得に関する学習の困難などを含む状態と考えられる。

Anhedonia is classically defined as the “loss of pleasure (Ribot, 1896).” Recently, this definition has been criticized as it only partially describes anhedonia, and there are suggestions that that anhedonia comprises multiple aspects. Specifically, anhedonia may be regarded as the dysregulation of the positive valence system within the framework of Research Domain Criteria, which includes the decline of motivation and effort for reward acquisition, decrease of intensity and maintenance of positive emotion at the time of reward acquisition, and difficulty in reward learning.

認知行動療法

Cognitive behavioral therapy

人はうつや不安状態になると物事のとらえ方が、より悲観的・否定的になり、その結果気分や行動が影響されるという理解にもとづき、その人の認知のあり方を治療者との対話を通じて検討し、問題解決につながる方策を案出し、実施することにより、その人の気分の改善を図ることを目的とした、構造化された精神療法の１つ。

Cognitive behavioral therapy (CBT) is a form of structured psychotherapy. CBT is based on the concept that humans experiencing depressed mood or anxiety tend to think negatively, and such cognition affects behavior. In CBT, the therapist examines patients’ cognition, elaborates, and tries ways to solve problems to improve the patients’ mood.

24. 参考文献リスト、研究に関係する指針・ガイドライン

24. References and related guidelines

45. Alexopoulos, G. S., Raue, P. J., Gunning, F., Kiosses, D. N., Kanellopoulos, D., Pollari, C., Banerjee, S., & Arean, P. A. (2016). “Engage” therapy: behavioral activation and improvement of late-life major depression, *The American Journal of Geriatric Psychiatry*, **24**(4), 320-326.

46. Ameli, R., Luckenbaugh, D.A., Gould, N. F., Holmes, M. K., Lally, N., Ballard, E.D., & Zarate, C.A. Jr. (2014) SHAPS-C: the Snaith-Hamilton pleasure scale modified for clinician administration. *PeerJ*, **17**(2), e429

47. Armento, M. E., & Hopko, D. R. (2007). The Environmental Reward Observation Scale (EROS): development, validity, and reliability, *Behavior Therapy*, **38**(2), 107-119.

48. Bang, H., Ni, L., & Davis, C. E. (2004). Assessment of blinding in clinical trials. *Controlled Clinical Trials*, **25**(2), 143-156.

49. Barlow, D. H., Gorman, J. M., Shear, M. K., & Woods, S. W. (2000). Cognitive-behavioral therapy, imipramine, or their combination for panic disorder: A randomized controlled trial. *JAMA*, **283**(19), 2529-2536.

50. Beck, A. T., Steer, R. A., & Brown, G. K. (1996). *Manual for the Beck Depression Inventory-II.* San Antonio, TX: Psychological Corporation. (小嶋雅代・古川壽亮（訳）(2003). 日本版 BDI-II—ベック抑うつ質問票—手引き　日本文化科学社).

51. Blom, E. H., Tymofiyeva, O., Chesney, M. A., Ho, T. C., Moran, P., Connolly, C. G., Duncan, L. G., Baldini, L., Weng, H. Y., Acree, M., Goldman, V., Hecht, F. M., & Yang, T. T. (2016). Feasibility and preliminary efficacy of a novel RDoC-based treatment program for adolescent depression: “Training for Awareness Resilience and Action” (TARA)—A pilot study. *Frontiers in Psychiatry*, **7,** 208.

52. Carl, J. R., Soskin, D. P., Kerns, C., & Barlow, D. H. (2013). Positive emotion regulation in emotional disorders: A theoretical review. *Clinical Psychology Review*, **33**, 343-360.

53. Clark, L. A., & Watson, D. (1988). Mood and the mundane: Relations between daily life events and self-reported mood. *Journal of Personality and Social Psychology*, **54,** 296-308.

54. Clark, L. A., & Watson, D. (1989). *The Japanese Positive and Negative Affect Schedule: Factor-based scales for the assessment of mood.* Unpublished manuscript. University of Iowa.

55. Craske, M. G., Meuret, A. E., Ritz, T., Treanor, M., & Dour, H. J. (2016). Treatment for anhedonia: A neuroscience driven approach. *Depression and Anxiety*, **33**, 927–938.

56. Cocks, K., & Torgerson, D. J. (2013). Sample size calculations for pilot randomized trials: a confidence interval approach. *Journal of Clinical Epidemiology*, **66**(2), 197-201.

57. Diener, E., Emmons, R.A., Larsen, R.J., & Griffin, S. (1985). The Satisfaction With Life Scale. *Journal of Personality Assessment*, **49**, 71-75.

58. Franken, I. H. A., Rassin, E., & Muris, P. (2007). The assessment of anhedonia in clinical and non-clinical populations: Further validation of the Snaith-Hamilton Pleasure Scale (SHAPS). *Journal of Affective Disorders*, **99**, 83-89.

59. Farchione, T. J., Fairholme, C. P., Ellard, K. K., Boisseau, C. L., Thompson-Hollands, J., Carl, J. R., Gallagher, M. W., & Barlow, D. H. (2012). Unified protocol for transdiagnostic treatment of emotional disorders: A randomized controlled trial. *Behavior Therapy*, **43**(3), 666–678.

60. Hamilton, M. (1960). A rating scale for depression. *Journal of Neurology, Neurosurgery & Psychiatry*, **23**, 56-62.

61. Harvey, A. G., Lee, J., Smith, R.L., Gumport, N.B., Hollon, S. D., Rabe-Hesketh, S., Hein, K., Dolsen, M. R., Hman, K. L., Kanady, J. C., Thompson, M.A., & Abrons, D. (2016). Improving outcome for mental disorders by enhancing memory for treatment. *Behaviour Research and Therapy*, **81**, 35-46.

62. Insel T, Cuthbert B, Garvey M, Heinssen R, Pine DS, Quinn K, Sanislow, C., & Wang, P. (2010). Research domain criteria (RDoC): toward a new classification framework for research on mental disorders. *American Journal of Psychiatry*, **167**, 748–751.

63. Kitamura, T., Kishida, Y., Katayama, R., Matsuoka, T., Miura, S. & Yamabe, K. (2003). Ryff's psychological well-being inventory: factorial structure and life history correlates among Japanese university students. *Psychological Reports*, **94**, 83-103.

64. Kojima, M., Furukawa, T. A., Takahashi, H., Kawai, M., Nagaya, T., & Tokudome, S. (2002). Cross-cultural validation of the Beck Depression Inventory-II in Japan. *Psychiatry Research*, **110**, 291-299.

65. 国里愛彦・高垣耕企・岡島義・中島俊・石川信一・金井嘉宏・岡本泰昌・坂野雄二・山脇成人. (2011). 日本語版 Environmental Reward Observation Scale (EROS) の作成と信頼性・妥当性の検討. 行動療法研究, **37**(1), 21-31.

66. 文部科学省・厚生労働省. (2014). 人を対象とする医学系研究に関する倫理指針.

67. 文部科学省・厚生労働省. (2015). 人を対象とする医学系研究に関する倫理指針　ガイダンス.

68. Nagayama, H., Kubo, S., Hatano, T., Hamada, S., Maeda, T., Hasegawa, T., Kadowaki, T., Terashi, H., Yoshioka, M., Nomoto, N., Kano, O., Inoue, M., Shimura, H., Takahashi, T., Uchiyama, T., Watanabe, H., Kaneko, S., Takahashi, T., & Baba, Y. (2012). Validity and reliability assessment of a Japanese version of the Snaith–Hamilton pleasure scale. *Internal Medicine.* **51,** 865–869.

69. National Collaborating Centre for Mental Health. (2010). Depression: the treatment and management of depression in adults. Updated ed. Leicester: British Psychological Society/London: Royal College of Psychiatrists.

70. Nock, M. K., Hwang, I., Sampson, N. A., & Kessler, R. C. (2009). Mental disorders, comorbidity and suicidal behavior: Results from the National Comorbidity Survey Replication. *Molecular Psychiatry*, **15**(8), 868–876.

71. Oohashi, T., Nishina, E., Honda, M., Yonekura, Y., Fuwamoto, Y., Kawai, N., Maekawa, T., Nakamura, S., Fukuyama, H., & Shibasaki, H. (2000). Inaudible high-frequency sounds affect brain activity: hypersonic effect. *Journal of Neurophysiology*, **83**, 3548-3558.

72. Ori, R., Amos, T., Bergman, H., Soares-Weiser, K., Ipser, J. C., & Stein, D. J. (2015). Augmentation of cognitive and behavioural therapies (CBT) with d-cycloserine for anxiety and related disorders. *Cochrane Database of Systematic Reviews*, **5**, Art. No.: CD007803. DOI: 10.1002/14651858.CD007803.pub2.

73. Otsubo, T., Tanaka, K., Koda, R., Shinoda, J., Sano, N., Tanaka, S., Aoyama, H., Mimura, M., Kamijima K. (2005). Reliability and validity of Japanese version of the mini-international neuropsychiatric interview. *Psychiatry and Clinical Neurosciences*, **59**(5), 517-526.

74. Pelizza, L., & Ferrari, A. (2009). Anhedonia in schizophrenia and major depression: state or trait? *Annals of General Psychiatry*, **8**(22), doi: 10.1186/1744-859X-8-22

75. Pizzagalli, D. A. (2014). Depression, stress, and anhedonia: toward a synthesis and integrated model. *Annual Review of Clinical Psychology*, **10,** 393–423.

76. Primakoff, L., Epstein, N., & Covi, L. (1986). Homework compliance: An uncontrolled variable in cognitive therapy outcome research. *Behavior Therapy*, **17**, 433–446.

77. Ribot T. (1896). La Psychologie des Sentiment [The Psychology of Feelings]. Paris: Felix Alcan.

78. Roll, D., Ray, S. E., Marcus, S. M., Passarelli, V., Money, R., Barlow, D. H., Wood, S. W., Shear, M K., & Gorman, J. M. (2004). Independent evaluator knowledge of treatment in a multicenter comparative treatment study of panic disorder. *Neuropsychopharmacology*, **29**(3), 612-618.

79. Ryff, C. D. (1989). Beyond Ponce de Leon and life satisfaction: new directions in quest of successful aging. *International Journal of Behavioral Development*, **12**, 35-55.

80. Snaith, R.P., Hamilton, M., Morley, S., Humayan, A., Hargreaves, D., & Trigwell, P. (1995). A scale for the assessment of hedonic tone the Snaith–Hamilton Pleasure Scale. *British Journal of Psychiatry*. **167**, 99–103.

81. Sheehan, D. V., Lecrubier, Y., Harnett-Sheehan, K., Amorim, P., Janavs, J., Weiller, E., Hergueta, T., Baker, R., & Dunbar, G. (1998). The Mini International Neuropsychiatric Interview (M.I.N.I.): The Development and Validation of a Structured Diagnostic Psychiatric Interview. *Journal of Clinical Psychiatry*, **59**(20), 22-33.

82. 角野善司. (1994). 人生に対する満足尺度（the Satisfaction With Life Scale [SWLS]）日本語版作成の試み. 日本教育心理学会総会発表論文集, **36**, 192.

83. Tabuse, H., Kalali, A., Azuma, H., Ozaki, N., Iwata, N., Naitoh, H., Higuchi, T., Kanba, S., Shioe, K., Akechi, T., & Furukawa, T. A. (2007). The new GRID Hamilton Rating Scale for depression demonstrates excellent inter-rater reliability for inexperienced and experienced raters before and after training. *Psychiatry Research*, **153**(1), 61–67.

84. Taylor, C. T., Lyubomirsky, S., and Stein, M. B. (2017). Upregulating the positive affect system in anxiety and depression: Outcomes of a positive activity intervention. *Depression and Anxiety*, **34**, 267–280.

85. Treadway, M. T., Buckholtz, J. W., Schwartzman, A. N., Lambert, W. E., & Zald, D. H. (2009). Worth the 'EEfRT'? The effort expenditure for rewards task as an objective measure of motivation and anhedonia. *PLoS ONE*, **4**(8), e6598.

86. Williams, J. B., Kobak, K. A., Bech, P.,. Evans, K., Lipsitz, J., Olin, J., Pearson, J., & Kalali, A. (2008). The GRID- HAMD: standardization of the Hamilton Depression Rating Scale. *International Clinical Psychopharmacology*, **23**(3), 120–129.

87. Vos, T., Barber, R. M., Bell, B., Bertozzi-Villa, A., Biryukov, S., Bolliger, I., et al. (2015). Global, regional, and national incidence, prevalence, and years lived with disability for 301 acute and chronic diseases and injuries in 188 countries, 1990–2013, a systematic analysis for the Global Burden of Disease Study 2013. *Lancet*, **386**(9995), 743–800.

研　究　計　画　書

Study Protocol

第 1 版：2017 年 9 月 6 日

First version: 6th, September, 2017

研究課題名	**アンヘドニアに対するポジティブ価システムに焦点を当てた認知行動療法の超高周波音響療法による増強効果：プラセボ対照ランダム化比較試験**
Research title	**Augmentation of positive valence system-focused cognitive behavior therapy by inaudible high-frequency sound therapy: A placebo-controlled randomized trial**
研究責任者（所属）	**伊藤正哉（認知行動療法センター研修指導部）**
Principal Investigator	**Masaya Ito, Ph.D（National Center of Cognitive-Behavior Therapy and Research, National Center of Neurology and Psychiatry）**

1. 研究の名称

1. Research title

アンヘドニアに対するポジティブ価システムに焦点を当てた認知行動療法の超高周波音響療法による増強効果：プラセボ対照ランダム化比較試験

Augmentation of positive valence system-focused cognitive behavior therapy by inaudible high-frequency sound therapy: A placebo-controlled randomized trial

2. 研究の実施体制

2. Site-specific research information

【国立精神・神経医療研究センターにおける共同研究者】

【Study members in National Center of Neurology and Psychiatry】

氏名<br>Name	所属<br>Affiliations	研究における役割及び責務<br>Roles and responsibilities	倫理講座の受講の有無（1年以内）<br>Experience in research ethics workshop participation	本研究に関する利益相反申告状況<br>Status of conflict of interest declaration
伊藤正哉<br>Masaya Ito	認知行動療法センター<br>National Center of	研究責任者、研究デザイン、認知行動療法の開発と実	あり<br>Yes	2017年9月6日提出済<br>Submitted on 6th

	Cognitive-Behavior Therapy, National Center of Neurology and Psychiatry	施<br><br>Principal investigator, study design, development of cognitive behavioral therapy protocol and implementation		of September, 2017
堀越勝<br>Masaru Horikoshi	認知行動療法センター<br>National Center of Cognitive-Behavior Therapy, National Center of Neurology and Psychiatry	研究デザイン<br><br>Study design	あり<br><br>Yes	2017 年 9 月 6 日提出済<br>Submitted on 6th of September, 2017
本田学<br>Manabu Honda	神経研究所<br>National Institute of Neuroscience, National Center of Neurology and Psychiatry	音響プロトコルの開発と運用<br>Development of protocol and management of audio intervention	あり<br><br>Yes	2017 年 9 月 6 日提出済<br>Submitted on 6th of September, 2017
山下祐一<br>Yuichi Yamashita	神経研究所<br>National Institute of Neuroscience, National Center of Neurology and Psychiatry	音響プロトコルの開発、音響機器のメンテナンス 割付の順番作成<br>Development of audio intervention protocol, maintenance of audio devices, generation and storage of comparison table of randomized	あり<br><br>Yes	2017 年 9 月 6 日提出済<br>Submitted on 6th of September, 2017

		sequences and sound tracks		
宮前光宏<br>Mitsuhiro Miyamae	神経研究所<br>National Institute of Neuroscience, National Center of Neurology and Psychiatry	研究デザイン、認知行動療法の開発と実施、症状評価、附属研究の主任研究者<br>Study design, development of cognitive behavioral therapy protocol and implementation, symptom assessment, PI for ancillary study	あり<br><br>Yes	2017年9月6日提出済<br>Submitted on 6th of September, 2017
上野修<br>Osamu Ueno	神経研究所<br>National Institute of Neuroscience, National Center of Neurology and Psychiatry	音響プロトコルの開発と運用、音響機器のメンテナンス<br>Development of protocol and management of audio intervention, maintenance of audio devices	あり<br><br>Yes	2017年9月6日提出済<br>Submitted on 6th of September, 2017
横山知加<br>Chika Yokoyama	認知行動療法センター<br>National Institute of Neuroscience, National Center of Neurology and Psychiatry	認知行動療法の開発と実施、試験コーディネート<br>Development of cognitive behavioral therapy protocol and implementation, coordination of trial	あり<br><br>Yes	2017年9月6日提出済<br>Submitted on 6th of September, 2017
伊藤まど	精神保健研究所成人	認知行動療法の開	あり	2017年9月6日

か<br>Madoka Ito	精神保健研究部･流動研究員<br>National Institute of Mental Health, Neuroscience, National Center of Neurology and Psychiatry	発<br>Development of cognitive behavioral therapy protocol	Yes	提出済<br>Submitted on 6th of September, 2017
丸尾和司<br>Kazushi Maruo	トランスレーショナル･メディカルセンター<br>Translational Medical Center, National Center of Neurology and Psychiatry	解析計画書の作成と、解析の実施<br>Development of statistical analysis plan and statistical analysis	あり<br><br>Yes	2017 年 9 月 6 日提出済<br>Submitted on 6th of September, 2017
駒沢あさみ<br>Asami Komazawa	認知行動療法センター<br>National Institute of Neuroscience, National Center of Neurology and Psychiatry	認知行動療法の開発と実施<br>Development of cognitive behavioral therapy protocol and implementation	あり<br><br>Yes	2017 年 9 月 6 日提出済<br>Submitted on 6th of September, 2017

【国立精神・神経医療研究センターにおける共同研究者以外の研究協力者の実施体制】

【Personnel external to National Center of Neurology and Psychiatry】

**氏名**<br>**Name**	**所属・役職**<br>**Affiliations**	**研究における役割及び責務**<br>**Roles and responsibilities**	**倫理講座の受講の有無（1 年以内）**<br>**Experience in research ethics workshop participation**	**本研究に関する利益相反審査結果**<br>**Status of conflict of interest declaration**
田島美幸<br><br>Miyuki Tajima	認知行動療法センター<br><br>National Institute of Neuroscience, National Center of Neurology and Psychiatry	個人情報管理者・個人情報匿名化担当者<br>Administrator of anonymization management	あり<br><br>Yes	9 月 6 日提出済<br><br>Submitted on 6th of September, 2017

【効果安全性評価委員会】

【Members of Data Safety Monitoring Board】

所属<br>Affiliation	氏名<br>Name	専門<br>Expertise
武蔵野大学<br>Musashino University	中島聡美<br>Satomi Nakajima, M.D., Ph.D.	精神医学<br>Psychiatry
東京大学<br>Tokyo University	西大輔<br>Daisuke Nishi, M.D., Ph.D.	精神医学<br>Psychiatry

【モニタリング】

【Monitoring】

所属<br>Affiliations	氏名<br>Name	研究における役割及び責務<br>Roles and responsibilities
認知行動療法センター<br>National Center of Cognitive-Behavior Therapy, National Center of Neurology and Psychiatry	伊藤正哉<br>Masaya Ito	モニタリング責任者<br>Responsible for monitoring

3. 研究の背景、科学的合理性の根拠及び社会的意義

3. Research background, scientific validity, and social significance

精神疾患が個人と社会に及ぼす損失は甚大である。なかでも、うつ病は世界の疾病負担の 3 位である（Vos et al., 2015）。精神疾患はわが国の 5 大疾病のひとつであり、最も患者数が多い（約 323 万人）。うつ病と不安障害の 1 年間の時点有病率は 7.9%である。うつ病の 1 年間の社会経済コストは年間 3 兆 900 億円に上る。

認知行動療法(Cognitive Behavioral Therapies; CBT)は、学習理論や認知理論に基づき、治療手続きが体系化された精神療法の総称である。診療ガイドラインや系統的レビューによると、中等症以上のうつ病(大うつ病性障害、持続性抑うつ障害)には、認知行動療法が推奨されている（e,g., 英国医療技術評価機構（National Collaborating Centre for Mental Health）, 2010）。日本においても、うつ病に対する認知行動療法が健康保険適用となった。現在の日本では、認知行動療法をいかに均てん化するかについての努力が展開しているところである。

Mental disorders cause devastating effects at both the individual and societal level. Among them, depressive disorder ranks third as a cause of global disease burden (Vos et al., 2015). Mental disorders are designated as one of the big-five diseases in Japan and together constitute the largest patient population among these big-five diseases (more than 3 million and 230,000 patients in

Japan). The annual prevalence of depressive and anxiety disorders in Japan is reported to be 7.9%. The annual socio-economic cost of depressive disorder is estimated to be more than 3 trillion yen.

Cognitive behavioral therapy (CBT) is a form of psychotherapy with systematic intervention protocols based on learning and cognitive theories. According to treatment guidelines and the results of systematic reviews, cognitive behavioral therapy is recommended for treating depression with moderate or severe symptoms (major depressive disorder and dysthymia) (e.g., National Institute of Clinical Excellence, 2010). In Japan, CBT for depression was subjected to national medical insurance beginning in 2013. There is a current focus to increase the use of CBT in the Japanese medical setting.

うつ病治療において、いまや認知行動療法は第一治療選択のひとつである。しかし、依然として改良の余地も大きい。認知行動療法を受けても約半数が再発したり、治療反応を示さない患者も一定数いることが知られている。そうした背景から、認知行動療法の効果を増強させる先端研究が展開されつつある。例えば、d-cycloserine という物質（もともとは結核薬）を認知行動療法の前に投与することにより、不安症に対する認知行動療法で行われる“情報の再学習”が強化される可能性が期待されている。他にも、増強を検討するさまざまな研究が芽生えつつある（治療中の記憶力の増強（Harvey et al., 2016）。

CBT is currently the first-line treatment for depression. Nevertheless, there is significant scope for improvement. Approximately half of patients show relapse of depression after the completion of CBT. A substantial proportion of patients do not respond to CBT. Based on these shortcomings, there have been increased research efforts to augment the effect of CBT for depression. For example, d-cycloserine, originally utilized for treating tuberculosis, is used to augment “information re-learning” during CBT for anxiety disorders. Other techniques for augmenting the efficacy of CBT have also been used, such as augmentation of memory technique (Harvey et al., 2016).

われわれは、認知行動療法を増強させる方法として、“アンヘドニアに対するポジティブ価システムに焦点を当てた認知行動療法”と“ハイパーソニック・エフェクト（超高周波音響効果, Hypersonic Effect）”に着目した。まず、ポジティブ価システムに焦点を当てる認知行動療法とは、うつ病の中でもアンヘドニア症状を改善することに焦点を当てた認知行動療法である。アンヘドニア症状は従来の治療では改善されにくい症状であり、ポジティブ感情の低下、意欲の減退、報酬に対する感受性の低下、歓びの喪失などの様々な定義が与えられている。近年提唱された研究領域基準（Insel et al., 2010）においては、このアンヘドニア症状はポジティブ価システム（Positive-Valence System）の失調として捉えられる。ポジティブ価システムの下位概念として、接近動機づけ、報酬獲得への初発反応性、報酬獲得への反応性の維持と長期反応性、報酬学習、習慣が含まれている。近年の認知行動療法は、神経科学とポジティブ心理学の知見を導入し、これらのシステムを特に強化するため

の介入を導入し、うつ病治療の改善を検証しつつある（Craske et al., 2016, Taylor et al., 2016, Alexopoulos et al., 2016, Blom et al., 2016）。

We focused on the “positive valence system-focused CBT for anhedonia” and “hyper-sonic effect” (effect of inaudible hyper sound) for this study. Positive valence system-focused CBT is a CBT that focuses on improving anhedonia. Anhedonia is defined as decreased positive emotions, decreased motivation for performing activities, deceased sensitivity to rewarding stimuli, or loss of pleasure. Anhedonia is known to be resistant to conventional treatments for depression. In the context of Research Domain Criteria (Insel et al., 2010), anhedonia can be classified as a dysfunction of the positive valence system. Sub-constructs of the positive valence system are constituted by approach motivation, initial responsiveness to reward attainment, sustained/longer-term responsiveness to reward attainment, reward learning, and habit. Recent studies on CBT have attempted to incorporate findings from neuroscience and positive psychology to augment these systems and test their efficacy (Craske et al., 2016; Taylor et al., 2016; Alexopoulos et al., 2016; Blom et al., 2016）.

一方で、ハイパーソニック・エフェクトとは、人間の可聴域上限を超える超高周波成分を豊富に含む音響情報が、報酬系神経回路を含む脳深部の神経活動を活性化し、人体に全身的影響を及ぼすことを指す。これまで研究協同者（本田学）の研究グループは、この現象を複数の非侵襲脳機能イメージングと様々な生理活性指標を用いて明らかにしてきた（Oohashi et al., 2000 他）。具体的には、超高周波成分を豊富に含む音情報は、同じ音から超高周波成分を除去した音情報（ハイカット音）と比較して、脳幹、視床から前頭前野に拡がるモノアミン作動性神経投射を含む情動系神経回路や報酬系回路の血流を増加させるとともに、それと並行して脳波 α 波のパワーを増強させること、視床下部の活性化を反映して NK 細胞活性を上昇させ、ストレスホルモンを低下させるといった全身反応を導くことを明らかにした。加えて、超高周波成分を含む音情報は、音質を向上し音の快適性を増強させるとともに、超高周波成分を豊富に含む音をより多く受容しようとする接近行動を引き起こすなど、報酬系の活性化を反映した被呈示者の心理行動的効果を導く。

The hyper-sonic effect refers to the whole-body effect from exposure to inaudible high-frequency sound via the activation of deep brain activity including reward related neural circuits. Our research group has demonstrated the phenomenon using various non-invasive brain function imaging and physiological measures (Ohashi et al., 2000). For example, we have demonstrated that inaudible high-frequency sound, in comparison to high-cut placebo sound, increases blood flow in the reward circuity and affects related circuity including monoaminergic projections distributed across the brain stem, thalamus, and prefrontal region, in parallel with enhancement of alpha brain-wave power. Compared to high-cut placebo sound, inaudible high-frequency sound enhanced natural killer cell activation, which reflects hypothalamic activity

and decreased stress hormone levels. In addition, inaudible high-frequency sound promoted psycho-behavioral effects that reflect activation of the reward system. Therefore, inaudible high-frequency sound seems to robustly enhance the subjective experience of sound quality and comfort, and promotes approach behavior to listen to the sound.

こうした複雑に変化する超高周波成分は、人間の遺伝子が進化的に形成されたと考えられる熱帯雨林の環境音に豊富に含まれる一方で、現代人の多くが生活する都市環境音にはほとんど含まれない。そこで研究分担者らは、自然環境音に豊富に含まれ現代社会の環境音にほとんど含まれない複雑性をもった超高周波成分という「必須情報」の不足が、深部脳を起点とするモノアミン神経系の変調を介して、気分障害をはじめとする精神・神経疾患の発症に無視できない影響を及ぼす可能性があるのではないかとの仮説を立てた。この仮説のもと、平成 22〜24 年度の厚生労働科研費医療技術実用化総合研究事業では、超高周波成分を豊富に含む音響情報の曝露の前後で、うつ病患者の状態不安指標が有意に改善することを示した。

Although such inaudible high-frequency sound with complex, changing sound quality is prevalent in environmental sounds in tropical rain forests, it is absent in urban city settings in which most modern people around the world live. We hypothesized that inaudible high-frequency with complex sound quality could be conceptualized as “essential information”, and the lack of this could have considerable effects on the onset of mental and neurological disorders via the dysfunction of monoaminergic neural systems. Based on this hypothesis, we have demonstrated that the exposure to sound information with inaudible high-frequency sound significantly improved state anxiety among patients with depression.

このように、ハイパーソニック・エフェクトは様々なイメージング・生理・心理・行動指標で観察されてきた。なかでも今回われわれが注目したメカニズムとして、うつ病患者の精神症状の改善効果と、学習効果の増強に関する知見を指摘できる。前者においては、超高周波音響の呈示によって、脳内の報酬系回路が活性化させることにより、「必須情報」が補完された結果として解釈できる。認知行動療法においても、報酬系回路を活性化させる治療要素（行動活性化）が含まれているため、そうした治療要素との相乗効果が期待できる。さらに、ハイパーソニックの学習効果増強に関する研究では、超高周波音響を呈示されながら実施される認知課題（N-back 課題）では、呈示がない場合よりも優れた成績が示された。先に述べた通り、認知行動療法は“情報の再学習”を治療媒介とする。そのため、ハイパーソニック・エフェクトによる学習効果の増強は、認知行動療法の効果増強にも応用できると考えられる。

This hyper-sonic effect has been observed in various imaging, physiological, psychological, and behavioral measures. Among these, we focused on two findings of the hyper-sonic effect: the

improvement of mental status among patients with depression, and the enhancement of learning. The former could be interpreted as a supplement of “essential information” via activation of brain reward circuity by exposure to inaudible high-frequency sound. One of the intervention techniques in CBT, behavioral activation, is aimed at activating the reward circuity. Therefore, it is expected that inaudible high-frequency sound and behavioral activation may have a synergistic effect. Furthermore, the latter findings showed that performance on a cognitive task (N-back task) was superior in groups exposed to inaudible high-frequency sound compared to groups not exposed to the sound. As discussed above, one of the treatment mechanisms of CBT is “re-learning of information.” Hence, enhancement of learning performance by the hyper-sonic effect may be applicable for augmenting CBT.

4. 研究の目的及び意義

4. Research objectives and significance

上述の背景から、本研究ではポジティブ価システムに焦点を当てた認知行動療法と超高周波音響を併用することにより、うつ症状のなかでもとくにアンヘドニアが顕著に改善されるかどうかを検討することを目的とした。すなわち、本臨床試験は、アンヘドニア症状を呈する成人 44 名を被験者対象集団とし、ポジティブ価システムに焦点を当てた認知行動療法に超高周波音響を呈示した試験治療の、同治療に超高周波を含まないプラセボ音響を呈示した対照治療に対する、Snaith-Hamilton Pleasure Scale にて測定されるアンヘドニア症状への有効性に対する優越性を検証することを目的とする。

Based on the rationale discussed above, we aimed to test the efficacy of combining the positive valence system-focused CBT with inaudible high-frequency sound to improve anhedonia. The objective of this clinical trial is to test the efficacy of the positive valence system-focused CBT with inaudible high-frequency sound compared to the positive valence system-focused CBT with placebo sound on anhedonia symptoms (Snaith-Hamilton Pleasure Scale) among 44 patients with anhedonia.

本研究では、さまざまな認知行動療法の中でも、アンヘドニアに対するポジティブ価システムに焦点を当てた介入技法がハイパーソニック・エフェクトにより増強されるかどうかを検証する。もしその増強効果が同定されれば、今後はより大規模な検証的ランダム化比較試験に進む。同時に、様々な疾患（e.g., 全般不安症、社交不安症、パニック症、心的外傷後ストレス障害など）や認知行動療法の他の治療要素（e.g., モニタリング、認知再構成、エクスポージャー、マインドフルネス）への適用へと拡張させて増強効果を検証する。検証的試験により、パイパーソニック・エフェクトによる増強効果が確認できれば、これまで認知行動療法に反応しなかった患者や、再発を呈していた患者に対して、新たな治療選択肢を提示することができるようになる。

Among the various forms of CBT, we will test the augmentation effect for intervention techniques that are focused on the positive valence system for anhedonia. If augmentation effects are observed in this study, we will proceed to a larger confirmatory randomized controlled trial. In parallel, we will extend research on this augmentation effect for various disorders (e.g., generalized anxiety disorder, social anxiety disorder, panic disorder, posttraumatic stress disorder, etc.) and various treatment components of CBT (e.g., monitoring, cognitive restructuring, exposure, and mindfulness). Confirming the augmentation of CBT by the hyper-sonic effect will enable new treatment choices for patients who do not respond to CBT or who experience relapse following the completion of CBT.

本研究で増強効果が観察できた場合には、その科学的根拠は多方面に重要なインパクトを持つ。有効な精神療法は認知行動療法以外にも指摘されている。また、そもそも薬物療法などの“物質”療法においても、医療者と患者間のコミュニケーション、すなわち“情報の相互伝達”を基盤として医療が行われる。このように、他の精神療法や、医療コミュニケーションにおける情報伝達と学習の効率化にハイパーソニックが寄与できる可能性への扉が開かれることになる。

If we are able to prove an augmentation effect, this will have impact on various fields. Effective psychotherapy is not limited to CBT. Pharmacotherapy is also based upon the communication or reciprocal exchange of information between physicians and patients. This study may open the door to the potential contribution of the hyper-sonic effect on information exchange in medical communication and learning efficiency.

また、本研究は先端的電子情報技術というわが国の強みを最大限に活用したアプローチをとる。音響療法を含む情報技術を応用した統合医療という大きな未来性を持つ学術・産業領域を、わが国先導の下に世界に提案するとともに、電子情報通信産業やメディア産業など、異分野から医療分野への効果的で摩擦の少ない参入を促すことが期待される。

This study adopts an approach that fully utilizes advanced electronic information technology, which is one of Japan’s strong points. This study takes the initiative of promoting integrative medicine utilizing information technology including sound therapy as highly promising academic and industrial endeavors. Furthermore, this study is expected to effectively promote the integration of the electronic information technology and media industries with the medical field.

5. 研究の方法及び期間

5. Research methods and timeline

（1）研究実施期間

（1）Research timeline

倫理委員会承認後から 2020 年 3 月 31 日まで

（研究対象者登録締切予定日：2019 年 5 月 31 日）

（倫理委員会承認後から 12 月にかけて、3 症例に対して、本研究のフローに従った予備試行を行う。その上で、必要に応じて変更申請を行う。）

From the date of IRB approval to 31st March, 2020

(Planned date of the final registration of participant, 31st May, 2020)

(After the date of IRB approval, we will conduct three pilot trial cases that is followed by procedure of the main trial to confirm the feasibility. If needed, we will submit the modified protocol to IRB.)

（2）研究の種類・デザイン

（2）Trial design

個人割り付け、治療介入、探索的、無作為化、マスキング（治療者、患者）、並行群間比較、プラセボ対照、単施設、第 II 相試験

Individual-level allocated, treatment intervention, exploratory, randomized, therapist and patient masked, parallel group, placebo controlled, single-site, phase II trial

（3）予定する研究対象者数

（3）Targeted sample size

本研究は先行研究が存在せず、事前に症例数設計で用いる適切な効果量を推定することが困難である。本研究で狙いとする認知行動療法の増強効果という点で参考になる文献として、不安症に対する認知行動療法の d-cycloserine による増強効果のメタアナリシスが報告されている。この報告では、諸種のアウトカムでの増強効果の基準化効果量が 0.07〜0.58 であった（Ori et al., 2015）。この範囲の中でも、本研究の増強効果では比較的小さい効果サイズを想定するのが妥当であると考えられる。そこで、臨床的意義のある最小限の基準化効果量を 0.2〜0.3 と設定し、パイロット RCT の精度に基づく症例数設計手順(Cocks & Torgerson，2013)から、1 群 20 例とした。これにうつ病に対する認知行動療法の脱落率 12.1%を考慮して、本研究では 2 群で計 44 例を目標症例数として設定した。なお、この症例数には、予備試行で行われる 3 症例を含めない。

平成 30 年 1 月－平成 31 年 5 月までの 17 ヶ月間に、毎月 2–3 例程度の登録を予定している。

It is difficult to estimate the appropriate sample size to be used as there are no previous studies directly related to this trial. It would be informative to refer to the results of a meta-analysis of the augmentation effect of d-cycloserine on CBT for anxiety disorders. It was reported that the standardized effect size of the augmentation effect on various outcomes ranged from 0.07–0.58 (Ori et al., 2015). Based on this range, we conservatively selected a relatively low effect size for the augmentation effect of inaudible high-frequency sound on the efficacy of CBT. Hence, we set the standardized effect size as 0.2–0.3 as the clinically significant minimum level. Following the procedure of sample size estimation for pilot randomized controlled trial (Cocks & Torgerson, 2013), we set the sample size per group as 20. Considering the reported proportion of drop-outs (12.1%), we set 44 as the targeted total sample size. Before starting this main pilot trial, we will conduct an external preliminary trial with three patients to examine the feasibility of this trial.

We estimate a registration of two to three patients per month from January 2018 to May 2020.

（4）研究のアウトライン

（4）Research outline

本研究への参加は計 19 週間であり、組入れ期間が-4–0 週、介入期間が 1–8 週、介入後評価が 9 週、追跡評価が 21 週に実施される。タイムラインを Fig.1（次ページ）に示す。この間、患者は通常の診療を続ける。本研究に関係する部分は、Fig.1 にあるように、説明と同意、評価、登録、介入 8 セッション、である。

Participation in this study is for 19 weeks: enrolment period from -4 to 0-week, intervention period from 1 to 8-week, post-intervention assessment at 9-week, and follow-up assessment at 21-week. The timeline is depicted in Fig. 1. During the study period, all patients will continue treatment as usual. Activities related to this trial are informed consent, assessment, registration, and 8 session intervention (Fig. 1).

本臨床試験の広報は、認知行動療法センターのホームページおよび病院内に設置したパンフレットを通して行う。国立精神・神経医療研究センター病院に外来通院する患者を対象として、患者本人の参加希望があれば、主治医と相談してもらうよう広報する。主治医の許可が得られれば、主治医から精神リハビリテーション部の認知行動療法初診に紹介していただくようにする。認知行動療法初診後に、臨床心理室で開かれているカンファレンスにおいて、本研究への紹介が妥当と判断されれば、本研究への紹介となり、患者とコンタクトをとり、説明と同意へと進む。

This clinical trial will be advertised on the National Center of Cognitive-Behavior Therapy and Research website and with pamphlets placed in the hospital. This study is intended to recruit outpatients in the NCNP hospital. If patients are willing to participate in the trial, we encourage the

patient to first consult their main doctors. After permission has been obtained from their doctors, they will be referred to the intake for CBT at the department of psychiatric rehabilitation in the NCNP hospital. After the intake, treatment indications, including referral to this study, will be examined at weekly meetings attended by staff including psychiatrists and clinical psychologists in the department. If the patient is subsequently referred to this study, the research coordinator will contact the patients to proceed with informed consent.

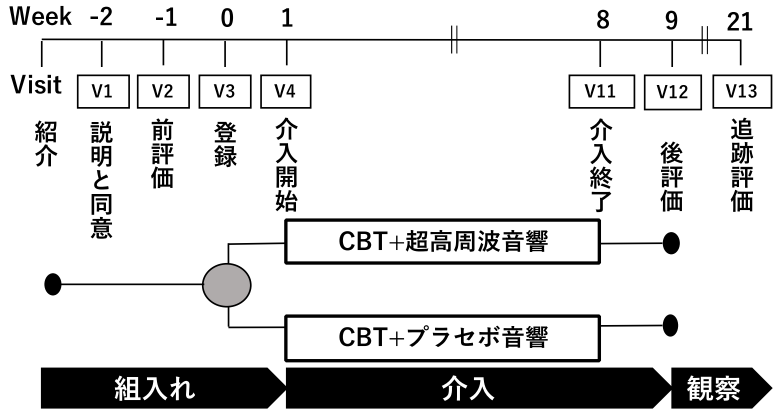

Fig.1 試験のタイムライン

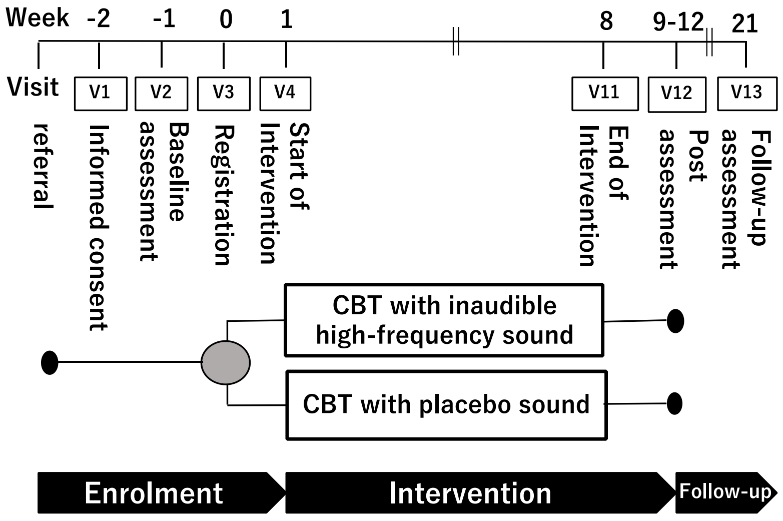

Fig.1 Timeline of the trial

（5）研究に用いる医薬品・医療機器、治療法等の情報

（5）Medicinal drugs, devises, or interventions for this study

アンヘドニアに対するポジティブ価システムに焦点を当てた認知行動療法(**Positive-valence system focused Cognitive-Behavioral Therapy for Anhedonia; PoCot**)：

ポジティブ価システムに焦点を当てたアンヘドニアに対する認知行動療法(**Positive-valence system focused Cognitive-Behavioral Therapy for Anhedonia; PoCot**)は、うつに対する認知行動療法の中でも行動活性化で用いられる技法を土台としつつ、近年のポジティブ価システムに介入する内容（Craske et al., 2016, Taylor et al., 2016, Alexopoulos et al., 2016, Blom et al., 2016）を参考に開発したものである。理論的な背景としては、報酬系に関する神経科学(Pizzagalli, 2014)、精神病理学(Pelizza & Ferrari, 2009; Franken et al., 2007)、そして感情調整研究（Carl et al., 2013）を基盤としている。基本的な治療原理は、うつ病という病態が報酬系情報システムが機能していない状態であるという理解のもとに、報酬系に関わるシステムが活性化されるような体験に従事することで、報酬系システムとそれが関与するうつという病態が改善されるという仮定に立っている。治療内容は下記の(6)に示す通りである。

Positive valence system-focused Cognitive-Behavioral Therapy for Anhedonia; PoCot：

The PoCot is developed based on the behavioral activation for depression and recently proposed interventions for positive valence system (Craske et al., 2016; Taylor et al., 2016; Alexopoulos et al., 2016; Blom et al., 2016). Theoretically, this program is based on the neuroscience of the reward system (Pizzagalli, 2014), psychopathology underlying anhedonia (Pelizza & Ferrari, 2009; Franken et al., 2007), and emotional regulation (Carl et al., 2013). The central treatment rationale is based on the concept of depression as a dysfunction of the reward-related information system (i.e., positive valence system). It is assumed that reward-related systems may be improved by engaging rewarding behaviors and fully experiencing positive emotions.

超高周波音響とプラセボ音響：

超高周波音響は、人の可聴域上限をこえる超高周波成分を豊富に含む熱帯雨林の自然環境音を用いる。プラセボ音響は、超高周波音響と同一の音源から高周波成分のみを取り除いた音響を用いる。音響提示装置には研究参加者自身では操作できない安全装置（操作パネルカバーなど）を装備し、装置の制御は実験スタッフが行う。音響情報の種類については、これまでの健常人を対象とした一連の実験室実験や、市街地で多数の健常者を対象とした実験、外来うつ病患者を対象とした実験などで使用実績があり、問題が生じていない音源を、本研究の目的のために特別に編集して用いる。音響情報の呈示音量は、これまでの実験同様、室内の会話の支障にならない低音量とし、その最適音量を検討する。

Inaudible high-frequency sound and placebo sound:

We will use natural environmental sounds that have been proven to include many inaudible

high-frequency sounds for auditory intervention. We will exclude the inaudible high-frequency sound component from the same natural environmental sound source, and use this as placebo sound. The operational panel of sound equipment will be concealed to prevent participants from touching and/or manipulating it. Operation of the sound equipment will be conducted by the therapist. The sound source has been used in laboratory experiments for healthy populations, large scale experiments in urban districts, and experiments for outpatients with depressive disorder. No adverse events or related problems were reported in these studies. Similar to previous studies, the volume of sound will be low to avoid disturbing the natural conversation in the room.

（6）試験薬の用法・用量、投与方法又は試験機器の適用方法

（6）Dosage and administration of trial medicine or method of trial device

アンヘドニアに対するポジティブ価システムに焦点を当てた認知行動療法：

対面での個人療法の形式をとった、ポジティブ価システムを強化する認知行動療法を行う。毎週 60 分、計 8 回のセッションを、国立精神・神経医療研究センターの音響装置が設定された部屋にて行う。60 分のセッションを毎週、計 8 回、個人療法として、訓練を受けた臨床心理士や医師が実施する。治療内容としては、初期のセッションでは、ポジティブ価システムが大切な理由を患者が理解できるように心理教育を行う。その上で、ポジティブな感覚や感情を５感で味わうための訓練や、ポジティブな出来事に注意を向けるためのモニタリングを開始する。さらには、ポジティブな感覚や感情を感じることを妨害する認知を同定し、代わりとなる認知を検討する。このようにして、日々の生活体験の中でポジティブな体験を重ねるとともに、本人にとってのポジティブな側面（強みや趣味）を同定し、それを意識したエクササイズを行う。このようにして、短期的なポジティブ体験（短期的報酬）への感受性と高めた上で、長期的な目標に向けた行動に従事するモジュールにとり組む。アンヘドニアの状態では、長期報酬への割引効果（※時間や労力のかかる目標は価値を置かれにくく行動が生起にくくなる）について心理教育を行った上で、本人にとっての長期的目標や価値を同定する。その上で、本人の長期目標のために取りうる行動の段階に分けて検討し、日々の生活において実施しやすい行動から取り組む。さらに、人間関係におけるポジティブ体験を強化するために、日々の対人的に感謝できる出来事を記録するとともに、コンプリメントを与え受けるという課題に取り組む。最終セッションでは、介入を通して学習した取り組みを継続するための方法を確認し、終結となる。

Positive Valence System-focused Cognitive-Behavioral Therapy for Anhedonia

The PoCot will be conducted in a face-to-face, individual format. Sixty-minute weekly sessions will be conducted in a room with sound equipment in the National Center of Neurology and Psychiatry. A trained clinical psychologist or physician will administer the PoCot. The earlier PoCot sessions will include psychoeducation emphasizing the importance of the positive valence system to recover from depression. The participant will then begin to practice savoring positive sensations and emotions using the five senses, and in monitoring the enhancement of attention to positive events in daily life. Participants will be requested to identify thoughts that interrupt the mindful acceptance of positive sensations and emotions, and to try to examine alternative thoughts. These practices are intended to promote accumulation of positive experiences in daily life. In parallel, the participant is gradually encouraged to identify and exercise their own positive aspects (e.g., strength and avocation). The focus of this program gradually shifts from enhancing the acceptability of short-term positive experiences (short-term reward) to engaging longer-term, goal-directed behavior. Psychoeducation on temporal discounting of reward in anhedonia will be provided. The participant will be asked to identify longer-term goals or values in their life. The goal will be divided into smaller steps and patients encouraged to engage in easier behaviors. Furthermore, patients will be asked to monitor events for which they are grateful in daily life, and to practice receiving and providing compliments to enhance positive interpersonal experiences. In the final session, participants will review the skills learned in the program and examine the ways of maintaining such activities.

超高周波音響とプラセボ音響呈示のための音源と音響機器：

超高周波音響は、人の可聴域上限をこえる超高周波成分を豊富に含む熱帯雨林の自然環境音を用いる。プラセボ音響は、超高周波音響と同一の音源から高周波成分のみを取り除いた音響を用いる。音響提示装置には研究参加者自身では操作できない安全装置（操作パネルカバーなど）を装備し、装置の制御は実験スタッフが行う。音響情報の種類については、これまでの健常人を対象とした一連の実験室実験や、市街地で多数の健常者を対象とした実験、外来うつ病患者を対象とした実験などで使用実績があり、問題が生じていない音源を、本研究の目的のために特別に編集して用いる。音響情報の呈示音量は、これまでの実験同様、室内の会話の支障にならない低音量とし、その最適音量を検討する。

Sound material and sound presentation system for inaudible high-frequency sound and placebo sound:

Rain forest sounds, a natural sound source containing the richest amount of high frequencies

with a conspicuously fluctuating structure, were chosen as the sound source for the experiments. As placebo sound, only the inaudible HFCs were excluded from the original sound material. The sound equipment system will be covered so as not to be able to operate by participant. Experimental staff will regulate the sound presentation equipment. The contents of sound has been used previous study in city environment for healthy population and in experiments with individual with depression without any problem. Sound volume will be small enough to be able to have a conversation in the therapy room.

用量・スケジュール変更規準

Criteria for changes in dose or frequency

本研究では短期の介入を取るために、全8セッションへの参加を被験者に求める。止むを得ずセッションのキャンセルが生じた場合には、遅滞なく日程の再調整を行う。

As short-term intervention will be employed, all participants will be requested to participate in every eight session. If participants have to cancel a scheduled session, an alternative date will be scheduled immediately.

（7）併用薬・併用療法についての規定

（7）Regulation for concurrent drugs or treatments

向精神薬の併用がある場合には、介入期間中には薬剤の種類と用量を一定にするよう、本人及び主治医の同意を予め得ることとする。他に、電気けいれん療法と構造化された精神療法は研究期間には実施しないよう依頼する。

If the participant takes any type of psychotropic medicine, we will obtain consent from the participant and their primary doctors to keep the dose and types of medicine consistent throughout the study period. In addition, we will request that participants refrain from receiving electroconvulsive therapy and/or other systematic psychotherapy during the study period.

（8）評価項目、評価方法

（8）Outcomes and assessment method

主要評価項目は、１週から 9 週にかけて毎週 Snaith–Hamilton Pleasure Scale（SHAPS）で測定されるアンヘドニア症状である。

副次評価項目は、9 週に測定される GRID Hamilton Depression Rating Scale 17 Item（GRID-HAMD）で測定されるうつ症状である。

The primary outcome is anhedonia symptoms assessed by the Snaith-Hamilton Pleasure Scale from week 1 to 9.

The secondary outcome is depression symptoms assessed by the GRID-Hamilton Depression Rating Scale 17 Item administered at week 9.

（19） 観察及び検査項目

(9) Observation or examination items

観察・検査・調査項目

Observation, examination, or survey items

23. 基礎情報：性別、生年月日、婚姻状況、年収、就学・就労状況、病名（主治医による診断）、主訴、登録以前までの治療歴（心理相談含む）、精神科既往歴、教育歴、家族歴、飲酒・喫煙
24. 宿題遵守：治療の一環として宿題の実施遵守を評価する。
25. 併用治療の遵守状況：Visit 毎に患者に確認するとともに、可能な場合はカルテ記載を随時確認することにより、薬物療法の安定性を確認する。
26. アンヘドニア症状：SHAPS 日本語版、SHAPS-C
27. うつ症状の重症度：GRID-HAMD, BDI-II
28. 精神疾患の有無：MINI（除外基準、併存疾患の確認）
29. 他のアウトカム指標：PANAS, SWLS, PWB
30. 治療メカニズム：EROS, EEfRT
31. 有害事象の確認：Visit 毎に口頭および所定の用紙にて確認する。
32. 治療プロセス：HC
33. 盲検化：IEKNO

23. Basic information: Gender, date of birth, marital status, income, current education and/or employment, diagnosis by primary doctor, chief complaints, treatment history, psychiatric history, educational history, family history, alcohol use, smoking.
24. Homework compliance: Homework compliance will be assessed as part of treatment.
25. Compliance for concurrent treatment: Consistency of pharmacotherapy will be checked at every visit. Where possible, the research coordinator will check it via electronic medical charts.
26. Anhedonia: Japanese version of SHAPS, SHAPS-C
27. Depression: GRID-HAMD, BDI-II
28. Presence or absence of mental disorders: MINI (confirmation of exclusion criteria and concurrent disorders)

29. Other outcomes: PANAS, SWLS, PWB
30. Treatment mechanism: EROS, EEfRT
31. Adverse events: Will be checked at every visit by oral questioning and a self-report sheet.
32. Treatment process: HC
33. Masking: IEKNO

観察・検査・調査スケジュール

Schedule for observation, examination, or survey

					ENROLMENT			INTERVENTION								POST	FU
TIME POINT (Week)					-2	-1	0	1–11								12	21
Visit					V1	V2	V3	V4	V5	V6	V7	V8	V9	V10	V11	V12	V13
(Burden for patient (minutes))																	
**ENROLLMENT :**																	
	Informed Concent			(50)	X												
	Randomization			(5)			X										
	Intake			(60)			X										
**ASSESSMENTS :**																	
	Diagnosis	MINI	IE	(25)		X											
Primary Outcome	Anhedonia	SHAPS	Pt	(4)		X		X	X	X	X	X	X	X	X	X	X
Secondary Outcome	Anhedonia	SHAPS-C	IE	(10)		X										X	X
Other Outcome	Depression/Anhedonia	GRID-HAMD	IE	(30)		X										X	X
		BDI-II	Pt	(5)			X					X				X	X
	Emotion	PANAS	Pt	(3)			X	X	X	X	X	X	X	X	X	X	X
	Life Satisfaction	SWLS	Pt	(2)			X					X				X	X
	Well-being	PWB	Pt	(6)			X					X				X	X
Treatment Mechanism	Anhedonia	EROS	Pt	(3)			X					X				X	X
		EEfRT	Pt	(20)		X										X	X
Safety	Adverse Event	AE	Th	-				X	X	X	X	X	X	X	X	X	X
Adherence	Homework Compliance	HC	Th	-					X	X	X	X	X	X	X		
Blinding		IEKNO	Pt/Th/IE	(2)				X								X	X
Burden for Participants(minutes)					(50)	(89)	(84)	(9)	(7)	(7)	(7)	(23)	(7)	(7)	(7)	(83)	(83)

Fig 2. Overall description of the measures and its timepoints.

IE: Interview with Independent Evaluator, Pt: Participant self-report, Th: Therapist self-report

上記の項目は調査スケジュール表（Fig.2）に基づき、試験施設にて検査する。調査結果を CRF に記載する。規定された観察日（検査日）のずれの許容範囲は±7 日とする。

The items above will be assessed at the trial site following the Examination Schedule Table. Results of the examination will be filled in CRF. An acceptable range of dates is one week before and after the scheduled date.

（20） 実施する検査について

(10) Assessment

7. 精神疾患の診断（Mini International Neuropsychiatric Interview 7.0.0）：

   MINI は、DSM-5 の主要な精神疾患や臨床状態を診断するための簡易構造化面接法である(Sheehan et al., 1998)。本研究では、DSM-5 診断を評価するために最新版の MINI 7.0.0 を使用する。評価項目は、抑うつエピソード、うつ病/大うつ病性障害、自殺念慮、自傷及び自殺行動、自殺行動障害、躁病エピソード、軽躁病エピソード、双極 I 型障害、双極 II 型障害、双極性障害、特定不能のもの、精神病性の特徴を伴う双極 I 型障害、パニック症/パニック障害、広場恐怖症、社交不安症/社会不安障害（社交恐怖）、強迫症/強迫性障害、心的外傷後ストレス障害、アルコール使用障害、物質使用障害（非アルコール）、精神病性障害、精神病性の特徴を伴う気分障害、神経性やせ症/神経性無食欲症、神経性過食症/神経性大食症、過食性障害、全般性不安症/全般性不安障害、医学的、器質的および薬物関連の病因を除外、反社会性パーソナリティ障害であり、各診断基準について「はい」「いいえ」で回答する。短時間で施行可能である（18.7±11.6 分、中央値 15 分）。旧版については、日本版についても信頼性と妥当性が確認されている(Otsubo et al., 2005)。妥当性については、Structured Clinical Interview for Diagnostic and Statistical Manual-III-R-patient version との基準関連妥当性が示されており（Kappa > .49）、信頼性については高い評者間一致度が報告されている（Kappa > .72）。

23. Diagnostic status of mental disorders (Mini International Neuropsychiatric Interview 7.0.0):

   The MINI is a brief structured interview for assessing major mental disorders and clinical status (Sheehan et al., 1998). We will use the updated 7.0.0 version. The MINI assesses the presence or absence of major depressive episodes, major depressive disorder, suicidality, suicide behavior disorder, manic episode, hypomanic episode, bipolar I disorder, bipolar II disorder, other specified bipolar and related disorder, panic disorder, agoraphobia, social anxiety disorder, obsessive-compulsive disorder, posttraumatic stress disorder, alcohol use disorder, substance use disorder (non-alcohol), any psychotic disorder, major depressive disorder with psychotic features, bipolar I disorder with psychotic features, anorexia nervosa, bulimia nervosa, binge-eating disorder, generalized anxiety disorder, and antisocial personality disorder. Medical, organic, or drug causes will be ruled out. Each diagnostic item is answered by YES/NO. Earlier versions of MINI have been demonstrated to be administrated in a short

period of time (mean 18.7 ± minutes, median 15 minutes). The reliability and validity of the Japanese translation of the earlier version of MINI has been demonstrated (Otsubo et al., 2005). In terms of validity, criterion-related validity with Structured Clinical Interview for Diagnostic and Statistical Manual-III-R-patient version has been reported (Kappa > .49). In terms of reliability, sufficient inter-rater concordance has been reported (Kappa > .72).

24. アンヘドニア（Snaith–Hamilton Pleasure Scale; SHAPS）：

SHAPS は、アンヘドニアの有無およびその重症度を測定するための自己記入式尺度である（Nagayama et al., 2012; Snaith et al., 1995）。14 項目から成り、1 から 4 の 4 件法で回答するが、評定肢は項目によって異なる（たとえば、項目 1「好きなテレビ番組やラジオ番組を楽しめますか？」に対する評定肢は「1. 少しも楽しくない」「2. 楽しくない」「3. 楽しい」「4. とても楽しい」）。得点が高いほど、アンヘドニアの重症度が高いことを示す。SHAPS 得点の合計が 20 点以上の場合、アンヘドニア症状を有するとみなされる（e.g. ClinicalTrials.gov; 臨床試験 NCT02874534, NCT02494050）。日本語版の信頼性に関しては、十分な値（α=.90）が報告されている（Nagayama et al., 2012）。回答時間 4 分程度。

8. Anhedonia（Snaith–Hamilton Pleasure Scale, SHAPS; Snaith–Hamilton Pleasure Scale-clinician administered, SHAPS-C）：

The SHAPS is a self-report questionnaire for assessing the presence/absence of anhedonia and its severity (Nagayama et al., 2012; Snaith et al., 1995). It consists of 14 items with 4-point Likert anchors. Each item is answered by different anchors. For example, Item 1 “I would enjoy my favourite television or radio programme” is answered as follows: strongly disagree, disagree, agree, or strongly agree. A higher score means more severe anhedonia symptoms. A total score higher than 20 is interpreted as the presence of anhedonia (e.g. ClinicalTrials.gov: NCT02874534, NCT02494050). Reliability of the Japanese version of SHAPS has been demonstrated (alpha = .90; Nagayama et al., 2012). The average time to answer is 4 minutes.

9. 抑うつ症状（GRID Hamilton Depression Rating Scale 17 Item; GRID-HAMD）：

ハミルトンうつ病評価尺度（Hamilton Depression Rating Scale; HAMD）は、抑うつ症状の重症度を測定するための評価尺度である（Hamilton, 1960）。本研究では、その改良版である GRID-HAMD17 項目版を用いる。GRID-HAMD は半構造化面接の形式をとり、評価者によって実施される。評価対象となる期間は、過去 1 週間である。評価項目は、抑うつ症状に関する 17 項目から成る。各項目の評点は、程度（5 段階評定）と頻度（4 段階評定）をそれぞれ評価した上で、縦軸に程度、横軸に頻度をとったグリッド上に表現される基準に従って決定される（たとえば、項目 1「抑うつ気分」では、程度が

「軽度」で頻度が「ほとんど常に」であれば 2 点という評点が与えられる）。合計得点は 17 項目すべての評点を合算して算出され、0-52 点の範囲を取る。高ければ高いほど、抑うつ症状が重症であることを示す。本尺度の信頼性に関しては、日本語版に関して優れた評定者間一致度（intraclass correlation coefficient; ICC = 0.95-0.99: Tabuse et al., 2007）が報告されている。また、内的一貫性に関しては原版において十分な値（α = 0.78）が示されている（Williams et al., 2008）。並存的妥当性に関しては、HAMD の別版である Structured Interview Guide for HAMD（SIGH-D）の各項目および合計得点と高い相関が報告されている（Williams et al., 2008）。施行時間 25 分程度。

25. Depression（GRID Hamilton Depression Rating Scale 17 Item, GRID-HAMD）：

Hamilton Depression Rating Scale (HAMD) is an interview for assessing the severity of depression (Hamilton, 1960). We will use the improved version of the original HAMD, which is called GRID-HAMD 17 item version. GRID-HAMD is conducted by the interviewer using a semi-structured format. The assessed period is during the week before the date of assessment. Each item is assessed in terms of severity (5-point Likert, vertical axis) and frequency (4-point Likert, horizontal axis). For example, item 1 “depressive mood” could be rated as 2 if the severity is “mild” and frequency is “almost always”. The total score ranges from 0 to 52. A higher score means more severe depression. In terms of reliability of the Japanese version, high inter-rater concordance has been reported (intraclass correlation coefficient; ICC = .95–.99, Tabuse et al., 2007). Sufficient internal consistency has been reported (alpha = .78) (Williams et al., 2008). In terms of concurrent validity, high correlations between GRID-HAMD and Structured Interview Guide for HAMD（SIGH-D）have been reported (Williams et al., 2008). It takes approximately 25 minutes to complete this interview.

26. 抑うつ症状（Beck Depression Inventory-II; BDI-II）：

BDI-II は、過去 2 週間の抑うつ症状を測定するための自己記入式尺度である（Beck, Steer, & Brown, 1996; 小嶋・古川, 2003）。21 項目から成る。各項目は、0 から 3 の 4 件法で回答されるが、評定肢は項目によって異なる。得点範囲は 0–63 点であり、得点が高いほど抑うつ症状が重症であることを示す。日本語版の信頼性に関しては、一般青年・成人を対象とした研究で十分な値（α=.87）が報告されている（Kojima, Furukawa, Takahashi, Kawai, Nagaya, & Tokudome, 2002）。また、日本語版の並存的妥当性に関しては、CES-D と有意な中程度の正の相関（r = .69）が報告されている（Kojima et al, 2002）。回答時間 5 分程度。

4. Depression（Beck Depression Inventory-II; BDI-II）：

BDI-II is a self-report scale for assessing depression over the past two weeks (Beck et al., 1996; Kojima & Furukawa, 2003). It consists of 21 items with a 4-point Likert scale (0–3). The anchor varies from item to item. The score range is 0 to 63. A higher score indicates more

severe depression. In terms of reliability of the Japanese version, sufficient internal consistency has been reported (alpha =.87) (Kojima et al., 2002). As for concurrent validity, the Japanese version of BDI-II was moderately correlated with CES-D (r = .69) (Kojima et al., 2002). It takes approximately 5 minutes to complete the scale.

27. ポジティブ・ネガティブ感情（The Positive and Negative Affect Schedule; PANAS）：

PANAS は、日々の感情体験を測定するための自己記入式尺度である（Clark & Watson, 1988; Clark & Watson, 1989）。22 項目から成る。各項目は、「1. ほとんど、または全くあてはまらない」から「5. 非常にあてはまる」の 5 件法で回答される。ネガティブな感情とポジティブな感情についての 2 つの下位尺度がある。各下位尺度は 11 項目で構成され、11-55 点という得点範囲をとる。得点が高いほど、ネガティブな感情またはポジティブな感情をより多く体験していることを示す。PANAS は様々なタイムフレームで用いられることがあるが、本研究では「過去 1 週間」というタイムフレームを採用する。回答時間 5 分程度。

5. Positive and Negative emotion (The Positive and Negative Affect Schedule, PANAS):

PANAS is a self-report measure for assessing daily emotional experiences (Clark & Watson, 1988; Clark & Watson, 1989). It consists of 22 items. Each item is rated on a 5-point Likert scale from 0 (very slightly or not at all) to 5 (extremely). There are two sub-scales: positive affect and negative affect. Each subscale consists of 11 items. The score range for the subscales is 11 to 55. A higher score means more frequent experience of positive and negative affect. PANAS can be used at various time frames. We use the time frame of “past one week.” It takes approximately 5 minutes to answer this scale.

28. 人生満足度（Satisfaction With Life Scale; SWLS）：

SWLS は、人生に対する満足の程度を測定するための自己記入式尺度である（Diener et al., 1985; 角野、1994）。5 項目から成る。各項目は、「1. 全くそうではない」から「7. 全くそうだ」の 7 件法で回答される。得点が高いほど、人生に対する満足度が高いことを示す。日本語版の信頼性に関しては、十分な値（α= .84〜.90）が報告されている（角野, 1994）。また、日本語版の妥当性に関しては、自尊心尺度との間に有意な中程度の正の相関（r = .59）および Y-G 性格検査の D 尺度（抑うつ性）と N 尺度（神経質）との間に有意な中程度の負の相関（D 尺度: r = -.50; N 尺度: r = -.34）が報告されている（角野, 1994）。回答時間 1 分程度。

6. Satisfaction with life（Satisfaction With Life Scale, SWLS）：

SWLS is a self-report measure for assessing the degree of life satisfaction (Diener et al., 1985; Sumino, 1994). It consists of five items. Each item is answered using a 7-point Likert scale from “1. Strongly disagree” to “7. Strongly agree.” A higher score indicates higher

satisfaction with life. The Japanese version is reported to have high internal consistency (alpha = .84–.90) (Sumino, 1994). In terms of the validity of the Japanese version, a moderate positive correlation with self-esteem (r = .59) and a moderate negative correlation with the Depression and Neuroticism scale in the Y-G personality inventory has been reported (r = -.50, -.34) (Sumino, 1994). It takes approximately 1 minute to answer this scale.

29. 心理的 well-being（Psychological Well-being Inventory; PWB）：

PWB は、心理的 well-being を測定するための自己記入式尺度である（Kitamura, Kishida, Katayama, Matsuoka, Miura, & Yamabe, 2004; Ryff, 1989）。人格的成長，人生における目的，自律性，環境制御力，自己受容，積極的な他者関係の 6 因子、計 18 項目で構成されている。各項目は「1. 全く当てはまらない」から「6. 大変当てはまる」の 6 件法で回答される。人格的成長は，発達と可能性の連続上にいて，新しい経験に向けて開かれている感覚を表す。人生における目的は，人生における目的と方向性の感覚を表す。自律性は，自己決定し，独立，内的に行動を調整できるという感覚を表す。自己受容は，自己に対する積極的な感覚を表す。環境制御力は，複雑な周囲の環境を統制できる有能さの感覚を表す。積極的な対人関係は，温かく，信頼できる他者関係を築いているという感覚を表す。回答時間 4 分程度。

11. Psychological well-being（Psychological Well-being Inventory, PWB）：

PWB is a self-report measure for assessing psychological well-being (Kitamura et al., 2004; Ryff, 1989). This 18-item scale has six subscales: purpose in life, autonomy, environmental mastery, personal growth, positive relations with others, and self-acceptance. Each item is rated using a 6-point Likert scale from “1. Completely disagree” to “6. Completely agree.” The definition of each sub-scale is described by Ryff (2014). Purpose in life is the feeling of meaning, purpose, and direction in one’s life. Autonomy is the feeling that one’s life is in accordance with one’s own personal convictions. Personal growth is the feeling that one is making use of one’s own personal talents and potential. Environmental mastery is the feeling of how well one manages life situations. Self-acceptance is the knowledge and acceptance one has of oneself, including awareness of personal limitations. Positive relations with others is a feeling of depth of connections one has in ties with significant others. It takes approximately 4 minutes to answer.

12. 環境内の報酬知覚（Environmental Reward Observation Scale; EROS）：

EROS は、行動に随伴する正の強化について主観的に評価する尺度である。10 項目からなり、4 件法（1-4 点）で回答する。得点範囲は 10-40 点であり、得点が高いほど、主観的に多くの報酬を知覚していることを表す。様々な population において信頼性および妥当性が報告されている（Armento & Hopko, 2007; 国里・高垣・岡島・中島・石

川・金井・岡本・坂野・山脇、2011）。日本人サンプルを対象としたデータにおいては、Cronbach’s α=.78、再検査信頼性は *r* =.75 と高い信頼性が報告されている（国里他、2011）。また、妥当性に関しては、抑うつ・不安症状（BDI-II, CES-D, STAI）との中程度から強い負の相関（順に *r* = -.55, -.57, -.65）が報告されている（国里他、2011）。回答時間 2 分程度。

30. Environmental Reward Observation (Environmental Reward Observation Scale, EROS):

EROS is a self-report measure for assessing the subjective feeling of positive reinforcement contingent with behavior. In total, 10 items are answered using 4-point Likert anchors (1 to 4). The score ranges from 10 to 40. A higher score means a more subjective observation of reward. Its reliability and validity has been reported in multiple populations (Armento & Hopko, 2007; Kunisato et al., 2011). High reliability has been reported in the results of the Japanese population (alpha = .78, test-retest correlation = .75) (Kunisato et al., 2011). In terms of validity, EROS was moderately to strongly correlated with depression and anxiety (*r* = -.55, -.57, -.65 with BDI-II, CES-D, STAI) (Kunisato et al., 2011). It takes approximately 2 minutes to answer this scale.

31. 報酬の行動評価(Effort-Expenditure for Rewards Task; EEfRT)：

EEfRT は、報酬の獲得可能性に関する予測的価値およびエフォートに基づいた意思決定の程度を測定するための行動課題であり、コンピュータを用いて実施される（Treadway, Buckholtz, Schwartzman, Lambert, & Zald, 2009）。主な手順は以下の通りである；（１）報酬獲得のための課題として「易しい課題」と「難しい課題」を選択する画面が 5 秒間呈示される。（２）「易しい課題」では、7 秒以内に 30 回のボタン押し（利き手使用）が求められる。（３）「難しい課題」では、21 秒以内に 100 回のボタン押し（非利き手使用）が求められる。（４）それぞれの課題を達成すると決められた報酬を得ることができる。具体的には、「易しい課題」では一律 1 ドル、「難しい課題」では 1.24 ドルから 4.30 ドルの幅のある報酬が設定される。つまり、低コスト/低報酬条件、高コスト/低報酬条件、高コスト/高報酬条件の 3 条件が設定される。ただし、研究参加者は毎回の試行で報酬を必ず得られるわけではなく、3 種類の確率（12%, 50%, 88%）のいずれかで報酬を得ることができる。（５）研究参加者は、各試行終了後に報酬を得られるかどうかのフィードバックを受ける。（６）研究参加者は、これまでの一連の行程を 1 試行とし、20 分間の制限時間の間にできだけ多く行う。施行時間 20 分程度。

9. Behavioral assessment for reward (Effort-Expenditure for Rewards Task, EEfRT)：

EEfRT is a computerized behavioral task for assessing reward expectancy and effort-based decision making (Treadway et al., 2009). The procedure of this task is as follows: 1) 5-second

display for selecting an easy or difficult task to obtain the reward, 2) easy task requires participants to push a key 30 times in 7 seconds using their dominant hand, 3) difficult task requires participants to push a key 100 time in 21 seconds using their non-dominant hand, 4) participants are eligible for a reward if they succeed in the task. The reward for the easy task is one dollar. The reward for the difficult task ranges from 1.24 to 4.30 dollars. As such, there are three conditions: low effort and low reward, high effort and low reward, and high effort and high reward. However, participants may not obtain a reward in every trial. The probability of receiving a reward is set at 12%, 50%, or 88%, 5) participants who succeed in the trial notice whether they get the reward immediately after each trial, 6) participants continue trials for 20 minutes. It takes 20 minutes to complete this task.

32. 宿題遵守（Homework Compliance Scale; HCS）：

HCS は、患者が宿題をどの程度遂行したかについて治療者が評価する尺度である（Primakoff, Epstein, & Covi, 1986）。1 項目からなり、毎セッションの終了時に 0-6 点で評価する。筆者らは、原著者の許可および協力を得て、バックトランスレーションのプロセスを経て日本語に翻訳した。

10. Homework compliance（Homework Compliance Scale, HCS）：

HCS is a therapist rating scale for assessing the degree of homework completion (Primakoff et al., 1986) It consists of one item. Therapist rates 0 to 6 points after each session. We translated this scale into Japanese via rigorous back-translation procedures with permission from the author of original version.

33. マスキングの評価（Independent Evaluator Knowledge of Outcome; IEKNO）：

本尺度は、パニック障害に対する大規模な臨床研究(Barlow, Gorman, Shear, & Woods, 2000)や、統一プロトコルの RCT（Farchione et al., 2012）において利用された尺度である(Roll et al., 2004)。3 項目からなり、患者がどちらの群に割り付けられたと考えるか、それについてどの程度の自信を持っているか 0 から 8 の Likert で回答を求める。そして、最後の項目では、割付を見抜いたと考えられる場合には、どのような情報からそう考えられるかを尋ねる。本研究では、Blinding Index(Bang et al., 2004)を算出するために、割付の推測について"介入群"、"対照群"、"わからない"の 3 つから選択する回答方式とする。

11. Masking (Independent Evaluator Knowledge of Outcome, IEKNO):

This scale has been used in a large scale clinical trial for panic disorder (Barlow, Gorman, Shear, & Woods, 2000) and randomized controlled trial for unified protocol （Farchione et al., 2012). It consists of three items: judgement of allocated groups, confidence, and source of the

judgement. In this study, we used the anchors “intervention group”, “comparison group”, and “I don’t know” to calculate the blinding index (Bang et al., 2004).

（21） 症例登録、割付の方法

(11) Method of registration and randomization

症例登録

Registration of participants

割付は、インフォームド・コンセントを経て評価面接により全ての選択・除外基準を確認した上で、研究スタッフとは独立した専用の担当者が、NCNP 開発の EDC システムによりオンライン上で行う（中央登録による割付の隠蔵）。順番の作成はコンピュータにより行われる。

After obtaining informed consent and confirming eligibility by an assessment interview, research staff specifically for this role will register the participant using EDC system developed by NCNP. Sequences will be generated by this EDC system.

割付方法と割付調整因子

Method of randomization and stratification

ブロックランダム化を行い、調整因子は設定しない。

We will use block randomization without any stratification.

割付表の保管と開錠手続き

Storage of randomization table and procedure for unblinding

割付表は割付担当者が保管する。重篤な有害事象が発生するなど、研究責任者が必要と認めた場合には、研究責任者が担当者に開錠を依頼する。

Personnel for allocation will store the randomization table. Whenever the primary investigator acknowledges the need for unblinding such as in the occurrence of a severe adverse event, the primary investigator will request the personnel for anonymization to open the key.

（22） 統計解析方法

(12) Statistical analysis

解析の概要

Summary of statistical analysis plan

データマネジャーがデータの固定と解析を行う。全ての主要・副次評価項目の解析において、介入群と対照群を比較する。全ての解析で、P 値が小数点 3 までの値で表現され、0.001 以下のものは $p < .001$ と記載する。解析ソフトは、SPSS、SAS、R を用

いる予定である。統計的検定を用いる解析では、両側検定として有意水準を 5%とする。本研究は主要評価項目の解析のためにデザインされているため、副次評価およびその他の解析（サブグループ解析・調整解析）は探索的なものとなる。

The data manager will fix and analyze the data. We will compare the intervention and comparison groups in primary and secondary analyses. The *p*-value will be expressed to three decimal places. If the *p*-value is under .001, it will be described as $p < .001$. We will use statistical software SPSS, SAS, and/or R. When we apply statistical tests, we will set statistical significance for 5% of a two-tailed test. As this study is designed to test the primary outcome, analyses for secondary and other outcomes, as well as for other purposes (sub-group analysis, adjusted analysis, etc.) will be exploratory.

主要評価項目の解析

Analysis of primary outcome

評価項目：1 週から 12 週にかけての SHAPS 日本語版得点

従属変数を 1–11 週までの 8 ビジット時点、および 12 週時点の評価項目とし、独立変数の固定効果要因を割付（介入群=0 vs 対照群=1）と測定時点（visit 1=1, visit 2=2, visit 3=3, visit 4=4, visit 5=5, visit 6=6, visit 7=7, visit 8=8, week 12=9)、両要因の交互作用項(割付け*測定時点)、および評価項目の pre 値（-1 週）とし、変量効果要因を反復測定した被験者として、線形混合モデルにより検討する。

Outcome: Score of the Japanese version of SHAPS assessed nine times from 1 to 12 weeks.

Liner mixed model will be conducted to analyze the primary outcome. The dependent variable is participants' SHAPS scores assessed at eight visit points from week 1 to 11 and week 12. Fixed-effects are allocation (intervention group = 0 vs. comparison group = 1), visit (visit 1=1, visit 2=2, visit 3=3, visit 4=4, visit 5=5, visit 6=6, visit 7=7, visit 8=8, week 12=9), and allocation-by-visit interaction, and SHAPS at pre-intervention (week -1). The random variable will be participant.

副次評価項目の解析

Analysis of secondary outcome

評価項目：12 週時点の SHAPS-C

ベースラインを共変量とした共分散分析を行う。

Outcome: Score of SHAPS-C at week 12

As with the primary analysis, linear mixed model will be conducted. In the model, the time point will be pre and post only.

他の評価項目の解析

Analysis of other outcomes

評価項目：12 週時点の BDI-II, PANAS, SWLS, PWB, EROS, EEfRT

主要評価項目と同様に、線形混合モデルによる検討する。ただし測定時点は、pre, week 5, post となる。

Outcome: BDI-II, PANAS, SWLS, PWB, EROS, EEfRT at weeks 5 and 12

LMM analysis will be conducted as same as primary analysis. In the model, the time point will be pre, week 5, and post.

欠測値の処理

Handling of missing data and sensitivity analysis

混合モデルによって解析することで欠損値に対処する。さらに、“best”または”worst”のそれぞれのケースシナリオを仮定した感度分析を実施する。

We use mixed model to treat missing data. Furthermore, we will conduct sensitivity analyses by assuming both “best” and “worst” case scenarios.

上記に定めていない症例やデータの取り扱いは、研究責任者と解析責任者で協議、決定する。

Other statistical analyses or data handling will be discussed and judged by the primary investigator and study statistician.

解析対象集団

Analysis populations

主要・副次評価項目についての解析はすべて、Intent-To-Treat 原則に基づき登録されて全参加者を対象として実施する。副次的な解析対象集団として、研究登録された後に中止症例に該当しなかった全例を当研究計画書に適合した対象集団（Per Protocol Set, PPS=Completer case）として、評価項目についての解析を行う。

Analyses for the primary and secondary outcome analysis will be conducted on Intent-To-Treat principle, which all registered participant are subject to analysis. As the secondary analysis set, we will conduct the outcome analysis by using the all registered participant who will not meet the discontinuing criteria as Per Protocol Set.

（23） 試料・情報の授受

(13) Provision or receipt of specimens or information

本研究は単施設で実施するため、外部との情報の授受はない。

We will not exchange any specimens or information as this is a single site study.

6. 研究対象者の選定方針

6. Enrolment criteria of research participants

（1）選択基準

（1）Inclusion criteria

7） アンヘドニア症状を有すること（SHAPS の 2 値化得点の合計が 3 点以上）

8） うつ症状が軽症（GRID-HAMD の 8 点）以上

9） 18 歳以上

(1) Anhedonia symptoms (Snaith-Hamilton Pleasure Scale score >= 3 by dichotomous calculation)

(2) Depressive symptoms are mild or severe (GRID Hamilton depression rating scale >= 8)

(3) Aged 18 years or older

【各選択基準の設定理由】

1) 有効性評価のため

2) 有効性評価のため

3) 有効性評価のため（研究対象者を SHAPS 日本語版の妥当性が検証されている成人に限定するため）

【Reason for each inclusion criteria】

7) For efficacy evaluation

8) For efficacy evaluation

9) For efficacy evaluation (Japanese version of SHAPS has not been examined for reliability and validity among the younger Japanese population)

（2）除外基準

(2) Exclusion criteria

13） 統合失調症および類縁疾患（MINI にて評価）

14） 双極性障害（MINI にて評価）

15） 物質使用障害（MINI にて評価）

16） 重篤な自殺念慮（MINI にて評価）

17） 治療に支障のある身体疾患や認知機能障害

18） その他に、認知行動療法を遂行する上で障害となる問題のある者

13） No schizophrenia and other psychotic disorders at baseline assessed by the Mini-International Neuropsychiatric Interview (MINI)

14） No bipolar disorder at baseline assessed by MINI

15） No substance use disorders at baseline assessed by MINI

16） No serious suicidal ideation at baseline assessed by MINI

17） No severe or unstable physical disorders or major cognitive deficits at baseline

18） Other problems that may be serious obstacles for conducting CBT

【各除外基準の設定理由】

1) 有効性評価のため

2) 安全性のため

3) 安全性のため

4) 安全性のため

5) 有効性評価のため

6) 安全性及び有効性評価のため

【Reason for each exclusion criteria】

13) For efficacy evaluation

14) For safety

15) For safety

16) For safety

17) For efficacy evaluation

18) For efficacy evaluation and safety

7. インフォームド・コンセント等を受ける手続等

7. Procedure for obtaining informed consent, etc.

倫理委員会で承認の得られた同意説明文書を研究対象者（代諾者が必要な場合は代諾者を含む、以下同じ）に渡し、文書及び口頭による十分な説明を行い、研究対象者の自由意思による同意を文書で取得する。研究対象者の同意に影響を及ぼす情報が得られたときや、研究対象者の同意に影響を及ぼすような研究計画書等の変更が行われるときは、速やかに研究対象者に情報提供し、研究に参加するか否かについて研究対象者の意思を予め確認するとともに、事前に倫理委員会の承認を得て同意説明文書等の改訂を行い、研究対象者の再同意を得ることとする。本研究では、有効なインフォームドコンセントを与えることができると判断された 18 歳以上の未成年者を対象に加える。それらの者を加える理由は、世界的には、成人として 18 歳以上を対象として研究されることが一般的であるためである。

Research coordinators will present the IRB approved informed consent briefing paper to the participants (including to legally acceptable representatives, if applicable), orally explain the research sufficiently using the briefing paper, and obtain consent that is reflected by the participant’s free will in written document. If any changes occur in the research protocol or the primary investigator acknowledges information that may influence participants’ consent, the primary investigator will

immediately provide such information to participants and confirm continued participation in this study. The primary investigator will also revise the informed consent briefing paper and re-obtain participants consent using the revised paper. Subjects in this study are those older than 18 years old with sufficient capacity to provide informed consent. This is because in this type of study around the world, 18 years of age or older is sufficient to be regarded as “adult”; in Japan, 20 years of age or older is considered “adult” `.

41. 同意説明文書の概略、研究により生じる知的財産権の帰属
42. 研究の名称、研究実施について所属機関の長の許可を受けている旨
43. 研究の目的及び意義
44. 研究対象者として選定された理由（研究対象となる方）、研究の方法及び期間、参加協力事項
45. 研究対象者に生じる負担並びに予測されるリスク及び利益
46. 研究が実施又は継続されることに同意した場合であっても随時これを撤回できる旨、研究が実施又は継続されることに同意しないこと又は同意を撤回することによって研究対象者等が不利益な取扱いを受けない旨
47. 研究に関する情報公開の方法
48. 研究対象者等の求めに応じて、他の研究対象者等の個人情報等の保護及び当該研究の独創性の確保に支障がない範囲内で研究計画書及び研究の方法に関する資料を入手又は閲覧できる旨並びにその入手又は閲覧の方法
49. 個人情報等の取り扱い（匿名化の方法を含む）、情報の保管
50. 情報の廃棄の方法、研究終了後のデータ取扱の方針
51. 研究の資金源、利益相反
52. 研究対象者等及びその関係者からの相談等への対応
53. 経済的負担及び謝礼の内容
54. 研究で用いられる治療方法以外の治療方法の内容
55. 研究終了後の医療の提供の有無とその内容
56. 研究対象者に関わる偶発的所見への対応
57. 健康被害に対する補償の有無及びその内容
58. 研究終了後のデータの二次利用の可能性とその対応方法
59. 研究機関の名称及び研究責任者の氏名
60. 問い合わせ先

41. Outline of the informed consent briefing paper, ownership of intellectual property
42. Title of the research and information on approval given by the chief executive of the research implementing entity concerning its implementation
43. Objectives and significance of the research

44. Reasons for enrolment in the research, method, and time period of the research
45. Potential burdens on research subjects and predictable risks and benefits
46. Condition that research subjects may withdraw their consent at any time even after they have given consent that the research commences or continues. Condition that the refusal or withdrawal of consent by a research subject regarding commencement or continual of the research does not cause any disadvantage to them
47. Means to make information on the research public
48. The fact that research subjects can request and obtain or read the research protocol and documents concerning method of the research, to the extent that it does not interfere with the protection of personal information, etc. of other research subjects or the originality of the research, as well as the procedure to obtain or read such protocols and documents
49. Handling of personal information, etc. (including process of anonymization and when anonymization is conducted), means for storage information
50. Means for disposal of information
51. Status of research-related conflicts of interest of the research implementing entity, such as research fund resources, as well as research-related conflicts of interest of each investigator such as his/her individual income
52. Response to consultation, etc. made by research subjects and other individuals concerned
53. When the research involves any financial expenditure on or remuneration for the research subject; a statement to that effect and details of such
54. When the research involves any medical technique beyond usual medical practice, description of alternative procedure(s) or course(s) of treatment
55. When the research involves any medical technique beyond usual medical practice, response related to the healthcare delivery to the research subjects after the research
56. When any significant finding concerning the subject’s health or generic characteristics which may be inherited by his/her offspring, etc. may be obtained through implementing the research, handling of the research results related to the research subject (including incidental findings)
57. When the research involves any invasiveness, whether compensation will be offered for research-related injury and details of such compensation
58. With respect to specimens and information acquired from the research subject, when any of those may be utilized or provided to other research implementing entities for research in the future that is not identified at the time of obtaining consent from the research subject; a statement to that effect and the contents of utilization assumed at the time of obtaining consent;
59. Names of the research implementing entity and the principal investigator
60. Contact information

8. 試料・情報、個人情報等の取扱い（匿名化する場合の方法、個人情報の安全管理方法など）

8. Handling of specimens, information, and personal information (method of anonymization, handling of personal information, etc.);

個人情報管理者（田島美幸）は、同意書など個人情報を含む研究必須文書等の紙媒体や電子媒体、個人情報と研究用 ID の対応表について、漏洩、盗難、紛失しないように以下の通り管理・保存する。

Administrator of the management of personal information (i.e., Miyuki Tajima, Ph.D.) will manage and store all research-related printed or electronic records with personal information (e.g., informed consent documents), correspondence table of personal information and research ID number to prevent any information being divulged, stolen, or lost. Details of the management and storage are as follows.

物理的安全管理

Physical security management

物理的安全管理措置は、あらゆる紙資料や電子データ（同意書、対照表、録音のデータを含む）の取り扱いを想定している。

Physical security management concerns any printed or electronic data including signed informed consent documents, correspondence table, recorded audio or video, etc.

9. 個人データを取り扱う区域の管理：個人データは国立精神・神経医療研究センター内において、患者から直接取得される（病院内での症状評価面接や自己記入式尺度への回答、PC を用いて行われる認知課題のデータ、認知行動療法セッションの録音）。この個人データは、国立精神・神経医療研究センター７号館３階の認知行動療法センター内の居室のキャビネットの中に保管する。個人データを保管し扱う区域は国立精神・神経医療研究センター７号館３階の認知行動療法センター内とする。認知行動療法センターへの入室はカードキーで管理されており、カードキーはセンターに雇用されている者のみが保有する。カードの所持状況は、認知行動療法センター長が帳簿を作り管理する。居室の鍵は、NCNP 全体の鍵管理の方針に従い、使用外の時間は NCNP 防災センターにて保管される。

3. Restriction area for handling the personal information

All personal information will be obtained from participants in the National Center of Neurology and Psychiatry (e.g., answering the psychiatric symptoms interview or self-report scales in the hospital, cognitive task using PC, and recorded audio date of the CBT session). These data will be stored in the cabinet in the National Center for Cognitive-Behavior Therapy and Research located on the 3rd floor of 7th building in the

National Center of Neurology and Psychiatry. Entry to the National Center for Cognitive-Behavior Therapy and Research is access restricted. Only center staff are permitted entry. The director of the center is responsible for the management of the card key and maintaining the information of the possession of card keys. Keys for each office room will be returned to the disaster prevention center at the end of every day.

10. 機器及び電子媒体等の盗難等の防止：盗難防止のために、上記①の管理に加えて、キャビネットもそれぞれ施錠可能なものを用いる。さらに、キャビネットの鍵は、パスワード認証が必要なキーボックスに保管する。キーボックスは、特殊な方法を用いなければ脱着できないような強度で壁に固定する。

2. Prevention of theft of devices and electronic media

To prevent theft, we will use a lockable cabinet. Furthermore, we will use the key box with password authentication. This key box will be placed on the wall firmly so as not to easily removed.

11. 電子媒体等を持ち運ぶ場合の漏えい等の防止：電子媒体は管理区域内のみで利用する。

3. Prevention of divulgation during electronic media usage

Electronic media will be used in the restricted area

12. 個人データの削除及び機器、電子媒体等の廃棄：匿名化された検査データなどの紙資料は、すべてただちに個人を特定するような情報（固有名詞など）を削除したかたちで保管する。これらの検査データなどと、ただちに個人が特定できる情報を含む研究データ（同意書、対応表、録音データ）は研究終了後 5 年後に個人情報をわからなくして、復元不可能な形で廃棄する。

4. Disposal of personal information and electronic devices and media

Printed data such as self-report scales will be immediately anonymized by deleting any identifying information (e.g., specific names etc.) and securely stored. Five years after the completion of the study, these anonymized data and other personal identifying data (signed informed consent documents, correspondence table, and recorded audio files) will be disposed of to avoid restoration.

技術的安全管理：

Technical Security Management:

技術的安全管理は電子化されたデータ（録音のデータを含む）の安全管理措置を想定しており、下記のような措置をとる。

Technical security management concerns the handling of electronic data including audio and video data.

9. アクセス制御：研究代表者の伊藤正哉、コーディネート担当の横山知加、宮前光宏のみが録音データが保管された HDD にアクセスできる。研究遂行上の必要に応じて、研究代表者が認める者に一時的にアクセスを許可することがある（データ入力やモニタリングなど）が、その場合にはアクセス制御を許可された者の監督下での扱いとする。電子データは強制暗号化とパスワード認証が求められる HDD を用いてデータを保管する。

1. Access restriction: Only the primary investigator (MI) and coordinators (CY, MM) are able to access the HDD where the audio data are stored. If necessary, the primary investigator may temporarily allow another person to access the data (e.g., for data entry and monitoring). In that case, the person has to treat the data under the monitoring of MI, CY, or MM. All electronic data will be stored in the encrypted HDD with password protection.

10. アクセス者の識別と認証：上記①に定めた者のみが HDD に保管されたデータにアクセスでき、認証パスワードを知ることができるようにする。

2. Identification and authentication of accessible personnel:
Only MI, CY, and MM can access the HDD and have the password.

11. 外部からの不正アクセス等の防止：録音データの再生においては、インターネットに接続されない端末もしくは機器を用いる。

3. Prevention of unauthorized access: When handling the data such as playing the recorded audio files, we will use the devices offline while not connected to the Internet.

12. 情報システムの使用に伴う漏えい等の防止：電子データは外部ネットワークにつながった状態の機器には接続しない。

4. Prevention of divulgation in the process of using electronic information system:
We will not connect any electronic data to outside networks.

人的安全管理：

Human security management:

5. 雇用契約及び委託契約の締結時における守秘義務規定：当研究は『平成 22 年規程第４０号保有する個人情報の保護に関する規程』が適用される NCNP 職員もしくは研究生によって実施される。

1. Confidentiality obligation in the employment and consignment contracts: All study staff in this study are subject to the policy for protection of personal information (NCNP policy No. 44, 2010).

6. 研究者等に対する教育・訓練の実施：手順書の徹底を図るよう訓練を実施するとともに、NCNP で実施される『平成 22 年規程第４０号保有する個人情報の保護に関する規程』第８条にて規定されている研修をはじめ、コンプライアンス研修、倫理研修に参加する。

2. Education and training for the researchers:

All study staff are subject to training following the standard procedure operations for this study. They will participate in the workshop described in article 8 in the NCNP policy for protection of personal information (NCNP policy No. 44, 2010) and other workshops for compliance and research ethics.

9. 研究参加のリスクと研究がもたらすベネフィット

9. Risks and benefits of research participation

（1）研究対象者に生じる負担

(1) Burdens on research subjects

本研究によって、研究の実施に伴って確定的に生じる事象としては、全 12 週間にわたる毎週の通院と評価と介入治療にかかる時間と労力の消費であるが、これらがすべからく研究対象者にとって好ましくない事象として知覚・認識されるとは限らない。

Burdens for the participants will be time and effort for the 12-week intervention and symptom evaluation. Perceptions of these burdens will vary between participants.

（2）研究対象者に生じ得る不利益（有害事象不具合等）

(2) Potential disadvantages for research subjects (e.g., adverse events)

本研究によって生じ得る不利益は想定されないが、自身の人生や生活を振り返って、今までしてきたのとは違う取り組みを行う中で、一時的にネガティブな気分を体験することはあり得る。

We do not expect any disadvantages to participants for participating in this research. However, participants may temporarily experience a negative mood in response to reflecting on their past life and practicing new behaviors.

（3）リスクを最小化する方法

(3) Strategy to minimize risks

健康被害が生じた場合、適切な医療等の対応が行われるように図る。本研究では、研究に伴う健康被害のリスクは、一般の外来診療に伴うもの以上は特にないものと考えられるため、健康被害に対する医療も通常の診療と同様に、被験者の保険診療内で行う。

If any health damage occurs, the primary investigator will arrange an appropriate medical response. As we assume that the risk of health damage is not likely to occur as is usual in outpatient care, we will use participants’ medical insurance for the medical expenses of any health damage, as used in usual-care.

（4）予想されるベネフィットと（1）（2）（3）を踏まえた総合評価

(4) Comprehensive assessment of burdens, risks, and benefits

本研究の参加者は、本研究で有効性を検証しようとしている症状を有する方であり、本研究に参加し介入を受けることで、直接的に本人の症状が改善する可能性がある。明確に想定される不利益は、評価と介入に要する労力と時間である。万が一に不利益が生じた場合には、適切な医療等の対応が行われるように図る。これらを総合評価し、本人に利益が生じる可能性があるだけでなく、研究成果により将来の医療の進歩に貢献できる可能性がある。その具体的意義については 1 に記載した通りである。

We developed the intervention and are going to test its efficacy specifically for anhedonia symptoms. We hypothesize that there are potential benefits for participants for symptom improvement by participating in this research. Clear disadvantages for the participants will be the effort and time required for symptom evaluation and treatment. If by any chance other disadvantages should occur, the primary investigator will arrange an appropriate medical response. By comprehensively assessing these aspects, this study has potential benefits for the participants and the study results may promote the progress of future medicine. The medical significance of the study has already been discussed in section 1 of this protocol.

（5）個々の研究対象者における中止基準

（5）Criteria for discontinuing interventions for a given trial participant

研究中止時の対応

Correspondence when the discontinuing occurs

個々の症例が以下のいずれかの中止基準に該当する場合、その症例の試験治療を中止する。中止の日付・時期(治療期間・追跡調査期間)、中止の理由、経過をカルテならびに CRF に明記するとともに、中止時点で主要評価項目と副次評価項目に関する評価を行い有効性・安全性の評価を行う。有害事象発生により中止した場合は、研究責任者は担当治療者、スーパーバイザー、主治医、NCNP 担当医と状況を検討し、介入中止になった理由に応じて適切な対応を決定し、可能なかぎり原状に回復するまでフォローする。中止後も可能なかぎり 12 週時点での評価を行う。

If a participant meets either of the following discontinuing criteria, the participant will terminate participation. The trial coordinator will record the date and period (intervention or follow-up period) of discontinuation, its reason, and the course in CRF. Whenever possible, participants will be requested to participate in primary and secondary outcome evaluations at the time of discontinuation. If the discontinuation occurred due to an adverse event, the primary investigator will discuss with the therapist, supervisor, primary doctor, and responsive doctor at

NCNP about the possible response to the patients, and monitor patients until they recover to the degree of baseline. Whenever possible, the trial coordinator will ask patients to participate in the outcome evaluation at week 12.

中止基準

Criteria for discontinuation of participation in the trial

11） 被験者から試験参加の辞退の申し出や同意の撤回があった場合
12） 被験者との連絡が不通となった場合
13） 重篤な有害事象により試験治療の継続が困難な場合
14） 試験全体が中止された場合
15） その他の理由により、研究責任者が試験を中止することが適当と判断した場合

11） Participant requests discontinuation or withdraws consent
12） Unable to contact the participant
13） Difficulty continuing participation due to severe adverse events
14） Discontinuation of the whole trial
15） Primary investigator’s judgement of discontinuation to be appropriate for any other reason

3） 研究全体の中止基準

3） Discontinuation of trial

7） 倫理委員会により中止の勧告あるいは指示があった場合は、試験を中止する。研究責任者および効果安全性評価委員で構成する委員会は、以下の事項に該当する場合は試験実施継続の可否を検討する。
8） 治療の品質、安全性、有効性に関する重大な情報が得られたとき。
9） 倫理委員会により実施計画等の変更の指示があり、これを受入れることが困難と判断されたとき。試験の中止または中断を決定した時は、速やかに倫理委員会にその理由とともに文書で報告する。試験中止の決定を行った後、関係医師および心理士等に速やかに伝達し、中止後の処理にあたるものとする。
7） If the IRB advises or orders trial discontinuation, this trial will be stopped. The committee consisting of the primary investigator and members of DSMB will examine the appropriateness of continuing the trial when the following occurs.
8） Acknowledgement of important information regarding quality, safety, and efficacy of the intervention.

9） When the IRB orders modification of trial procedures and the primary investigator judges such modification to be difficult. If the primary investigator decides to stop or discontinue the trial, the primary investigator will report the reason immediately to IRB in written form. After deciding to discontinue the trial, the primary investigator will immediately notify the related doctors, psychologists, and other medical staff, and deal with the discontinuation.

10. 研究に用いられる情報に係る資料の保管及び廃棄の方法

10. Method for storage and disposal of information including records related to information utilized in research

個人情報管理者は、同意書など個人情報を含む研究必須文書等の紙媒体や電子媒体、個人情報と研究用 ID の対照表を、認知行動療法センターに設置した鍵のかかる保管庫にて漏洩、盗難、紛失しないように管理・保存する。紙媒体や電子媒体は、研究終了後から 5 年間経過後に、個人情報を分からなくしたことを確認した上で破棄される。なお、個人情報と研究用 ID の対照表の破棄後、連結不可能匿名化状態となった電子データベースは、追加解析や他の研究のため利用されることがある。この連結不可能匿名化データの電子ファイルは認知行動療法センター内の LAN やインターネットに接続されていないパソコンにてパスワードをかけて暗号化した上でハードディスクドライブに保存し、研究責任者の管理のもと保存される。なお、録音された記録の一部については、コンサルテーションや事例検討のために、セキュリティの確保されたクラウド上で共有されることがある。共有された録音データは、毎回のコンサルテーションや事例検討後に、確実にデータ消去を行う。

本試験のデータが他機関にて分析されたり、他の研究のために利用されたりする具体的な予定はないものの、その可能性は否定できない。本試験の匿名化されたデータの将来的な活用については、被験者に説明し、同意を得る。

A manager of private information will store the necessary documents or electronic records that contain personal information (such as documents for informed consent) and correspondence table for personal information and research ID in a locked cabinet to prevent any divulgation, theft, or loss. All documents and electronic records will be disposed of after the termination of research with erasure of personal information. After the disposal of correspondence table, the unlinked anonymized data may be used for additional analysis or other research purposes. The primary investigator will store the unlinked anonymized data in the encrypted HDD under password lock separated from any LAN or Internet. Parts of the recorded audio may be shared on a secure cloud system for case consultation. Such shared audio dates will be erased with absolute certainty after every consultation session.

Although we have no plans to transfer the data to other institutions or to use the data for other research purposes at this time, we cannot exclude these possibilities. We will obtain informed consent from the participants regarding the future utilization of the anonymized data.

11. 研究機関の長への報告内容及び方法

11. Matters to be reported to the chief executive of the research implementing entity and relevant procedures

研究機関の長（理事長）への報告については下記の通りとする。

Primary investigator will report to the chief executive as follows.

9）年 1 回、研究実施状況について報告し、研究継続の適否について倫理委員会の審査を受ける。

10）重篤な有害事象が発生した場合は、速やかに理事長に報告し、研究継続の適否について倫理委員会の審査を受ける。

11）介入法の有効性・安全性に関する重要な情報が得られた場合は、研究責任者の見解を記載し、理事長に報告し、研究継続の適否について倫理委員会の審査を受ける。

12）研究の終了時（中止または中断の場合を含む）には、理事長に報告する。

9）Primary investigator will report the progress of research once a year. The IRB will review it and examine the appropriateness of continuing the research.

10）If a severe adverse event occurs, the primary investigator will immediately report it to the chief executive. The IRB will review it and examine the appropriateness of continuing the research.

11）If the primary investigator acknowledges any important information regarding the efficacy and safety of the intervention, the primary investigator will report the event with assessment by the investigator. The IRB will review it and examine the appropriateness of continuing the research.

12）Primary investigator will report the termination or discontinuation of research.

12. 研究に係る資金と利益相反に関する状況

12. Status of research-related conflicts of interest of the research implementing entity, such as research fund resources, and research-related conflicts of interest of each investigator, such as his/her individual income

本研究は、“日本医療研究開発機構研究費　平成 29 年度「統合医療」に係る医療の質向上・科学的根拠収集研究事業　1-4 漢方及び鍼灸を除く各種療法に関する科学的知見を創出する

ための研究"に対して、課題名『超高周波音響療法による認知行動療法の増強効果』を資金源とし、研究代表者は堀越勝、研究期間は本倫理申請承認から平成 32 年 3 月 31 日である。また、行動指標をアウトカムとした附属研究については、パブリックヘルス財団　2017 年度ストレス科学分野「ストレスマネジメント」に対して、課題名『認知行動療法面接中における超高周波音響呈示のブースト効果研究：抑うつ症状の改善に焦点を当てて』を資金源とし、研究代表者は宮前光宏、研究期間は 2017 年４月１日から 2018 年 3 月 31 日で助成を受けている。また、本研究に係る全ての研究者及びその配偶者などの家族は、本研究で用いる超高周波音響装置を製造している業者との間に経済的利害関係、雇用関係は一切無い。従って、研究者が企業等とは独立して計画し実施するものであり、研究結果及び解析等に影響を及ぼすことは無い。本研究に携わる研究者等は、いずれも利益相反状態にないことを確認している。

Funding resources for this research are provided by the Japan Agency for Medical Research and Development as the Grant for Research Project for Improving Quality in Healthcare and Collecting Scientific Evidence on Integrative Medicine (2017). The approved title for the project was "Augmentation of cognitive behavior therapy by inaudible high-frequency sound therapy" awarded to Masaru Horikoshi. The planned study period is from the approval date of this research protocol by IRB to 31st of March 2020. The ancillary study for examining the augmentation effect of inaudible high-frequency sound of positive valence system-focused CBT on the performance of behavioral tasks is funded by the Public Health Research Foundation as the research grant for stress management in the field of stress science ("Research on the boosting effect of inaudible high-frequency sound exposure during the session of cognitive behavioral therapy: Focusing on the improvement of depression") awarded to Mitsuhiro Miyamae. The research period was from 1st of April 2017 to 31st of March 2018. None of the researchers involved in this project, or their family members, had any conflict of interest with the company that built the audio systems. Hence, this research will be conducted independently of any company or other entity, resulting in no influence on the results or analysis. All of the relevant researchers in this study confirmed that they had no conflict of interest.

13. 研究に関する情報公開の方法

13. Means to disclose research information

臨床試験登録

Registration in clinical trial registry

本臨床試験は、大学病院医療情報ネットワーク(UMIN)「臨床試験登録システム」に登録予定である。

This clinical trial will be registered in a public database operated by the National University Hospital Council of Japan.

成果の帰属と公表

Attribution and publication of research outcomes

本研究によっていかなる結果が得られても、その結果は被験者を特定できないようにした上で、学術専門誌などにて公表される。Primary Outcome Paper とプロトコル論文は伊藤が草稿を執筆し、共同主任研究者（堀越）がその内容を確認した上で、共著者の確認・修正を全著者が納得するまで繰り返した上で投稿する。二次解析などの他の論文は、Primary Outcome Paper が公表された後に公表することとする。行動指標をメインアウトカムとして、治療メカニズムを検証する論文に関しては、宮前が草稿を執筆し、共著者の確認・修正を全著者が納得するまで繰り返した上で投稿する。

Regardless of the results, the primary investigator will publish the results in anonymized format in the academic journal. MI will write the first draft for the primary outcome paper and protocol paper. After confirmation by the co-primary investigator (MH), the draft will be repeatedly modified until all authors have approved the final version. Other papers for secondary analysis will be published after acceptance of the primary outcome paper. For the behavioral task outcomes, MM will write a first draft and continually modify the paper until all co-authors have given approval.

14. 研究対象者等及びその関係者からの相談等への対応

14. Means to respond to inquiries made by the research subjects and/or other individuals concerned

研究対象者等及びその関係者からの相談については、下記相談窓口を説明文書に明示し、申し出があった場合には遅滞なく対応する。

The primary investigator will reveal the address and phone number of the inquiry counter on the document used for informed consent for any possible consultation by subjects or related individuals. If any consultation is raised, the primary investigator will respond immediately.

研究相談窓口

Inquiry counter

〒187-8551

東京都小平市小川東町四丁目 1 番 1 号

国立研究開発法人国立精神・神経医療研究センター 認知行動療法センター

電話番号 042-341-271２(内線 3605 または 3606)

対応時間：月・水・金（9 : 30−17 : 00）

氏名 伊藤正哉 所属・職名 認知行動療法センター 室長

Masaya Ito,

Ogawa higashi 4-1-1, Kodaira, Tokyo,

National Center of Neurology and Psychiatry, 187-8551
Phone: 042-341-2712 (Ex 3605, 3606)
Time: 9:30 to 17:00; Monday, Wednesday, and Friday

倫理委員会事務局への連絡先
Contact to Institution Review Board
〒187-8551
東京都小平市小川東町四丁目１番１号
国立研究開発国立精神・神経医療研究センター倫理委員会事務局
e-mail:rinri-jimu@ncnp.go.jp

Ogawa higashi 4-1-1, Kodaira, Tokyo,
National Center of Neurology and Psychiatry, 187-8551
Executive office of IRB
e-mail: rinri-jimu@ncnp.go.jp

15. 研究対象者等に経済的負担又は謝礼がある場合には、その旨及びその内容
15. Financial expenditure or remuneration for research subjects and associated details
本研究への参加により追加でご負担いただく費用、また、研究参加に対する謝礼はない。
There is no financial expenditure or remuneration for participation in this research.

32. 侵襲（軽微な侵襲を除く。）を伴う研究において重篤な有害事象が発生した際の対応
16. Means to respond in the case of serious adverse events (e.g., if the research involves invasiveness, not including minor invasiveness)
有害事象及び副作用の定義
Definition of adverse event or side effect
有害事象には、研究期間中に発生する有害と認められるあらゆる事象が含まれる。
An adverse event could be any adverse phenomenon during participation in this research.

有害事象の評価及び判定規準
Evaluation and criteria for adverse event
所定の項目（口渇、便秘、排尿障害、視力調節障害、起立性低血圧、眠気、倦怠感、不眠、不安・焦燥、落ち込み・意欲低下、食欲不振、体重増加、体重減少、性欲低下、

動悸、ふるえ、発汗、頭痛、ふらつき、その他）について、その有無を確認する。担当者は所定の用紙を用いて、「前回以来、身体的または精神的な症状で急に悪化したり発生しましたか」と口頭にて尋ね、患者の応答を求める（Solicit 形式であり、Voluntary 形式ではない）。

The presence or absence of adverse events will be assessed using the following items: dry mouth, astriction, dysuria, vision dysregulation (accommodation disturbance), orthostatic hypotension, sleepiness, fatigue, sleeplessness, anxiety/agitation, depression/anhedonia, lack of appetite, gain or loss of body weight, loss of sexual desire, palpitations, thrill, diaphoresis, headache, dizziness, other. Using forms, research staff will ask about any adverse event as follows, “Have you been experiencing any worsening or occurrence of physical or mental symptoms?”

有害事象の報告と発現時の対応

Response to adverse events

本研究では被験者の Visit ごとに、有害事象全体の評価を行う。有害事象が発現した場合、担当者はその内容と重篤性を評価する。担当者は出来る範囲で必要な対応を施し、研究責任者と相談の上で必要と判断され、被験者がうつ症状について何らかの治療を受けている場合には、NCNP での主治医または外部医療機関での主治医と相談し、必要な処置を施すよう求め、経過を充分観察することとする。担当者は有害事象の内容、発現日・消失日、程度、処置、転帰、重篤性評価、治療との関連性等を CRF に記載する。また、有害事象に対する治療が必要となった場合には、担当医または主治医と相談の上、担当者、担当医、または主治医が被験者にその旨を伝える。

We will evaluate the occurrence of any adverse event at every visit. In the case of an adverse event, research staff will evaluate the content and severity, and enact the necessary response. If the patient receives usual outpatient treatment in NCNP hospital or other medical institutes, the primary investigator will report the event to the primary doctors and request the necessary responses under their treatment. The primary investigator will continue the course after the occurrence of an adverse event. The research staff will record the content of the adverse event, occurrence date, date of improvement, severity, conducted response, course, and relevance to the intervention on the CRF. If additional treatment is required for the adverse event, the primary investigator, therapist, or primary doctor will provide an explanation.

予測される有害事象等

Expected adverse events

精神障害を対象とした治療において、もっとも注意が払うべき有害事象として、自殺念慮や企図が挙げられる。大規模な疫学調査によれば、大うつ病性障害における自殺企図は OR=3.2（95% CI 2.5–4.2）と報告されている(Nock et al., 2009)。その他に、上記に挙げた症状はうつ症状を有する者において予測される有害事象であると考えられる。
Generally, adverse events that should be taken into consideration are suicidal thoughts and attempts. According to the result of a large scale epidemiological survey, suicidal attempts among patients with major depressive disorder is OR=3.2 (95% CI 2.5–4.2) (Nock et al., 2009). Other expected adverse events are reflected in the items for adverse event.

33. 当該研究によって生じた健康被害に対する補償の有無及びその内容
17. Compensation for any harm caused by study participation

万が一、本研究の介入期間中に健康被害が生じた場合、適切な医療等の対応が行われるように図る。本研究では、研究に伴う健康被害のリスクは、一般の言語をコミュニケーション（面接、診療など）に伴うもの以上は特にないものと考えられるため、健康被害に対する医療も通常の診療と同様に、被験者の保険診療内で行う。なお、この本研究への参加に起因した健康被害が生じた場合、補償金、医療費・医療手当等の補償は行われないことを、あらかじめ被験者から同意を得るものとする。

If any harm occurs due to participation in this research, the primary investigator will respond to ensure the appropriate medical care or treatment. As we do not expect the risk of any harm beyond what typically occurs for face-to-face verbal communication (e.g., interview or examination), any medical care for harm will be conducted using medical insurance as is typically used for usual care. We will provide explanations and obtain consent from subjects about the lack of compensation for any harm during participation in this research.

34. 研究対象者への研究実施後における医療の提供に関する対応
18. Response related to healthcare delivery to research subjects following research

本試験参加終了後においても、本試験に関する疑問等を被験者が抱いた場合には、研究事務局への連絡ができるものとする。

If the subject raises any questions regarding this trial after completing participation, the subject will be able to contact the inquiry counter of this research.

35. 研究対象者に係る研究結果（偶発的所見を含む。）の取扱い
19. Means of handling subjects’ information (including incidental findings)

本研究において、偶発的所見が発見される可能性はほとんど存在しない。

We expect no incidental findings from participation in this trial.

36.　委託する業務内容及び委託先の監督方法

20. Content of the work to be entrusted and means of supervision over the contractors

本研究において、外部組織に業務を委託する予定はない。

No part of the work will be entrusted.

37.　試料及び情報の二次利用とそれに伴う他機関への提供の可能性

21. Possibility of secondary utilization and/or provision of the specimens and information acquired from subjects

本試験のデータが他機関にて分析されたり、他の研究のために利用されたりする具体的な予定はないものの、その可能性は否定できない。本試験の匿名化されたデータの将来的な活用については、被験者に説明し、同意を得る。

Although there is currently no plan for other institutions to analyze or utilize the data acquired by this trial, we cannot exclude this possibility. We will obtain informed consent from the subjects regarding the future utilization of anonymized data.

38.　モニタリング及び監査

22. Monitoring and audit

（1）モニタリング

(1) Monitoring

本試験では、研究責任者自身が、コーディネーター及びデータ管理者と連携して、施設モニタリング及び中央モニタリングを行い、その結果を効果安全性委員に報告する。基本的には、データ管理者が CRF 又は原資料とデータベースに入力されたデータの照合（施設モニタリング）を行う。中央モニタリングは解析担当者が行い、年に一度（10 月）研究責任者から効果安全性委員に報告書が送られる。中央モニタリングは全症例に対して行い、施設モニタリングは登録された最初の 3 例を対象に行い、その後、登録第 4 – 44 番目の症例に対して、予めランダム抽出しておいた 3 例を対象に行う。どの事例をランダム抽出したかは、CRF 作成者およびデータ入力担当者には伝えないようにする。研究責任者及び効果安全性委員会の委員が必要性を指摘した場合には、モニタリング委員会を開催する。また、効果安全性委員は、データ管理者へとデータ公開を求めることができる。データ取得と入力、モニタリングの流れは以下の通りである。

The primary investigator, in collaboration with the research coordinator and data manager, will conduct on-side and central monitoring, and report the results to the members of DSMB. The data manager will verify the entered data by referring to the CRF and/or primary source material (i.e., on-site monitoring). The statistician will conduct the central monitoring. The primary investigator will report the results to members of DSMB once a year (in October). All registered cases will be subject to central monitoring. On-site monitoring will be conducted for the first three registered cases, and then for three randomly pre-identified cases from the 4th to 44th registered cases. Developer of CRF and staff for entering data will not be masked to the pre-identified cases for on-site monitoring. A meeting for monitoring will be convened if the primary investigator or members of DSMB suggest the need. In addition, the members of DSMB may request data from the data manager. The procedure for data acquisition, entry, and monitoring is as follows.

15. 研究候補者に関係する First contact（紹介等）：コーディネーターが研究用 ID を付与
16. 同意取得後、ベースライン評価：評価結果をコーディネーターが CRF に記入
17. コーディネーター作成の CRF 及び自記式尺度（原資料）を研究スタッフがデータベースに入力
18. 担当者が基礎情報に関する CRF を記入
19. プロセス指標等、介入群の評価結果を研究スタッフがデータベースに入力
20. 中間・介入後評価結果をコーディネーターが CRF に入力
21. コーディネーターが記入した CRF 及び自記式尺度（原資料）を研究スタッフがデータベースに入力

※ 随時、データ管理者が CRF 及び自記式尺度と入力されたデータの照合（施設モニタリング）
※ 中央モニタリングは解析担当者が行う
※ 中央モニタリング毎に、研究責任者が効果安全性委員に報告書を送付

15. First contact with the participant (referral, etc.): Research coordinator gives research ID to the participant.
16. Baseline assessment after obtaining informed consent: Research coordinator records the assessment results of on the CRF.
17. Research staff will enter the data by referring to the CRF and self-report measures (primary source).
18. Therapist will fill in the CRF regarding the basic information.
19. Research staff will enter the results of process measures and other assessments.

20. Research coordinator will fill in the CRF regarding the results of mid- and post-assessment.
21. Research staff will enter the information on the CRF and self-report measures.
    - Data manager will check the accuracy of data entry from the CRF and self-report measures (i.e., on-site monitoring).
    - Statistician will conduct central monitoring.
    - Primary investigator will send the central monitoring report document to the members of DSMB.

（2）監査

(2) Audit

本試験についての監査は予定していない。

No audit is planned.

39. 用語の解説

23. Explanation of terms

アンヘドニア

Anhedonia

古典的には「歓びの喪失」として定義される状態を指す（Ribot, 1896）。ただし、近年では、この定義はアンヘドニアの一側面を表現しているだけに過ぎないという指摘があり、より多面的な構成概念であることが示唆されている。具体的には、アンヘドニアは研究領域基準におけるポジティブ価システムの失調として捉えることができ、報酬獲得に対する動機づけおよびエフォートの低減、報酬獲得時の快感情の低減および持続困難、報酬獲得に関する学習の困難などを含む状態と考えられる。

Anhedonia is classically defined as the “loss of pleasure (Ribot, 1896).” Recently, this definition has been criticized as it only partially describes anhedonia, and there are suggestions that that anhedonia comprises multiple aspects. Specifically, anhedonia may be regarded as the dysregulation of the positive valence system within the framework of Research Domain Criteria, which includes the decline of motivation and effort for reward acquisition, decrease of intensity and maintenance of positive emotion at the time of reward acquisition, and difficulty in reward learning.

認知行動療法

Cognitive behavioral therapy

人はうつや不安状態になると物事のとらえ方が、より悲観的・否定的になり、その結果気分や行動が影響されるという理解にもとづき、その人の認知のあり方を治療者との対話を通じて検討し、問題解決につながる方策を案出し、実施することにより、その人の気分の改善を図ることを目的とした、構造化された精神療法の１つ。

Cognitive behavioral therapy (CBT) is a form of structured psychotherapy. CBT is based on the concept that humans experiencing depressed mood or anxiety tend to think negatively, and such cognition affects behavior. In CBT, the therapist examines patients' cognition, elaborates, and tries ways to solve problems to improve the patients' mood.

24. 参考文献リスト、研究に関係する指針・ガイドライン

24. References and related guidelines

88. Alexopoulos, G. S., Raue, P. J., Gunning, F., Kiosses, D. N., Kanellopoulos, D., Pollari, C., Banerjee, S., & Arean, P. A. (2016). "Engage" therapy: behavioral activation and improvement of late-life major depression, *The American Journal of Geriatric Psychiatry*, **24**(4), 320-326.

89. Armento, M. E., & Hopko, D. R. (2007). The Environmental Reward Observation Scale (EROS): development, validity, and reliability, *Behavior Therapy*, **38**(2), 107-119.

90. Bang, H., Ni, L., & Davis, C. E. (2004). Assessment of blinding in clinical trials. *Controlled Clinical Trials*, **25**(2), 143-156.

91. Barlow, D. H., Gorman, J. M., Shear, M. K., & Woods, S. W. (2000). Cognitive-behavioral therapy, imipramine, or their combination for panic disorder: A randomized controlled trial. *JAMA*, **283**(19), 2529-2536.

92. Beck, A. T., Steer, R. A., & Brown, G. K. (1996). *Manual for the Beck Depression Inventory-II.* San Antonio, TX: Psychological Corporation. (小嶋雅代・古川壽亮（訳）(2003).　日本版 BDI-II—ベック抑うつ質問票—手引き　日本文化科学社).

93. Blom, E. H., Tymofiyeva, O., Chesney, M. A., Ho, T. C., Moran, P., Connolly, C. G., Duncan, L. G., Baldini, L., Weng, H. Y., Acree, M., Goldman, V., Hecht, F. M., & Yang, T. T. (2016). Feasibility and preliminary efficacy of a novel RDoC-based treatment program for adolescent depression: "Training for Awareness Resilience and Action" (TARA)—A pilot study. *Frontiers in Psychiatry*, **7,** 208.

94. Carl, J. R., Soskin, D. P., Kerns, C., & Barlow, D. H. (2013). Positive emotion regulation in emotional disorders: A theoretical review. *Clinical Psychology Review,* **33**, 343-360.

95. Clark, L. A., & Watson, D. (1988). Mood and the mundane: Relations between daily life events and self-reported mood. *Journal of Personality and Social Psychology*, **54,** 296-308.

96. Clark, L. A., & Watson, D. (1989). *The Japanese Positive and Negative Affect Schedule: Factor-based scales for the assessment of mood.* Unpublished manuscript. University of Iowa.

97. Craske, M. G., Meuret, A. E., Ritz, T., Treanor, M., & Dour, H. J. (2016). Treatment for anhedonia: A neuroscience driven approach. *Depression and Anxiety*, **33**, 927–938.

98. Cocks, K., & Torgerson, D. J. (2013). Sample size calculations for pilot randomized trials: a confidence interval approach. *Journal of Clinical Epidemiology*, **66**(2), 197-201.

99. Diener, E., Emmons, R.A., Larsen, R.J., & Griffin, S. (1985). The Satisfaction With Life Scale. *Journal of Personality Assessment*, **49**, 71-75.

100. Franken, I. H. A., Rassin, E., & Muris, P. (2007). The assessment of anhedonia in clinical and non-clinical populations: Further validation of the Snaith-Hamilton Pleasure Scale (SHAPS). *Journal of Affective Disorders*, **99**, 83-89.

101. Farchione, T. J., Fairholme, C. P., Ellard, K. K., Boisseau, C. L., Thompson-Hollands, J., Carl, J. R., Gallagher, M. W., & Barlow, D. H. (2012). Unified protocol for transdiagnostic treatment of emotional disorders: A randomized controlled trial. *Behavior Therapy*, **43**(3), 666–678.

102. Hamilton, M. (1960). A rating scale for depression. *Journal of Neurology, Neurosurgery & Psychiatry*, **23**, 56-62.

103. Harvey, A. G., Lee, J., Smith, R.L., Gumport, N.B., Hollon, S. D., Rabe-Hesketh, S., Hein, K., Dolsen, M. R., Hman, K. L., Kanady, J. C., Thompson, M.A., & Abrons, D. (2016). Improving outcome for mental disorders by enhancing memory for treatment. *Behaviour Research and Therapy*, **81**, 35-46.

104. Insel T, Cuthbert B, Garvey M, Heinssen R, Pine DS, Quinn K, Sanislow, C., & Wang, P. (2010). Research domain criteria (RDoC): toward a new classification framework for research on mental disorders. *American Journal of Psychiatry*, **167**, 748–751.

105. Kitamura, T., Kishida, Y., Katayama, R., Matsuoka, T., Miura, S. & Yamabe, K. (2003). Ryff's psychological well-being inventory: factorial structure and life history correlates among Japanese university students. *Psychological Reports*, **94**, 83-103.

106. Kojima, M., Furukawa, T. A., Takahashi, H., Kawai, M., Nagaya, T., & Tokudome, S. (2002). Cross-cultural validation of the Beck Depression Inventory-II in Japan. *Psychiatry Research*, **110**, 291-299.

107. 国里愛彦・高垣耕企・岡島義・中島俊・石川信一・金井嘉宏・岡本泰昌・坂野雄二・山脇成人. (2011). 日本語版 Environmental Reward Observation Scale (EROS) の作成と信頼性・妥当性の検討. 行動療法研究, **37**(1), 21-31.

108. 文部科学省・厚生労働省. (2014). 人を対象とする医学系研究に関する倫理指針.

109. 文部科学省・厚生労働省. (2015). 人を対象とする医学系研究に関する倫理指針　ガイダンス.

110. Nagayama, H., Kubo, S., Hatano, T., Hamada, S., Maeda, T., Hasegawa, T., Kadowaki, T., Terashi, H., Yoshioka, M., Nomoto, N., Kano, O., Inoue, M., Shimura, H., Takahashi, T., Uchiyama, T., Watanabe, H., Kaneko, S., Takahashi, T., & Baba, Y. (2012). Validity and reliability assessment of a Japanese version of the Snaith–Hamilton pleasure scale. *Internal Medicine.* **51,** 865–869.

111. National Collaborating Centre for Mental Health. (2010). Depression: the treatment and management of depression in adults. Updated ed. Leicester: British Psychological Society/London: Royal College of Psychiatrists.

112. Nock, M. K., Hwang, I., Sampson, N. A., & Kessler, R. C. (2009). Mental disorders, comorbidity and suicidal behavior: Results from the National Comorbidity Survey Replication. *Molecular Psychiatry*, **15**(8), 868–876.

113. Oohashi, T., Nishina, E., Honda, M., Yonekura, Y., Fuwamoto, Y., Kawai, N., Maekawa, T., Nakamura, S., Fukuyama, H., & Shibasaki, H. (2000). Inaudible high-frequency sounds affect brain activity: hypersonic effect. *Journal of Neurophysiology*, **83**, 3548-3558.

114. Ori, R., Amos, T., Bergman, H., Soares-Weiser, K., Ipser, J. C., & Stein, D. J. (2015). Augmentation of cognitive and behavioural therapies (CBT) with d-cycloserine for anxiety and related disorders. *Cochrane Database of Systematic Reviews*, **5**, Art. No.: CD007803. DOI: 10.1002/14651858.CD007803.pub2.

115. Otsubo, T., Tanaka, K., Koda, R., Shinoda, J., Sano, N., Tanaka, S., Aoyama, H., Mimura, M., Kamijima K. (2005). Reliability and validity of Japanese version of the mini-international neuropsychiatric interview. *Psychiatry and Clinical Neurosciences*, **59**(5), 517-526.

116. Pelizza, L., & Ferrari, A. (2009). Anhedonia in schizophrenia and major depression: state or trait? *Annals of General Psychiatry*, **8**(22), doi: 10.1186/1744-859X-8-22

117. Pizzagalli, D. A. (2014). Depression, stress, and anhedonia: toward a synthesis and integrated model. *Annual Review of Clinical Psychology*, **10,** 393–423.

118. Primakoff, L., Epstein, N., & Covi, L. (1986). Homework compliance: An uncontrolled variable in cognitive therapy outcome research. *Behavior Therapy*, **17**, 433–446.

119. Ribot T. (1896). La Psychologie des Sentiment [The Psychology of Feelings]. Paris: Felix Alcan.

120. Roll, D., Ray, S. E., Marcus, S. M., Passarelli, V., Money, R., Barlow, D. H., Wood, S. W., Shear, M K., & Gorman, J. M. (2004). Independent evaluator knowledge of treatment in a multicenter comparative treatment study of panic disorder. *Neuropsychopharmacology*, **29**(3), 612-618.

121. Ryff, C. D. (1989). Beyond Ponce de Leon and life satisfaction: new directions in quest of successful aging. *International Journal of Behavioral Development*, **12**, 35-55.

122. Snaith, R.P., Hamilton, M., Morley, S., Humayan, A., Hargreaves, D., & Trigwell, P. (1995). A scale for the assessment of hedonic tone the Snaith–Hamilton Pleasure Scale. *British Journal of Psychiatry*. **167**, 99–103.

123. Sheehan, D. V., Lecrubier, Y., Harnett-Sheehan, K., Amorim, P., Janavs, J., Weiller, E., Hergueta, T., Baker, R., & Dunbar, G. (1998). The Mini International Neuropsychiatric Interview (M.I.N.I.): The Development and Validation of a Structured Diagnostic Psychiatric Interview. *Journal of Clinical Psychiatry*, **59**(20), 22-33.

124. 角野善司. (1994). 人生に対する満足度尺度（the Satisfaction With Life Scale [SWLS]）日本語版作成の試み. 日本教育心理学会総会発表論文集, **36**, 192.

125. Tabuse, H., Kalali, A., Azuma, H., Ozaki, N., Iwata, N., Naitoh, H., Higuchi, T., Kanba, S., Shioe, K., Akechi, T., & Furukawa, T. A. (2007). The new GRID Hamilton Rating Scale for depression demonstrates excellent inter-rater reliability for inexperienced and experienced raters before and after training. *Psychiatry Research*, **153**(1), 61–67.

126. Taylor, C. T., Lyubomirsky, S., and Stein, M. B. (2017). Upregulating the positive affect system in anxiety and depression: Outcomes of a positive activity intervention. *Depression and Anxiety*, **34**, 267–280.

127. Treadway, M. T., Buckholtz, J. W., Schwartzman, A. N., Lambert, W. E., & Zald, D. H. (2009). Worth the 'EEfRT'? The effort expenditure for rewards task as an objective measure of motivation and anhedonia. *PLoS ONE*, **4**(8), e6598.

128. Williams, J. B., Kobak, K. A., Bech, P.,. Evans, K., Lipsitz, J., Olin, J., Pearson, J., & Kalali, A. (2008). The GRID- HAMD: standardization of the Hamilton Depression Rating Scale. *International Clinical Psychopharmacology*, **23**(3), 120–129.

129. Vos, T., Barber, R. M., Bell, B., Bertozzi-Villa, A., Biryukov, S., Bolliger, I., et al. (2015). Global, regional, and national incidence, prevalence, and years lived with disability for 301 acute and chronic diseases and injuries in 188 countries, 1990–2013, a systematic analysis for the Global Burden of Disease Study 2013. *Lancet*, **386**(9995), 743–800.

Statistical analysis plan for the augmentation of positive valence system-focused cognitive behavior therapy by inaudible high-frequency sound: A placebo-controlled randomized trial

Masaya Ito, Ph.D., and Kazushi Maruo, Ph.D.

Draft version: 9th of August 2018
Complete version: 1st of November 2018
First revised version: 23th of May 2019

This SAP follows the “Guidelines for content of statistical analysis plans in clinical trials (Gamble et al., 2017, JAMA, 318(23):2337-2343. doi:10.1001/jama.2017.18556)”

Section 1: Administrative Information

1.Title and trial registration

1a. Title

Statistical analysis plan for the augmentation of positive valence system-focused cognitive behavior therapy by inaudible high-frequency sound: A placebo-controlled randomized trial.

1b. Trial registration

UMIN000031948

2. SAP version

First draft, 9th of August 2018

First complete version, 1st of November 2018

Second modified version: 23th of May 2019

3. Protocol version

3rd version, 9th of August 2018

4. SAP revisions

4a. SAP revision history

Draft version developed on the 9th of August 2018

Complete version developed on the 1st of November 2018

First revised version developed on the 23rd of May, 2019.

4b. Justification for each SAP revision

The modified parts in first revision are 27 and 32c. In 27, we added strategy to handling the situation of no convergence using unstructured structure as the covariance structure for the mixed model for repeated measures. Other parts were edited using a professional English editing service.

4c. Timing of SAP revisions in relation to interim analysis, etc.

We have no plans to conduct an interim analysis.

5. Roles and responsibilities

Masaya Ito, Ph.D., National Center of Neurology and Psychiatry, conceived and drafted the SAP.

Kazushi Maruo, Ph.D., University of Tsukuba, modified the drafted version of the SAP.

6. Signatures of:

6a. Person writing the SAP	Masaya Ito	____________________
6b. Senior statistician responsible	Kazushi Maruo	____________________
6c. Chief investigator/clinical lead	Masaya Ito	____________________

Section 2: Introduction

7. Background and rationale

The background and rationale for this study is written on pages 5–8 of the 3rd version of the IRB approved study protocol.

8. Objectives

We aimed to test the efficacy of combining a positive valence system-focused CBT with an inaudible high-frequency sound to improve anhedonia. The objective of this clinical trial was to test the efficacy of a positive valence system-focused CBT with an inaudible high-frequency sound compared to a positive valence system-focused CBT with a placebo sound on the symptoms of anhedonia (Snaith-Hamilton Pleasure Scale) in 44 patients with anhedonia.

Section 3: Study Methods

9. Trial design

Individual-level, allocated with a ratio of 1:1, treatment intervention, exploratory, randomized, therapist-, patient-, and evaluator-masked, parallel group, placebo-controlled, single-site, phase II trial.

10. Randomization

The procedure of randomization is written on page. 30 in the 3rd version of the IRB approved study protocol.

11. Sample size

The details of the sample size calculation is written on page. 10 of the 3rd version of the IRB approved study protocol.

12. Framework

The purpose of this trial is to test the superiority of a positive valence system focused-cognitive behavioral therapy with an inaudible high-frequency sound compared to a positive valence system focused-cognitive behavioral therapy with a placebo sound.

13. Statistical interim analyses and stopping guidance

13a. Information on the interim analysis

We have no plan to conduct an interim analysis.

13b. Any planned adjustment of the significance level due to the interim analysis

N/A

13c. Details of guidance for stopping the trial early

We have no plan to stop the trial due to the results of the interim analysis because we have no plans to conduct an interim analysis. However, we will discontinue the trial based on the following criteria (page. 41 of the 3rd version of IRB approved study protocol).

10） If the IRB advises or orders the discontinuation of the trial, this trial will be stopped. The committee consisting of the primary investigator and members of the DSMB will examine the appropriateness of continuing the trial when the following occurs.

11） Acknowledgement of important information regarding quality, safety, and efficacy of the intervention.

12） When the IRB orders modification of the trial procedures and the primary investigator judges such modifications to be difficult. If the primary investigator decides to stop or discontinue the trial, the primary investigator will report the reason immediately to the IRB in a written form. After deciding to discontinue the trial, the primary investigator will immediately notify the related doctors, psychologists, and other medical staff, and deal with the consequences of the discontinuation.

14. Timing of the final analysis

All outcomes will be analyzed collectively after fixing the data obtained by the final assessment (follow-up) of the final participant (44th participant).

15. Timing of outcome assessments

Assessments related to the primary analysis will be conducted at visits 2, 4, 5, 6, 7, 8, 9, 10, 11, and 12. The overall description of the outcome assessment points is shown in Fig.2 in the 3rd version of the study protocol (also displayed below).

					ENROLMENT			INTERVENTION								POST	FU
TIME POINT (Week)					-2	-1	0	1–11								12	21
Visit					V1	V2	V3	V4	V5	V6	V7	V8	V9	V10	V11	V12	V13
				(Burden for patient (minutes))													
ENROLLMENT :																	
	Informed Concent			(50)	X												
	Randomization			(5)			X										
	Intake			(60)			X										
ASSESSMENTS :																	
Diagnosis		MINI	IE	(25)		X											
Primary Outcome	Anhedonia	SHAPS	Pt	(4)		X		X	X	X	X	X	X	X	X	X	X
Secondary Outcome	Anhedonia	SHAPS-C	IE	(10)		X										X	X
Other Outcome	Depression/Anhedonia	GRID-HAMD	IE	(30)		X										X	X
		BDI-II	Pt	(5)			X					X				X	X
	Emotion	PANAS	Pt	(3)			X	X	X	X	X	X	X	X	X	X	X
	Life Satisfaction	SWLS	Pt	(2)			X					X				X	X
	Well-being	PWB	Pt	(6)			X					X				X	X
Treatment Mechanism	Anhedonia	EROS	Pt	(3)			X					X				X	X
		EEfRT	Pt	(20)		X										X	X
Safety	Adverse Event	AE	Th	-				X	X	X	X	X	X	X	X	X	X
Adherence	Homework Compliance	HC	Th	-					X	X	X	X	X	X	X		
Blinding		IEKNO	Pt/Th/IE	(2)				X								X	X
Burden for Participants(minutes)					(50)	(89)	(84)	(9)	(7)	(7)	(7)	(23)	(7)	(7)	(7)	(83)	(83)

Fig 2. Overall description of the measures and its timepoints.
IE: Interview with Independent Evaluator, Pt: Participant self-report, Th: Therapist self-report

Section 4: Statistical Principles

16. Level of statistical significance

The *p*-value will be expressed to three decimal places. If the *p*-value is under .001, it will be described as $p < .001$. When the appropriate statistical tests are applied, we will set the statistical significance at 5% using a two-tailed test.

17. Description and rationale for any adjustment for multiplicity

As this study is designed to test the primary outcome, analyses for secondary and other outcomes, as well as for other purposes (sub-group analysis, adjusted analysis, etc.) will be exploratory. We have no plan to adjust for multiplicity with regards to the analyses of the secondary and other outcomes.

18. Confidence intervals to be reported

We will report confidence intervals with a confidence level of 95%

19. Adherence and protocol deviations

19a. Definition of adherence to the intervention and how this is assessed including the extent of the exposure

PoCot: The definition of adherence to the PoCot is dependent on the extent to which the therapist will follow the predetermined intervention procedure.

Audio exposure: Adherence to audio exposure is defined as an exposure to a predetermined sound duration with a predetermined volume at a predetermined distance.

19b. Description of how adherence to the intervention will be presented

PoCot: Adherence to the PoCot will be evaluated by the other staff therapists using the adherence evaluation scale. We will randomly sample 35 sessions (10 percent of all planned 8 sessions for 44 participants).

Audio sound exposure: When the therapist operates the sound panel by switching on the audio track, the therapist will also record the operation and confirm the track number and the volume. In addition, the presentation of the inaudible high-frequency (or placebo) sound by a predetermined track will be verified once a month by the sound management staff (YY, SK).

19c. Description of any protocol deviations will be summarized

The content of any protocol deviations will be recorded for each participant. After the completion of the trial, we will classify and summarize the deviations in a table.

20. Analysis populations

Analyses of the primary and secondary outcomes will be conducted by following the Intent-To-Treat principle. All the registered participants will be subject to analysis. As the secondary analysis set, we will conduct the outcome analysis by using the all registered participant who will not meet the discontinuing criteria as Per Protocol Set.

Section 5: Trial Population

21. Screening data

All participants will be referred from a psychiatrist at the National Center Hospital of Neurology and Psychiatry. Then, the participants will be required to sign an informed consent to participate in the study. Hence, we will not screen the participants.

22. Eligibility

The inclusion and exclusion criteria for this study are as follows (also written on pages. 33–35 in the 3rd version of the IRB approved study protocol).

1). Inclusion criteria

(1) Anhedonia symptoms (Snaith-Hamilton Pleasure Scale score >= 20)

(2) Depressive symptoms ranging from mild to severe (GRID Hamilton depression rating scale >= 8)

(3) Aged 18 years or older

2). Exclusion criteria

19） No current psychotic disorders at baseline as assessed by the Mini-International Neuropsychiatric Interview (MINI)

20） No current manic episodes at baseline assessed by MINI

21） No severe substance use disorders at baseline assessed by MINI

22） No serious suicidal ideation at baseline assessed by MINI

22. Recruitment

The information to be included in the CONSORT flow diagram include:

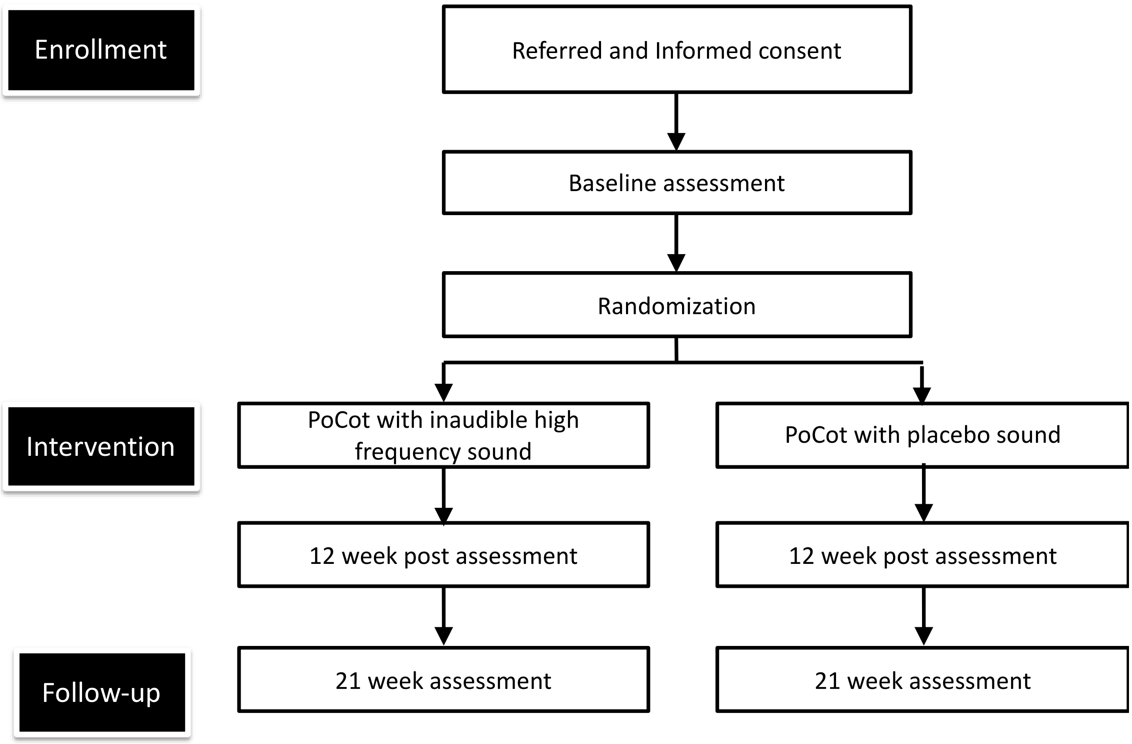

24. Withdrawal/follow-up

24a. Level of withdrawal

The participants will be able to present their willingness to withdraw anytime during the trial. We defined level 1 withdrawal as withdrawal during the period from registration to the 12-week assessment, and level 2 withdrawal (lost to follow-up) as withdrawal during the period from the 12-week assessment to the 21-week assessment.

24b. Timing of withdrawal/lost to follow-up data

Participants will be able to withdraw anytime during the trial.

24c. Reasons and details of how the withdrawal/lost to follow-up data will be presented.

We will describe the reasons for any withdrawals in accordance with the discontinuation criteria.

25. Baseline patient characteristics

25a. List of baseline characteristics to be summarized

The baseline characteristics were: Age, gender, marital status, current occupation (education and/or employment), numbers of previously utilized medical institutions, years with mental illness,

previous psychiatric hospitalization, principal diagnosis, comorbid diagnoses, current psychotropic medication, psychiatric illness, habitual use of tobacco and alcohol, and past education.

25b. Details of how the baseline characteristics will be descriptively summarized

Baseline characteristics will be presented using the following table:

Table 1. Baseline characteristics to be presented in the primary outcome paper

	Total (N =)	PoCot with inaudible high-frequency sound (N =)	PoCot with placebo sound (N =)
Age, mean (SD), y			
Gender			
Marital status			
Single			
Married			
Divorced			
Widowed			
Other			
Current occupation			
Employment			
Education			
Medical leave from job			
Medical leave from education			
Home maker			
Part time job			
Retired			
Principal diagnosis			
Depressive disorder			
Anxiety disorder			
Other			
Comorbid diagnoses			
Depressive disorder			
Anxiety disorder			
Other			
Current psychotoropic medication			
SSRI			
SNRI			
Benzodiazepine			
Tricyclic antidepressant			
Years with mental problem			
Number of previously utilized medical institutions			
Previous psychiatric hospitalizations			
Psychiatric illness			
Habitual use of alcohol			
Habitual use of tobacco			
Past Education			
Elementary school			
Junior high school			
High school			
Professional school			
Two-year college			
University/college			
Graduate school			

A category with less than 4 will be integrated with the "other" category

Section 6: Analysis

26. Outcome definitions

26a. Specification of outcomes and timings

The primary outcome is the score of the Japanese version of the Snaith-Hamilton Pleasure Scale (SHAPS) assessed nine times from week 1 to week 12.

The secondary outcomes are the score of the SHAPS-C at week 12 and the BDI-II, PANAS, SWLS, PWB, EROS, and EEfRT at weeks 5 and 12.

26b. Specific measurement and units

The total score of the Japanese version of the SHAPS will be used. The score ranges from 14 to 56.

26c. Any calculation or transformation used to derive the outcome

We will use the total score of the Japanese version of the SHAPS. The total score will be based on the responses to the 14 items.

27. Analysis methods

27a. Which analysis methods will be used and how the treatment effects will be presented

[Analysis of the primary outcome]

A mixed model for repeated measures (MMRM) will be conducted to analyze the primary outcome. The dependent variable is the participants' SHAPS scores assessed at eight visit points from weeks 1 to 11 (intervention period) and week 12 (post-assessment). Fixed-effects are allocation, visit, and allocation-by-visit interaction, and SHAPS at pre-intervention (week -1). The covariance structure is specified as the unstructured structure. Then, the treatment effects will be presented as the difference in the adjusted means between the allocations at week 12 (visit 12: primary visit). Following this, the estimation of the confidence intervals and the t-test for the treatment effect will be conducted. The t-test will be the primary statistical test. If the model estimation process will fail to converge, the covariance structures will be specified as 1st order autoregression structure and the robust inference proposed by Mancl and DeRouen (*Biometrics* 2001; 57: 126–134.) will be conducted. The inference on the treatment effect for the other weeks and the standardized treatment effect will also be conducted.

The sample SAS code for the primary analysis is as follows:

```
proc mixed data=[Data];
  class [Subject] [Week] [Allocation];
  model [Outcome] = [Allocation] [Week] [Allocation] * [Week] [Baseline] / s cl ddfm = kr;
  repeated [Week] / subject = [Subject] type = UN;
```

```
  lsmeans [Allocation] * [Week] / diff cl;
run;
```

For robust inference:

```
proc glmmix data=[Data] empirical = firores;
  class [Subject] [Week] [Allocation];
  model [Outcome] = [Allocation] [Week] [Allocation] * [Week] [Baseline] / s cl;
  random [Week] / subject = [Subject] residual type = AR(1);
  lsmeans [Allocation] * [Week] / diff cl;
run;
```

[Analysis of the secondary outcomes]

Analysis of covariance including baseline will be conducted to test the intervention effect on the SHAPS-C scores at week 12. In addition, an MMRM analysis will be conducted for the other outcomes (BDI-II, PANAS, SWLS, PWB, EROS, and EEfRT at weeks 5 and 12) similar to the primary analysis. Furthermore, inference on the differences in the adjusted means between allocations at each visit will be conducted.

27b. Any adjustment for covariates

We will set SHAPS at pre-intervention as the covariate for the primary analysis.

27c. Methods used for verification of the assumptions for the statistical methods

If the sample size ratio between allocations at week 12 is larger than 1.5 or smaller than 0.67 due to missingness, a likelihood ratio test for heteroscedasticity between allocations will be conducted. The normality of the error distribution for the statistical model will not be evaluated because extreme values and/or heavily skewed distributions will not be observed for the primary outcome and the effect of non-normality on the treatment effect would be negligible.

27d. Details of alternative methods to be used if the distributional assumptions do not hold

We will conduct an MMRM analysis with the assumption of heteroscedasticity between the allocations if heteroscedasticity is detected.

27e. Any planned sensitivity analyses for each outcome where applicable

We will conduct sensitivity analyses by applying the analysis of covariance at week 12 assuming both “best” and “worst” case scenarios as imputations of missing data.

27f. Include any planned subgroup analysis for each outcome including how the subgroups are defined

We will conduct subgroup analyses by dividing the participants into those who have a principal diagnosis of depressive disorder and those who have a principal diagnosis of anxiety disorder as assessed by MINI.

28. Missing data

We will use the MMRM analysis to treat any missing data. Furthermore, we will conduct sensitivity analyses by assuming both “best” and “worst” case scenarios.

29. Additional analyses

Additional analyses will be conducted if necessary.

30. Harmful effects

The presence or absence of adverse events will be assessed using the following items: dry mouth, astriction, dysuria, vision dysregulation (accommodation disturbance), orthostatic hypotension, sleepiness, fatigue, sleeplessness, anxiety/agitation, depression/anhedonia, lack of appetite, gain or loss of body weight, loss of sexual desire, palpitations, thrill, diaphoresis, headache, dizziness, other. Using forms, research staff (therapists or evaluators) will ask about any adverse event as follows, “Have you been experiencing any worsening or occurrence of physical or mental symptoms?”

The research staff will record the content of the adverse event, occurrence date, date of improvement, severity, conducted response, course, and relevance to the intervention on the CRF. These records will be summarized in Table 2. The occurrence of adverse events will be compared between the intervention and comparison group by using chi-square tests.

Table 2. Summary of adverse events

	During treatment		During follow-up	
	PoCot with inaudible high-frequency sound (N =)	PoCot with placebo sound (N =)	PoCot with inaudible high-frequency sound (N =)	PoCot with placebo sound (N =)
# of patients reporting AEs				
# of AEs reported				
# of SAEs reported				
# Related to study				
Increased Anhedonia				
Increased Depression				
Suicidal ideation				
etc				
# Related to study				
Increased Anhedonia				
Increased Depression				
Suicidal ideation				
etc.				
AEs by type				
Psychological				
Medical				

Note. The subcategory of the AEs will be presented in accordance with its occurrence.

31. Statistical software

We will use the statistical software SPSS Ver 19 or later, SAS Ver. 9,4 or later, and/or R Ver. 3.5.0 or later

32. References

32a. References to be provided for nonstandard statistical methods.

We will not use nonstandard statistical methods.

32b. Reference to Data Management Plan

Data management plan for this trial is available only in Japanese.

32c. Reference to the Trial Master File and Statistical Master File

Even though we will not develop a detailed trial master file for this trial, every document will be stored in a locked cabinet in a locked room at the National Center of Neurology and Psychiatry, Japan.

32d. Reference to other standard operating procedures or documents to be adhered to

The standard operating procedures for this trial include the following. All of these documents were written in Japanese;

Monitoring plan

SOP for data safety management board

SOP for clinical research coordinator

SOP for allocation

SOP for management of personal information

SOP for trial therapist

SOP for reporting adverse event

SOP for management of primary data source

Reference

Mancl, L. A. and T. A. DeRouen (2001). "A covariance estimator for GEE with improved small-sample properties." Biometrics **57**(1): 126-134.
