# Supplementary material for: Augmentation of Positive Valence System–Focused Cognitive Behavioral Therapy by Inaudible High-Frequency Sounds for Anhedonia: A Trial Protocol for a Pilot Study
Source: JAMA Netw Open. 2019 Nov 20;2(11):e1915819. doi: 10.1001/jamanetworkopen.2019.15819 (PMC6902816; doi:10.1001/jamanetworkopen.2019.15819)
Supplement: Supplement 2. — eAppendix. Biological and Theoretical Rationale and Treatment Descriptions of Positive Valence System–Focused Cognitive Behavioral Therapy eFigure 1. Speaker and Super-Tweeter eFigure 2. Layout of the Therapy Room eTable. Description and Findings Related to Positive Valence System–Focused Cognitive Behavioral Therapy Treatment Modules for Anhedonia eReferences. [file jamanetwopen-2-e1915819-s002.pdf]

Supplementary Online Content

Ito M, Miyamae M, Yokoyama C, et al. Augmentation of positive valence system–focused cognitive behavioral therapy by inaudible high-frequency sounds for anhedonia: a trial protocol for a pilot study. *JAMA Netw Open*. 2019:2(11):e1915819. doi:10.1001/jamanetworkopen.2019.15819

eAppendix. Biological and Theoretical Rationale and Treatment Descriptions of Positive Valence System–Focused Cognitive Behavioral Therapy
eFigure 1. Speaker and Super-Tweeter
eFigure 2. Layout of the Therapy Room
eTable. Description and Findings Related to Positive Valence System–Focused Cognitive Behavioral Therapy Treatment Modules for Anhedonia
eReferences

This supplementary material has been provided by the authors to give readers additional information about their work.

© 2019 Ito M et al. *JAMA Network Open.*

eAppendix. Biological and Theoretical Rationale and Treatment Descriptions of Positive Valence System–Focused Cognitive Behavioral Therapy

As discussed in previous literature regarding the psychological treatments developed to specifically target anhedonia or achieve a positive affect [1-3], neuroscience research has been demonstrated that anhedonia is associated with various neuronal circuity or neurotransmitters related to reward processing. In the same manner as previous novel positive affect interventions, we referred to the accumulating findings of biological, neuroscience and emotion research in our rationale for the selected treatment targets in the positive valence system-focused cognitive behavior therapy (PoCot). Since there is little evidence of specific intervention to the subprocesses or the entire process of reward processing in individuals with anhedonia, we referred to the intervention techniques that have been shown to be effective in enhancing one's positive affect or related constructs (e.g., well-being and satisfaction with life) among people in clinical and non-clinical populations. The goal, description, targeted reward processes, biological/theoretical rationale, and findings that relate to utilized intervention technique are described in eTable 1. Cited references in eTable 1 are presented in the references section of this supplemental text. In this supplemental text, we focus on the biological/neurological correlates of the positive-valence system to clarify our treatment targets in PoCot and describe the intervention technique to improve the targeted processes. Though we could not acquire the treatment protocol from the author describing positive affect training at the time we conducted this pilot clinical trial (June, 2017), our basic strategy in developing the PoCot was largely influenced by the positive affect training developed by Dr. Craske and her research team [2].

As with positive affect training [2], we targeted the 3 major components of reward processing: anticipation, consumption, and learning [4] [5]. According to Craske et al.(2016), these components are described as follows "the anticipatory component refers to motivation for rewarding stimuli, such as planning and looking forward to a vacation, and is related to the effort expended to receive reward. It is dominated by 'wanting.' The consummatory component refers to the pleasure or hedonic impact of rewarding stimuli, such as the pleasure while on vacation, and is dominated by 'liking.' The learning component typically involves Pavlovian or instrumental associations and predictions about future rewards based on past experiences, such as the decision to take another vacation given the rewards of the last vacation (p.930)". We targeted these 3 components because biological or neuronal studies have shown that anhedonia is associated with the deficits in these subsystems of reward processing. Moreover, clear treatment technique descriptions corresponding to these components were provided in literature [2]. In the psychoeducation conducted in the first PoCot session, we presented this framework as the treatment rationale for targeting these subsystems of reward processing (i.e., wanting, liking, learning).

In terms of biological correlates of reward anticipation, it is suggested that dopaminergic signaling and specific neuronal regions such as the ventral tegmental area, amygdala, and ventral striatum

© 2019 Ito M et al. *JAMA Network Open.*

are involved [6] [4]. Furthermore, individuals with depression showed reduced activation in reward circuitries during reward anticipation [7] [8] [9]. Also, people with dysphoria may exhibit diminished positive emotions in future positive events [10]. To improve these deficits, PoCot participants were asked to schedule and conduct the activities that inspire positive emotions for them. A similar technique is used in positive affect intervention [11], positive affect training [2], and Engage therapy [1].

Reward consumption is suggested to related to opioid and endocannabinoid pathways [12]. In terms of neuronal regions, reward consumption is associated with the activation in the ventral striatum and orbitofrontal cortex [4]. Moreover, a lower score on a self-report measure concerning positive emotions to positive stimuli is related to anhedonia [13]. Though the evidence is mixed, some studies suggest the relationship involves a reduction in attention to positive stimuli in individuals with depression compared to controls [14]. To enhance attention to positive stimuli and fully experience the positive sensations and emotions, the participants were asked to practice savoring positive sensations and emotions, and to attentively monitor the positive events in daily life. This type of savoring excise is reported to successfully enhance positive emotions in non-clinical populations [15].

Reward learning is thought to coincide with the reward anticipation and consumption. Notably, reward learning is associated with dopaminergic signaling [16]. In terms of neuronal regions, reward learning and decision making may be associated with the anterior cingulate, orbitofrontal, ventral medial prefrontal, and dorsal lateral prefrontal cortices [4]. Patients with depression showed significantly reduced reward learning signals in the ventral striatum, rostal and dorsal anterior cingulate, retrosplenial cortex, midbrain, and hippocampus [17]. Also, reduced reward learning signals correlate with depression severity [17]. Moreover, it is suggested that lower reward learning predicts higher anhedonia symptoms for the next month [18]. To improve the reward learning process, PoCot participants were asked to monitor and record their actions during the positive events. Instead of focusing on external or situational factors, they were asked to focus what they actually did to making such positive events happen and what they did when the positive experience continued. This training is expected to enhance learning and instrumental conditioning between their behavior and positive outcomes (i.e., reward learning). This technique is similar to scheduling pleasant events in positive affect training [2]. Also similar to positive affect training, we asked participants to vividly recall and savor the positive experiences during the subsequent sessions. This technique is also expected to enhance and consolidate reward learning [19].

In addition to the above findings, we have learned from “Engage” therapy, an approach theoretically based on the framework of the positive-valence system in research domain criteria [1]. In “Engage” therapy, the subprocesses of the positive valence system such as reward valuation, effort valuation, action selection, preference-based decision making, and reward learning are targeted in the intervention. In developing PoCot, we included the treatment techniques in “Engage” therapy (e.g., reward exposure.)

© 2019 Ito M et al. *JAMA Network Open.*

eFigure 1. Speaker and Super-Tweeter

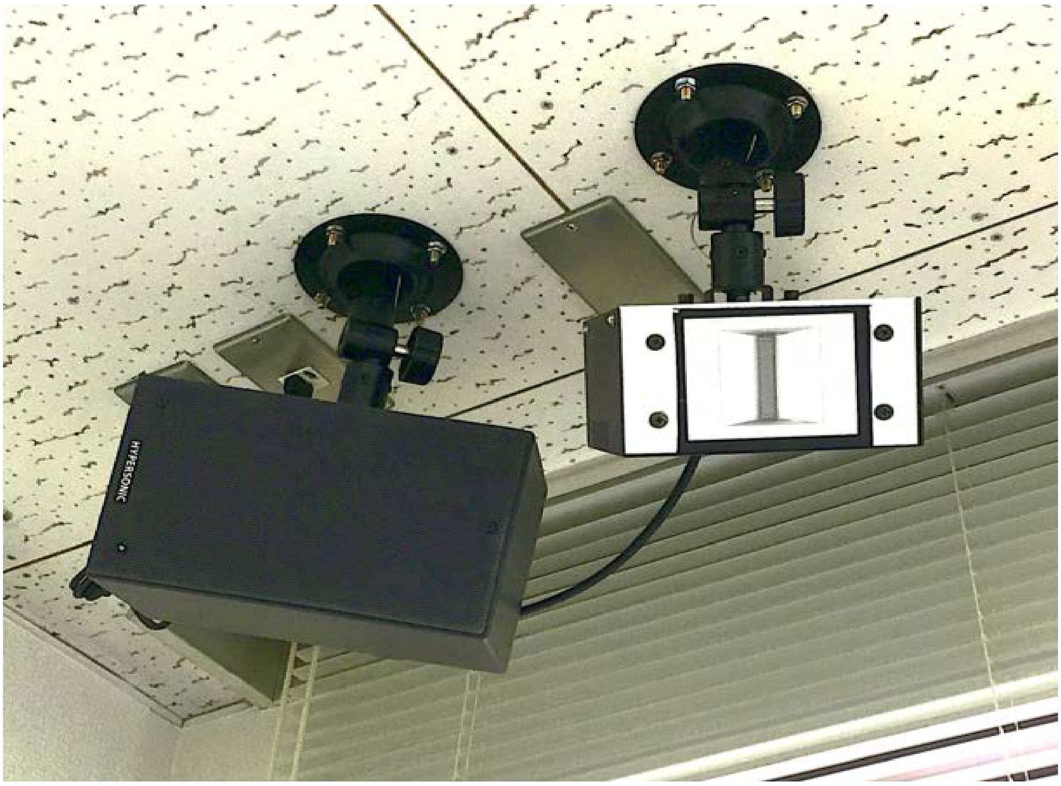

Speaker (left side) and super tweeter (right side).

© 2019 Ito M et al. *JAMA Network Open.*

eFigure 2. Layout of the Therapy Room

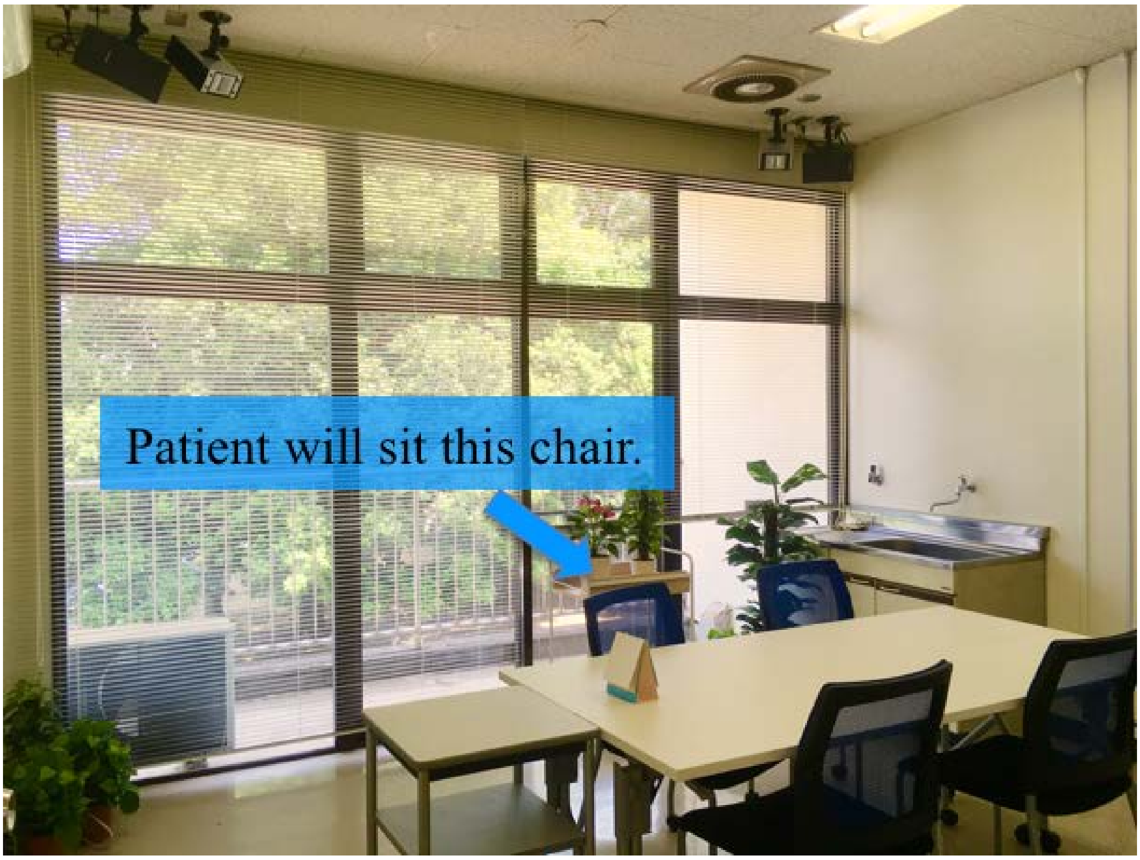

© 2019 Ito M et al. *JAMA Network Open.*

eTable. Description and Findings Related to Positive Valence System–Focused Cognitive Behavioral Therapy Treatment Modules for Anhedonia

Session	Module	Goal	Description	Targeted reward processing	Biological/theoretical rationale	Findings related to intervention technique
#1	Psychoeducation	Understand the treatment rationale that focuses on the positive-valence system; self-monitoring of positive events; savoring exercise	Present the neuroscience model of the positive valence system (wanting, liking, learning). Begin scheduling pleasant events, record positive events and associated behaviors and emotions in a diary, practice savoring exercise	Anticipation, consumption, learning	Neuroscience model of reward processing [2] [20] [5]<br>Deficits in reward anticipation, consumption, and learning [21] [22] [23] [24] [25]<br>Deficits in positive emotion regulation [26]	Record positive events [27]<br>Pleasant event scheduling [2] [28]<br>Savoring exercise [15]<br>Savoring the moment [29]

© 2019 Ito M et al. *JAMA Network Open.*

#2	Heal yourself	Further understand the rationale (e.g., the function of positive emotions); direct attention to positive events (especially those that are healing and/or comforting), sensations, and emotions.	Participants attentively monitor positive events during the week and the savoring exercise via a diary; present the nature and function of various positive emotions; continue positive diary and savoring exercises	Consumption, learning	Discrete model of positive emotions and neurological correlates [30]<br>Function of positive emotions [31]<br>Relationship between chronic stress and anhedonia [32]<br>Possibility of vagal afference as a treatment of depression [33]<br>Positive emotion regulation [34]	Training attention to positive stimuli [35]<br>Modification of the attribution process [36]
#3	Your favorite things	Awareness and understanding of one's own preferences (e.g., favorite sensations, activities, etc.)	Participants are asked to identify their own preferences in various aspects (e.g., 5 sensations, people, places, activities, music, words, things they never engaged, etc.); schedule and conditioning for the	Consumption, learning	Model of orbitofrontal cortex functions (Reward value representations) [37]<br>Relationships between ventral striatum hypoactivity to positive stimuli and anhedonia [9] [38] [39] [40]<br>Reduced reward experience in individual with depression [41]	Reward exposure [1]<br>Positive activities [42]

© 2019 Ito M et al. *JAMA Network Open.*

			participant's favorite activities; identify dampening thoughts			
#4	Positive memories and strength	Identify past positive events; identity strengths and weaknesses in contexts	Participants are asked to recall positive memories (e.g., achievements, support, enjoyment, appreciation); bring photos related to positive events; Identify the strengths and weaknesses in a related context; schedule an activity that will utilize a strength	Consumption, learning	Human character strengths and virtues [43]<br>Strength and vulnerability integration [44]<br>Vulnerability as a strength [45]<br>Relations of positive autobiographical memory retrieval, reduced temporal discounting, and striatum activation [46]<br>Positive functions of nostalgia [47]	Identify and utilize one's own strength [48]<br>Strength based positive psychology intervention [49]<br>Positive photo appreciation [50]<br>Positive affect stimulation and sustainment [27]<br>Recall happiest experiences [51]

© 2019 Ito M et al. *JAMA Network Open.*

#5	Positivity in an effortful task	Understand the importance of effortful decision making and a process-oriented view	Present the model of effortful based decision making; train attention to the micro-positive process in conducting effortful activities; schedule engagement in effortful activities and focusing on the positive aspects during it	Anticipation, consumption, learning	Temporal discounting in individuals with depression [52 53]<br>Choice of everyday activities [54]<br>Effort-based decision making [55]	Breaking down an achievable task [2]<br>Modification of the attribution process [36]
#6	Long-term goals and values	Identify the personal values and long-term goals; engage in the related activities	Participants identify their personal values and long-term goals in various domains (e.g., school/career, social relationships etc.); schedule value or goal related activities that feel meaningful to them	Anticipation, consumption, learning	Value clarification in behavioral activation [56]<br>Individual with mild depression less likely to engage in behaviors perceived as rewarding in terms of intermediate and more distal [57]	Personal value [58]<br>Meaningful activities [59 60]<br>Goal-training program [61]

© 2019 Ito M et al. *JAMA Network Open.*

#7	Gratitude and kindness	Maintain positive interpersonal relationships via gratitude and acts of kindness	Participants identify their comfortable interpersonal relationships; schedule daily gratitude acknowledgement and acts of kindness	Consumption, learning	Theoretical review of gratitude [62]<br>Acts of kindness [63]	Gratitude [32,46,47]<br>Acts of kindness [48,49,50]
#8	Preventing relapse	Acknowledge the treatment progress; plan for continuing practice	Review the progress by comparing various reward processing aspects with the baseline; develop a plan to maintain benefits.	Learning	Relapse prevention [64]	Modification of the attribution process [36]<br>Relapse prevention in positive affect training [2] and positive affect intervention [11]
Note. Full references are included in the Supplemental text						

© 2019 Ito M et al. *JAMA Network Open.*

eReferences

1. Alexopoulos GS, Arean P. A model for streamlining psychotherapy in the RDoC era: the example of 'Engage'. *Molecular psychiatry.* 2014;19(1):14-19.
2. Craske MG, Meuret AE, Ritz T, Treanor M, Dour HJ. Treatment for Anhedonia: A Neuroscience Driven Approach. *Depression and anxiety.* 2016;33(10):927-938.
3. Henje Blom E, Tymofiyeva O, Chesney MA, et al. Feasibility and Preliminary Efficacy of a Novel RDoC-Based Treatment Program for Adolescent Depression: "Training for Awareness Resilience and Action" (TARA)-A Pilot Study. *Frontiers in psychiatry.* 2016;7:208.
4. Der-Avakian A, Markou A. The neurobiology of anhedonia and other reward-related deficits. *Trends in neurosciences.* 2012;35(1):68-77.
5. Romer Thomsen K, Whybrow PC, Kringelbach ML. Reconceptualizing anhedonia: novel perspectives on balancing the pleasure networks in the human brain. *Front Behav Neurosci.* 2015;9:49.
6. Castro DC, Berridge KC. Advances in the neurobiological bases for food 'liking' versus 'wanting'. *Physiol Behav.* 2014;136:22-30.
7. Forbes EE, Hariri AR, Martin SL, et al. Altered striatal activation predicting real-world positive affect in adolescent major depressive disorder. *The American journal of psychiatry.* 2009;166(1):64-73.
8. McCabe C, Cowen PJ, Harmer CJ. Neural representation of reward in recovered depressed patients. *Psychopharmacology (Berl).* 2009;205(4):667-677.
9. Pizzagalli DA, Holmes AJ, Dillon DG, et al. Reduced caudate and nucleus accumbens response to rewards in unmedicated individuals with major depressive disorder. *Am J Psychiatry.* 2009;166(6):702-710.
10. Hoerger M, Quirk SW, Chapman BP, Duberstein PR. Affective forecasting and self-rated symptoms of depression, anxiety, and hypomania: evidence for a dysphoric forecasting bias. *Cogn Emot.* 2012;26(6):1098-1106.
11. Taylor CT, Lyubomirsky S, Stein MB. Upregulating the positive affect system in anxiety and depression: outcomes of a positive activity intervention. *Depress Anxiety.* 2017;34(3):267-280.
12. Mahler SV, Smith KS, Berridge KC. Endocannabinoid hedonic hotspot for sensory pleasure: anandamide in nucleus accumbens shell enhances 'liking' of a sweet reward. *Neuropsychopharmacology : official publication of the American College of Neuropsychopharmacology.* 2007;32(11):2267-2278.
13. Berlin I, Givry-Steiner L, Lecrubier Y, Puech AJ. Measures of anhedonia and hedonic responses to sucrose in depressive and schizophrenic patients in comparison with healthy subjects. *Eur Psychiatry.* 1998;13(6):303-309.
14. Peckham AD, McHugh RK, Otto MW. A meta-analysis of the magnitude of biased attention in depression. *Depression and anxiety.* 2010;27(12):1135-1142.
15. Jose PE, Lim BT, Bryant FB. Does savoring increase happiness? A daily diary study. *J Posit Psychol.* 2012;7(3):176-187.
16. Flagel SB, Clark JJ, Robinson TE, et al. A selective role for dopamine in stimulus-reward learning. *Nature.* 2011;469(7328):53-57.

© 2019 Ito M et al. *JAMA Network Open.*

17. Kumar P, Waiter G, Ahearn T, Milders M, Reid I, Steele JD. Abnormal temporal difference reward-learning signals in major depression. *Brain.* 2008;131(Pt 8):2084-2093.
18. Pizzagalli DA, Jahn AL, O'Shea JP. Toward an objective characterization of an anhedonic phenotype: a signal-detection approach. *Biological psychiatry.* 2005;57(4):319-327.
19. Pictet A, Coughtrey AE, Mathews A, Holmes EA. Fishing for happiness: the effects of generating positive imagery on mood and behaviour. *Behav Res Ther.* 2011;49(12):885-891.
20. Berridge KC, Kringelbach ML. Pleasure systems in the brain. *Neuron.* 2015;86(3):646-664.
21. Treadway MT, Zald DH. Reconsidering anhedonia in depression: lessons from translational neuroscience. *Neuroscience and biobehavioral reviews.* 2011;35(3):537-555.
22. Pizzagalli DA. Depression, stress, and anhedonia: toward a synthesis and integrated model. *Annu Rev Clin Psychol.* 2014;10:393-423.
23. Pizzagalli DA, Iosifescu D, Hallett LA, Ratner KG, Fava M. Reduced hedonic capacity in major depressive disorder: evidence from a probabilistic reward task. *J Psychiatr Res.* 2008;43(1):76-87.
24. McFarland BR, Klein DN. Emotional reactivity in depression: diminished responsiveness to anticipated reward but not to anticipated punishment or to nonreward or avoidance. *Depression and anxiety.* 2009;26(2):117-122.
25. Russo SJ, Nestler EJ. The brain reward circuitry in mood disorders. *Nat Rev Neurosci.* 2013;14(9):609-625.
26. Carl JR, Soskin DP, Kerns C, Barlow DH. Positive emotion regulation in emotional disorders: a theoretical review. *Clin Psychol Rev.* 2013;33(3):343-360.
27. McMakin DL, Siegle GJ, Shirk SR. Positive affect stimulation and sustainment (PASS) module for depressed mood: A preliminary investigation of treatment-related effects. *Cognit Ther Res.* 2011;35(3):217-226.
28. Lewinson PM. A behavioral approach in depression. In: Friedman RJ, Katz MM, eds. *The Psychology of Depression: Contemporary Theory and Research.* New York, NY: Wiley; 1974:145–185.
29. Hurley DB, Kwon P. Results of a study to increase savoring the moment: Differential impact on positive and negative outcomes. *Journal of Happiness Studies.* 2012;13(4):579-588.
30. Shiota MN, Campos B, Oveis C, Hertenstein MJ, Simon-Thomas E, Keltner D. Beyond happiness: Building a science of discrete positive emotions. *American Psychologist.* 2017;72(7):617-643.
31. Garland EL, Fredrickson B, Kring AM, Johnson DP, Meyer PS, Penn DL. Upward spirals of positive emotions counter downward spirals of negativity: insights from the broaden-and-build theory and affective neuroscience on the treatment of emotion dysfunctions and deficits in psychopathology. *Clinical psychology review.* 2010;30(7):849-864.
32. Pizzagalli DA, Bogdan R, Ratner KG, Jahn AL. Increased perceived stress is associated with blunted hedonic capacity: potential implications for depression research. *Behav Res Ther.* 2007;45(11):2742-2753.
33. Park MC, Goldman MA, Carpenter LL, Price LH, Friehs GM. Vagus nerve stimulation for depression: rationale, anatomical and physiological basis of efficacy and future prospects. *Acta Neurochir Suppl.* 2007;97(Pt 2):407-416.
34. Weytens F, Luminet O, Verhofstadt LL, Mikolajczak M. An integrative theory-driven positive emotion regulation intervention. *PloS One.* 2014;9(4):e95677.

© 2019 Ito M et al. *JAMA Network Open.*

35. Wadlinger HA, Isaacowitz DM. Fixing our focus: training attention to regulate emotion. *Pers Soc Psychol Rev.* 2011;15(1):75-102.
36. Peters KD, Constans JI, Mathews A. Experimental modification of attribution processes. *J Abnorm Psychol.* 2011;120(1):168-173.
37. O'Doherty JP. Reward representations and reward-related learning in the human brain: insights from neuroimaging. *Curr Opin Neurobiol.* 2004;14(6):769-776.
38. Epstein J, Pan H, Kocsis JH, et al. Lack of ventral striatal response to positive stimuli in depressed versus normal subjects. *The American journal of psychiatry.* 2006;163(10):1784-1790.
39. Keedwell PA, Andrew C, Williams SC, Brammer MJ, Phillips ML. The neural correlates of anhedonia in major depressive disorder. *Biological psychiatry.* 2005;58(11):843-853.
40. Wacker J, Dillon DG, Pizzagalli DA. The role of the nucleus accumbens and rostral anterior cingulate cortex in anhedonia: integration of resting EEG, fMRI, and volumetric techniques. *Neuroimage.* 2009;46(1):327-337.
41. Wichers MC, Barge-Schaapveld Dq Fau - Nicolson NA, Nicolson Na Fau - Peeters F, et al. Reduced stress-sensitivity or increased reward experience: the psychological mechanism of response to antidepressant medication. 2008(1740-634X (Electronic)).
42. Layous K, Kurtz J, Chancellor J, Lyubomirsky S. Reframing the ordinary: Imagining time as scarce increases well-being. *J Posit Psychol.* 2018;13(3):301-308.
43. Peterson C, Seligman MEP. *Character strengths and virtues: A handbook and classification.* New York, NY, US: Oxford University Press; 2004.
44. Charles ST. Strength and vulnerability integration: a model of emotional well-being across adulthood. *Psychological bulletin.* 2010;136(6):1068-1091.
45. Malterud K, Solvang P. Vulnerability as a strength: why, when, and how? *Scand J Public Health Suppl.* 2005;66:3-6.
46. Lambert C, Da Silva S, Ceniti AK, Rizvi SJ, Foussias G, Kennedy SH. Anhedonia in depression and schizophrenia: A transdiagnostic challenge. *CNS neuroscience & therapeutics.* 2018;24(7):615-623.
47. Sedikides C, Wildschut T, Arndt J, Routledge C. Nostalgia: Past, Present, and Future. *Current Directions in Psychological Science.* 2008;17(5):304-307.
48. Seligman ME, Steen TA, Park N, Park N, Peterson C. Positive psychology progress: empirical validation of interventions. 2005;60(5):410-421.
49. Proyer RT, Gander F, Wellenzohn S, Ruch W. Strengths-based positive psychology interventions: a randomized placebo-controlled online trial on long-term effects for a signature strengths- vs. a lesser strengths-intervention. *Front Psychol.* 2015;6:456.
50. Ishihara M, Saito T, Sakurai T, Shimada H, Arai H. Effect of a positive photo appreciation program on depressive mood in older adults: a pilot randomized controlled trial. 2018;15(7):1472. doi: 10.3390/ijerph15071472.
51. Lyubomirsky S, Sousa L, Dickerhoof R. The costs and benefits of writing, talking, and thinking about life's triumphs and defeats. *J Pers Soc Psychol.* 2006;90(4):692-708.

52. Pulcu E, Trotter PD, Thomas EJ, et al. Temporal discounting in major depressive disorder. *Psychol Med.* 2014;44(9):1825-1834.
53. Takahashi T, Oono H, Inoue T, et al. Depressive patients are more impulsive and inconsistent in intertemporal choice behavior for monetary gain and loss than healthy subjects--an analysis based on Tsallis' statistics. *Neuro Endocrinol Lett.* 2008;29(3):351-358.
54. Taquet M, Quoidbach J, de Montjoye YA, Desseilles M, Gross JJ. Hedonism and the choice of everyday activities. *Proc Natl Acad Sci U S A.* 2016;113(35):9769-9773.
55. Treadway MT, Bossaller NA, Shelton RC, Zald DH. Effort-based decision-making in major depressive disorder: a translational model of motivational anhedonia. *J Abnorm Psychol.* 2012;121(3):553-558.
56. Depression in context: Strategies for guided action [press release]. New York, NY, US: W W Norton & Co2001.
57. Hopko DR, Armento Me Fau - Cantu MS, Cantu Ms Fau - Chambers LL, Chambers Ll Fau - Lejuez CW, Lejuez CW. The use of daily diaries to assess the relations among mood state, overt behavior, and reward value of activities. 2003(0005-7967 (Print)).
58. Nelson SK, Fuller JA, Choi I, Lyubomirsky S. Beyond self-protection: self-affirmation benefits hedonic and eudaimonic well-being. *Pers Soc Psychol Bull.* 2014;40(8):998-1011.
59. Park N, Peterson C, Ruch W. Orientations to happiness and life satisfaction in twenty-seven nations. *J Posit Psychol.* 2009;4(4):273-279.
60. Csikszentmihalyi M. *Flow. The Psychology of Optimal Experience*. New York, NY: HarperPerennial; 1990.
61. Sheldon KM, Kasser T, Smith K, Share T. Personal goals and psychological growth: testing an intervention to enhance goal attainment and personality integration. *J Pers.* 2002;70(1):5-31.
62. Wood AM, Froh JJ, Geraghty AW. Gratitude and well-being: a review and theoretical integration. *Clinical psychology review.* 2010;30(7):890-905.
63. Otake K, Shimai S, Tanaka-Matsumi JU, Otsui K, Fredrickson BL. Happy people become happier through kindness: a counting kindnesses intervention. *J Happiness Stud.* 2006;7(3):361-375.
64. Beck JS, Beck AT. *Cognitive therapy: Basics and beyond.* Guilford press New York; 1995.

© 2019 Ito M et al. *JAMA Network Open.*
